# Synthesis of Cucurbitacin B Derivatives as Potential Anti-Hepatocellular Carcinoma Agents

**DOI:** 10.3390/molecules23123345

**Published:** 2018-12-18

**Authors:** Weizhi Ge, Xinyi Chen, Fangzhi Han, Zhongquan Liu, Tianpeng Wang, Mengmeng Wang, Yue Chen, Yahui Ding, Quan Zhang

**Affiliations:** 1 State Key Laboratory of Medicinal Chemical Biology, College of Pharmacy and Tianjin Key Laboratory of Molecular Drug Research, Nankai University, Tianjin 300353, China; geweizhi@aliyun.com (W.G.); chenxinyinku@126.com (X.C.); hfz904529505@163.com (F.H.); lzq9734@163.com (Z.L.); wangtianpeng888@126.com (T.W.); 2 Accendatech Company, Ltd., Tianjin 300384, China; mengmeng.wang@accendatech.com

**Keywords:** Cucurbitacin B, Anti-hepatocellular carcinoma, Derivative, Toxicity, Synthesis

## Abstract

Cucurbitacin B shows potent activity against tumor cells, but its high toxicity limits its application in the clinic. A series of cucurbitacin B derivatives was synthesized and evaluated for their anti-hepatocellular carcinoma (HCC) activities against the HepG-2 cell line. These compounds were also tested for their toxicity against the L-O2 normal cell line. The compound with the most potential, **10b**, exhibited potent activity against the HepG-2 cell line with an IC_50_ value of 0.63 μM. Moreover, compound **10b** showed the highest TI value (4.71), which is a 14.7-fold improvement compared to its parent compound cucurbitacin B. A preliminary molecular mechanism study of **10b** indicated that **10b** could inhibit P-STAT3 to induce the activation of mitochondrial apoptotic pathways. An in vivo acute toxicity study indicated that the compound **10b** has preferable safety and tolerability compared with cucurbitacin B. These findings indicate that compound **10b** might be considered as a lead compound for exploring effective anti-HCC drugs.

## 1. Introduction

Cancer is a disease with high morbidity and mortality. It is the second leading cause of death, after cardiovascular disease, worldwide [1,2]. The World Health Organization has reported that there were approximately 17.5 million new cancer cases and more than 8.7 million cancer-related deaths worldwide in 2015, and cancer-related deaths are expected to increase to 13.1 million by 2030 [3,4,5]. Cancer is a type of abnormal and excessive growth of tissue, in which abnormal body cells begin to divide uncontrollably by ignoring the principles of normal cell division. Cancer cells invade or spread to other healthy parts of the body, resulting in the formation of new tumor; this process is called metastasis. Approximately 90% of cancer-linked deaths are caused by it [5,6,7]. So far, cancer treatments mostly rely on traditional chemotherapy, especially for late-stage or complex cancers. Unfortunately, side effects and acquired drug resistance limit the effectiveness of traditional chemotherapy. Side effects, such as extreme fatigue and leukopenia, are caused by traditional chemotherapy’s low selectivity towards cancer cells and normal cells. These unpleasant side effects seriously reduce the life quality of cancer patients [8,9,10]. Therefore, the development of new chemotherapeutic drugs with high efficiency and low-toxicity side effects is a great challenge for medicinal chemists. Natural compounds can induce apoptosis by targeting multiple cellular signaling pathways; so, the development of new pharmacologically effective chemotherapeutic agents from natural compounds may be feasible [11,12,13].

**Figure 1:**
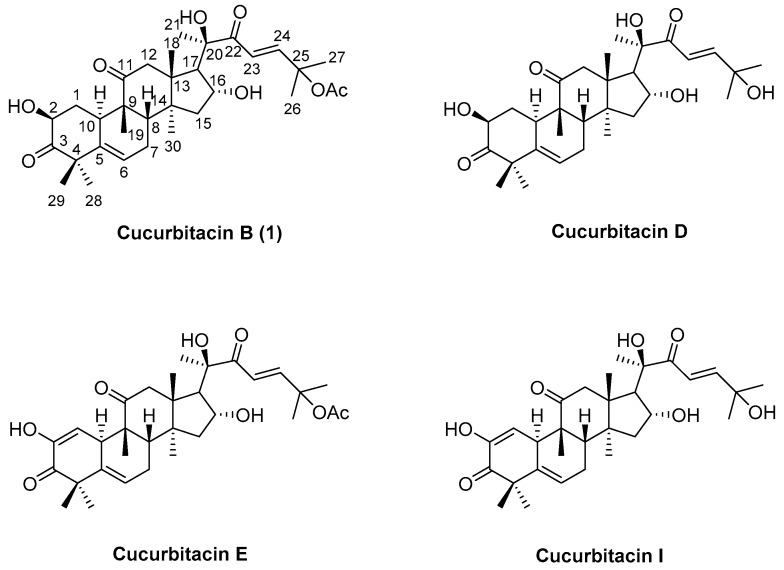
The chemical structures of cucurbitacin B (**1**), cucurbitacin D, cucurbitacin E, and cucurbitacin I.

Cucurbitacins are highly oxygenated tetracyclic and bitter triterpenes that are widely present in plants of the *Cucurbitaceae* family [14,15]. Cucurbitacins have for centuries been exploited for their anti-inflammatory and hepatoprotective activities as traditional herbal medicines in Asia [16]. Cucurbitacins have more than 200 derivatives and are divided into 12 classes, from cucurbitacin A to T, which have been classified according to structural features in ring A, side chain modifications, stereochemistry considerations, and glycosylated forms [14,17,18]. Cucurbitacins have been the subject of extensive scientific research due to their broad range of pharmacological effects, including anti-inflammatory, antitumor, antidiabetic, antifertility, antiviral, and anticancer activities [19,20,21,22,23]. Cucurbitacin B, D, E, and I (Figure 1) are the most active anticancer cucurbitacin derivatives [14,24]. Recent studies have revealed that cucurbitacins can cause cell-cycle arrest, apoptosis, and the suppression of cancer cell growth through inhibition of the JAK-STAT3, Wnt, PI3K/Akt, and MAPK signaling pathways, which play important roles in the apoptosis and survival of cancer cells [14,25].

Cucurbitacin B (**1**) is one of the most abundant forms of cucurbitacins, and one of the best-studied compounds of the cucurbitacins family [26,27,28,29,30]. Cucurbitacin B possesses a variety of bioactivities, such as anti-inflammatory, hepatoprotective, and anticancer activities. It has demonstrated potent anticancer activities in solid tumors in vitro and in vivo [31,32]. Cucurbitacin B is capable of inhibiting the growth of a wide spectrum of malignant human cells, such as laryngeal squamous cell carcinoma, breast cancer, pancreatic cancer, hepatocellular carcinoma (HCC), lung cancer, and melanoma cells [28,33,34,35,36]. The mechanism underlying the anticancer effect of cucurbitacin B is not clear; however, several studies have shown that cucurbitacin B inhibits the proliferation of, and induces apoptosis in, human cancer cells via STAT3 pathway inhibition and increased intracellular reactive oxygen species (ROS) [29,36,37,38,39,40].

At present, the main problem with cucurbitacin B is its high toxicity, which renders it unlikely to be a drug-able agent. Its low selectivity and narrow treatment window severely limit its clinical application. With cucurbitacin B as the starting substrate, we designed and synthesized a series of cucurbitacin B derivatives for the exploration of structure–activity relationships and the discovery of potential anti-HCC agents with potent anti-HCC activity and low toxicity.

## 2. Results and Discussion

### 2.1. Chemistry

**Scheme 1:**
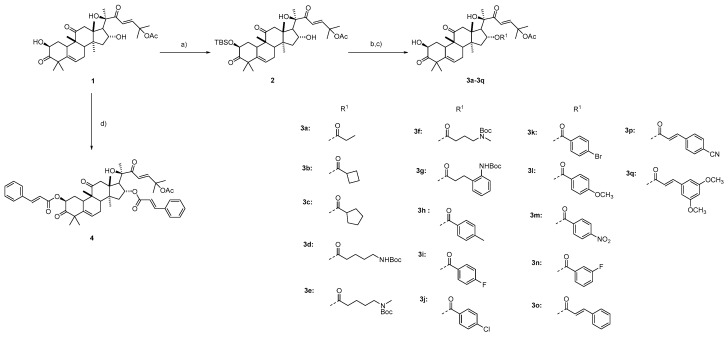
The synthesis of compounds **3a**–**3q** and **4**. Reagents and conditions: a) TBSCl, imidazole, DCM, 43%; b) R^1^COOH, EDCI, TEA, DMAP, DCM; c) TBAF/AcOH, THF, 47–86% yield in two steps; d) Cinnamic acid, EDCI, TEA, DMAP, DCM, 90%.

The starting material cucurbitacin B was isolated from *Cucumis melo* L. The presence of free hydroxyl groups allowed us to prepare ester derivatives of cucurbitacin B for an evaluation of the influence of the ester side chain on their anti-HCC activities. It was reported that the introduction of an acyl group to a 2-hydroxyl moiety greatly reduced the anticancer activity [41]. We planned to explore the effect of selective acylation on a 16-hydroxyl group on the anti-HCC activity. However, a 2-hydroxyl group is more reactive than a 16-hydroxyl group for acylation [41]. Therefore, we first designed protection for the 2-hydroxyl group. As shown in Scheme 1, the treatment of compound **1** with *t*-butyldimethylsilyl chloride (TBSCl) in the presence of imidazole selectively provided the 2-TBS-protected derivative **2** without the influence of the hydroxyl group linked to C20 of the cucurbicin B. The hydroxyl group linked to C20 is a tertiary hydroxyl group that shows a higher steric hindrance effect than the C16 hydroxyl group. Therefore, the C16 hydroxyl group was selectively esterified without affecting the hydroxyl group linked to C20 of the cucurbicin B. Compound **2** was coupled with different patterns of carboxylic acids in the presence of 1-ethyl-3-(3-dimethyllaminopropyl)carbodiimide hydrochloride (EDCI) and 4-dimethylaminopyridine (DMAP), followed by removal of the TBS protection to afford the esters **3a**–**3q** in yields of 48–86%. Direct conjuation of compound **1** with cinnamic acid provided the derivative **4** in a high yield (90%).

**Scheme 2:**
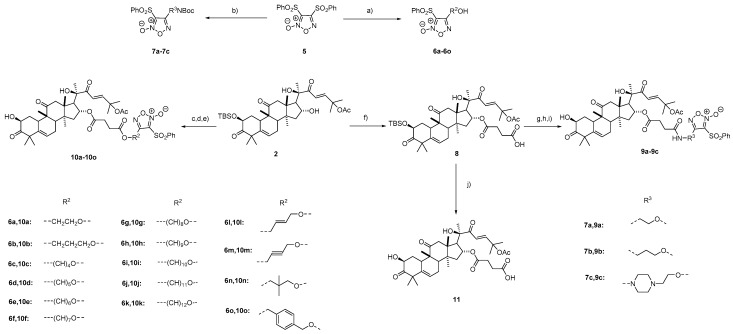
The synthesis of compounds **9a**–**9c**, **10a**–**10o**, and **11**. Reagents and conditions: a) Corresponding diol, 2N NaOH, THF; b) Corresponding alcohol amine, 2N NaOH, THF; c) **6a–6o**, succinic anhydride, DMAP, DCM; d) EDCI, TEA, DMAP, DCM; e) TBAF, AcOH, THF, 61–82% yield in two steps; f) succinic anhydride, DMAP, DCM; g) **7a**–**7c**, TFA, DCM; h) HATU, DIPEA, DMF; i) TBAF, AcOH, THF, 44–73% yield in three steps; j) TBAF, AcOH, THF, 79%.

It was reported that the introduction of a phenylsulfonyl-substituted furoxan NO-releasing moiety could reduce the toxicity of brusatol [42,43,44,45,46]. Accordingly, 18 cucurbitacin B derivatives, **9a**–**9c** and **10a**–**10o**, with the introduction of a phenylsulfonyl-substituted furoxan NO-releasing moiety were designed (Scheme 2). Compound **5** was prepared according to a reported procedure [42]. Reaction of **5** with different diols or alchoholamines gave **6a**–**6o** or **7a**–**7c**, respectively [42,43,44,46]. Compounds **6a**–**6o** were treated with succinic anhydride in the presence of DMAP to produce the corresponding acids, followed by conjuation with compound **2** and removal of the TBS group to yield **10a**–**10o** in yields of 61–82% in two steps. The acid **8** was obtained by the reaction of **2** and succinic anhydride. The removal of Boc groups of **7a**–**7c** gave the corresponding amines, followed by coupling with the acid **8** in the presence of 2-(7-azabenzotriazol-1-yl)-*N*,*N*,*N*′,*N*′-tetramethyluronium hexafluorophosphate (HATU) and *N*,*N*-diisopropylethylamine (DIPEA) and deprotection of the TBS group to afford **9a**–**9c** in yields of 44–73% in three steps. The removal of the TBS group of the acid **8** yielded compound **11** in a yield of 79%.

### 2.2. In Vitro Biological Activities against the HepG-2 Cell Line and the L-O2 Cell Line

The synthesized cucurbitacin B derivatives **3a**–**3q**, **4**, **9a**–**9c**, **10a**–**10o**, and **11** were evaluated for their activity against proliferation of the human hepatocellular carcinoma cell line HepG-2 and the normal hepatocellular cell line L-O2. Cucurbitacin B was also included for comparison. The therapeutic index (TI), a major pharmaceutical parameter that estimates possible future clinical development, was determined as the ratio of IC_50_ for L-O2 to IC_50_ for HepG-2. The bioactivity of each compound was evaluated by the combination of its IC_50_ and TI. These results are shown in Table 1 and Table 2.

The parent compound **1** exhibited potent activity against HepG-2 (IC_50_ = 0.06 µM), but appeared to have high toxicity (IC_50_ = 0.019 µM), which led to a very low therapeutic index (0.32). It was reported that the selective introduction of an acyl group to a 2-hydroxyl moiety greatly reduced the anticancer activity [41]. To further explore the influence of introducing different acyl moieties to a 16-hydroxyl group on anti-HCC activity, we synthesized the 16-mono-acylated derivatives **3a**–**3q**. For subseries of **3a**–**3q**, all compounds showed decreased cytotoxicity against the normal hepatocellular cell line L-O2 (IC_50_ = 0.0422–9.59 µM) compared to the parent compound **1** (IC_50_ = 0.019 µM), which indicated that the introduction of an acyl moiety to a 16-hydroxyl group could reduce the toxicity to normal cells. However, their anti-HCC activities against HepG-2 cells were also greatly decreased, with IC_50_ values ranging from 0.22 µM to 9.62 µM. Among subseries **3a**–**3q**, compound **3o** with a 16-mono-cinnamyl substitution showed the highest TI value (TI = 2.9). Therefore, we further synthesized compound **4** with a 2,16-di-cinnamyl substitution for testing its anti-HCC activity. Unfortunately, compound **4** lost inhibitory activity against HepG-2 (IC_50_ > 20 µM).

Considering that the introduction of a phenylsulfonyl-substituted furoxan NO-releasing moiety could reduce the toxicity of brusatol [42,43,44,45], derivatives **9a**–**9c** were synthesized and evaluated for their anti-HCC activity. As shown in Table 2, compounds **9a**–**9c** were less toxic than the parent compound **1** and showed improved TI values (ranging from 1.5 to 2.84) compared to compound **1** (TI = 0.32). These results prompted us to further synthesize more derivatives with a phenylsulfonyl-substituted furoxan NO-releasing moiety, i.e., compounds **10a**–**10o**. They featured with a different linker between cucurbitacin B and the phenylsulfonyl-substituted furoxan NO-releasing moiety. Compounds **10a**–**10o** exhibited potent or moderate activity against HepG-2 cells. Importantly, their cytotoxicity against L-O2 was significantly reduced compared to their parent compound cucurbitacin B, with IC_50_ values ranging from 1.17 to 3.31 µM. The compound with the most potential, **10b**, showed the highest TI value (4.71), which is a 14.7-fold improvement compared to its parent compound cucurbitacin B. Moreover, it still maintained potent activity against HepG-2 cells, with an IC_50_ value of 0.63 µM. These results suggested that the introduction of a phenylsulfonyl-substituted furoxan NO-releasing moiety could reduce the toxicity against normal cells and maintain potent anti-HCC activity.

The compound with the most potential, **10b**, was selected for a preliminary study on its mechanism of action.

### 2.3. Preliminary Study of Compound ***10b***’s Mechanism of Action

**Figure 2:**
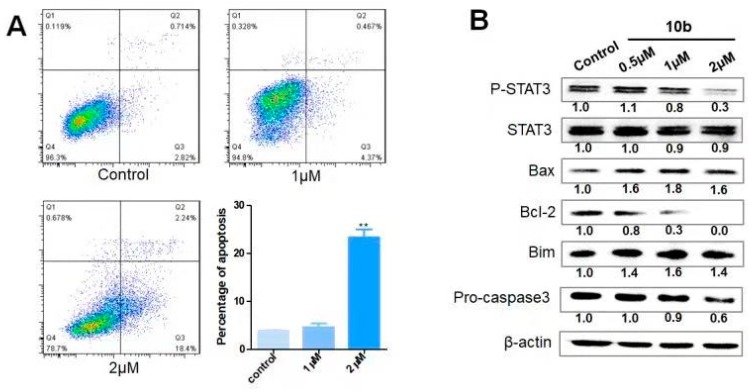
(**A**) The representative images and the statistical results of flow cytometry after treatment with compound **10b** at 0, 1, and 2 µM in a cell apoptosis assay. ** *p* < 0.01. (**B**) The level of STAT3, P-STAT3, Bax, Bim, Bcl-2, and caspase 3 after treatment with compound **10b** at different concentrations by a Western blot assay.

To determine whether the cytotoxic effect of compound **10b** was associated with apoptosis, a cell apoptosis assay was performed by flow cytometry. As shown in Figure 2A, compound **10b** significantly induced cell apoptosis in a dose-dependent manner. To further analyze the mechanism by which compound **10b** induces apoptosis, a Western blot assay was performed. STAT3 is associated with the expression of its downstream mitochondrial proteins, such as Bcl-2, Bax, and Bim, which are are critical regulators of cell apoptosis. As a result, we investigated the protein level of STAT3 and P-STAT3 using a Western blot analysis. As shown in Figure 2B, the total expression of STAT3 remained little changed. STAT3 is usually activated by phosphorylation to perform its functions. Inhibition of P-STAT3 plays an important role in cancer therapy. Our data showed that the level of P-STAT3 was clearly inhibited after treatment with compound **10b** in a dose-dependent manner. Moreover, the expression of the apoptotic-related members Bax and Bim were strongly increased, while the expression of the anti-apoptotic-related member Bcl-2 was substantially diminished after treatment with compound **10b**. As shown in Figure 1B, the level of caspase 3 was decreased. Accordingly, we proposed that compound **10b** inhibited P-STAT3 to induce mitochondrial-pathway-mediated apoptosis.

### 2.4. In Vivo Acute Toxicity Study of ***10b***

**Figure 3:**
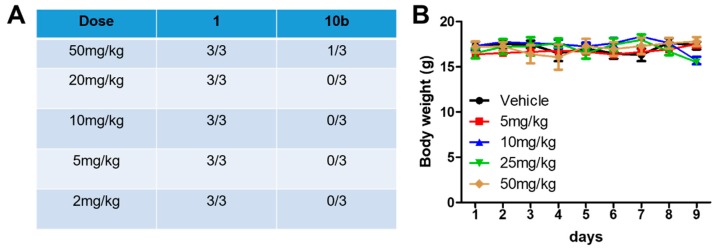
(**A**) The percentage of death in mice after the administration of cucurbitacin B and compound **10b**. (**B**) The body weight change after the administration of compound **10b** by intravenous injection.

To evaluate the toxicity of compound **10b**, an acute toxicity assay was performed using Balb/C mice. After the administration of cucurbitacin B or compound **10b** by intravenous injection, the clinical symptoms, deaths, and body weight were observed. As shown in Figure 3A, the percentage of death was 3/3, 3/3, 3/3, 3/3, and 3/3 after the administration of cucurbitacin B at a dose of 2 mg/kg, 5 mg/kg, 10 mg/kg, 20 mg/kg, and 50 mg/kg, respectively, while the percentage of death was 0/3, 0/3, 0/3, 0/3, and 1/3 after the administration of compound **10b** at a dose of 2 mg/kg, 5 mg/kg, 10 mg/kg, 20 mg/kg, and 50 mg/kg, respectively. Moreover, as shown in Figure 3B, the body weight did not change after the administration of compound **10b** at different doses as compared to the vehicle group. These results demonstrate that compound **10b** shows preferable safety and tolerability as compared with cucurbitacin B.

## 3. Experimental

### 3.1. Chemistry

#### 3.1.1. General Methods

Unless otherwise mentioned, all reactions were carried out under a nitrogen atmosphere with dry solvents under anhydrous conditions. Reactions were monitored by thin-layer chromatography carried out on 0.25 mm Tsingdao silica gel plates (60F-254). Visualization was achieved using UV light, phosphomolybdic acid in ethanol, or potassium permanganate in water, each followed by heating. Tsingdao silica gel (60, particle size 0.040–0.063 mm) was used for flash column chromatography. NMR spectra were recorded with a 400 MHz (^1^H: 400 MHz, ^13^C: 100 MHz) spectrometer. Data are reported as follows: chemical shift, multiplicity (s = singlet, d = doublet, t = triplet, q = quartet, br. = broad, m = multiplet), coupling constants, and integration. Purity testing was done by means of analytical HPLC on a Shimadzu LD-20A system with an ODS-C18 column (4.6 × 150 mm, 5 μm) eluted at 1–1.3 mL/min with Milli-Q water and CH_3_CN. The purity of all tested compounds was ≥95% by HPLC (Supplementary Materials).

#### 3.1.2. Preparation of Cucurbitacin B (**1**)

The starting material cucurbitacin B (**1**) was obtained from Pedicellus Melo in a yield of only 0.3%. (purity: 97%). ^1^H NMR (400 MHz, CDCl_3_) δ 7.04 (d, *J* = 15.6 Hz, 1H), 6.46 (d, *J* = 15.6 Hz, 1H), 5.77 (d, *J* = 5.3 Hz, 1H), 4.47–4.37 (m, 1H), 4.33 (dd, *J* = 14.7, 7.4 Hz, 1H), 4.26 (s, 1H), 3.61 (d, *J* = 3.8 Hz, 1H), 3.23 (d, *J* = 14.5 Hz, 1H), 2.72 (d, *J* = 12.8 Hz, 1H), 2.66 (d, *J* = 14.6 Hz, 1H), 2.48 (d, *J* = 7.0 Hz, 1H), 2.39 (dd, *J* = 19.4, 7.9 Hz, 1H), 2.29 (ddd, *J* = 12.3, 5.7, 3.3 Hz, 1H), 2.07–1.92 (m, 6H), 1.85 (t, *J* = 10.2 Hz, 2H), 1.55 (s, 3H), 1.53 (s, 3H), 1.45–1.39 (m, 4H), 1.34 (s, 3H), 1.32 (s, 3H), 1.26 (s, 3H), 1.06 (s, 3H), 0.96 (s, 3H). ^13^C NMR (100 MHz, CDCl_3_) δ 213.2, 212.3, 202.6, 170.3, 152.1, 140.5, 120.5, 120.4, 79.4, 78.3, 71.7, 71.3, 58.3, 50.8, 50.3, 48.7, 48.5, 48.2, 45.4, 42.5, 36.1, 33.8, 29.5, 26.5, 26.1, 24.0, 23.9, 22.1, 21.4, 20.2, 19.9, 18.9. HRMS (ESI) calcd. for C_32_H_46_NaO_8_ [M + Na]^+^ 581.3085, found 581.3088.

#### 3.1.3. Procedure for the Synthesis of Compounds **2**

*(6R,E)-6-((2S,9R,13R,14S,16R)-2-((tert-butyldimethylsilyl)oxy)-16-Hydroxy-4,4,9,13,14-pentamethyl-3,11-dioxo-2,3,4,7,8,9,10,11,12,13,14,15,16,17-tetradecahydro-1H-cyclopenta[a]phenanthren-17-yl)-6-hydroxy-2-methyl-5-oxohept-3-en-2-yl acetate* (**2**). To a solution of cucurbitacin B (**1**) (200.0 mg, 0.36 mmol) and TBSCl (80.9 mg, 0.54 mmol) in dry DCM (5 mL) was added imidazole (48.7 mg, 0.72 mmol) at 0 °C. The mixture was stirred for 4 h at room temperature. The reaction was quenched with saturated aqueous NH_4_Cl and extracted with CH_2_Cl_2_ (3 × 15 mL). The combined organic layers were washed with saturated brine, dried over Na_2_SO_4_, and concentrated to give an oily crude product, which was purified on a silica gel column to yield compound **2** (104 mg, 43.2%) as a white solid. ^1^H NMR (400 MHz, CDCl_3_) δ 7.04 (d, *J* = 15.6 Hz, 1H), 6.45 (d, *J* = 15.6 Hz, 1H), 5.73 (d, *J* = 5.1 Hz, 1H), 4.45 (dd, *J* = 12.7, 5.7 Hz, 1H), 4.34 (dd, *J* = 14.3, 7.1 Hz, 1H), 4.25 (s, 1H), 3.25 (d, *J* = 14.5 Hz, 1H), 2.69 (t, *J* = 14.6 Hz, 2H), 2.49 (d, *J* = 7.0 Hz, 1H), 2.38 (dd, *J* = 19.4, 7.8 Hz, 1H), 2.10–2.02 (m, 1H), 2.01–1.91 (m, 5H), 1.86 (dd, *J* = 13.0, 9.3 Hz, 1H), 1.77 (d, *J* = 6.5 Hz, 1H), 1.54 (d, *J* = 7.2 Hz, 6H), 1.42 (s, 4H), 1.34 (s, 3H), 1.27 (s, 3H), 1.24 (s, 1H), 1.22 (s, 3H), 1.06 (s, 3H), 0.97 (s, 3H), 0.88 (s, 9H), 0.11 (s, 3H), −0.02 (s, 3H). ^13^C NMR (100 MHz, CDCl_3_) δ 212.5, 210.7, 202.6, 170.3, 151.9, 140.5, 120.5, 119.9, 79.4, 78.4, 73.8, 71.4, 58.3, 51.1, 50.8, 48.9, 48.7, 48.3, 45.5, 42.6, 36.2, 34.5, 29.4, 26.5, 26.1, 25.9, 24.1, 23.9, 22.1, 21.8, 20.2, 19.9, 19.0, 18.6, −4.4, −5.4. HRMS (ESI) calcd. for C_38_H_60_NaO_8_ [M + Na]^+^ 695.3950, found 695.3954. 

#### 3.1.4. Procedure for the Synthesis of Compounds **3a**–**3q**

To a solution of compound **2** (100 mg, 0.15 mmol), EDCI (85 mg, 0.45 mmol), DMAP (1.2 mg, 0.01 mmol), and the corresponding acid (0.45 mmol, 3 eq) in CH_2_Cl_2_ (2 mL) was added Et_3_N (62.5 μL, 0.45 mmol) at 0 °C. The mixture was stirred for 8 h at room temperature. The reaction was quenched with saturated aqueous NaHCO_3_ and extracted with CH_2_Cl_2_ (3 × 15 mL). The combined organic layers were washed with saturated brine, dried over Na_2_SO_4_, and concentrated to give an oily crude product, which was simple purified to yield a white solid. The solid was used for the next step directly. The solid was dissolved in THF (2 mL). HOAc (38 μL, 0.65 mmol) and TBAF (169 mg, 0.65 mmol) were added to the mixture. The mixture was stirred at room temperature for 24 h, and then diluted with ethyl acetate (20 mL). The organic phase was washed with H_2_O (3 × 20 mL), dried over Na_2_SO_4_, and concentrated. The residue was purified by column chromatography on silica gel to obtain a white solid **3a**–**3q**.

*(2S,9R,13R,14S,16R)-17-((R,E)-6-Acetoxy-2-hydroxy-6-methyl-3-oxohept-4-en-2-yl)-2-hydroxy-4,4,9,13,14-pentamethyl-3,11-dioxo-2,3,4,7,8,9,10,11,12,13,14,15,16,17-tetradecahydro-1H-cyclopenta[a]phenanthren-16-yl propionate* (**3a**) (65% yield in two steps). (purity: 97%) ^1^H NMR (400 MHz, CDCl_3_) δ 7.12 (dd, *J* = 15.5, 2.0 Hz, 1H), 6.40 (dd, *J* = 15.6, 1.8 Hz, 1H), 5.76 (s, 1H), 5.17 (t, *J* = 7.7 Hz, 1H), 4.49–4.35 (m, 1H), 4.29 (s, 1H), 3.60 (s, 1H), 3.24 (d, *J* = 14.5 Hz, 1H), 2.71 (dd, *J* = 20.2, 10.3 Hz, 3H), 2.46–2.24 (m, 2H), 2.09 (dt, *J* = 7.5, 5.6 Hz, 2H), 2.05–1.97 (m, 5H), 1.71–1.51 (m, 6H), 1.40 (s, 3H), 1.37 (s, 1H), 1.32 (s, 3H), 1.29–1.22 (m, 9H), 1.07 (s, 3H), 1.04 (dd, *J* = 7.6, 2.0 Hz, 2H), 1.01 (s, 3H). ^13^C NMR (100 MHz, CDCl_3_) δ 213.0, 211.8, 201.0, 173.9, 169.7, 152.7, 140.5, 120.5, 119.4, 79.3, 77.8, 73.6, 71.7, 54.3, 50.3, 50.1, 48.7, 48.5, 48.1, 43.3, 42.2, 36.1, 33.8, 29.5, 27.3, 26.6, 26.5, 23.9, 23.8, 22.0, 21.4, 20.2, 19.8, 18.9, 9.1. HRMS (ESI) calcd. for C_35_H_50_NaO_9_ [M + Na]^+^ 637.3347, found 637.3350.

*(9R,13R,14S,16R)-17-((R,E)-6-Acetoxy-2-hydroxy-6-methyl-3-oxohept-4-en-2-yl)-2-hydroxy-4,4,9,13,14-pentamethyl-3,11-dioxo-2,3,4,7,8,9,10,11,12,13,14,15,16,17-tetradecahydro-1H-cyclopenta[a]phenanthren-16-yl cyclobutanecarboxylate* (**3b**) (86% yield in two steps). (purity: 95%). ^1^H NMR (400 MHz, CDCl_3_) δ 7.08 (d, *J* = 15.5 Hz, 1H), 6.41 (d, *J* = 15.5 Hz, 1H), 5.76 (s, 1H), 5.19 (t, *J* = 7.7 Hz, 1H), 4.40 (d, *J* = 8.1 Hz, 1H), 4.26 (s, 1H), 3.59 (s, 1H), 3.23 (d, *J* = 14.5 Hz, 1H), 2.99–2.83 (m, 1H), 2.71 (dd, *J* = 17.2, 10.4 Hz, 3H), 2.34 (dd, *J* = 38.6, 14.7 Hz, 2H), 2.22–2.12 (m, 2H), 2.08–1.96 (m, 7H), 1.93–1.83 (m, 2H), 1.58 (s, 6H), 1.40 (s, 3H), 1.32 (s, 3H), 1.26 (s, 9H), 1.07 (s, 3H), 1.00 (s, 3H). ^13^C NMR (100 MHz, CDCl_3_) δ 213.0, 211.8, 201.1, 174.9, 169.7, 152.4, 140.6, 120.5, 119.6, 79.3, 77.9, 73.6, 71.7, 54.3, 50.3, 50.2, 48.7, 48.5, 47.9, 43.5, 42.2, 37.8, 36.1, 33.8, 29.8, 29.5, 26.5, 25.1, 24.9, 23.9, 23.9, 22.0, 21.4, 20.2, 19.8, 18.8, 18.5. HRMS (ESI) calcd. for C_37_H_52_NaO_9_ [M + Na]^+^ 663.3504 found 663.3508. 

*(2S,9R,13R,14S,16R)-17-((R,E)-6-Acetoxy-2-hydroxy-6-methyl-3-oxohept-4-en-2-yl)-2-hydroxy-4,4,9,13,14-pentamethyl-3,11-dioxo-2,3,4,7,8,9,10,11,12,13,14,15,16,17-tetradecahydro-1H-cyclopenta[a]phenanthren-16-yl cyclopentanecarboxylate* (**3c**) (52% yield in two steps). (purity: 98%). ^1^H NMR (400 MHz, CDCl_3_) δ 7.08 (d, *J* = 15.6 Hz, 1H), 6.42 (d, *J* = 15.6 Hz, 1H), 5.75 (dd, *J* = 3.7, 1.9 Hz, 1H), 5.16 (t, *J* = 7.8 Hz, 1H), 4.39 (ddd, *J* = 12.8, 5.8, 3.9 Hz, 1H), 4.26 (s, 1H), 3.59 (d, *J* = 3.9 Hz, 1H), 3.23 (d, *J* = 14.6 Hz, 1H), 2.71 (dd, *J* = 12.8, 11.3 Hz, 3H), 2.53–2.44 (m, 1H), 2.39 (dd, *J* = 19.5, 7.9 Hz, 1H), 2.28 (ddd, *J* = 12.5, 5.8, 3.4 Hz, 1H), 2.04–1.86 (m, 6H), 1.81–1.59 (m, 6H), 1.56 (d, *J* = 2.2 Hz, 6H), 1.51–1.48 (m, 2H), 1.39 (s, 3H), 1.31 (s, 3H), 1.26 (d, *J* = 3.1 Hz, 6H), 1.23 (s, 2H), 1.06 (s, 3H), 0.99 (s, 3H). ^13^C NMR (100 MHz, CDCl_3_) δ 213.0, 211.8, 201.0, 176.1, 169.7, 152.5, 140.5, 120.5, 119.6, 79.3, 77.9, 73.6, 71.7, 54.2, 50.3, 50.1, 48.7, 48.5, 47.9, 43.6, 43.5, 42.2, 30.1, 29.5, 29.5, 26.5, 26.4, 25.8, 25.7, 23.9, 23.8, 21.9, 21.4, 20.1, 19.8, 18.8. HRMS (ESI) calcd. for C_38_H_54_NaO_9_ [M + Na]^+^ 677.3660, found 677.3665.

*(2S,9R,13R,14S,16R)-17-((R,E)-6-Acetoxy-2-hydroxy-6-methyl-3-oxohept-4-en-2-yl)-2-hydroxy-4,4,9,13,14-pentamethyl-3,11-dioxo-2,3,4,7,8,9,10,11,12,13,14,15,16,17-tetradecahydro-1H-cyclopenta[a]phenanthren-16-yl 5-((tert-butoxycarbonyl)amino)pentanoate* (**3d**) (67% yield in two steps). (purity: 95%). ^1^H NMR (400 MHz, CDCl_3_) δ 7.13 (d, *J* = 15.6 Hz, 1H), 6.38 (d, *J* = 15.6 Hz, 1H), 5.76 (d, *J* = 5.3 Hz, 1H), 5.17 (t, *J* = 8.0 Hz, 1H), 4.70 (s, 1H), 4.47–4.32 (m, 1H), 4.26 (s, 1H), 3.59 (d, *J* = 3.8 Hz, 1H), 3.23 (d, *J* = 14.6 Hz, 1H), 3.13–3.04 (m, 2H), 2.70 (dd, *J* = 19.5, 11.0 Hz, 3H), 2.39 (dd, *J* = 19.4, 7.7 Hz, 1H), 2.29 (ddd, *J* = 12.4, 5.7, 3.4 Hz, 1H), 2.10 (t, *J* = 7.2 Hz, 2H), 2.01 (s, 3H), 2.00–1.92 (m, 3H), 1.57 (s, 3H), 1.54 (s, 4H), 1.47–1.40 (m, 12H), 1.39 (s, 3H), 1.35 (s, 1H), 1.32 (s, 3H), 1.27 (d, *J* = 4.8 Hz, 6H), 1.23 (s, 1H), 1.07 (s, 3H), 1.00 (s, 3H). ^13^C NMR (100 MHz, CDCl_3_) δ 213.0, 211.7, 201.0, 172.9, 169.8, 156.1, 152.9, 140.5, 120.5, 119.4, 79.3, 77.8, 73.6, 71.7, 54.2, 50.3, 50.0, 48.7, 48.5, 48.1, 43.4, 42.2, 40.1, 36.1, 33.8, 33.3, 29.8, 29.5, 29.3, 28.5, 26.9, 26.4, 23.9, 23.8, 21.9, 21.8, 21.4, 20.2, 19.8, 18.9. HRMS (ESI) calcd. for C_42_H_63_NNaO_11_ [M + Na]^+^ 780.4293, found 780.4296. 

*(2S,9R,13R,14S,16R)-17-((R,E)-6-Acetoxy-2-hydroxy-6-methyl-3-oxohept-4-en-2-yl)-2-hydroxy-4,4,9,13,14-pentamethyl-3,11-dioxo-2,3,4,7,8,9,10,11,12,13,14,15,16,17-tetradecahydro-1H-cyclopenta[a]phenanthren-16-yl 5-((tert-butoxycarbonyl)(methyl)amino)pentanoate* (**3e**) (66% yield in two steps). (purity: 96%). ^1^H NMR (400 MHz, CDCl_3_) δ 7.11 (d, *J* = 15.6 Hz, 1H), 6.38 (d, *J* = 15.6 Hz, 1H), 5.75 (d, *J* = 5.4 Hz, 1H), 5.18 (t, *J* = 7.9 Hz, 1H), 4.44–4.35 (m, 1H), 4.26 (s, 1H), 3.59 (d, *J* = 3.8 Hz, 1H), 3.28–3.12 (m, 3H), 2.80 (s, 3H), 2.69 (dd, *J* = 20.6, 11.0 Hz, 3H), 2.39 (dd, *J* = 19.5, 7.8 Hz, 1H), 2.28 (ddd, *J* = 12.3, 5.6, 3.4 Hz, 1H), 2.10 (s, 2H), 2.03–1.86 (m, 7H), 1.55 (d, *J* = 5.6 Hz, 6H), 1.51–1.45 (m, 4H), 1.42 (s, 9H), 1.39 (s, 3H), 1.31 (s, 3H), 1.27 (s, 3H), 1.25 (s, 3H), 1.23 (s, 1H), 1.06 (s, 3H), 0.99 (s, 3H). ^13^C NMR (100 MHz, CDCl_3_) δ 212.9, 211.7, 200.9, 172.9, 169.6, 155.9, 152.7, 140.6, 120.4, 119.4, 79.2, 77.8, 73.5, 71.7, 54.3, 50.3, 50.0, 48.7, 48.5, 48.1, 47.9, 43.4, 42.2, 36.1, 34.2, 33.8, 33.5, 29.8,29.4, 28.6, 26.7, 26.5, 23.9, 23.8, 21.9, 21.9, 21.4, 20.1, 19.8, 18.9. HRMS (ESI) calcd. for C_43_H_65_NNaO_11_ [M + Na]^+^ 794.4450, found 794.4453. 

*(2S,9R,13R,14S,16R)-17-((R,E)-6-Acetoxy-2-hydroxy-6-methyl-3-oxohept-4-en-2-yl)-2-hydroxy-4,4,9,13,14-pentamethyl-3,11-dioxo-2,3,4,7,8,9,10,11,12,13,14,15,16,17-tetradecahydro-1H-cyclopenta[a]phenanthren-16-yl 4-((tert-butoxycarbonyl)(methyl)amino)butanoate* (**3f**) (64% yield in two steps). (purity: 98%). ^1^H NMR (400 MHz, CDCl_3_) δ 7.11 (d, *J* = 15.6 Hz, 1H), 6.39 (d, *J* = 15.6 Hz, 1H), 5.76 (d, *J* = 5.3 Hz, 1H), 5.20 (t, *J* = 8.0 Hz, 1H), 4.44–4.35 (m, 1H), 4.25 (s, 1H), 3.59 (d, *J* = 3.7 Hz, 1H), 3.28–3.12 (m, 3H), 2.82 (s, 3H), 2.70 (dd, *J* = 18.3, 11.0 Hz, 3H), 2.40 (dd, *J* = 19.4, 7.5 Hz, 1H), 2.30 (ddd, *J* = 12.3, 5.5, 3.3 Hz, 1H), 2.08 (s, 2H), 2.01 (s, 3H), 1.99 (d, *J* = 5.7 Hz, 2H), 1.77–1.69 (m, 2H), 1.56 (d, *J* = 5.9 Hz, 6H), 1.43 (s, 9H), 1.40 (s, 3H), 1.32 (s, 3H), 1.28 (s, 3H), 1.26 (s, 3H), 1.24 (s, 3H), 1.07 (s, 3H), 1.00 (s, 3H). ^13^C NMR (100 MHz, CDCl_3_) δ 212.9, 211.7, 201.1, 172.6, 169.7, 155.9, 152.8, 140.6, 120.5, 119.4, 79.2, 77.8, 73.7, 71.7, 54.3, 50.34, 50.1, 48.7, 48.5, 48.1, 43.3, 42.2, 36.1, 34.4, 33.9, 30.9, 29.8, 29.5, 28.6, 26.5, 23.9, 23.8, 21.9, 21.4, 20.2, 19.8, 18.9. HRMS (ESI) calcd. for C_42_H_63_NNaO_11_ [M + Na]^+^ 780.4293, found 780.4295. 

*(2S,9R,13R,14S,16R)-17-((R,E)-6-Acetoxy-2-hydroxy-6-methyl-3-oxohept-4-en-2-yl)-2-hydroxy-4,4,9,13,14-pentamethyl-3,11-dioxo-2,3,4,7,8,9,10,11,12,13,14,15,16,17-tetradecahydro-1H-cyclopenta[a]phenanthren-16-yl 3-(2-((tert-butoxycarbonyl)amino)phenyl)propanoate* (**3g**) (47% yield in two steps). (purity: 95%). ^1^H NMR (400 MHz, CDCl_3_) δ 7.65 (d, *J* = 7.7 Hz, 1H), 7.20–7.04 (m, 4H), 7.00 (t, *J* = 7.3 Hz, 1H), 6.34 (d, *J* = 15.6 Hz, 1H), 5.74 (d, *J* = 3.5 Hz, 1H), 5.15 (t, *J* = 7.9 Hz, 1H), 4.38 (d, *J* = 9.1 Hz, 1H), 4.25 (s, 1H), 3.61 (s, 1H), 3.19 (d, *J* = 14.5 Hz, 1H), 2.81 (dt, *J* = 23.3, 7.6 Hz, 2H), 2.69 (d, *J* = 14.2 Hz, 2H), 2.61 (d, *J* = 7.3 Hz, 1H), 2.44 (t, *J* = 7.7 Hz, 2H), 2.36 (dd, *J* = 20.4, 7.5 Hz, 1H), 2.27 (d, *J* = 11.7 Hz, 1H), 1.95 (s, 3H), 1.94–1.78 (m, 3H), 1.53 (d, *J* = 9.1 Hz, 3H), 1.49 (s, 12H), 1.36 (s, 3H), 1.31 (s, 3H), 1.24 (s, 5H), 1.11 (s, 3H), 1.04 (s, 3H), 0.96 (s, 3H). ^13^C NMR (100 MHz, CDCl_3_) δ 212.95, 211.70, 200.86, 173.16, 169.85, 153.82, 153.04, 140.37, 136.13, 129.31, 127.00, 124.28, 120.35, 119.09, 80.19, 79.15, 77.67, 74.02, 71.64, 65.61, 54.07, 50.28, 49.93, 48.55, 48.36, 47.92, 42.97, 42.07, 35.99, 34.12, 33.71, 29.36, 28.45, 27.06, 26.18, 25.59, 23.75, 23.57, 21.83, 21.34, 20.08, 19.69, 18.59. HRMS (ESI) calcd. for C_46_H_63_NNaO_11_ [M + Na]^+^ 828.4293, found 828.4296. 

*(2S,9R,13R,14S,16R)-17-((R,E)-6-Acetoxy-2-hydroxy-6-methyl-3-oxohept-4-en-2-yl)-2-hydroxy-4,4,9,13,14-pentamethyl-3,11-dioxo-2,3,4,7,8,9,10,11,12,13,14,15,16,17-tetradecahydro-1H-cyclopenta[a]phenanthren-16-yl 4-methylbenzoate* (**3h**) (79% yield in two steps). (purity: 97%). ^1^H NMR (400 MHz, CDCl_3_) δ 7.75 (d, *J* = 8.0 Hz, 2H), 7.16 (d, *J* = 7.9 Hz, 2H), 6.87 (d, *J* = 15.5 Hz, 1H), 6.51 (d, *J* = 15.5 Hz, 1H), 5.74 (d, *J* = 4.2 Hz, 1H), 5.54 (t, *J* = 7.8 Hz, 1H), 4.42 (d, *J* = 10.8 Hz, 1H), 4.19 (s, 1H), 3.61 (d, *J* = 2.2 Hz, 1H), 3.29 (d, *J* = 14.5 Hz, 1H), 2.89 (d, *J* = 7.2 Hz, 1H), 2.74 (d, *J* = 18.2 Hz, 2H), 2.47 – 2.31 (m, 4H), 2.10 (dd, *J* = 13.6, 9.1 Hz, 1H), 2.03 (d, *J* = 12.0 Hz, 5H), 1.46 (s, 6H), 1.40 (s, 3H), 1.37 (s, 3H), 1.31 (s, 3H), 1.25 (d, *J* = 8.9 Hz, 6H), 1.09 (s, 3H), 1.06 (s, 3H). ^13^C NMR (100 MHz, CDCl_3_) δ 213.0, 211.9, 201.3, 169.8, 165.9, 152.5, 143.8, 140.5, 129.7, 129.1, 127.4, 120.5, 119.5, 79.5, 78.2, 74.1, 71.7, 54.8, 50.4, 48.7, 48.5, 48.0, 43.6, 42.3, 36.1, 33.8, 32.0, 29.8, 29.5, 26.6, 25.8, 24.2, 23.9, 22.8, 22.1, 21.8, 21.4, 20.2, 19.8, 18.8. HRMS (ESI) calcd. for C_40_H_52_NaO_9_ [M + Na]^+^ 699.3504, found 699.3508. 

*(2S,9R,13R,14S,16R)-17-((R,E)-6-Acetoxy-2-hydroxy-6-methyl-3-oxohept-4-en-2-yl)-2-hydroxy-4,4,9,13,14-pentamethyl-3,11-dioxo-2,3,4,7,8,9,10,11,12,13,14,15,16,17-tetradecahydro-1H-cyclopenta[a]phenanthren-16-yl 4-fluorobenzoate* (**3i**) (81% yield in two steps). (purity: 98%). ^1^H NMR (400 MHz, CDCl_3_) δ 7.96–7.82 (m, 2H), 7.05 (t, *J* = 8.4 Hz, 2H), 6.90 (d, *J* = 15.6 Hz, 1H), 6.48 (d, *J* = 15.6 Hz, 1H), 5.74 (d, *J* = 3.6 Hz, 1H), 5.55 (t, *J* = 7.8 Hz, 1H), 4.42 (dd, *J* = 12.7, 5.7 Hz, 1H), 4.14 (s, 1H), 3.61 (s, 1H), 3.29 (d, *J* = 14.6 Hz, 1H), 2.87 (d, *J* = 7.3 Hz, 1H), 2.78 (d, *J* = 14.7 Hz, 1H), 2.73 (d, *J* = 12.4 Hz, 1H), 2.41 (dd, *J* = 18.9, 6.5 Hz, 1H), 2.35–2.27 (m, 1H), 2.19–2.07 (m, 1H), 2.03 (d, *J* = 10.1 Hz, 4H), 1.97–1.86 (m, 1H), 1.46 (s, 6H), 1.43 (s, 3H), 1.36 (s, 3H), 1.31 (s, 3H), 1.26 (s, 3H), 1.24 (s, 2H), 1.09 (s, 3H), 1.06 (s, 3H). ^19^F NMR (376 MHz, CDCl_3_) δ −105.30–−105.40 (m). ^13^C NMR (100 MHz, CDCl_3_) δ 213.0, 211.7, 201.3, 169.7, 165.9 (d, *J* = 254.2 Hz), 164.9, 152.8, 140.5, 132.2 (d, *J* = 9.2 Hz), 131.1, 128.9, 126.3 (d, *J* = 2.7 Hz), 120.4, 119.3, 115.6 (d, *J* = 22.0 Hz), 79.4, 78.1, 74.5, 71.7, 54.6, 50.4, 50.3, 48.7, 48.5, 48.0, 43.6, 42.2, 36.1, 33.8, 29.4, 26.7, 26.1, 24.1, 23.9, 22.1, 21.4, 20.2, 19.8, 18.9. HRMS (ESI) calcd. for C_39_H_49_FNaO_9_ [M + Na]^+^ 703.3253, found 703.3258.

*(2S,9R,13R,14S,16R)-17-((R,E)-6-Acetoxy-2-hydroxy-6-methyl-3-oxohept-4-en-2-yl)-2-hydroxy-4,4,9,13,14-pentamethyl-3,11-dioxo-2,3,4,7,8,9,10,11,12,13,14,15,16,17-tetradecahydro-1H-cyclopenta[a]phenanthren-16-yl 4-chlorobenzoate* (**3j**) (69% yield in two steps). (purity: 95%). ^1^H NMR (400 MHz, CDCl_3_) δ 7.79 (d, *J* = 8.3 Hz, 2H), 7.34 (d, *J* = 8.3 Hz, 2H), 6.88 (d, *J* = 15.6 Hz, 1H), 6.48 (d, *J* = 15.6 Hz, 1H), 5.73 (s, 1H), 5.55 (t, *J* = 7.8 Hz, 1H), 4.41 (dd, *J* = 12.7, 5.7 Hz, 1H), 3.28 (d, *J* = 14.5 Hz, 1H), 2.87 (d, *J* = 7.2 Hz, 1H), 2.81–2.67 (m, 2H), 2.50–2.26 (m, 2H), 2.16–2.06 (m, 1H), 2.02 (d, *J* = 10.9 Hz, 4H), 1.97–1.86 (m, 2H), 1.45 (s, 6H), 1.42 (s, 3H), 1.35 (s, 3H), 1.30 (s, 3H), 1.25 (s, 3H), 1.24 (s, 3H), 1.08 (s, 3H), 1.05 (s, 3H). ^13^C NMR (100 MHz, CDCl_3_) δ 212.9, 211.7, 201.3, 169.7, 165.0, 152.8, 140.5, 139.6, 131.1, 128.8, 120.4, 119.4, 79.4, 78.1, 74.6, 71.7, 54.6, 50.3, 50.3, 48.6, 48.4, 48.0, 43.5, 42.2, 36.1, 33.8, 29.4, 26.6, 26.1, 24.1, 23.9, 22.0, 21.3, 20.2, 19.8, 18.9. HRMS (ESI) calcd. for C_39_H_49_ClNaO_9_ [M + Na]^+^ 719.2957, found 719.2962.

*(2S,9R,13R,14S,16R)-17-((R,E)-6-Acetoxy-2-hydroxy-6-methyl-3-oxohept-4-en-2-yl)-2-hydroxy-4,4,9,13,14-pentamethyl-3,11-dioxo-2,3,4,7,8,9,10,11,12,13,14,15,16,17-tetradecahydro-1H-cyclopenta[a]phenanthren-16-yl 4-bromobenzoate* (**3k**) (64% yield in two steps). (purity: 95%). ^1^H NMR (400 MHz, CDCl_3_) δ 7.73 (d, *J* = 8.5 Hz, 2H), 7.52 (d, *J* = 8.5 Hz, 2H), 6.90 (d, *J* = 15.6 Hz, 1H), 6.47 (d, *J* = 15.6 Hz, 1H), 5.74 (d, *J* = 5.4 Hz, 1H), 5.55 (t, *J* = 7.9 Hz, 1H), 4.42 (dd, *J* = 12.9, 5.9 Hz, 1H), 4.13 (d, *J* = 7.0 Hz, 1H), 3.29 (d, *J* = 14.7 Hz, 1H), 2.87 (d, *J* = 7.4 Hz, 1H), 2.76 (dd, *J* = 21.3, 13.9 Hz, 2H), 2.47–2.28 (m, 2H), 2.16–2.07 (m, 1H), 2.06–2.01 (m, 4H), 1.95 (d, *J* = 3.4 Hz, 1H), 1.46 (d, *J* = 4.2 Hz, 6H), 1.44 (s, 3H), 1.36 (s, 3H), 1.31 (s, 3H), 1.27 (s, 3H), 1.24 (s, 3H), 1.09 (s, 3H), 1.06 (s, 3H). ^13^C NMR (100 MHz, CDCl_3_) δ 213.0, 211.7, 201.2, 169.8, 165.2, 152.9, 140.5, 131.8, 131.2, 128.9, 128.3, 120.4, 119.3, 79.4, 78.1, 74.7, 71.7, 54.6, 50.4, 50.3, 48.7, 48.5, 48.0, 43.5, 42.2, 36.1, 33.8, 29.4, 26.7, 26.1, 24.1, 23.9, 22.1, 21.4, 20.2, 19.8, 18.9. HRMS (ESI) calcd. for C_39_H_49_BrNaO_9_ [M + Na]^+^ 763.2452, found 763.2458.

*(2S,9R,13R,14S,16R)-17-((R,E)-6-Acetoxy-2-hydroxy-6-methyl-3-oxohept-4-en-2-yl)-2-hydroxy-4,4,9,13,14-pentamethyl-3,11-dioxo-2,3,4,7,8,9,10,11,12,13,14,15,16,17-tetradecahydro-1H-cyclopenta[a]phenanthren-16-yl 4-methoxybenzoate* (**3l**) (78% yield in two steps). (purity: 99%). ^1^H NMR (400 MHz, CDCl_3_) δ 7.82 (d, *J* = 8.8 Hz, 2H), 6.87 (t, *J* = 12.0 Hz, 3H), 6.51 (d, *J* = 15.6 Hz, 1H), 5.74 (d, *J* = 5.4 Hz, 1H), 5.54 (t, *J* = 7.9 Hz, 1H), 4.42 (dd, *J* = 7.2, 3.2 Hz, 1H), 4.15 (s, 1H), 3.83 (s, 3H), 3.60 (d, *J* = 3.4 Hz, 1H), 3.29 (d, *J* = 14.6 Hz, 1H), 2.88 (d, *J* = 7.4 Hz, 1H), 2.78 (d, *J* = 14.7 Hz, 1H), 2.47–2.28 (m, 2H), 2.14–2.03 (m, 2H), 2.02 (s, 3H), 1.97–1.88 (m, 1H), 1.46 (d, *J* = 5.3 Hz, 6H), 1.42 (s, 3H), 1.37 (s, 3H), 1.32 (d, *J* = 5.5 Hz, 3H), 1.27 (d, *J* = 3.5 Hz, 3H), 1.25 (s, 3H), 1.09 (s, 3H), 1.06 (s, 3H). ^13^C NMR (100 MHz, CDCl_3_) δ 213.1, 211.9, 201.4, 169.8, 165.6, 163.6, 152.5, 140.6, 131.7, 122.6, 120.5, 119.6, 113.7, 79.5, 78.3, 74.0, 71.8, 55.6, 54.9, 50.4, 48.8, 48.5, 48.0, 43.6, 42.3, 36.1, 33.9, 29.8, 29.5, 26.7, 25.9, 24.3, 22.8, 22.1, 21.4, 20.2, 19.8, 18.9. HRMS (ESI) calcd. for C_40_H_52_BrNaO_10_ [M + Na]^+^ 715.3453, found 715.3458. 

*(2S,9R,13R,14S,16R)-17-((R,E)-6-Acetoxy-2-hydroxy-6-methyl-3-oxohept-4-en-2-yl)-2-hydroxy-4,4,9,13,14-pentamethyl-3,11-dioxo-2,3,4,7,8,9,10,11,12,13,14,15,16,17-tetradecahydro-1H-cyclopenta[a]phenanthren-16-yl 4-nitrobenzoate* (**3m**) (64% yield in two steps). (purity: 95%). ^1^H NMR (400 MHz, CDCl_3_) δ 8.24 (d, *J* = 8.7 Hz, 2H), 8.05 (d, *J* = 8.7 Hz, 2H), 6.94 (d, *J* = 15.6 Hz, 1H), 6.47 (d, *J* = 15.6 Hz, 1H), 5.74 (s, 1H), 5.61 (t, *J* = 7.9 Hz, 1H), 4.42 (d, *J* = 10.8 Hz, 1H), 4.12 (s, 1H), 3.60 (d, *J* = 2.8 Hz, 1H), 3.29 (d, *J* = 14.6 Hz, 1H), 2.90 (d, *J* = 7.4 Hz, 1H), 2.80 (d, *J* = 14.6 Hz, 1H), 2.73 (d, *J* = 12.8 Hz, 1H), 2.52 – 2.27 (m, 2H), 2.16 (dd, *J* = 14.1, 9.0 Hz, 1H), 2.07–1.98 (m, 4H), 1.48 (s, 3H), 1.47 (s, 3H), 1.37 (s, 3H), 1.31 (s, 3H), 1.27 (s, 3H), 1.25 (s, 6H), 1.10 (s, 3H), 1.08 (s, 3H). ^13^C NMR (100 MHz, CDCl_3_) δ 212.9, 211.5, 201.1, 169.8, 164.2, 153.4, 150.7, 140.7, 135.4, 130.9, 128.9, 123.7, 120.4, 119.2, 79.3, 77.9, 75.5, 71.7, 54.3, 50.4, 50.3, 48.6, 48.5, 48.1, 43.6, 42.2, 36.1, 33.9, 29.8, 29.5, 26.6, 24.0, 23.9, 22.0, 21.4, 20.2, 19.7, 19.0. HRMS (ESI) calcd. for C_39_H_49_NNaO_11_ [M + Na]^+^ 730.3198, found 730.3210. 

*(2S,9R,13R,14S,16R)-17-((R,E)-6-Acetoxy-2-hydroxy-6-methyl-3-oxohept-4-en-2-yl)-2-hydroxy-4,4,9,13,14-pentamethyl-3,11-dioxo-2,3,4,7,8,9,10,11,12,13,14,15,16,17-tetradecahydro-1H-cyclopenta[a]phenanthren-16-yl 3-fluorobenzoate* (**3n**) (80% yield in two steps). (purity: 95%). ^1^H NMR (400 MHz, CDCl_3_) δ 7.69 (d, *J* = 7.3 Hz, 1H), 7.51 (d, *J* = 8.3 Hz, 1H), 7.42–7.33 (m, 1H), 7.22 (d, *J* = 7.4 Hz, 1H), 6.89 (d, *J* = 15.5 Hz, 1H), 6.49 (d, *J* = 15.6 Hz, 1H), 5.74 (s, 1H), 5.57 (t, *J* = 7.5 Hz, 1H), 4.42 (d, *J* = 7.2 Hz, 1H), 4.19 (s, 1H), 3.60 (s, 1H), 3.30 (d, *J* = 14.4 Hz, 1H), 2.89 (d, *J* = 7.0 Hz, 1H), 2.85–2.70 (m, 2H), 2.41 (d, *J* = 19.3 Hz, 1H), 2.31 (s, 1H), 2.12 (t, *J* = 11.3 Hz, 1H), 2.03 (d, *J* = 15.4 Hz, 3H), 1.93 (d, *J* = 15.3 Hz, 1H), 1.46 (s, 6H), 1.42 (s, 3H), 1.37 (s, 3H), 1.31 (s, 3H), 1.26 (d, *J* = 10.4 Hz, 6H), 1.08 (d, *J* = 10.4 Hz, 6H). ^13^C NMR (100 MHz, CDCl_3_) δ 212.9, 211.6, 201.3, 169.7, 164.7, 162.5 (d, *J* = 247.4 Hz), 152.9, 140.6, 132.2 (d, *J* = 7.1 Hz), 130.2 (d, *J* = 7.8 Hz), 125.6 (d, *J* = 2.7 Hz), 120.4, 120.2 (d, *J* = 21.3 Hz), 119.4, 116.4 (d, *J* = 22.7 Hz), 79.4, 78.1, 74.8, 71.7, 54.6, 50.4, 48.7, 48.5, 48.1, 43.6, 42.3, 36.1, 33.9, 29.5, 26.7, 25.7, 24.1, 23.1, 22.1, 21.4, 20.2, 19.8, 18.9. ^19^F NMR (376 MHz, CDCl_3_) δ−112.09 (dd, *J* = 13.9, 8.1 Hz). HRMS (ESI) calcd. for C_39_H_49_FNaO_9_ [M + Na]^+^ 703.3253, found 703.3258. 

*(2S,9R,13R,14S,16R)-17-((R,E)-6-Acetoxy-2-hydroxy-6-methyl-3-oxohept-4-en-2-yl)-2-hydroxy-4,4,9,13,14-pentamethyl-3,11-dioxo-2,3,4,7,8,9,10,11,12,13,14,15,16,17-tetradecahydro-1H-cyclopenta[a]phenanthren-16-yl cinnamate* (**3o**) (81% yield in two steps). (purity: 97%). ^1^H NMR (400 MHz, CDCl_3_) δ 7.60 (d, *J* = 15.9 Hz, 1H), 7.52 (s, 2H), 7.35 (s, 3H), 7.07 (d, *J* = 15.5 Hz, 1H), 6.45 (d, *J* = 15.5 Hz, 1H), 6.32 (d, *J* = 16.0 Hz, 1H), 5.76 (s, 1H), 5.37 (t, *J* = 7.5 Hz, 1H), 4.41 (d, *J* = 7.7 Hz, 1H), 4.29 (s, 1H), 3.61 (s, 1H), 3.28 (d, *J* = 14.5 Hz, 1H), 2.85–2.66 (m, 3H), 2.48–2.26 (m, 2H), 2.03 (s, 2H), 1.97 (s, 3H), 1.52 (d, *J* = 11.6 Hz, 6H), 1.43 (s, 3H), 1.34 (s, 3H), 1.32 (s, 3H), 1.27 (s, 3H), 1.25 (s, 3H), 1.09 (s, 3H), 1.04 (s, 3H). ^13^C NMR (101 MHz, CDCl_3_) δ 213.0, 211.8, 200.9, 169.7, 166.5, 152.8, 145.4, 140.5, 134.5, 130.4, 128.9, 128.3, 120.5, 119.3, 117.8, 79.2, 77.9, 73.7, 71.7, 54.5, 50.3, 50.2, 48.7, 48.5, 48.1, 43.4, 42.3, 36.1, 33.9, 29.8, 29.4, 26.8, 26.5, 23.9, 21.9, 21.4, 20.2, 19.8, 18.9. HRMS (ESI) calcd. for C_41_H_52_NaO_9_ [M + Na]^+^ 711.3504, found 711.3507. 

*(2S,9R,13R,14S,16R)-17-((R,E)-6-Acetoxy-2-hydroxy-6-methyl-3-oxohept-4-en-2-yl)-2-hydroxy-4,4,9,13,14-pentamethyl-3,11-dioxo-2,3,4,7,8,9,10,11,12,13,14,15,16,17-tetradecahydro-1H-cyclopenta[a]phenanthren-16-yl (E)-3-(4-cyanophenyl)acrylate* (**3p**) (85% yield in two steps). (purity: 97%). ^1^H NMR (400 MHz, CDCl_3_) δ 7.65 (dd, *J* = 17.0, 8.1 Hz, 4H), 7.58 (d, *J* = 15.9 Hz, 1H), 7.11 (d, *J* = 15.5 Hz, 1H), 6.50 (d, *J* = 15.8 Hz, 1H), 6.41 (d, *J* = 15.6 Hz, 1H), 5.75 (d, *J* = 3.9 Hz, 1H), 5.36 (t, *J* = 8.1 Hz, 1H), 4.41 (dd, *J* = 12.4, 5.3 Hz, 1H), 4.29 (s, 1H), 3.61 (s, 1H), 3.27 (d, *J* = 14.6 Hz, 1H), 2.75 (dd, *J* = 19.3, 10.9 Hz, 3H), 2.48 – 2.27 (m, 2H), 2.02 (d, *J* = 8.0 Hz, 2H), 1.98 (s, 3H), 1.56 (s, 3H), 1.48 (s, 3H), 1.43 (s, 3H), 1.32 (d, *J* = 5.5 Hz, 6H), 1.26 (s, 3H), 1.24 (s, 3H), 1.09 (s, 3H), 1.05 (s, 3H). ^13^C NMR (100 MHz, CDCl_3_) δ 212.9, 211.7, 200.8, 169.7, 165.9, 153.1, 142.9, 140.6, 138.9, 132.6, 128.8, 121.6, 120.4, 119.1, 113.4, 79.2, 77.8, 74.2, 71.7, 54.2, 50.3, 50.1, 48.7, 48.5, 48.1, 43.4, 42.2, 36.1, 33.9, 29.8, 29.4, 27.3, 26.5, 23.9, 23.8, 21.9, 21.4, 20.2, 19.8, 18.9. HRMS (ESI) calcd. for C_42_H_51_NNaO_9_ [M + Na]^+^ 736.3456, found 736.3458.

*(2S,9R,13R,14S,16R)-17-((R,E)-6-Acetoxy-2-hydroxy-6-methyl-3-oxohept-4-en-2-yl)-2-hydroxy-4,4,9,13,14-pentamethyl-3,11-dioxo-2,3,4,7,8,9,10,11,12,13,14,15,16,17-tetradecahydro-1H-cyclopenta[a]phenanthren-16-yl (E)-3-(3,5-dimethoxyphenyl)acrylate* (**3q**) (82% yield in two steps). (purity: 95%). ^1^H NMR (400 MHz, CDCl_3_) δ 7.51 (d, *J* = 15.9 Hz, 1H), 7.07 (d, *J* = 15.6 Hz, 1H), 6.66 (d, *J* = 2.2 Hz, 2H), 6.45 (t, *J* = 9.2 Hz, 2H), 6.28 (d, *J* = 15.9 Hz, 1H), 5.75 (d, *J* = 5.5 Hz, 1H), 5.36 (t, *J* = 7.8 Hz, 1H), 4.41 (dd, *J* = 12.9, 6.0 Hz, 1H), 3.78 (s, 7H), 3.27 (d, *J* = 14.7 Hz, 1H), 2.82 – 2.68 (m, 3H), 2.48 – 2.26 (m, 2H), 2.11 – 1.94 (m, 6H), 1.53 (d, *J* = 3.2 Hz, 6H), 1.43 (s, 3H), 1.34 (s, 3H), 1.31 (s, 3H), 1.26 (s, 3H), 1.24 (s, 3H), 1.08 (s, 3H), 1.04 (s, 3H). ^13^C NMR (100 MHz, CDCl_3_) δ 213.0, 211.9, 200.9, 169.8, 166.4, 161.1, 152.9, 145.5, 140.5, 136.3, 120.5, 119.3, 118.2, 106.4, 102.5, 79.2, 77.9, 73.8, 71.7, 55.5, 54.4, 50.3, 50.2, 48.7, 48.5, 48.1, 43.4, 42.2, 36.1, 33.8, 29.8, 29.4, 26.8, 26.7, 23.9, 21.9, 21.4, 20.2, 19.8, 18.8. HRMS (ESI) calcd. for C_43_H_56_NaO_11_ [M + Na]^+^ 771.3715, found 771.3718.

#### 3.1.5. Procedure for the Synthesis of Compounds **4**

*(2S,9R,13R,14S,16R)-17-((R,E)-6-Acetoxy-2-hydroxy-6-methyl-3-oxohept-4-en-2-yl)-4,4,9,13,14-pentamethyl-3,11-dioxo-2,3,4,7,8,9,10,11,12,13,14,15,16,17-tetradecahydro-1H-cyclopenta[a]phenanthrene-2,16-diyl (2E,2′E)-bis(3-phenylacrylate)* (**4**). To a solution of compound **1** (100.0 mg, 0.18 mmol), EDCI (171.6 mg, 0.9 mmol), DMAP (1.2 mg, 0.01 mmol), and cinnamic acid (133.4 mg, 0.9 mmol) in CH_2_Cl_2_ (2 mL) was added Et_3_N (125 μL, 0.9 mmol) at 0 °C. The mixture was stirred for 8 h at room temperature. The reaction was quenched with saturated aqueous NaHCO_3_ and extracted with CH_2_Cl_2_ (3 × 15 mL). The combined organic layers were washed with saturated brine, dried over Na_2_SO_4_, and concentrated to give an oily crude product, which was purified on a silica gel column to yield compound **4** (112mg, yield: 76%) as a white solid. (purity: 95%). ^1^H NMR (400 MHz, CDCl_3_) δ 7.74 (d, *J* = 16.0 Hz, 1H), 7.61 (d, *J* = 15.9 Hz, 1H), 7.52 (s, 4H), 7.37 (d, *J* = 8.6 Hz, 6H), 7.08 (d, *J* = 15.5 Hz, 1H), 6.49 (t, *J* = 15.0 Hz, 2H), 6.34 (d, *J* = 16.0 Hz, 1H), 5.79 (s, 1H), 5.64 (dd, *J* = 13.2, 4.5 Hz, 1H), 5.38 (t, *J* = 7.5 Hz, 1H), 3.31 (d, *J* = 14.5 Hz, 1H), 2.90 (d, *J* = 11.9 Hz, 1H), 2.79 (t, *J* = 10.5 Hz, 2H), 2.43 (d, *J* = 19.1 Hz, 1H), 2.23 (d, *J* = 11.6 Hz, 1H), 2.07 (t, *J* = 10.6 Hz, 2H), 1.99 (s, 3H), 1.54 (s, 3H), 1.52 (s, 3H), 1.45 (s, 3H), 1.37 (d, *J* = 4.4 Hz, 5H), 1.30 (s, 3H), 1.25 (s, 5H), 1.13 (s, 3H), 1.06 (s, 3H). ^13^C NMR (100 MHz, CDCl_3_) δ 212.1, 205.8, 200.9, 169.7, 166.5, 166.0, 152.9, 145.9, 145.4, 139.9, 134.5, 134.4, 130.6, 130.4, 129.0, 128.9, 128.3, 120.6, 119.2, 117.8, 117.4, 79.3, 77.9, 73.7, 73.5, 54.4, 51.4, 50.2, 48.8, 48.6, 48.1, 43.4, 42.3, 34.5, 32.2, 32.0, 29.5, 28.9, 26.7, 26.6, 23.9, 22.8, 21.9, 21.5, 20.1, 19.9, 18.8. HRMS (ESI) calcd. for C_50_H_58_NaO_10_ [M + Na]^+^ 841.3922, found 841.3928.

#### 3.1.6. Procedure for the Synthesis of Compounds **6a**–**6o** and **7a**–**7c**

Compounds **6a**–**6o** and **7a**–**7c** were synthesized according to the procedure previously reported [42,43,44,46].

#### 3.1.7. Procedure for the Synthesis of Compound **8**

*4-(((2S,9R,13R,14S,16R)-17-((R,E)-6-Acetoxy-2-hydroxy-6-methyl-3-oxohept-4-en-2-yl)-2-((tert-butyldimethylsilyl)oxy)-4,4,9,13,14-pentamethyl-3,11-dioxo-2,3,4,7,8,9,10,11,12,13,14,15,16,17-tetradecahydro-1H-cyclopenta[a]phenanthren-16-yl)oxy)-4-oxobutanoic acid* (**8**). To a solution of compound **2** (50.0 mg, 0.074 mmol) in dry DCM (2 mL) was added succinic anhydride (37.4 mg, 0.37 mmol) and DMAP (10.0 mg, 0.08 mmol). The mixture was stirred for 4 h at room temperature. The reaction was quenched with 1N HCl, the pH was adjusted to 2–3, and extraction was performed with CH_2_Cl_2_ (3 × 15 mL). The combined organic layers were washed with saturated brine, dried over Na_2_SO_4_, and concentrated to give an oily crude product, which was purified on a silica gel column to yield compound **8** (49.0 mg, yield: 86%) as a white solid. ^1^H NMR (400 MHz, DMSO-*d6*) δ 12.21 (s, 1H), 6.88 (d, *J* = 15.7 Hz, 1H), 6.73 (d, *J* = 15.8 Hz, 1H), 5.65 (s, 1H), 5.32 (s, 2H), 4.72 (d, *J* = 11.9 Hz, 1H), 3.47 (d, *J* = 14.2 Hz, 1H), 3.02 (d, *J* = 11.6 Hz, 1H), 2.60 (d, *J* = 6.2 Hz, 1H), 2.44 (s, 1H), 2.39–2.17 (m, 5H), 1.95 (s, 3H), 1.84 (dd, *J* = 26.6, 10.6 Hz, 4H), 1.46 (s, 6H), 1.26 (s, 3H), 1.20 (s, 7H), 1.12 (d, *J* = 15.3 Hz, 4H), 0.88 (s, 3H), 0.83 (s, 12H), 0.02 (s, 3H), −0.05 (s, 3H). ^13^C NMR (100 MHz, DMSO-*d6*) δ 212.3, 210.7, 203.5, 173.4, 171.3, 169.4, 150.3, 140.5, 120.4, 118.8, 79.3, 78.3, 73.5, 73.0, 55.1, 50.6, 49.8, 48.7, 47.7, 47.3, 42.7, 41.6, 35.9, 32.5, 29.0, 28.9, 28.7, 26.3, 26.2, 25.8, 24.7, 23.4, 21.7, 21.5, 19.8, 19.3, 18.1, 18.0, −4.5, −5.2. HRMS (ESI) calcd. for C_42_H_64_NaO_11_Si [M + Na]^+^ 795.4110, found 795.4115.

#### 3.1.8. General Procedure for the Synthesis of Compounds **9a**–**9c**

Compounds **7a**–**7c** (1.5 mmol) were dissolved in dry DCM. TFA (20% in DCM) was dropwise added into the reaction mixture at 0 °C, then the mixture was allowed to warm at 20 °C for 2 h. The solvent was removed by reduced pressure. The residue was purified by flash chromatography to obtain the crude amine as a colored oily matter.

Compound **8** (154 mg, 0.2 mmol) and HATU (95.1 mg, 0.25 mmol) were dissolved in dry DMF (4 mL), then DIPEA (44 μL, 0.25 mmol) was added. The mixture was stirred at 30 °C for 1 h, then the corresponding amine (0.6 mmol, 3 eq.) was added. The reaction was stirred for 2 h at this temperature. Then, the reaction was quenched with saturated aqueous NH_4_Cl and extracted with EA (3 × 15 mL). The combined organic layers were washed with saturated brine, dried over Na_2_SO_4_, and concentrated to give an oily crude product, which was purified on a silica gel column (PE/EA = 3:1–1:1) to give compound **7** as a yellow solid, which was simple purified to yield a white solid. The solid was used for the next step directly. The solid was dissolved in THF (2 mL). To the mixture was added HOAc (38 μL, 0.65 mmol) and TBAF (169 mg, 0.65 mmol). The mixture was stirred at room temperature for 24 h, and then diluted with ethyl acetate (20 mL). The organic phase was washed with H_2_O (3 × 20 mL), dried over Na_2_SO_4_, and concentrated. The residue was purified by column chromatography on silica gel to obtain a white solid **9a**–**9c**.

*3-((l1-oxidanyl)(oxo)(phenyl)-l5-sulfanyl)-4-(2-(4-(((9R,13R,14S,16R)-17-((R,E)-6-acetoxy-2-hydroxy-6-methyl-3-oxohept-4-en-2-yl)-2-Hydroxy-4,4,9,13,14-pentamethyl-3,11-dioxo-2,3,4,7,8,9,10,11,12,13,14,15,16,17-tetradecahydro-1H-cyclopenta[a]phenanthren-16-yl)oxy)-4-oxobutanamido)ethoxy)-1,2,5-oxadiazole 2-oxide* (**9a**) (67% yield in three steps). (purity: 99%). ^1^H NMR (400 MHz, CDCl_3_) δ 8.13 – 8.02 (m, 2H), 7.78 (t, *J* = 7.5 Hz, 1H), 7.65 (t, *J* = 7.8 Hz, 2H), 7.16 (d, *J* = 15.6 Hz, 1H), 6.57 (t, *J* = 5.7 Hz, 1H), 6.40 (d, *J* = 15.6 Hz, 1H), 5.76 (d, *J* = 5.4 Hz, 1H), 5.18 (t, *J* = 7.9 Hz, 1H), 4.52 (t, *J* = 5.8 Hz, 2H), 4.46–4.34 (m, 1H), 4.25 (s, 1H), 3.64–3.42 (m, 3H), 3.24 (d, *J* = 14.6 Hz, 1H), 2.71 (dd, *J* = 17.2, 7.2 Hz, 3H), 2.56–2.27 (m, 6H), 2.16–2.05 (m, 2H), 2.02 (s, 3H), 2.00–1.87 (m, 3H), 1.56 (d, *J* = 7.8 Hz, 6H), 1.40 (s, 3H), 1.33 (s, 3H), 1.26 (s, 3H), 1.24 (s, 3H), 1.07 (s, 3H), 1.00 (s, 3H). ^13^C NMR (100 MHz, CDCl_3_) δ 213.1, 211.8, 201.2, 172.5, 172.0, 170.1, 158.9, 153.1, 140.4, 137.8, 136.0, 129.9, 128.7, 120.5, 119.3, 110.6, 79.4, 77.8, 73.9, 71.7, 70.7, 54.2, 50.4, 50.1, 48.7, 48.5, 48.1, 43.1, 42.2, 37.1, 36.1, 33.8, 30.8, 29.4, 28.6, 26.9, 26.5, 23.8, 23.7, 22.1, 21.4, 20.2, 19.8, 18.9. HRMS (ESI) calcd. for C_46_H_59_N_3_NaO_15_S [M + Na]^+^ 948.3559, found 948.3562.

*4-(3-(4-(((9R,13R,14S,16R)-17-((R,E)-6-Acetoxy-2-hydroxy-6-methyl-3-oxohept-4-en-2-yl)-2-hydroxy-4,4,9,13,14-pentamethyl-3,11-dioxo-2,3,4,7,8,9,10,11,12,13,14,15,16,17-tetradecahydro-1H-cyclopenta[a]phenanthren-16-yl)oxy)-4-oxobutanamido)propoxy)-3-(phenylsulfonyl)-1,2,5-oxadiazole 2-oxide* (**9b**) (73% yield in three steps) (purity: 99%). ^1^H NMR (400 MHz, CDCl_3_) δ 8.11–8.01 (m, 2H), 7.77 (t, *J* = 7.5 Hz, 1H), 7.64 (t, *J* = 7.9 Hz, 2H), 7.18 (d, *J* = 15.6 Hz, 1H), 6.65 (t, *J* = 5.9 Hz, 1H), 6.40 (d, *J* = 15.6 Hz, 1H), 5.75 (d, *J* = 5.5 Hz, 1H), 5.17 (t, *J* = 7.9 Hz, 1H), 4.50 (t, *J* = 5.1 Hz, 2H), 4.45–4.35 (m, 1H), 4.25 (s, 1H), 3.79–3.66 (m, 2H), 3.61 (d, *J* = 3.8 Hz, 1H), 3.24 (d, *J* = 14.6 Hz, 1H), 2.70 (t, *J* = 11.3 Hz, 3H), 2.57–2.24 (m, 6H), 2.03 (s, 3H), 1.99–1.80 (m, 4H), 1.58 (s, 3H), 1.54 (s, 3H), 1.40 (s, 3H), 1.32 (s, 3H), 1.28 (s, 3H), 1.24 (d, *J* = 4.4 Hz, 6H), 1.07 (s, 3H), 1.00 (s, 3H). ^13^C NMR (100 MHz, CDCl_3_) δ 213.1, 211.8, 201.3, 172.3, 172.2, 170.3, 159.1, 153.3, 140.4, 137.9, 135.9, 129.9, 128.7, 120.5, 119.3, 110.7, 79.4, 77.8, 74.0, 71.7, 70.5, 54.0, 50.4, 50.1, 48.6, 48.5, 48.1, 43.1, 42.2, 38.4, 36.1, 33.8, 31.0, 29.8, 29.4, 29.4, 27.2, 26.4, 23.8, 23.6, 22.1, 21.4, 20.1, 19.8, 18.9. HRMS (ESI) calcd. for C_47_H_61_N_3_NaO_15_S [M + Na]^+^ 962.3716, found 962.3720.

*4-(2-(4-(4-(((9R,13R,14S,16R)-17-((R,E)-6-Acetoxy-2-hydroxy-6-methyl-3-oxohept-4-en-2-yl)-2-hydroxy-4,4,9,13,14-pentamethyl-3,11-dioxo-2,3,4,7,8,9,10,11,12,13,14,15,16,17-tetradecahydro-1H-cyclopenta[a]phenanthren-16-yl)oxy)-4-oxobutanoyl)piperazin-1-yl)ethoxy)-3-(phenylsulfonyl)-1,2,5-oxadiazole 2-oxide* (**9c**) (44% yield in three steps). (purity: 95%). ^1^H NMR (400 MHz, CDCl_3_) δ 8.04 (d, *J* = 7.6 Hz, 2H), 7.76 (t, *J* = 7.5 Hz, 1H), 7.62 (t, *J* = 7.9 Hz, 2H), 7.16 (d, *J* = 15.6 Hz, 1H), 6.41 (d, *J* = 15.6 Hz, 1H), 5.75 (d, *J* = 5.4 Hz, 1H), 5.26–5.12 (m, 1H), 4.55 (t, *J* = 5.3 Hz, 2H), 4.40 (dd, *J* = 12.8, 5.9 Hz, 1H), 4.29 (s, 1H), 3.68–3.52 (m, 3H), 3.47 (t, *J* = 8.3 Hz, 2H), 3.25 (d, *J* = 14.6 Hz, 1H), 2.88 (t, *J* = 5.3 Hz, 2H), 2.79 (s, 1H), 2.71 (dd, *J* = 11.0, 7.4 Hz, 3H), 2.63 (dt, *J* = 9.5, 6.1 Hz, 2H), 2.54 (dd, *J* = 9.3, 4.9 Hz, 2H), 2.50–2.25 (m, 5H), 2.01 (s, 3H), 1.98 (d, *J* = 9.7 Hz, 2H), 1.57 (d, *J* = 1.3 Hz, 6H), 1.41 (s, 3H), 1.31 (d, *J* = 7.5 Hz, 6H), 1.26 (s, 3H), 1.24 (s, 3H), 1.07 (s, 3H), 1.00 (s, 3H). ^13^C NMR (100 MHz, CDCl_3_) δ 213.1, 211.9, 201.2, 172.7, 169.8, 169.7, 159.0, 153.0, 140.4, 138.1, 135.8, 129.8, 128.6, 120.5, 119.4, 110.6, 79.2, 77.8, 73.9, 71.7, 69.4, 56.2, 54.3, 53.5, 53.2, 50.4, 50.1, 48.7, 48.5, 48.1, 45.3, 43.1, 42.2, 41.8, 38.7, 36.1, 33.8, 29.4, 28.9, 27.8, 26.7, 26.6, 23.7, 22.0, 21.4, 20.2, 19.8, 18.9. HRMS (ESI) calcd. for C_50_H_67_N_4_O_15_S [M + H]^+^ 995.4318, found 995.4322.

#### 3.1.9. General Procedure for the Synthesis of Compounds **10a**–**10o**

To a solution of **6a**–**6o** (0.5 mmol) in dry DCM (20 mL) was added succinic anhydride (75 mg, 0.75 mmol) and DMAP (61 mg, 0.5 mmol). The mixture was stirred for 4 h at room temperature. The reaction was quenched with 1N HCl, the pH was adjusted to 2–3 and extraction was performed with CH_2_Cl_2_ (3 × 15 mL). The combined organic layers were washed with saturated brine, dried over Na_2_SO_4_, and concentrated to give an oily crude product, which was purified on a silica gel column to obtain the corresponding acid in a yield of 86–96%.

To a solution of compound **2** (135 mg, 0.2 mmol), EDCI (96 mg, 0.5 mmol), DMAP (1.2 mg, 0.01 mmol), and the corresponding acid (0.4 mmol, 2 eq.) in CH_2_Cl_2_ (2 mL) was added Et_3_N (69.5 μL, 0.5 mmol) at 0 °C. The mixture was stirred for 8 h at room temperature. The reaction was quenched with saturated aqueous NaHCO_3_ and extracted with CH_2_Cl_2_ (3 × 15 mL). The combined organic layers were washed with saturated brine, dried over Na_2_SO_4_, and concentrated to give an oily crude product, which was simple purified to yield a white solid. The solid was used for the next step directly. The solid was dissolved in THF (2 mL). HOAc (38 μL, 0.65 mmol) and TBAF (169 mg, 0.65 mmol) were added to the mixture. The mixture was stirred at room temperature for 24 h, and then diluted with ethyl acetate (20 mL). The organic phase was washed with H_2_O (3 × 20 mL), dried over Na_2_SO_4_, and concentrated. The residue was purified by column chromatography on silica gel to obtain a white solid **10a**–**10o**.

*4-(2-((4-(((9R,13R,14S,16R)-17-((R,E)-6-Acetoxy-2-hydroxy-6-methyl-3-oxohept-4-en-2-yl)-2-hydroxy-4,4,9,13,14-pentamethyl-3,11-dioxo-2,3,4,7,8,9,10,11,12,13,14,15,16,17-tetradecahydro-1H-cyclopenta[a]phenanthren-16-yl)oxy)-4-oxobutanoyl)oxy)ethoxy)-3-(phenylsulfonyl)-1,2,5-oxadiazole 2-oxide* (**10a**) (81% yield in two steps). (purity: 95%). ^1^H NMR (400 MHz, CDCl_3_) δ 8.05 (d, *J* = 7.7 Hz, 2H), 7.77 (t, *J* = 7.5 Hz, 1H), 7.63 (t, *J* = 7.8 Hz, 2H), 7.18 (d, *J* = 15.6 Hz, 1H), 6.40 (d, *J* = 15.6 Hz, 1H), 5.75 (d, *J* = 5.2 Hz, 1H), 5.19 (s, 1H), 4.67–4.56 (m, 2H), 4.55–4.46 (m, 2H), 4.45–4.35 (m, 1H), 4.28 (s, 1H), 3.60 (d, *J* = 3.7 Hz, 1H), 3.25 (d, *J* = 14.6 Hz, 1H), 2.78–2.66 (m, 3H), 2.65–2.25 (m, 6H), 2.05–1.85 (m, 6H), 1.58 (s, 3H), 1.55 (s, 3H), 1.40 (s, 3H), 1.32 (s, 3H), 1.29 (s, 3H), 1.25 (s, 3H), 1.24 (s, 2H), 1.07 (s, 3H), 1.00 (s, 3H). ^13^C NMR (100 MHz, CDCl_3_) δ 213.1, 211.8, 201.1, 172.2, 171.7, 169.8, 158.8, 153.2, 140.5, 138.0, 135.9, 129.9, 128.7, 120.5, 119.2, 110.5, 79.2, 77.8, 74.1, 71.7, 68.9, 61.5, 54.2, 50.3, 50.1, 48.6, 48.5, 48.1, 43.1, 42.2, 36.1, 33.8, 29.4, 28.8, 28.6, 26.9, 26.3, 23.8, 23.7, 22.0, 21.4, 20.1, 19.8, 18.9. HRMS (ESI) calcd. for C_46_H_58_N_2_NaO_16_S [M + Na]^+^ 949.3399, found, 949.3402.

*4-(3-((4-(((9R,13R,14S,16R)-17-((R,E)-6-Acetoxy-2-hydroxy-6-methyl-3-oxohept-4-en-2-yl)-2-hydroxy-4,4,9,13,14-pentamethyl-3,11-dioxo-2,3,4,7,8,9,10,11,12,13,14,15,16,17-tetradecahydro-1H-cyclopenta[a]phenanthren-16-yl)oxy)-4-oxobutanoyl)oxy)propoxy)-3-(phenylsulfonyl)-1,2,5-oxadiazole 2-oxide* (**10b**) (74% yield in two steps). (purity: 99%). ^1^H NMR (400 MHz, CDCl_3_) δ 8.05 (d, *J* = 8.0 Hz, 2H), 7.76 (t, *J* = 7.4 Hz, 1H), 7.63 (t, *J* = 7.7 Hz, 2H), 7.17 (d, *J* = 15.6 Hz, 1H), 6.39 (d, *J* = 15.6 Hz, 1H), 5.76 (d, *J* = 5.0 Hz, 1H), 5.18 (t, *J* = 8.0 Hz, 1H), 4.51 (t, *J* = 6.0 Hz, 2H), 4.45 – 4.36 (m, 1H), 4.36 – 4.21 (m, 3H), 3.60 (d, *J* = 3.7 Hz, 1H), 3.24 (d, *J* = 14.6 Hz, 1H), 2.71 (dd, *J* = 15.6, 11.0 Hz, 3H), 2.65–2.34 (m, 5H), 2.30 (dd, *J* = 9.0, 5.7 Hz, 1H), 2.21 (p, *J* = 5.9 Hz, 2H), 2.01 (s, 3H), 2.00–1.86 (m, 3H), 1.58 (s, 3H), 1.55 (s, 3H), 1.45–1.33 (m, 4H), 1.32 (s, 3H), 1.28 (s, 3H), 1.26 (s, 3H), 1.24 (s, 1H), 1.07 (s, 3H), 1.00 (s, 3H). ^13^C NMR (100 MHz, CDCl_3_) δ 213.1, 211.7, 201.0, 172.3, 171.8, 169.8, 159.0, 153.1, 140.5, 138.1, 135.8, 129.8, 128.7, 120.5, 119.3, 110.6, 79.2, 77.8, 74.1, 71.7, 68.1, 60.6, 54.2, 50.3, 50.0, 48.6, 48.5, 48.1, 43.1, 42.2, 36.1, 33.8, 29.4, 28.9, 28.7, 28.1, 26.9, 26.4, 23.8, 23.7, 22.0, 21.4, 20.2, 19.8, 18.9. HRMS (ESI) calcd. for C_47_H_60_N_2_NaO_16_S [M + Na]^+^ 963.3556, found 963.3560.

*4-(4-((4-(((9R,13R,14S,16R)-17-((R,E)-6-Acetoxy-2-hydroxy-6-methyl-3-oxohept-4-en-2-yl)-2-hydroxy-4,4,9,13,14-pentamethyl-3,11-dioxo-2,3,4,7,8,9,10,11,12,13,14,15,16,17-tetradecahydro-1H-cyclopenta[a]phenanthren-16-yl)oxy)-4-oxobutanoyl)oxy)butoxy)-3-(phenylsulfonyl)-1,2,5-oxadiazole 2-oxide* (**10c**) (78% yield in two steps). (purity: 99%). ^1^H NMR (400 MHz, CDCl_3_) δ 8.09–8.00 (m, 2H), 7.76 (t, *J* = 7.5 Hz, 1H), 7.63 (t, *J* = 7.9 Hz, 2H), 7.17 (d, *J* = 15.6 Hz, 1H), 6.40 (d, *J* = 15.6 Hz, 1H), 5.76 (d, *J* = 5.5 Hz, 1H), 5.19 (t, *J* = 7.9 Hz, 1H), 4.45 (t, *J* = 6.3 Hz, 2H), 4.42–4.36 (m, 1H), 4.27 (s, 1H), 4.23–4.11 (m, 2H), 3.60 (d, *J* = 3.9 Hz, 1H), 3.25 (d, *J* = 14.6 Hz, 1H), 2.78–2.65 (m, 3H), 2.65–2.24 (m, 6H), 2.01 (s, 3H), 2.00–1.93 (m, 4H), 1.82 (dt, *J* = 13.0, 6.4 Hz, 2H), 1.67 (s, 1H), 1.58 (s, 3H), 1.55 (s, 3H), 1.41 (s, 3H), 1.37 (d, *J* = 14.5 Hz, 1H), 1.32 (s, 3H), 1.29 (s, 3H), 1.26 (s, 3H), 1.22 (d, *J* = 13.6 Hz, 1H), 1.07 (s, 3H), 1.00 (s, 3H). ^13^C NMR (100 MHz, CDCl_3_) δ 213.0, 211.7, 201.0, 172.4, 171.8, 169.8, 159.0, 153.1, 140.5, 138.1, 135.8, 129.8, 128.7, 120.5, 119.3, 110.6, 79.2, 77.8, 74.1, 71.7, 71.0, 64.0, 54.2, 50.3, 50.1, 48.6, 48.5, 48.1, 43.1, 42.2, 36.1, 33.8, 29.4, 28.9, 28.7, 26.9, 26.4, 25.3, 25.1, 23.9, 23.7, 22.0, 21.4, 20.2, 19.8, 18.9. HRMS (ESI) calcd. for C_48_H_62_N_2_NaO_16_S [M + Na]^+^ 977.3712, found 977.3718.

*4-((5-((4-(((9R,13R,14S,16R)-17-((R,E)-6-Acetoxy-2-hydroxy-6-methyl-3-oxohept-4-en-2-yl)-2-hydroxy-4,4,9,13,14-pentamethyl-3,11-dioxo-2,3,4,7,8,9,10,11,12,13,14,15,16,17-tetradecahydro-1H-cyclopenta[a]phenanthren-16-yl)oxy)-4-oxobutanoyl)oxy)pentyl)oxy)-3-(phenylsulfonyl)-1,2,5-oxadiazole 2-oxide* (**10d**) (82% yield in two steps). (purity: 98%). ^1^H NMR (400 MHz, CDCl_3_) δ 8.04 (d, *J* = 7.9 Hz, 2H), 7.76 (t, *J* = 7.4 Hz, 1H), 7.62 (t, *J* = 7.7 Hz, 2H), 7.17 (d, *J* = 15.6 Hz, 1H), 6.40 (d, *J* = 15.6 Hz, 1H), 5.76 (d, *J* = 4.7 Hz, 1H), 5.19 (s, 1H), 4.42 (t, *J* = 6.3 Hz, 3H), 4.26 (s, 1H), 4.12 (dd, *J* = 9.8, 6.2 Hz, 2H), 3.59 (d, *J* = 3.6 Hz, 1H), 3.24 (d, *J* = 14.6 Hz, 1H), 2.80–2.66 (m, 3H), 2.64–2.25 (m, 6H), 2.09–1.85 (m, 8H), 1.76–1.61 (m, 3H), 1.58 (s, 3H), 1.55 (d, *J* = 7.0 Hz, 4H), 1.40 (d, *J* = 6.5 Hz, 3H), 1.32 (s, 3H), 1.29 (s, 3H), 1.25 (d, *J* = 7.4 Hz, 5H), 1.07 (s, H), 1.00 (s, 3H). ^13^C NMR (100 MHz, CDCl_3_) δ 213.0, 211.7, 201.0, 172.5, 171.8, 169.7, 159.1, 153.0, 140.5, 138.2, 135.8, 129.8, 128.7, 120.5, 119.3, 110.6, 79.2, 77.8, 74.1, 71.7, 71.4, 64.4, 54.3, 50.3, 50.1, 48.7, 48.5, 48.1, 43.2, 42.2, 36.1, 33.8, 29.5, 29.0, 28.8, 28.2, 28.2, 26.8, 26.4, 23.9, 23.7, 22.3, 22.0, 21.4, 20.2, 19.8, 18.9. HRMS (MALDI) calcd. for C_49_H_64_N_2_NaO_16_S [M + Na]^+^ 991.3869, found 991.3875.

*4-((6-((4-(((9R,13R,14S,16R)-17-((R,E)-6-Acetoxy-2-hydroxy-6-methyl-3-oxohept-4-en-2-yl)-2-hydroxy-4,4,9,13,14-pentamethyl-3,11-dioxo-2,3,4,7,8,9,10,11,12,13,14,15,16,17-tetradecahydro-1H-cyclopenta[a]phenanthren-16-yl)oxy)-4-oxobutanoyl)oxy)hexyl)oxy)-3-(phenylsulfonyl)-1,2,5-oxadiazole 2-oxide* (**10e**) (72% yield in two steps). (purity: 96%). ^1^H NMR (400 MHz, CDCl_3_) δ 8.05 (d, *J* = 7.7 Hz, 2H), 7.76 (t, *J* = 7.4 Hz, 1H), 7.62 (t, *J* = 7.8 Hz, 2H), 7.17 (d, *J* = 15.6 Hz, 1H), 6.40 (d, *J* = 15.6 Hz, 1H), 5.75 (s, 1H), 5.19 (t, *J* = 7.9 Hz, 1H), 4.42 (t, *J* = 6.4 Hz, 3H), 4.28 (s, 1H), 4.08 (dt, *J* = 10.7, 6.3 Hz, 2H), 3.60 (d, *J* = 3.7 Hz, 1H), 3.25 (d, *J* = 14.5 Hz, 1H), 2.71 (dd, *J* = 15.1, 11.0 Hz, 3H), 2.64 – 2.26 (m, 6H), 2.06–1.93 (m, 6H), 1.91–1.85 (m, 2H), 1.71–1.62 (m, 2H), 1.58 (s, 3H), 1.56 (s, 3H), 1.50–1.39 (m, 7H), 1.33 (s, 3H), 1.29 (s, 3H), 1.26 (s, 3H), 1.24 (s, 2H), 1.07 (s, 3H), 1.00 (s, 3H). ^13^C NMR (100 MHz, CDCl_3_) δ 213.0, 211.7, 201.0, 172.5, 171.8, 169.7, 159.2, 153.1, 140.5, 138.2, 135.8, 129.8, 128.7, 120.5, 119.3, 110.6, 79.2, 77.8, 74.0, 71.7, 71.5, 64.6, 54.3, 50.4, 50.1, 48.7, 48.5, 48.1, 43.2, 42.2, 36.1, 33.8, 29.5, 29.0, 28.8, 28.6, 28.4, 26.8, 26.4, 25.6, 25.4, 23.9, 23.7, 22.0, 21.4, 20.2, 19.8, 18.9. HRMS (ESI) calcd. for C_50_H_66_N_2_NaO_16_S [M + Na]^+^ 1005.4025, found 1005.4028.

*4-((7-((4-(((9R,13R,14S,16R)-17-((R,E)-6-Acetoxy-2-hydroxy-6-methyl-3-oxohept-4-en-2-yl)-2-hydroxy-4,4,9,13,14-pentamethyl-3,11-dioxo-2,3,4,7,8,9,10,11,12,13,14,15,16,17-tetradecahydro-1H-cyclopenta[a]phenanthren-16-yl)oxy)-4-oxobutanoyl)oxy)heptyl)oxy)-3-(phenylsulfonyl)-1,2,5-oxadiazole 2-oxide* (**10f**) (76% yield in two steps). (purity: 95%). ^1^H NMR (400 MHz, CDCl_3_) δ 8.04 (d, *J* = 7.9 Hz, 2H), 7.76 (t, *J* = 7.5 Hz, 1H), 7.62 (t, *J* = 7.8 Hz, 2H), 7.16 (d, *J* = 15.6 Hz, 1H), 6.40 (d, *J* = 15.6 Hz, 1H), 5.75 (d, *J* = 5.3 Hz, 1H), 5.19 (t, *J* = 7.9 Hz, 1H), 4.40 (t, *J* = 6.5 Hz, 3H), 4.27 (s, 1H), 4.07 (td, *J* = 6.6, 2.0 Hz, 2H), 3.59 (d, *J* = 3.8 Hz, 1H), 3.24 (d, *J* = 14.6 Hz, 1H), 2.71 (dd, *J* = 16.0, 7.8 Hz, 3H), 2.63–2.24 (m, 6H), 2.04–1.82 (m, 8H), 1.67–1.60 (m, 2H), 1.58 (s, 3H), 1.55 (s, 3H), 1.44 (dd, *J* = 8.3, 5.8 Hz, 2H), 1.40 (s, 3H), 1.35 (s, 4H), 1.32 (s, 3H), 1.28 (s, 3H), 1.26 (s, 3H), 1.24 (s, 2H), 1.07 (s, 3H), 1.00 (s, 3H). ^13^C NMR (100 MHz, CDCl_3_) δ 213.0, 211.7, 201.0, 172.5, 171.8, 169.7, 159.2, 153.1, 140.5, 138.2, 135.8, 129.8, 128.6, 120.5, 119.3, 110.6, 79.2, 77.8, 74.0, 71.7, 71.6, 64.8, 54.2, 50.3, 50.1, 48.6, 48.5, 48.1, 43.1, 42.2, 36.1, 33.8, 29.4, 29.0, 28.8, 28.8, 28.6, 28.4, 26.8, 26.4, 25.9, 25.6, 23.8, 23.7, 22.0, 21.4, 20.2, 19.8, 18.9. HRMS (MALDI) calcd. for C_51_H_68_N_2_NaO_16_S [M + Na]^+^ 1019.4182, found 1019.4185.

*4-((8-((4-(((9R,13R,14S,16R)-17-((R,E)-6-Acetoxy-2-hydroxy-6-methyl-3-oxohept-4-en-2-yl)-2-hydroxy-4,4,9,13,14-pentamethyl-3,11-dioxo-2,3,4,7,8,9,10,11,12,13,14,15,16,17-tetradecahydro-1H-cyclopenta[a]phenanthren-16-yl)oxy)-4-oxobutanoyl)oxy)octyl)oxy)-3-(phenylsulfonyl)-1,2,5-oxadiazole 2-oxide* (**10g**) (66% yield in two steps). (purity: 99%). ^1^H NMR (400 MHz, CDCl_3_) δ 8.04 (dd, *J* = 8.5, 1.2 Hz, 2H), 7.79–7.72 (m, 1H), 7.61 (dd, *J* = 10.8, 5.0 Hz, 2H), 7.16 (d, *J* = 15.6 Hz, 1H), 6.40 (d, *J* = 15.6 Hz, 1H), 5.75 (d, *J* = 5.6 Hz, 1H), 5.19 (t, *J* = 7.8 Hz, 1H), 4.40 (t, *J* = 6.5 Hz, 3H), 4.26 (s, 1H), 4.06 (td, *J* = 6.7, 1.7 Hz, 2H), 3.58 (d, *J* = 3.7 Hz, 1H), 3.24 (d, *J* = 14.7 Hz, 1H), 2.71 (dd, *J* = 15.6, 7.5 Hz, 3H), 2.60–2.34 (m, 5H), 2.30 (ddd, *J* = 9.1, 6.7, 3.4 Hz, 1H), 2.04–1.92 (m, 6H), 1.90–1.81 (m, 2H), 1.66–1.60 (m, 2H), 1.57 (d, *J* = 8.1 Hz, 6H), 1.45 (dd, *J* = 9.4, 5.9 Hz, 2H), 1.40 (s, 3H), 1.39 (s, 1H), 1.35 (d, *J* = 1.4 Hz, 6H), 1.32 (s, 3H), 1.28 (s, 3H), 1.26 (s, 3H), 1.24 (s, 1H), 1.07 (s, 3H), 1.00 (s, 3H). ^13^C NMR (100 MHz, CDCl_3_) δ 212.9, 211.6, 200.9, 172.3, 171.7, 169.6, 159.1, 152.9, 140.4, 138.1, 135.6, 129.6, 128.5, 120.3, 119.2, 110.5, 79.1, 77.7, 73.9, 71.6, 71.6, 64.8, 54.2, 50.2, 50.0, 48.5, 48.4, 48.0, 43.1, 42.1, 36.0, 33.7, 29.3, 29.1, 29.0, 28.9, 28.7, 28.6, 28.4, 26.7, 26.3, 25.8, 25.5, 23.8, 23.6, 21.8, 21.3, 20.0, 19.7, 18.8. HRMS (MALDI) calcd. for C_52_H_70_N_2_NaO_16_S [M + Na]^+^ 1033.4338, found 1033.4342.

*4-((9-((4-(((9R,13R,14S,16R)-17-((R,E)-6-Acetoxy-2-hydroxy-6-methyl-3-oxohept-4-en-2-yl)-2-hydroxy-4,4,9,13,14-pentamethyl-3,11-dioxo-2,3,4,7,8,9,10,11,12,13,14,15,16,17-tetradecahydro-1H-cyclopenta[a]phenanthren-16-yl)oxy)-4-oxobutanoyl)oxy)nonyl)oxy)-3-(phenylsulfonyl)-1,2,5-oxadiazole 2-oxide* (**10h**) (69% yield in two steps). (purity: 97%). ^1^H NMR (400 MHz, CDCl_3_) δ 8.05 (dd, *J* = 8.4, 1.1 Hz, 2H), 7.76 (dd, *J* = 10.7, 4.3 Hz, 1H), 7.62 (t, *J* = 7.9 Hz, 2H), 7.16 (d, *J* = 15.6 Hz, 1H), 6.41 (d, *J* = 15.6 Hz, 1H), 5.76 (d, *J* = 5.6 Hz, 1H), 5.20 (t, *J* = 7.8 Hz, 1H), 4.41 (t, *J* = 6.6 Hz, 3H), 4.27 (s, 1H), 4.08 (dt, *J* = 6.9, 6.5 Hz, 2H), 3.70 (s, 1H), 3.59 (d, *J* = 3.9 Hz, 1H), 3.25 (d, *J* = 14.7 Hz, 1H), 2.72 (dd, *J* = 15.2, 11.0 Hz, 3H), 2.61–2.35 (m, 5H), 2.35–2.27 (m, 1H), 2.04–1.93 (m, 6H), 1.92–1.81 (m, 2H), 1.61 (d, *J* = 7.5 Hz, 3H), 1.59 (s, 3H), 1.56 (s, 3H), 1.44 (d, *J* = 7.4 Hz, 2H), 1.41 (s, 3H), 1.33 (s, 8H), 1.29 (s, 3H), 1.27 (s, 3H), 1.25 (s, 3H), 1.08 (s, 3H), 1.01 (s, 3H). ^13^C NMR (100 MHz, CDCl_3_) δ 213.1, 211.7, 201.0, 172.5, 171.8, 169.8, 159.1, 153.1, 140.5, 138.2, 135.7, 129.8, 128.7, 120.5, 119.3, 110.6, 100.1, 79.2, 77.8, 74.0, 71.7, 65.0, 54.3, 50.4, 50.1, 48.7, 48.5, 48.1, 43.2, 42.2, 36.1, 33.8, 29.8, 29.5, 29.3, 29.2, 29.0, 28.8, 28.7, 28.5, 26.8, 26.4, 26.0, 25.7, 23.9, 23.7, 22.0, 21.4, 20.2, 19.8, 18.9. HRMS (MALDI) calcd. for C_53_H_72_N_2_NaO_16_S [M + Na]^+^ 1047.4495, found 1047.4498.

*4-((10-((4-(((9R,13R,14S,16R)-17-((R,E)-6-Acetoxy-2-hydroxy-6-methyl-3-oxohept-4-en-2-yl)-2-hydroxy-4,4,9,13,14-pentamethyl-3,11-dioxo-2,3,4,7,8,9,10,11,12,13,14,15,16,17-tetradecahydro-1H-cyclopenta[a]phenanthren-16-yl)oxy)-4-oxobutanoyl)oxy)decyl)oxy)-3-(phenylsulfonyl)-1,2,5-oxadiazole 2-oxide* (**10i**) (71% yield in two steps). (purity: 95%). ^1^H NMR (400 MHz, CDCl_3_) δ 8.08–7.98 (m, 2H), 7.74 (t, *J* = 7.5 Hz, 1H), 7.60 (t, *J* = 7.9 Hz, 2H), 7.14 (d, *J* = 15.6 Hz, 1H), 6.40 (d, *J* = 15.6 Hz, 1H), 5.74 (d, *J* = 5.5 Hz, 1H), 5.18 (t, *J* = 7.9 Hz, 1H), 4.44–4.35 (m, 3H), 4.27 (d, *J* = 7.8 Hz, 1H), 4.10–3.99 (m, 2H), 3.58 (d, *J* = 3.9 Hz, 1H), 3.24 (d, *J* = 14.6 Hz, 1H), 2.76–2.66 (m, 3H), 2.60–2.23 (m, 6H), 2.04–1.90 (m, 6H), 1.90–1.79 (m, 2H), 1.65–1.58 (m, 2H), 1.56 (s, 3H), 1.55 (s, 3H), 1.38–1.44 (m, 6H), 1.31 (s, 13H), 1.27 (s, 3H), 1.25 (s, 3H), 1.23 (s, 1H), 1.05 (s, 3H), 0.99 (s, 3H). ^13^C NMR (100 MHz, CDCl_3_) δ 213.0, 211.7, 201.0, 172.4, 171.7, 169.7, 159.1, 153.0, 140.5, 138.2, 135.7, 129.7, 128.6, 120.4, 119.3, 110.6, 79.2, 77.8, 74.0, 71.7, 71.7, 64.9, 54.2, 50.3, 50.0, 48.6, 48.5, 48.1, 43.1, 42.2, 36.0, 33.8, 29.5, 29.4, 29.3, 29.1, 29.0, 28.8, 28.7, 28.5, 26.7, 26.4, 25.9, 25.6, 23.8, 23.7, 21.9, 21.3, 20.1, 19.8, 18.8. HRMS (MALDI) calcd. for C_54_H_74_N_2_NaO_16_S [M + Na]^+^ 1061.4651, found 1061.4655.

*4-((11-((4-(((9R,13R,14S,16R)-17-((R,E)-6-Acetoxy-2-hydroxy-6-methyl-3-oxohept-4-en-2-yl)-2-hydroxy-4,4,9,13,14-pentamethyl-3,11-dioxo-2,3,4,7,8,9,10,11,12,13,14,15,16,17-tetradecahydro-1H-cyclopenta[a]phenanthren-16-yl)oxy)-4-oxobutanoyl)oxy)undecyl)oxy)-3-(phenylsulfonyl)-1,2,5-oxadiazole 2-oxide* (**10j**) (75% yield in two steps). (purity: 95%). ^1^H NMR (400 MHz, CDCl_3_) δ 8.05 (d, *J* = 8.1 Hz, 2H), 7.75 (t, *J* = 7.3 Hz, 1H), 7.61 (t, *J* = 7.5 Hz, 2H), 7.16 (d, *J* = 15.5 Hz, 1H), 6.40 (d, *J* = 15.6 Hz, 1H), 5.76 (d, *J* = 4.0 Hz, 1H), 5.20 (t, *J* = 8.0 Hz, 1H), 4.41 (t, *J* = 6.5 Hz, 3H), 4.27 (s, 1H), 4.05 (t, *J* = 6.7 Hz, 2H), 3.59 (s, 1H), 3.24 (d, *J* = 14.6 Hz, 1H), 2.71 (dd, *J* = 14.1, 11.1 Hz, 3H), 2.65–2.26 (m, 6H), 2.05–1.92 (m, 6H), 1.92–1.79 (m, 3H), 1.61 (d, *J* = 6.1 Hz, 1H), 1.58 (s, 3H), 1.56 (s, 3H), 1.48–1.38 (m, 6H), 1.28 (dd, *J* = 21.2, 12.1 Hz, 22H), 1.07 (s, 3H), 1.00 (s, 3H). ^13^C NMR (100 MHz, CDCl_3_) δ 213.0, 211.7, 201.0, 172.5, 171.8, 169.7, 159.2, 153.0, 140.6, 138.3, 135.7, 129.8, 128.7, 120.5, 119.3, 110.6, 79.2, 77.8, 74.0, 71.8, 71.7, 65.0, 54.3, 50.4, 50.1, 48.7, 48.5, 48.1, 43.2, 42.2, 36.1, 33.8, 29.8, 29.6, 29.6, 29.5, 29.4, 29.2, 29.0, 28.9, 28.7, 28.5, 26.8, 26.4, 26.0, 25.7, 23.9, 23.7, 22.0, 21.4, 20.2, 19.8, 18.9. HRMS (MALDI) calcd. for C_55_H_76_N_2_NaO_16_S [M + Na]^+^ 1075.4808, found 1075.4812.

*4-((12-((4-(((9R,13R,14S,16R)-17-((R,E)-6-Acetoxy-2-hydroxy-6-methyl-3-oxohept-4-en-2-yl)-2-hydroxy-4,4,9,13,14-pentamethyl-3,11-dioxo-2,3,4,7,8,9,10,11,12,13,14,15,16,17-tetradecahydro-1H-cyclopenta[a]phenanthren-16-yl)oxy)-4-oxobutanoyl)oxy)dodecyl)oxy)-3-(phenylsulfonyl)-1,2,5-oxadiazole 2-oxide* (**10k**) (73% yield in two steps). (purity: 96%). ^1^H NMR (400 MHz, CDCl_3_) δ 8.04 (d, *J* = 7.5 Hz, 2H), 7.75 (t, *J* = 7.5 Hz, 1H), 7.61 (t, *J* = 7.8 Hz, 2H), 7.15 (d, *J* = 15.6 Hz, 1H), 6.41 (d, *J* = 15.6 Hz, 1H), 5.76 (d, *J* = 5.3 Hz, 1H), 5.20 (t, *J* = 7.9 Hz, 1H), 4.40 (t, *J* = 6.6 Hz, 3H), 4.26 (s, 1H), 4.05 (t, *J* = 6.8 Hz, 2H), 3.58 (d, *J* = 3.6 Hz, 1H), 3.24 (d, *J* = 14.7 Hz, 1H), 2.79–2.65 (m, 3H), 2.60–2.34 (m, 5H), 2.30 (ddd, *J* = 12.3, 6.1, 3.2 Hz, 1H), 2.05–1.92 (m, 6H), 1.91–1.81 (m, 2H), 1.61 (d, *J* = 6.2 Hz, 2H), 1.57 (d, *J* = 7.9 Hz, 6H), 1.46–1.37 (m, 6H), 1.32 (s, 6H), 1.28 (s, 12H), 1.26 (s, 3H), 1.24 (s, 3H), 1.07 (s, 3H), 1.00 (s, 3H). ^13^C NMR (100 MHz, CDCl_3_) δ 213.0, 211.7, 201.1, 172.4, 171.8, 169.7, 159.2, 153.0, 140.6, 138.3, 135.7, 129.7, 128.7, 120.5, 119.4, 110.6, 79.2, 77.8, 74.0, 71.8, 71.7, 65.0, 54.3, 50.3, 50.1, 48.7, 48.5, 48.1, 43.2, 42.2, 36.1, 33.9, 29.8, 29.7, 29.6, 29.6, 29.5, 29.4, 29.2, 29.0, 28.9, 28.7, 28.5, 26.8, 26.4, 26.0, 25.7, 23.9, 23.8, 22.0, 21.4, 20.2, 19.8, 18.9. HRMS (MALDI) calcd. for C_56_H_78_N_2_NaO_16_S [M + Na]^+^ 1089.4964, found 1089.4968.

*4-(((E)-4-((4-(((9R,13R,14S,16R)-17-((R,E)-6-Acetoxy-2-hydroxy-6-methyl-3-oxohept-4-en-2-yl)-2-hydroxy-4,4,9,13,14-pentamethyl-3,11-dioxo-2,3,4,7,8,9,10,11,12,13,14,15,16,17-tetradecahydro-1H-cyclopenta[a]phenanthren-16-yl)oxy)-4-oxobutanoyl)oxy)but-2-en-1-yl)oxy)-3-(phenylsulfonyl)-1,2,5-oxadiazole 2-oxide* (**10l**) (61% yield in two steps). (purity: 99%). ^1^H NMR (400 MHz, CDCl_3_) δ 8.06 (d, *J* = 7.5 Hz, 2H), 7.77 (t, *J* = 7.5 Hz, 1H), 7.63 (t, *J* = 7.8 Hz, 2H), 7.18 (d, *J* = 15.6 Hz, 1H), 6.39 (d, *J* = 15.6 Hz, 1H), 5.91 (t, *J* = 5.5 Hz, 2H), 5.76 (d, *J* = 5.4 Hz, 1H), 5.20 (t, *J* = 8.0 Hz, 1H), 5.05 (d, *J* = 5.0 Hz, 2H), 4.74 (d, *J* = 4.9 Hz, 2H), 4.46 – 4.36 (m, 1H), 4.28 (s, 1H), 3.60 (d, *J* = 3.8 Hz, 1H), 3.25 (d, *J* = 14.7 Hz, 1H), 2.72 (dd, *J* = 19.1, 10.9 Hz, 3H), 2.66–2.27 (m, 7H), 2.04–1.97 (m, 4H), 1.58 (s, 3H), 1.55 (s, 3H), 1.41 (s, 3H), 1.33 (s, 3H), 1.29 (s, 3H), 1.26 (s, 3H), 1.25 (s, 3H), 1.08 (s, 3H), 1.01 (s, 3H). ^13^C NMR (100 MHz, CDCl_3_) δ 213.1, 211.7, 201.0, 172.1, 171.8, 169.8, 153.2, 140.5, 135.9, 130.4, 129.9, 128.7, 125.9, 120.5, 119.2, 79.2, 77.8, 74.1, 71.7, 66.7, 60.3, 54.2, 50.4, 50.1, 48.6, 48.5, 48.2, 43.2, 42.2, 36.1, 33.8, 29.8, 29.5, 28.9, 28.7, 26.9, 26.4, 23.9, 23.7, 22.0, 21.4, 20.2, 19.8, 18.9. HRMS (ESI) calcd. for C_48_H_60_N_2_NaO_16_S [M+Na]^+^ 975.3556, found 975.3560.

*4-((4-((4-(((9R,13R,14S,16R)-17-((R,E)-6-Acetoxy-2-hydroxy-6-methyl-3-oxohept-4-en-2-yl)-2-hydroxy-4,4,9,13,14-pentamethyl-3,11-dioxo-2,3,4,7,8,9,10,11,12,13,14,15,16,17-tetradecahydro-1H-cyclopenta[a]phenanthren-16-yl)oxy)-4-oxobutanoyl)oxy)but-2-yn-1-yl)oxy)-3-(phenylsulfonyl)-1,2,5-oxadiazole 2-oxide* (**10m**) (77% yield in two steps). (purity: 99%). ^1^H NMR (400 MHz, CDCl_3_) δ 8.07 (d, *J* = 7.5 Hz, 2H), 7.77 (t, *J* = 7.3 Hz, 1H), 7.64 (t, *J* = 7.6 Hz, 2H), 7.18 (d, *J* = 14.9 Hz, 1H), 6.40 (d, *J* = 15.4 Hz, 1H), 5.77 (s, 1H), 5.21 (t, *J* = 7.5 Hz, 1H), 5.10 (s, 2H), 4.79–4.67 (m, 2H), 4.41 (d, *J* = 12.7 Hz, 1H), 4.27 (s, 1H), 3.59 (s, 1H), 3.25 (d, *J* = 14.8 Hz, 1H), 2.74–2.67 (m, 3H), 2.65–2.54 (m, 2H), 2.43 (d, *J* = 4.5 Hz, 2H), 2.40–2.27 (m, 2H), 2.01 (d, *J* = 9.7 Hz, 4H), 1.59 (s, 3H), 1.56 (s, 3H), 1.41 (s, 3H), 1.33 (s, 3H), 1.29 (s, 3H), 1.27 (s, 3H), 1.25 (s, 4H), 1.08 (s, 3H), 1.01 (s, 3H). ^13^C NMR (100 MHz, CDCl_3_) δ 213.0, 211.7, 201.1, 171.6, 169.8, 158.0, 153.2, 140.6, 137.8, 135.9, 129.9, 128.8, 120.5, 119.3, 100.0, 84.0, 79.2, 78.8, 77.8, 74.2, 71.8, 58.7, 54.3, 52.2, 50.4, 50.1, 48.7, 48.5, 48.2, 43.2, 42.2, 36.1, 33.9, 29.8, 29.5, 28.8, 28.7, 27.0, 26.4, 23.9, 23.8, 22.0, 21.4, 20.2, 19.8, 18.9. HRMS (ESI) calcd. for C_48_H_58_N_2_NaO_16_S [M + Na]^+^ 973.3399, found 973.3402.

*4-(3-((4-(((9R,13R,14S,16R)-17-((R,E)-6-Acetoxy-2-hydroxy-6-methyl-3-oxohept-4-en-2-yl)-2-hydroxy-4,4,9,13,14-pentamethyl-3,11-dioxo-2,3,4,7,8,9,10,11,12,13,14,15,16,17-tetradecahydro-1H-cyclopenta[a]phenanthren-16-yl)oxy)-4-oxobutanoyl)oxy)-2,2-dimethylpropoxy)-3-(phenylsulfonyl)-1,2,5-oxadiazole 2-oxide* (**10n**) (71% yield in two steps). (purity: 95%). ^1^H NMR (400 MHz, CDCl_3_) δ 8.04 (d, *J* = 7.9 Hz, 2H), 7.75 (t, *J* = 7.4 Hz, 1H), 7.62 (t, *J* = 7.7 Hz, 2H), 7.17 (d, *J* = 15.6 Hz, 1H), 6.39 (d, *J* = 15.6 Hz, 1H), 5.76 (d, *J* = 4.3 Hz, 1H), 5.15 (t, *J* = 8.0 Hz, 1H), 4.45–4.35 (m, 1H), 4.26 (s, 1H), 4.22–4.14 (m, 2H), 4.09 (d, *J* = 10.9 Hz, 1H), 3.98 (d, *J* = 11.0 Hz, 1H), 3.60 (d, *J* = 3.5 Hz, 1H), 3.24 (d, *J* = 14.6 Hz, 1H), 2.76–2.26 (m, 9H), 2.04–1.85 (m, 6H), 1.56 (d, *J* = 11.9 Hz, 6H), 1.40 (s, 3H), 1.32 (s, 3H), 1.28 (s, 3H), 1.26 (s, 3H), 1.24 (s, 2H), 1.09 (s, 6H), 1.07 (s, 3H), 1.00 (s, 3H). ^13^C NMR (100 MHz, CDCl_3_) δ 213.1, 211.7, 201.1, 172.2, 171.8, 169.8, 159.3, 153.1, 140.5, 138.3, 135.8, 129.9, 128.7, 120.6, 119.3, 110.6, 79.2, 77.8, 75.8, 74.1, 71.8, 68.8, 54.3, 50.4, 50.1, 48.7, 48.5, 48.2, 43.1, 42.2, 35.6, 33.9, 30.5, 29.5, 28.9, 28.8, 27.0, 26.4, 23.7, 22.0, 21.7, 21.4, 20.2, 19.8, 18.9. HRMS (ESI) calcd. for C_49_H_64_N_2_NaO_16_S [M + Na]^+^ 991.3869, found 991.3873.

*4-((4-(((4-(((9R,13R,14S,16R)-17-((R,E)-6-Acetoxy-2-hydroxy-6-methyl-3-oxohept-4-en-2-yl)-2-hydroxy-4,4,9,13,14-pentamethyl-3,11-dioxo-2,3,4,7,8,9,10,11,12,13,14,15,16,17-tetradecahydro-1H-cyclopenta[a]phenanthren-16-yl)oxy)-4-oxobutanoyl)oxy)methyl)benzyl)oxy)-3-(phenylsulfonyl)-1,2,5-oxadiazole 2-oxide* (**10o**) (63% yield in two steps). (purity: 95%). ^1^H NMR (400 MHz, CDCl_3_) δ 8.07 – 7.98 (m, 2H), 7.74 (d, *J* = 7.5 Hz, 1H), 7.59 (t, *J* = 7.9 Hz, 2H), 7.45 (d, *J* = 8.1 Hz, 2H), 7.39 (d, *J* = 8.2 Hz, 2H), 7.17 (d, *J* = 15.6 Hz, 1H), 7.17 (d, *J* = 15.6 Hz, 1H), 6.40 (d, *J* = 15.6 Hz, 1H), 5.76 (d, *J* = 5.5 Hz, 1H), 5.44 (s, 2H), 5.21 (t, *J* = 7.9 Hz, 1H), 5.15 (s, 2H), 4.45–4.36 (m, 1H), 4.28 (s, 1H), 3.60 (d, *J* = 3.9 Hz, 1H), 3.25 (d, *J* = 14.6 Hz, 1H), 2.78–2.25 (m, 10H), 2.05–1.86 (m, 6H), 1.57 (s, 3H), 1.54 (s, 3H), 1.41 (s, 3H), 1.33 (s, 3H), 1.29 (s, 3H), 1.25 (d, *J* = 6.5 Hz, 4H), 1.07 (s, 3H), 1.01 (s, 3H). ^13^C NMR (101 MHz, CDCl_3_) δ 213.0, 211.7, 201.0, 172.2, 171.8, 169.8, 158.7, 153.2, 140.5, 138.1, 137.0, 135.8, 133.9, 129.8, 128.7, 128.6, 128.5, 120.5, 119.3, 110.6, 79.2, 77.8, 74.1, 72.3, 71.7, 66.0, 54.3, 50.3, 50.1, 48.6, 48.5, 48.1, 43.2, 42.2, 36.1, 33.8, 29.5, 29.0, 28.8, 26.9, 26.4, 23.9, 23.7, 22.0, 21.4, 20.2, 19.8, 18.9. HRMS (ESI) calcd. for C_52_H_62_N_2_NaO_16_S [M + Na]^+^ 1025.3712, found 1025.3718.

#### 3.1.10. Procedure for the Synthesis of Compound **11**

*4-(((2S,9R,13R,14S,16R)-17-((R,E)-6-Acetoxy-2-hydroxy-6-methyl-3-oxohept-4-en-2-yl)-2-hydroxy-4,4,9,13,14-pentamethyl-3,11-dioxo-2,3,4,7,8,9,10,11,12,13,14,15,16,17-tetradecahydro-1H-cyclopenta[a]phenanthren-16-yl)oxy)-4-oxobutanoic acid* (**11**). Compound **8** (77 mg, 0.1 mmol) was dissolved in THF (5 mL). HOAc (28 μL, 0.5 mmol) and TBAF (131 mg, 0.5 mmol) were added to the mixture. The mixture was stirred at room temperature for 24 h, and then diluted with ethyl acetate (20 mL). The organic phase was washed with H_2_O (3 × 20 mL), dried over Na_2_SO_4_, and concentrated. The residue was purified by column chromatography on silica gel to obtain a white solid **11** (52 mg, 79% yield). (purity: 95%). ^1^H NMR (400 MHz, CDCl_3_) δ 7.16 (d, *J* = 15.6 Hz, 1H), 6.41 (d, *J* = 15.6 Hz, 1H), 5.77 (d, *J* = 5.5 Hz, 1H), 5.22 (t, *J* = 7.8 Hz, 1H), 4.41 (dd, *J* = 12.9, 6.0 Hz, 1H), 3.25 (d, *J* = 14.6 Hz, 1H), 2.77–2.67 (m, 3H), 2.58 (dd, *J* = 12.2, 6.5 Hz, 2H), 2.43 (t, *J* = 6.7 Hz, 2H), 2.38 (d, *J* = 7.8 Hz, 1H), 2.31 (ddd, *J* = 12.5, 5.8, 3.4 Hz, 1H), 2.04–1.93 (m, 6H), 1.59 (s, 3H), 1.55 (s, 3H), 1.41 (s, 3H), 1.39 (s, 1H), 1.33 (s, 3H), 1.28 (d, *J* = 4.5 Hz, 6H), 1.25 (s, 3H), 1.08 (s, 3H), 1.01 (s, 3H). ^13^C NMR (100 MHz, CDCl_3_) δ 213.1, 211.8, 201.1, 176.9, 171.7, 170.0, 153.1, 140.5, 120.5, 119.3, 79.4, 77.9, 74.3, 71.8, 54.3, 50.4, 50.1, 48.7, 48.5, 48.1, 43.2, 42.2, 36.09, 33.9, 29.5, 28.9, 28.8, 27.0, 26.3, 23.9, 23.8, 21.9, 21.4, 20.2, 19.9, 18.8. HRMS (ESI) calcd. for C_36_H_50_NaO_11_ [M + Na]^+^ 681.3245, found 681.3250.

### 3.2. Biological Assay

#### 3.2.1. Cell Culture 

HepG-2 human hepatocellular carcinoma cells and L-O2 human normal liver cells were maintained in DMEM culture medium supplemented with 10% FBS at 37 °C under a humidified atmosphere with 5% CO_2_.

#### 3.2.2. MTT Assay

HepG-2 and L-O2 cells in logarithmic growth phase were seeded into a 96-well plate at 5 × 10^3^/well/200 µL. After 12 h, compounds at different concentrations were added and incubated for 72 h at 37 °C, 5% CO_2_. Then, 20 µL MTT (5 mg/mL) was added into each well and incubated for 4 h. The supernatant was discarded, and the precipitate was dissolved with DMSO. The absorbance of the optical density (OD) at 570 nm was detected using a microplate reader and the IC_50_ was calculated with GraphPad Prism 5.

#### 3.2.3. Cell Apoptosis Assay

HepG-2 cells were seeded into a 24-well plate with 5 × 10^4^/well/1 mL. After 12 h, cells were treated with compound **10b** at different concentrations for 48 h. The cells were collected, washed with cold PBS, and stained with Annexin-V/PI according to the manufacturer’s instructions. Then, the cells were analyzed with flow cytometry.

#### 3.2.4. Western Blot Assay

HepG-2 cells were treated with compound **10b** for 24 h, and the cell lysates were collected. Then, the total protein was separated by SDS-PAGE and transferred to PVDF membranes. The membranes were incubated with primary antibodies (1:1000) at 4 °C overnight. After being washed with PBST (PBS + 0.5% Tween 20) 5 times, the membranes were incubated with HRP-conjugated secondary antibodies (1:5000 dilution) for 2 h at room temperature. The membranes were then washed with PBST 5 times and the protein blots were detected by ECL chemiluminescence.

#### 3.2.5. Animal Assay

Five-week-old Balb/C mice were purchased from the Chinese Academy of Sciences and maintained under specific pathogen free (SPF) conditions. The mice were divided into a control group, a cucurbitacin B group, and a compound **10b** group. The control group mice were treated with vehicle buffer intravenously. The cucurbitacin B group mice were treated with cucurbitacin B at doses of 2 mg/kg, 5 mg/kg, 10 mg/kg, 20 mg/kg, and 50 mg/kg, respectively (three mice each dose). The compound **10b** group mice were treated with compound **10b** at doses of 2 mg/kg, 5 mg/kg, 10 mg/kg, 20 mg/kg, and 50 mg/kg respectively (three mice each dose). The clinical symptoms, deaths, and body weight were observed and recorded.

## 4. Conclusion

In summary, 37 cucurbitacin B derivatives were synthesized and evaluated for their anti-hepatocellular carcinoma activities against the HepG-2 cell line. These compounds were also tested for their toxicity against the L-O2 normal cell line. These studies indicated that the introduction of a phenylsulfonyl-substituted furoxan NO-releasing moiety could reduce the toxicity against normal cells and maintain potent anti-HCC activity to some extent. The compound with the most potential, **10b**, exhibited potent activity against the HepG-2 cell line, with an IC_50_ value of 0.63 μM. Moreover, compound **10b** showed the highest TI value (4.71), which is a 14.7-fold improvement compared to its parent compound cucurbitacin B (Table 2). The preliminary study of the molecular mechanism of **10b** indicated that **10b** could inhibit P-STAT3 to induce the activation of mitochondrial apoptotic pathways (Figure 2). An in vivo acute toxicity study indicated that compound **10b** has preferable safety and tolerability compared with cucurbitacin B (Figure 3). These findings indicate that compound **10b** might be considered as a lead compound for exploring effective anti-HCC drugs.

## Figures and Tables

**Table 1:** The inhibitory activity and clog P of **3a**–**3q** and **4** against the HepG-2 and L-O2 cell lines.

Compound	IC_50_^a^(μM)		TI^b^	Clog P ^d^
	HepG-2	L-O2		
**1**	0.060 ± 0.02	0.019 ± 0.003	0.32	2.96
**3a**	1.01 ± 0.04	1.02 ± 0.58	1	4.19
**3b**	0.54 ± 0.04	0.52 ± 0.08	0.96	4.57
**3c**	2.45 ± 1.75	2.37 ± 0.09	0.97	5.13
**3d**	4.89 ± 0.92	8.01 ± 0.47	1.64	5.10
**3e**	9.28 ± 0.37	5.50 ± 2.48	0.59	5.70
**3f**	0.93 ± 0.06	0.41 ± 0.11	0.44	5.85
**3g**	0.22 ± 0.11	0.042 ± 0.001	0.19	5.90
**3h**	9.62 ± 1.34	6.90 ± 1.37	0.72	6.09
**3i**	7.42 ± 0.40	4.42 ± 0.97	0.60	5.73
**3j**	4.67 ± 0.28	3.57 ± 2.58	0.76	6.30
**3k**	2.99 ± 2.06	1.93 ± 0.32	0.65	6.45
**3l**	3.71 ± 1.69	2.47 ± 0.47	0.67	5.76
**3m**	0.92 ± 0.44	0.74 ± 0.15	0.8	5.33
**3n**	4.05 ± 1.39	2.48 ± 0.52	0.61	5.73
**3o**	3.31 ± 0.24	9.59 ± 2.38	2.90	5.94
**3p**	2.34 ± 0.91	1.47 ± 0.36	0.63	5.38
**3q**	3.85 ± 0.63	5.25 ± 1.63	1.36	5.95
**4**	>20	>20	N.D ^c^	8.78
ADR ^e^	0.30 ± 0.05	0.041 ± 0.013	0.14	-

^a^ All values are the mean of three independent experiments. ^b^ TI: Therapeutic Index = IC_50 (L-O2)_/IC_50 (HepG-2)._^c^ N.D., Not determined. ^d^ clog P was calculated by ChemBioDraw Ultra 14.0. ^e^ ADR, adriamycin, was used as a positive control.

**Table 2:** The inhibitory activity and clog P of **9a**–**9c**, **10a**–**10o**, and **11** against the HepG-2 and L-O2 cell lines.

Compound	IC_50_^a^(μM)		TI ^b^	Clog P ^d^
	HepG-2	L-O2		
**1**	0.060 ± 0.02	0.019 ± 0.003	0.32	2.96
**9a**	0.46 ± 0.14	0.69 ± 0.43	1.5	2.06
**9b**	0.53 ± 0.01	0.80 ± 0.40	1.51	3.29
**9c**	0.92 ± 0.16	2.61 ± 1.77	2.84	3.73
**10a**	0.82 ± 0.33	2.04 ± 0.67	2.49	3.57
**10b**	0.63 ± 0.29	2.97 ± 0.23	4.71	3.97
**10c**	0.52 ± 0.09	2.10 ± 1.41	4.03	4.09
**10d**	0.64 ± 0.26	2.62 ± 0.52	4.1	4.62
**10e**	1.15 ± 0.24	3.21 ± 0.09	2.79	5.15
**10f**	0.83 ± 0.38	3.25 ± 0.40	3.92	5.68
**10g**	1.19 ± 0.55	4.29 ± 0.66	3.61	6.21
**10h**	1.15 ± 0.24	1.17 ± 0.08	1.02	6.74
**10i**	6.92 ± 0.75	3.27 ± 0.43	0.47	7.27
**10j**	10.28 ± 1.11	6.69 ± 3.95	0.65	7.79
**10k**	>20	>20	N.D ^c^	8.32
**10l**	1.01 ± 0.40	2.72 ± 0.38	2.69	4.01
**10m**	1.35 ± 0.10	2.94 ± 0.09	2.18	3.85
**10n**	1.11 ± 0.22	3.31 ± 0.18	0.34	4.77
**10o**	1.02 ± 0.28	3.00 ± 0.26	2.94	5.08
**11**	>20	>20	N.D ^c^	3.43
ADR ^e^	0.30 ± 0.05	0.041 ± 0.013	0.14	-

^a^ All values are the mean of three independent experiments. ^b^ TI: Therapeutic Index = IC_50 (L-O2)_/ IC_50 (HepG-2)._^c^ N.D., Not determined. ^d^ clog P was calculated by ChemBioDraw Ultra 14.0. ^e^ ADR, adriamycin, was used as a positive control.

## Supplementary Materials

Copies of the ^1^H/^13^C NMR spectra, HPLC, and dose–response curves of all new compounds.
