# Supplementary material for: Synthesis of Cucurbitacin B Derivatives as Potential Anti-Hepatocellular Carcinoma Agents
Source: Molecules. 2018 Dec 18;23(12):3345. doi: 10.3390/molecules23123345 (PMC6321601; doi:10.3390/molecules23123345)
Supplement: Supplementary file 1 [file molecules-23-03345-s001.pdf]

Synthesis of Cucurbitacin B Derivatives as Potential Anti-hepatocellular Carcinoma Agents

Weizhi Ge,[a] Xinyi Chen,[a] Fangzhi Han,[a] Zhongquan Liu,[a] Tianpeng Wang,[a] Mengmeng Wang,[b] Yue Chen,[a,] Yahui Ding[a,]* and Quan Zhang[a,]**

[a]*State Key Laboratory of Medicinal Chemical Biology, College of Pharmacy and Tianjin Key Laboratory of Molecular Drug Research, Nankai University, Haihe Education Park, 38 Tongyan Road, Tianjin 300353, People's Republic of China*

[b]*Accendatech Company, Ltd., Tianjin 300384, People's Republic of China*

SUPPORTING INFORMATION

1. Copies of ^{1}HNMR and ^{13}C NMR··························S2

2. The purity of the compounds tested by HPLC ··························S44

3. The dose-response curves of compounds ··························S54

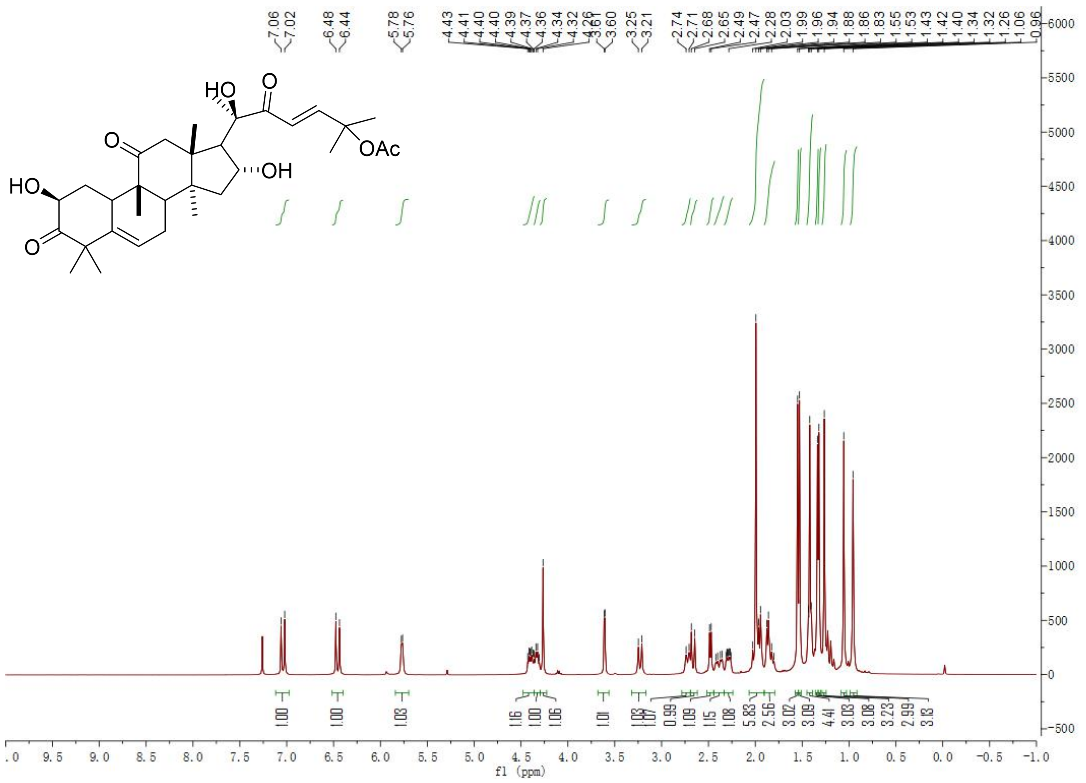

^1H NMR of cucurbitacin B (**1**)

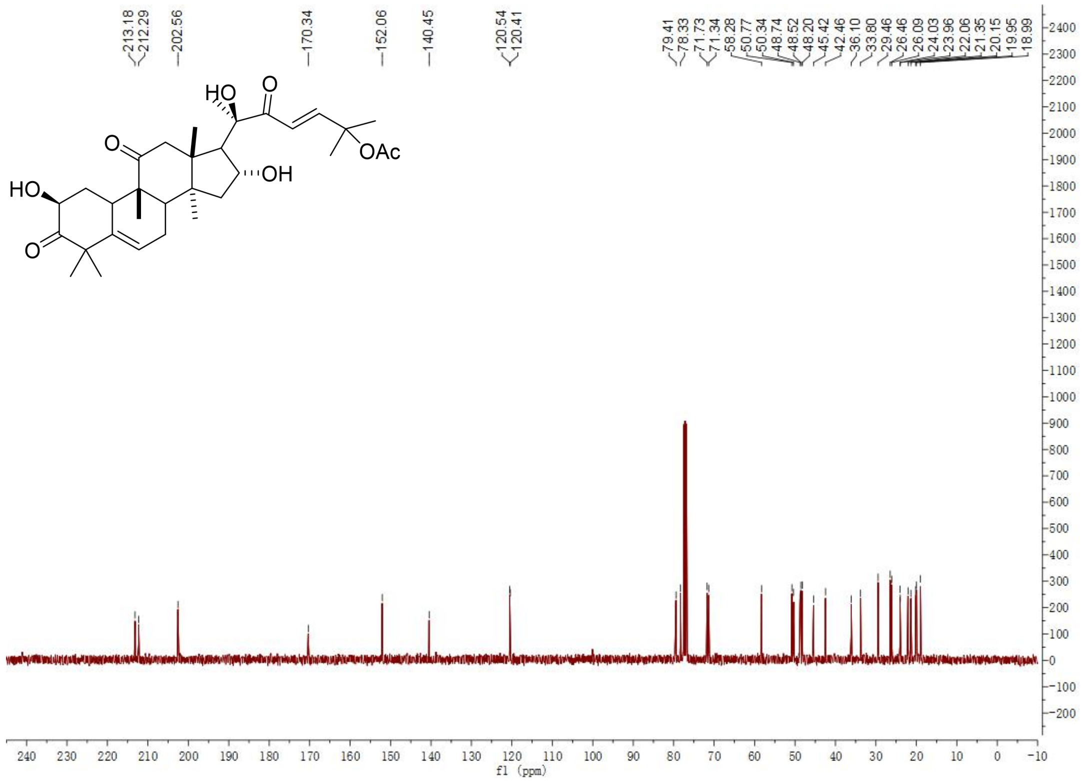

^{13}C NMR of cucurbitacin B (**1**)

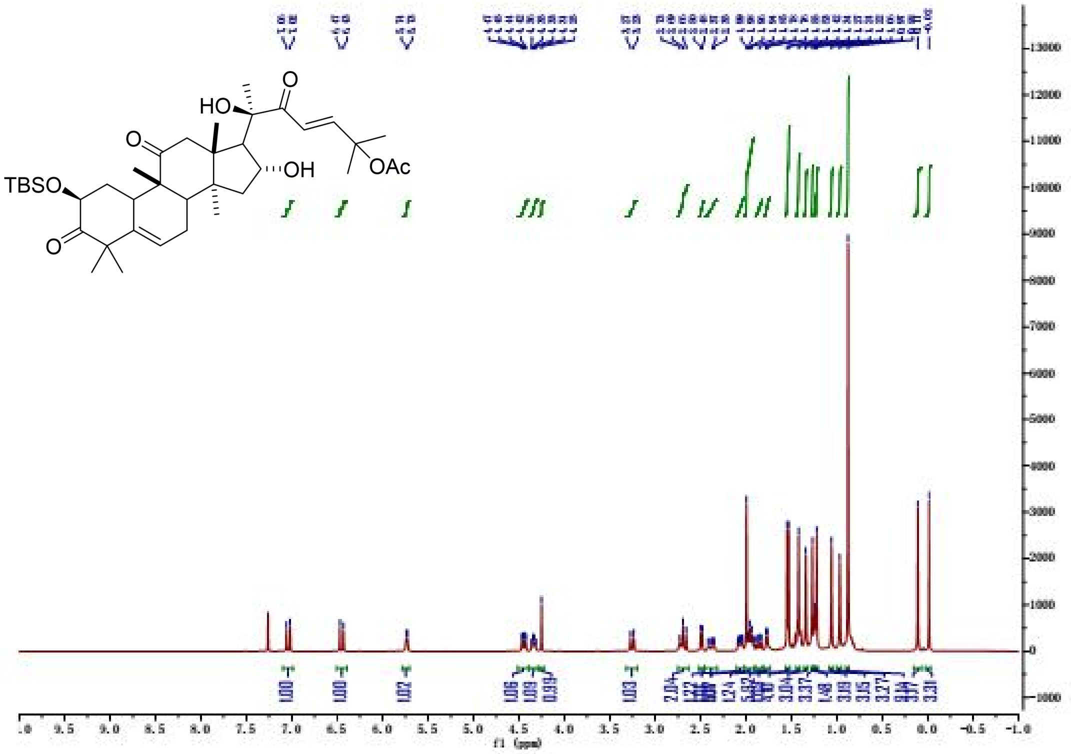

^{1}H NMR of compound **2**

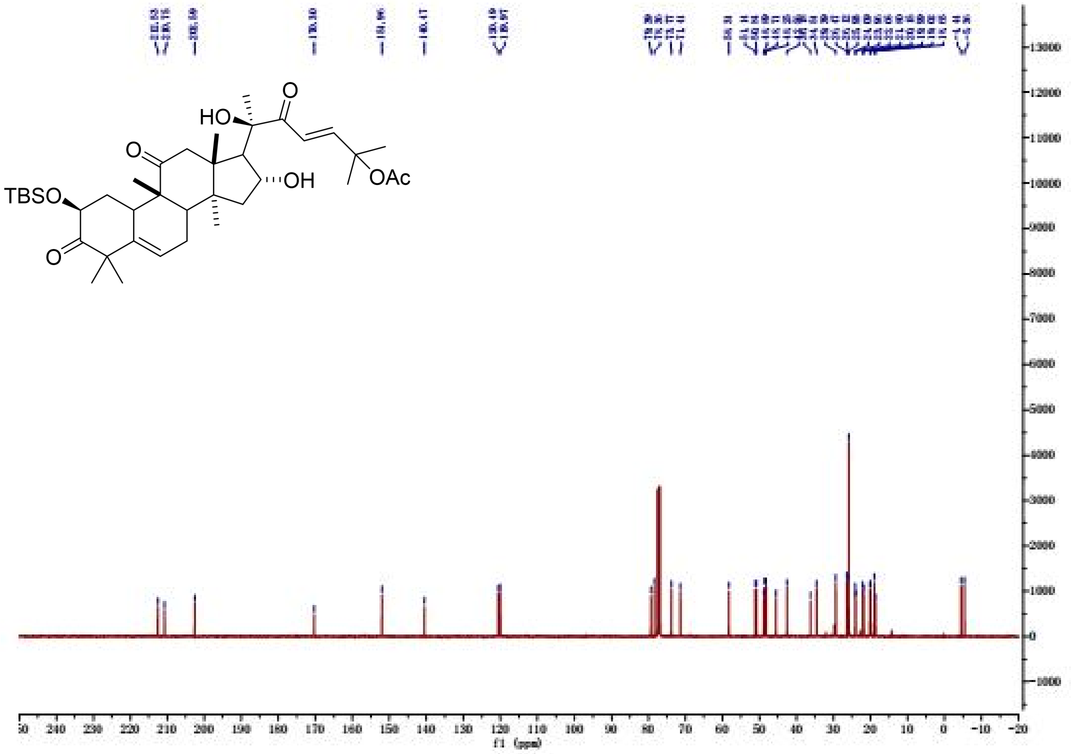

^{13}C NMR of compound **2**

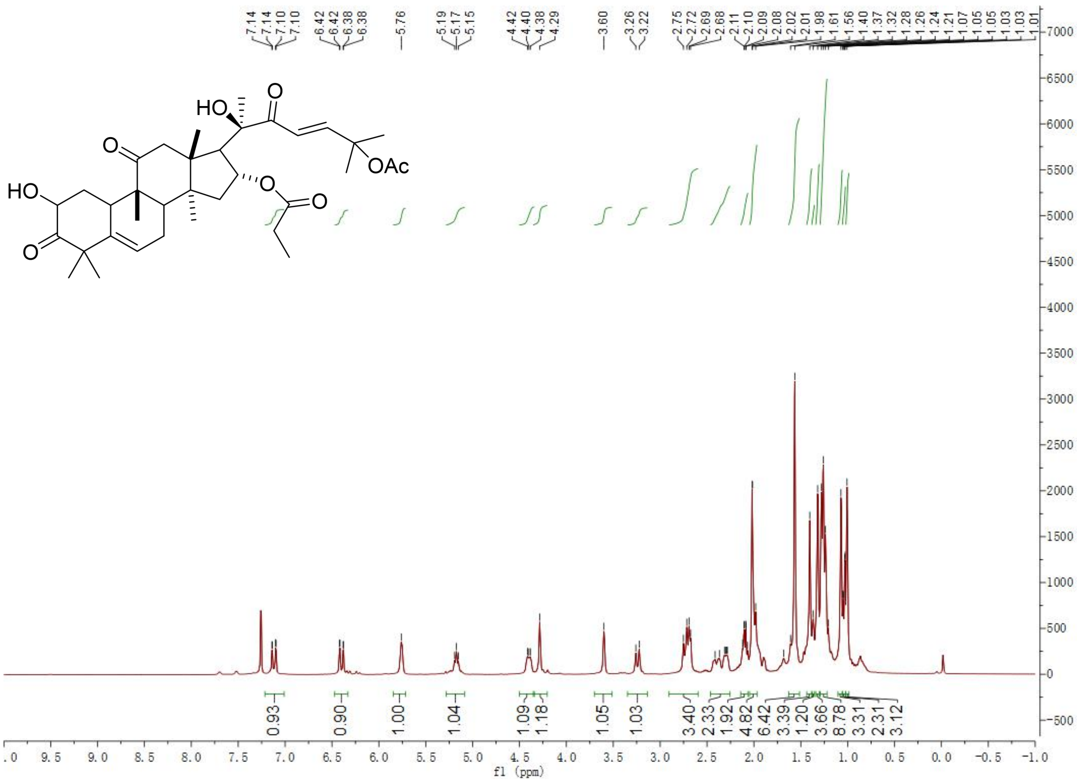

^{1}H NMR of compound **3a**

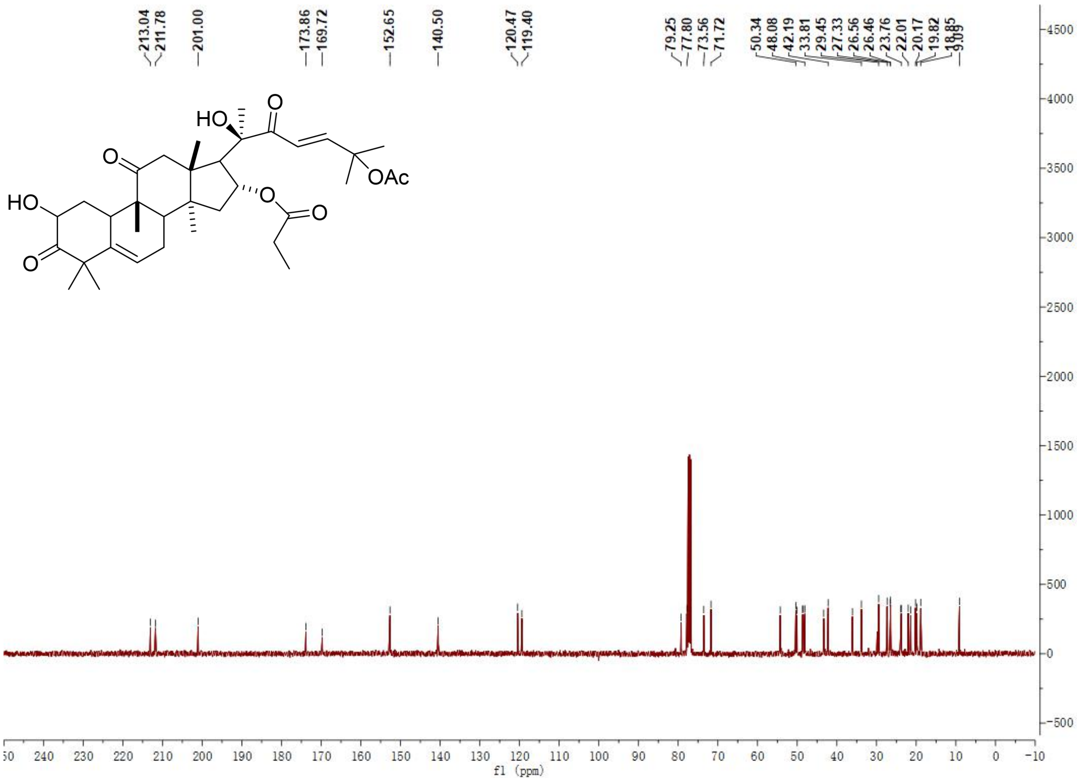

^{13}C NMR of compound **3a**

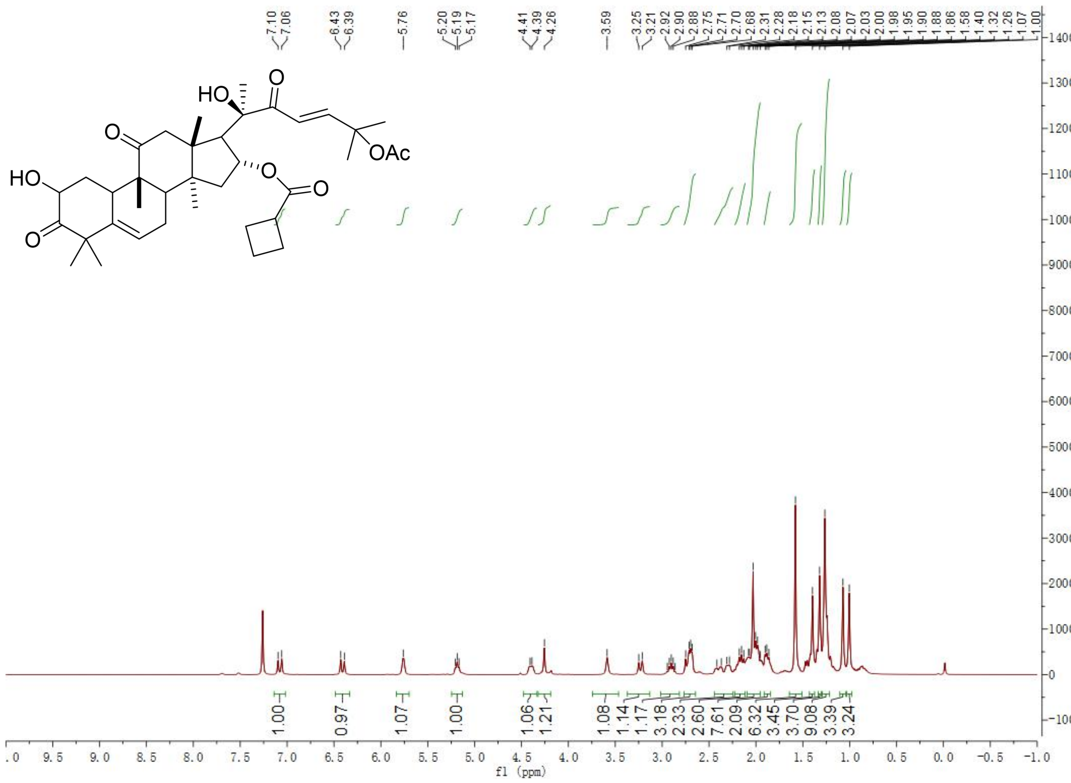

^{1}H NMR of compound **3b**

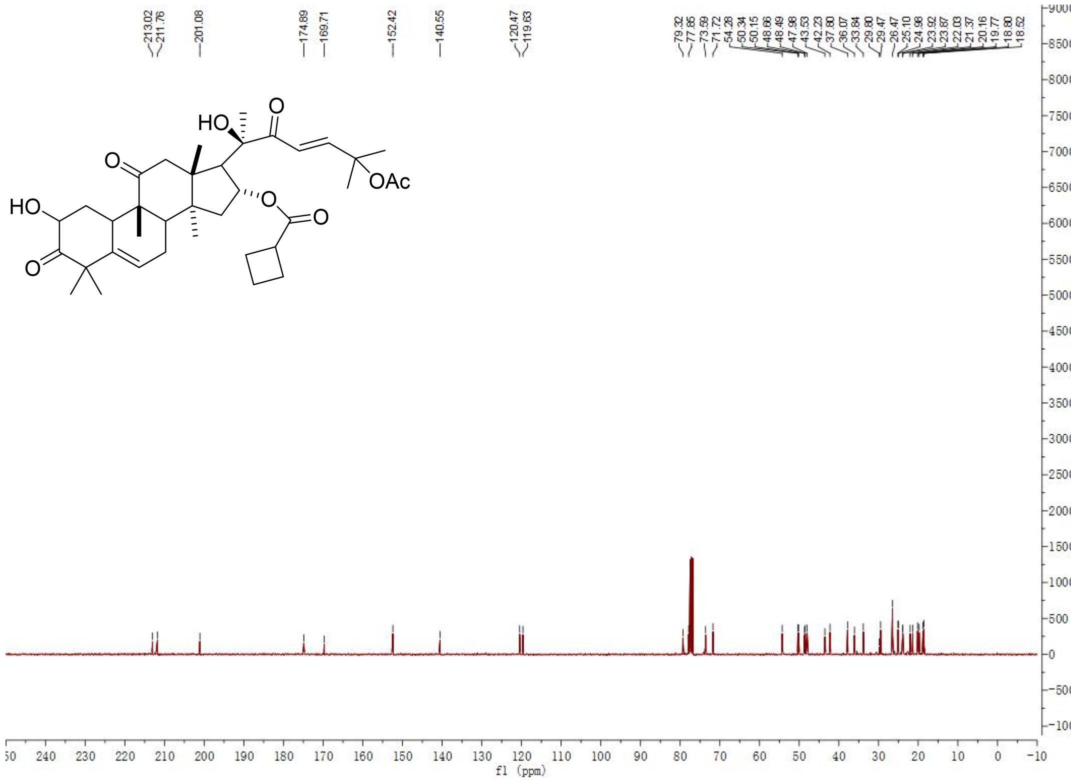

^{13}C NMR of compound **3b**

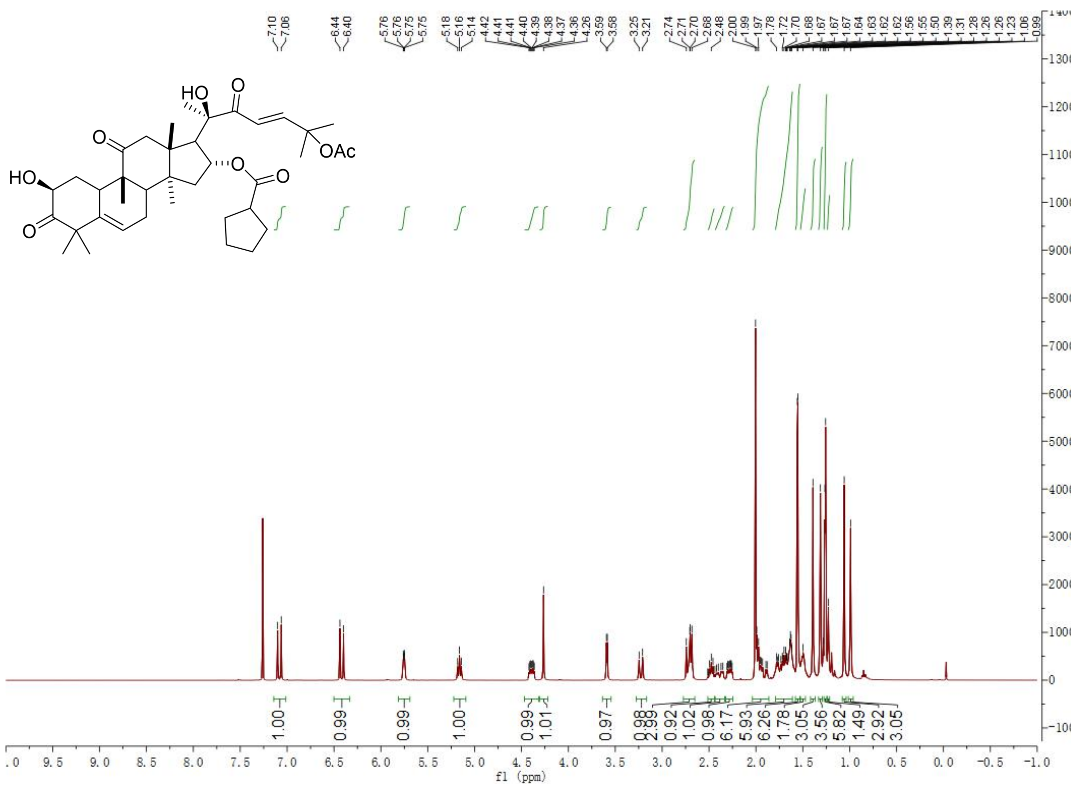

^{1}H NMR of compound **3c**

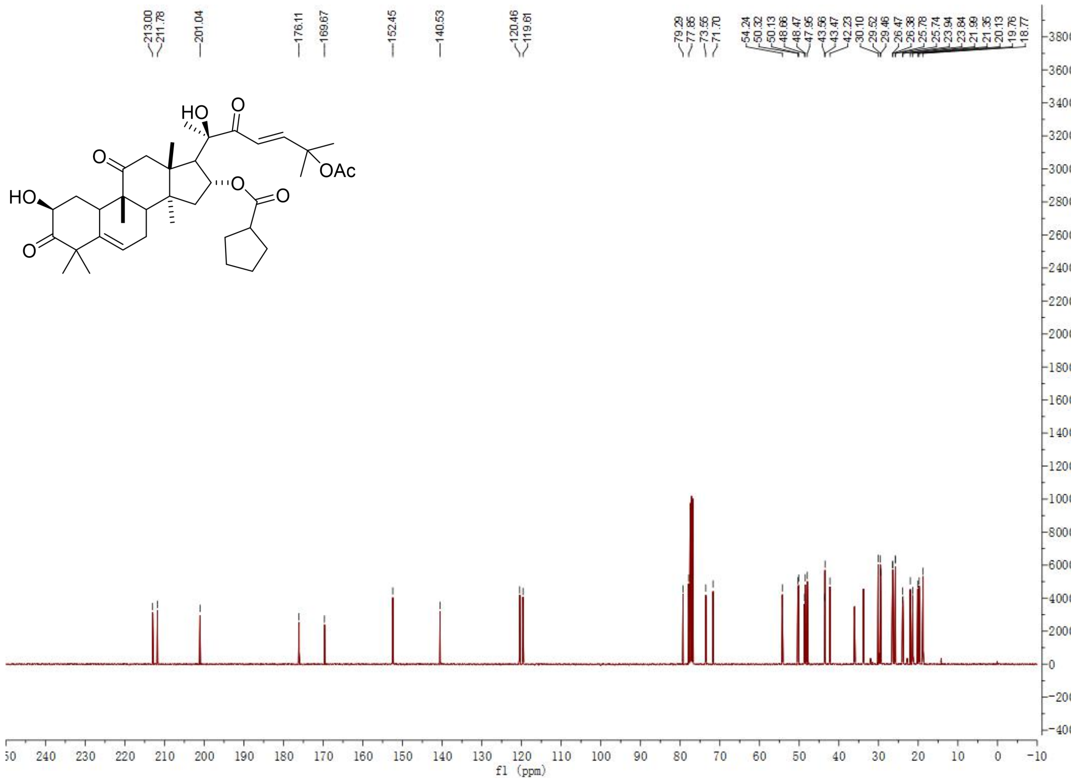

^{13}C NMR of compound **3c**

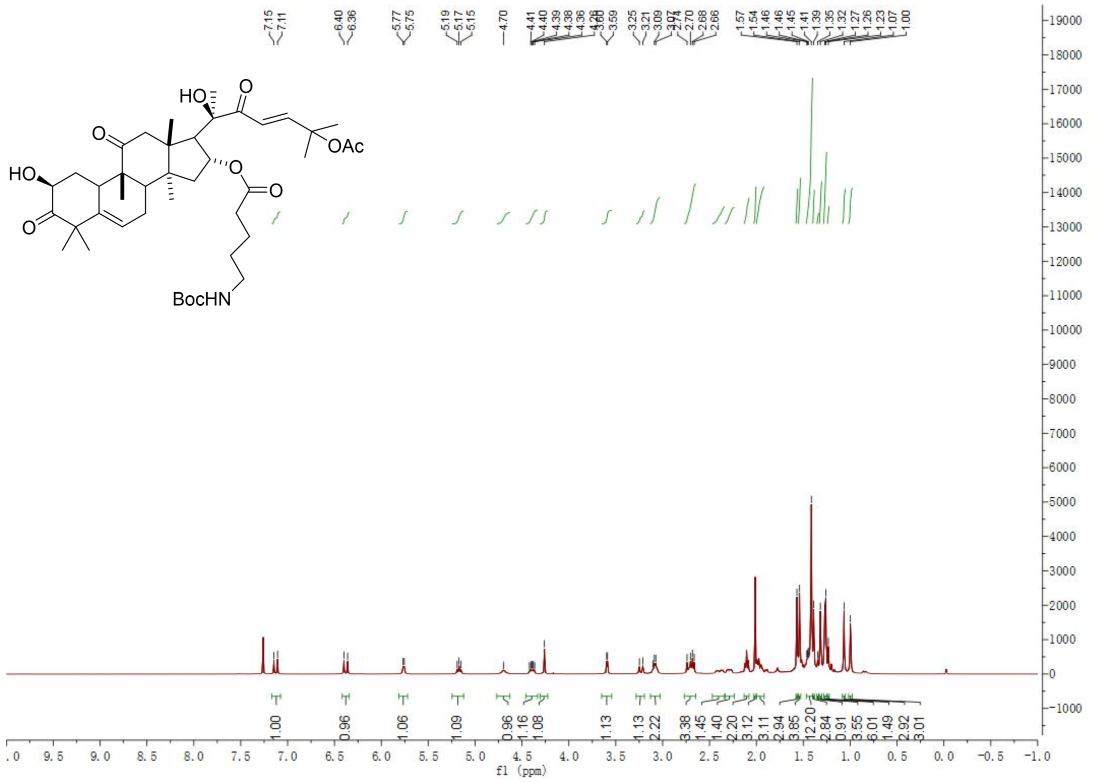

^{1}H NMR of compound **3d**

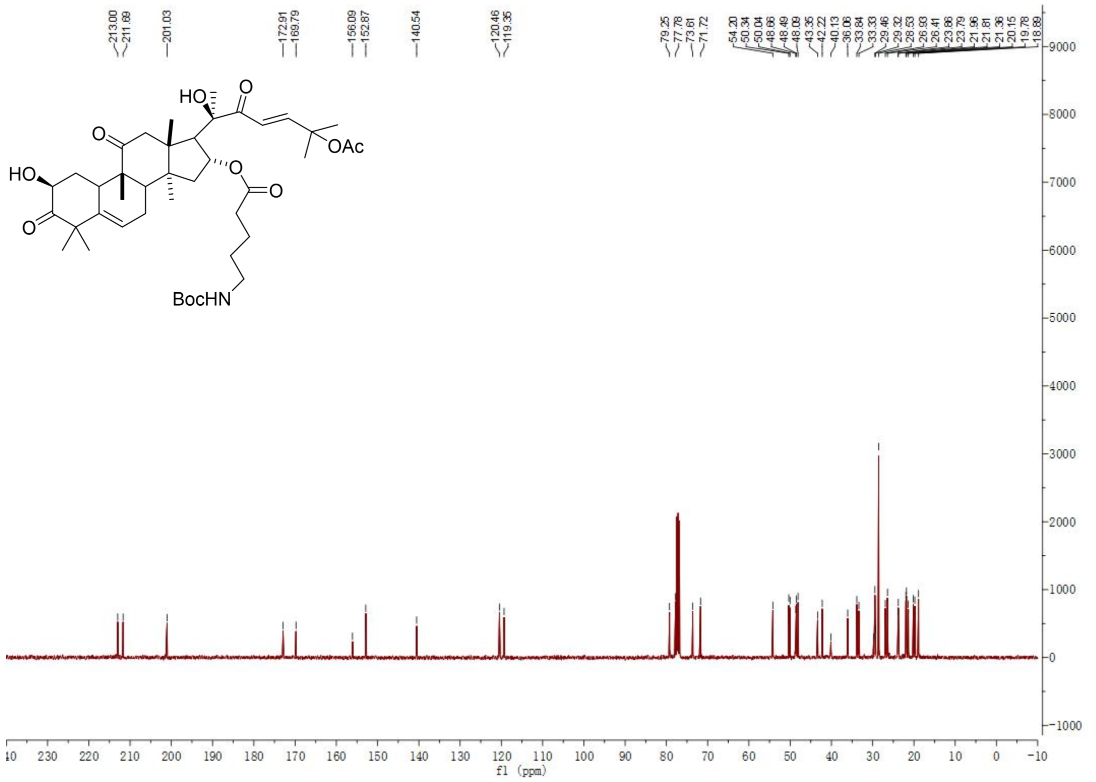

^{13}C NMR of compound **3d**

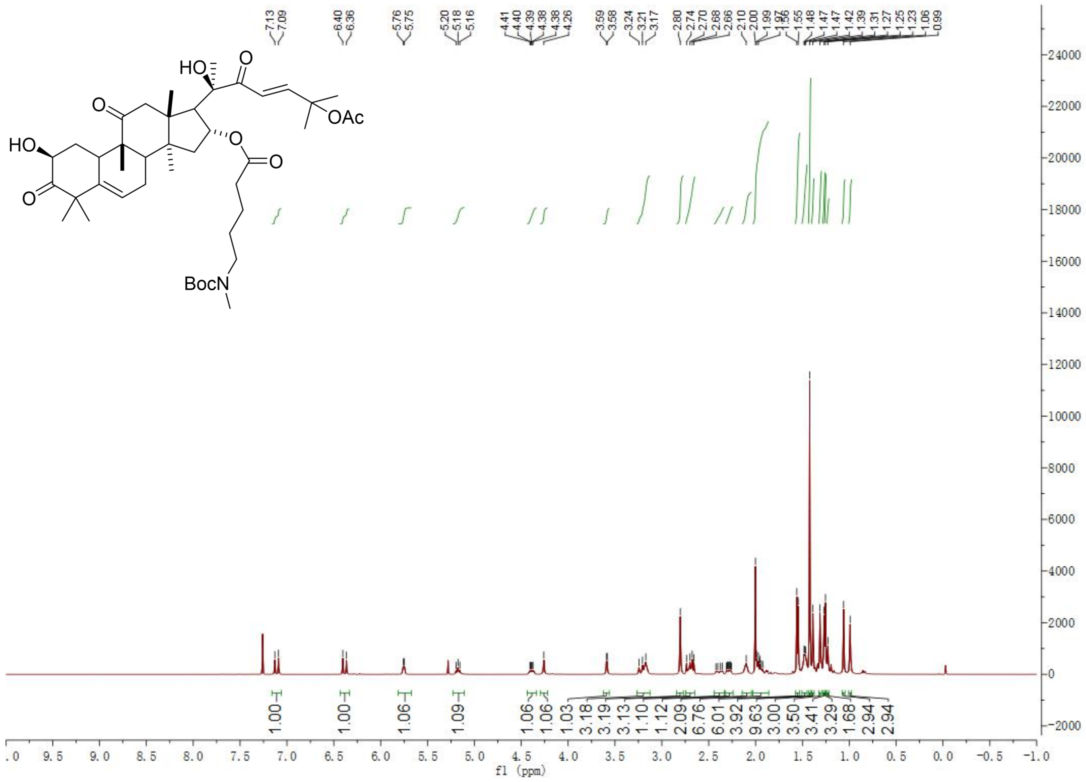

^{1}H NMR of compound **3e**

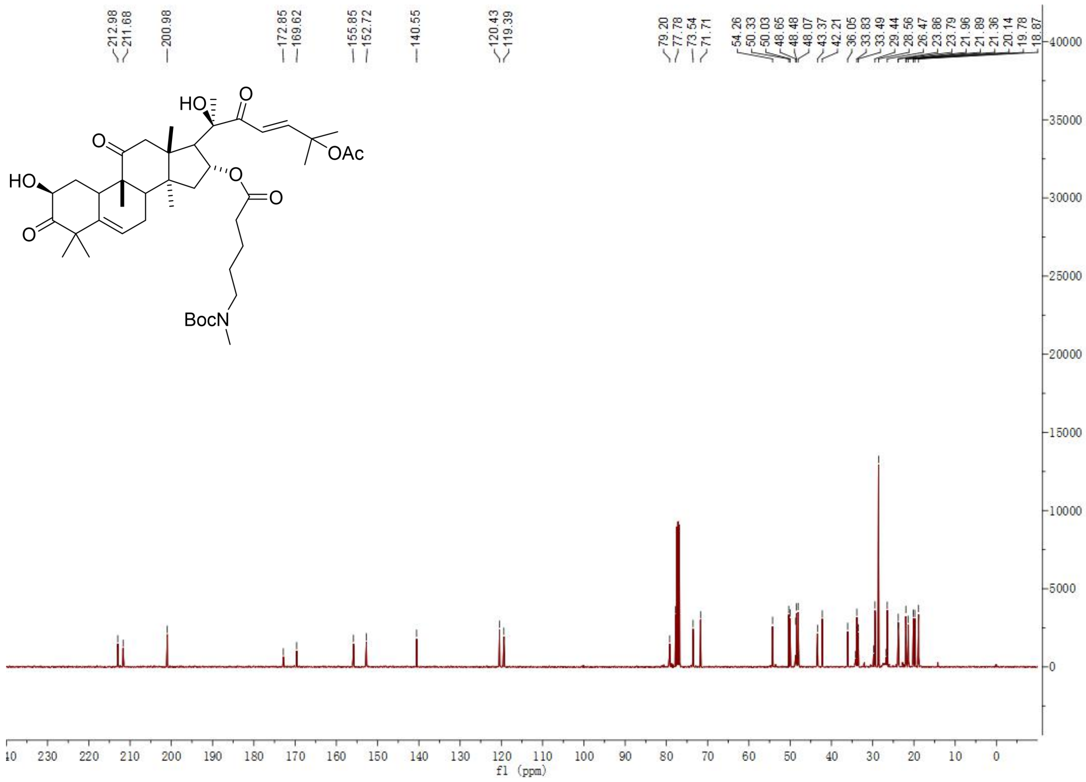

^{13}C NMR of compound **3e**

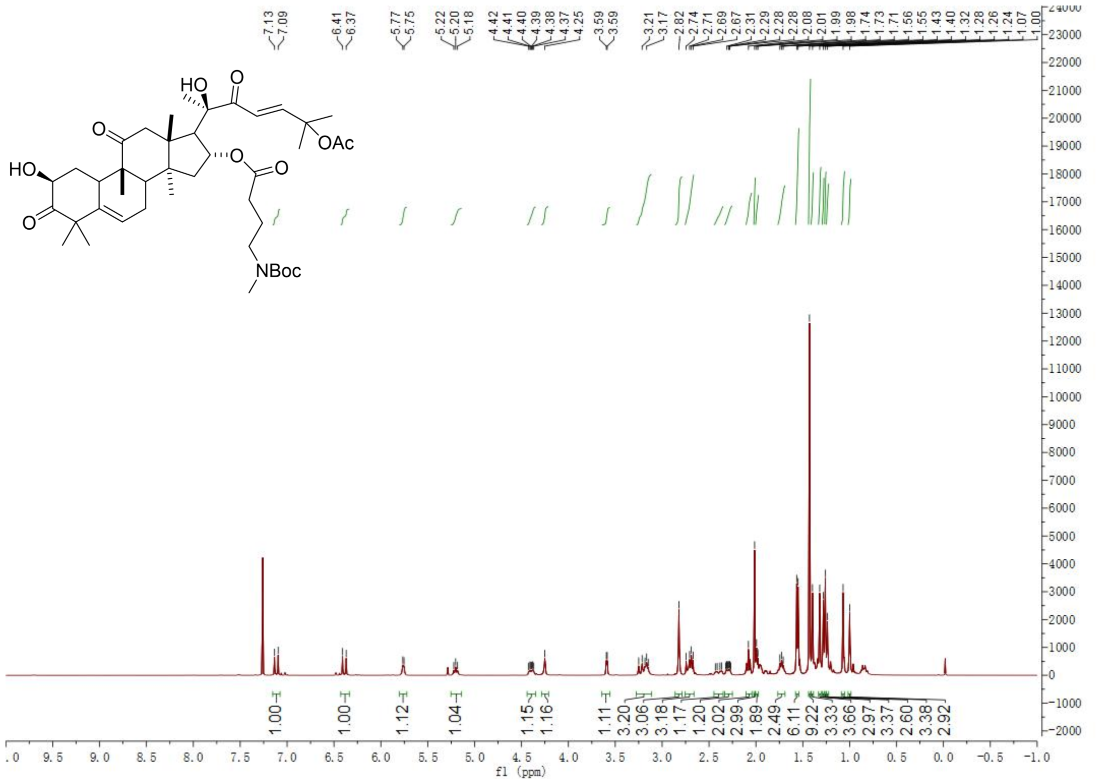

^{1}H NMR of compound **3f**

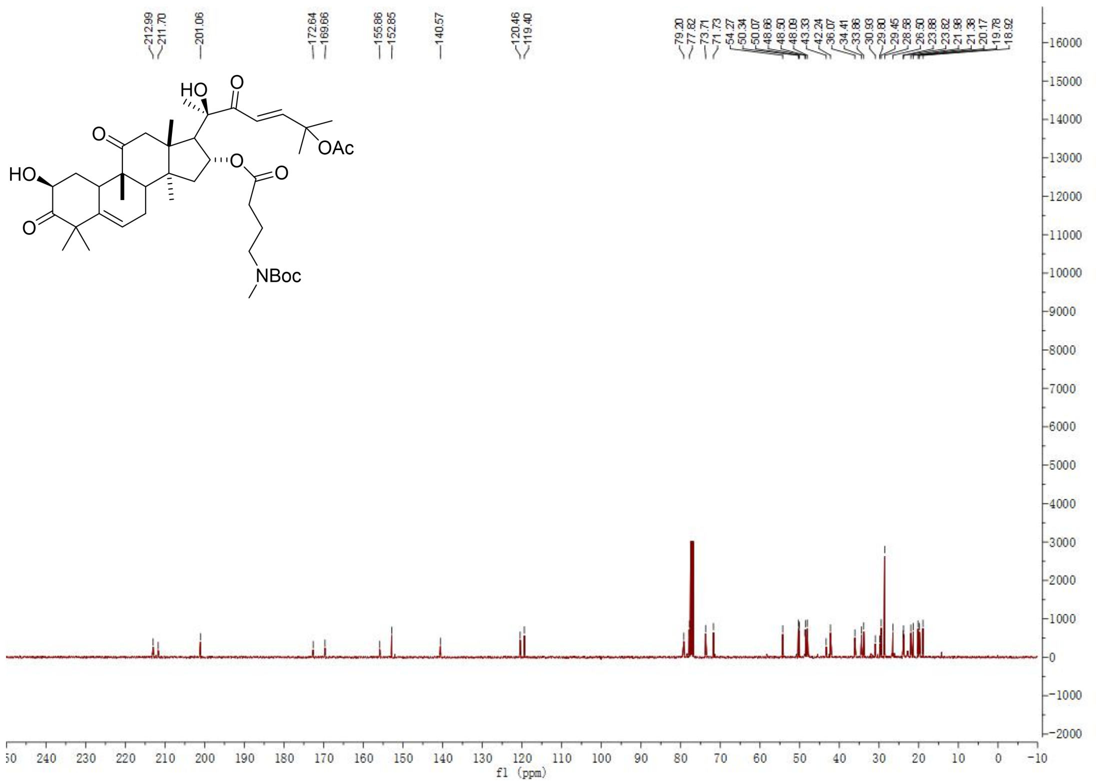

^{13}C NMR of compound **3f**

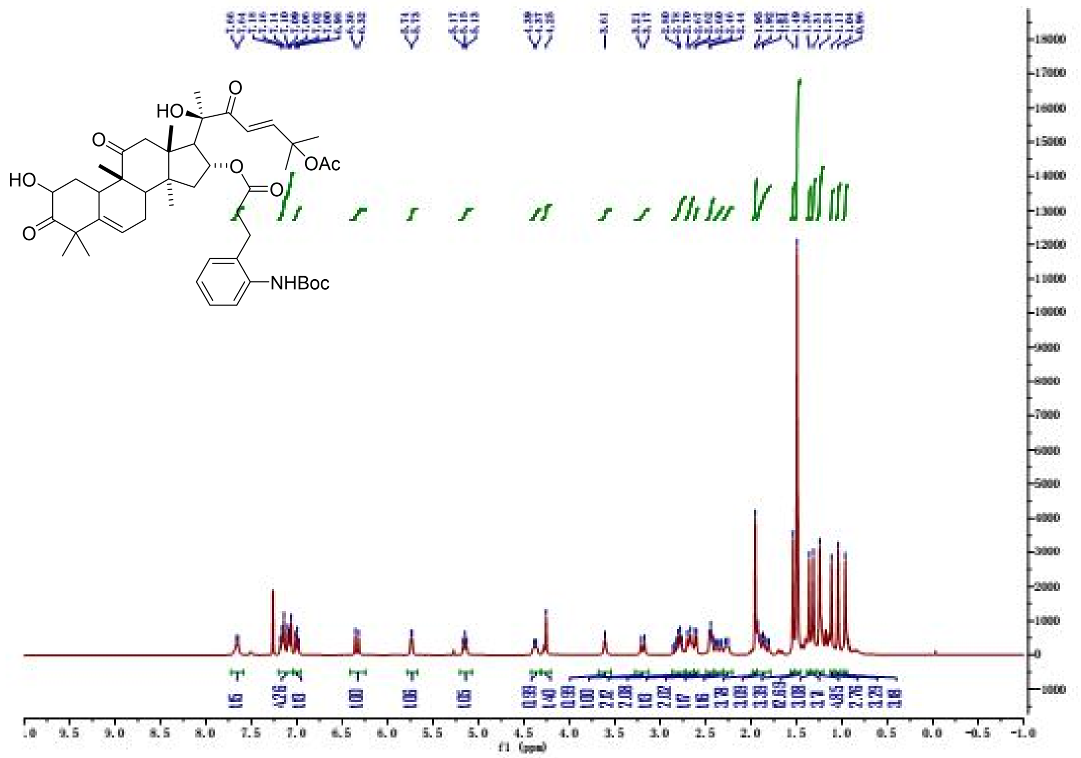

^{1}H NMR of compound **3g**

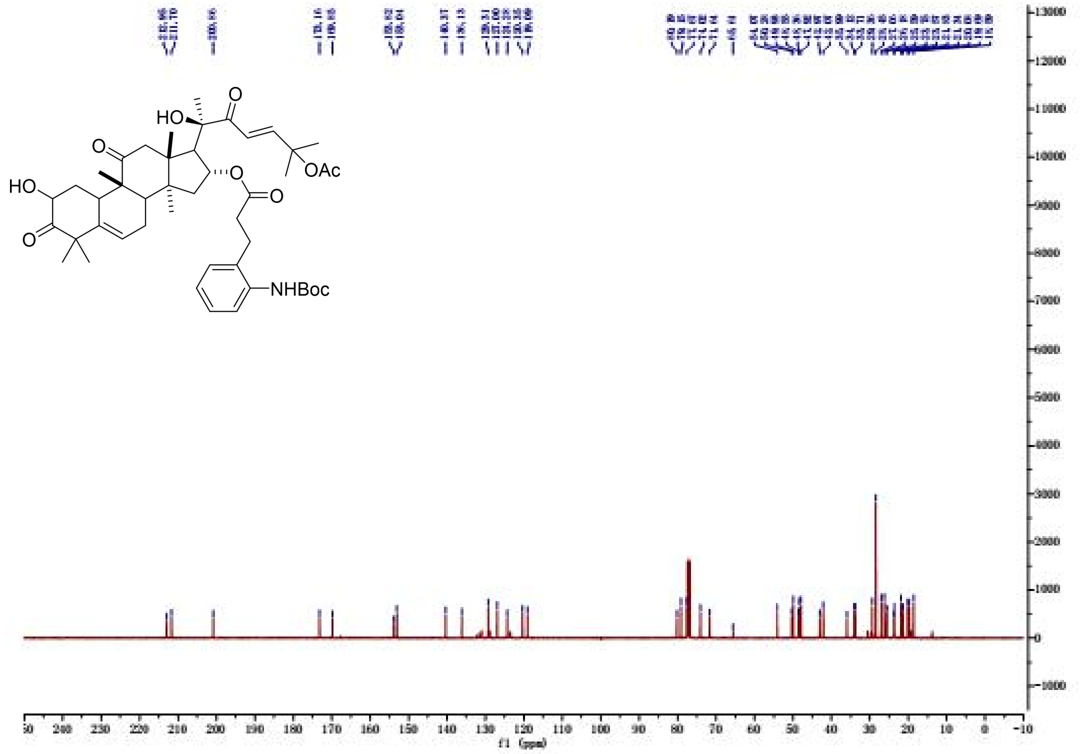

^{13}C NMR of compound **3g**

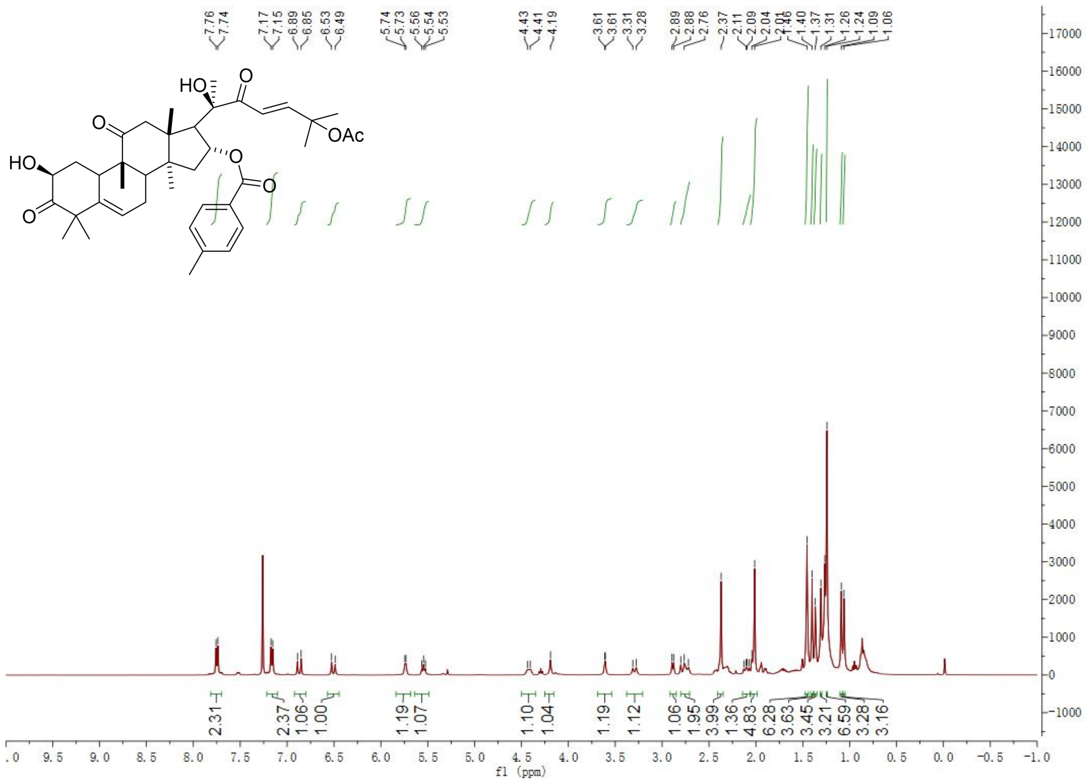

^1H NMR of compound **3h**

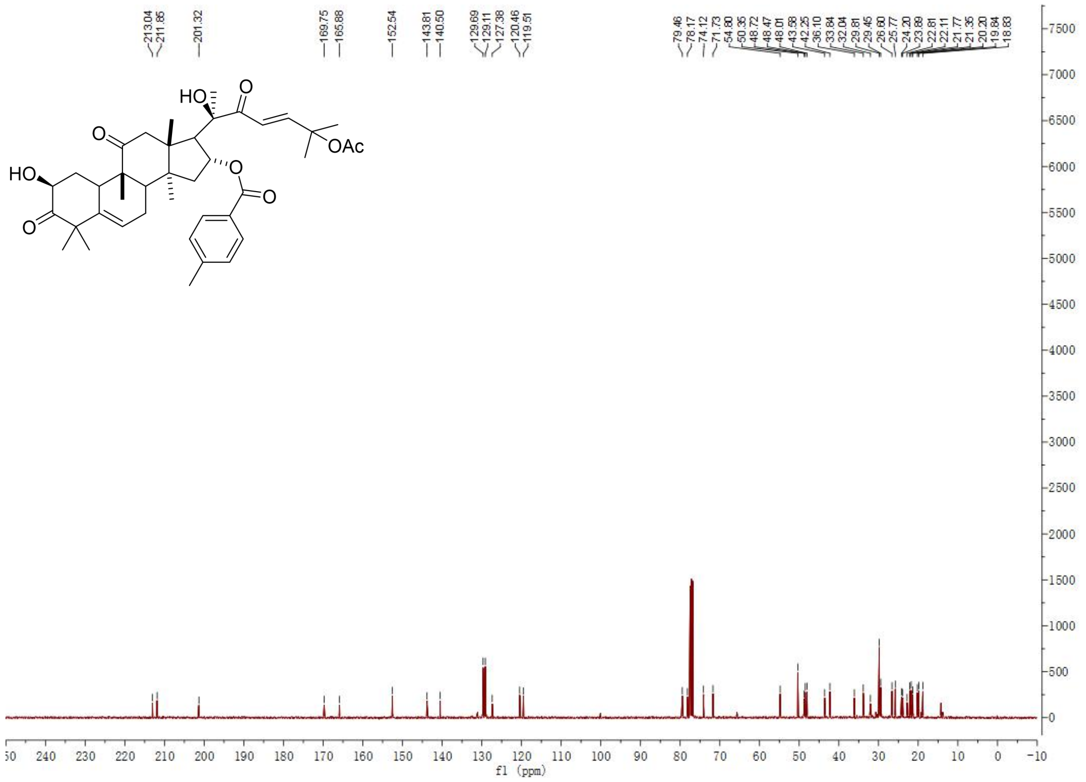

^{13}C NMR of compound **3g**

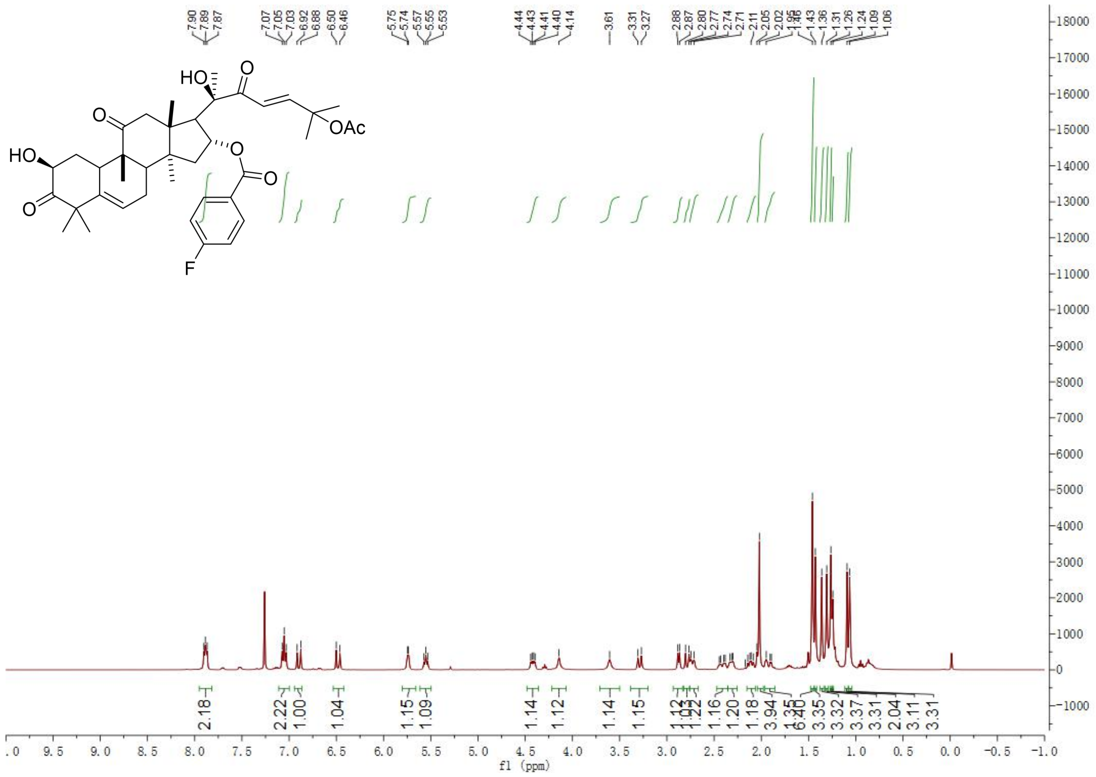

^{1}H NMR of compound **3i**

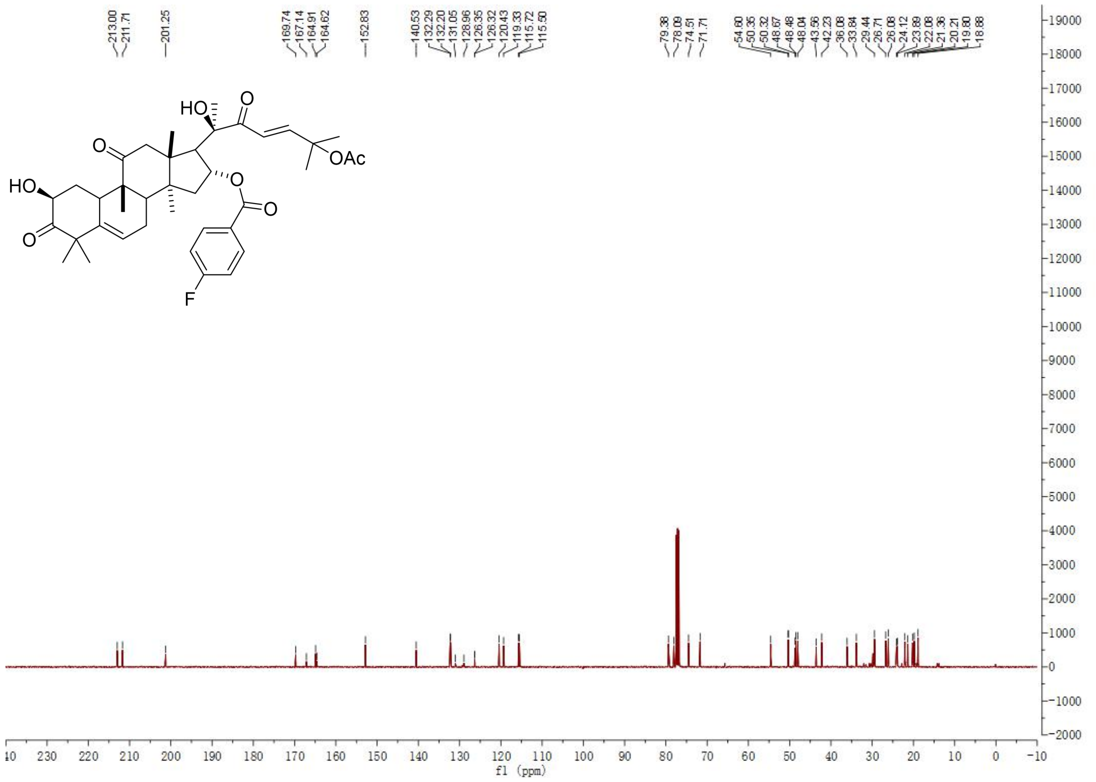

^{13}C NMR of compound **3i**

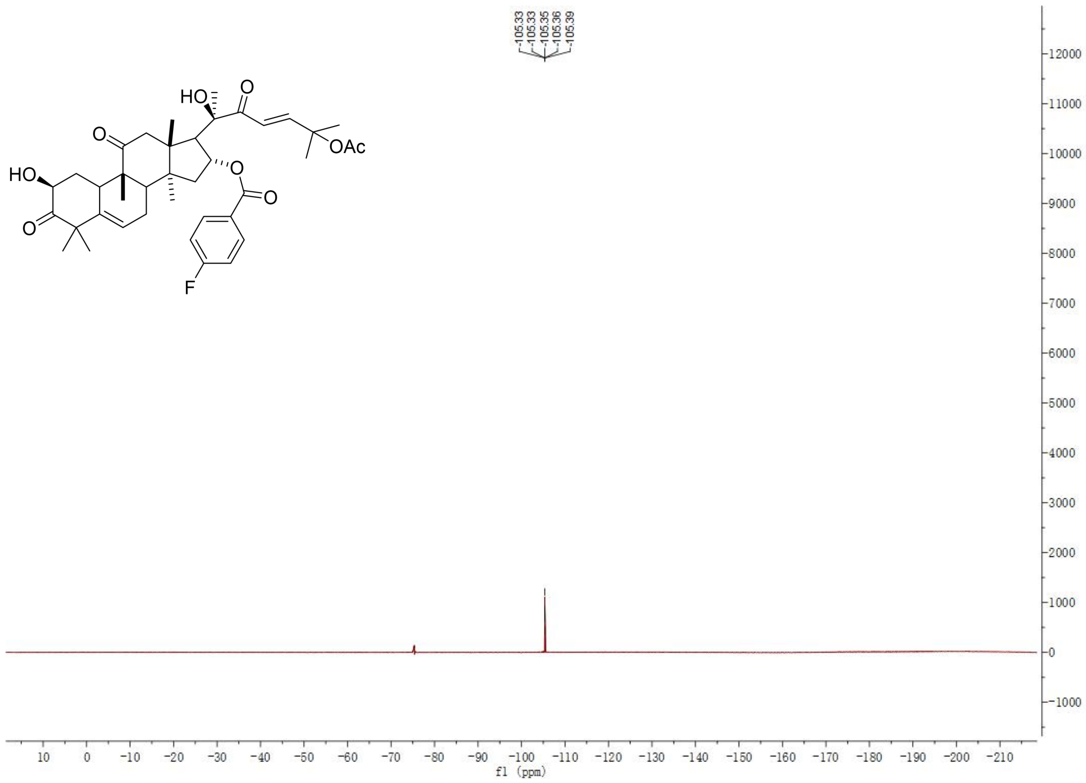

^{19}F NMR of compound **3i**

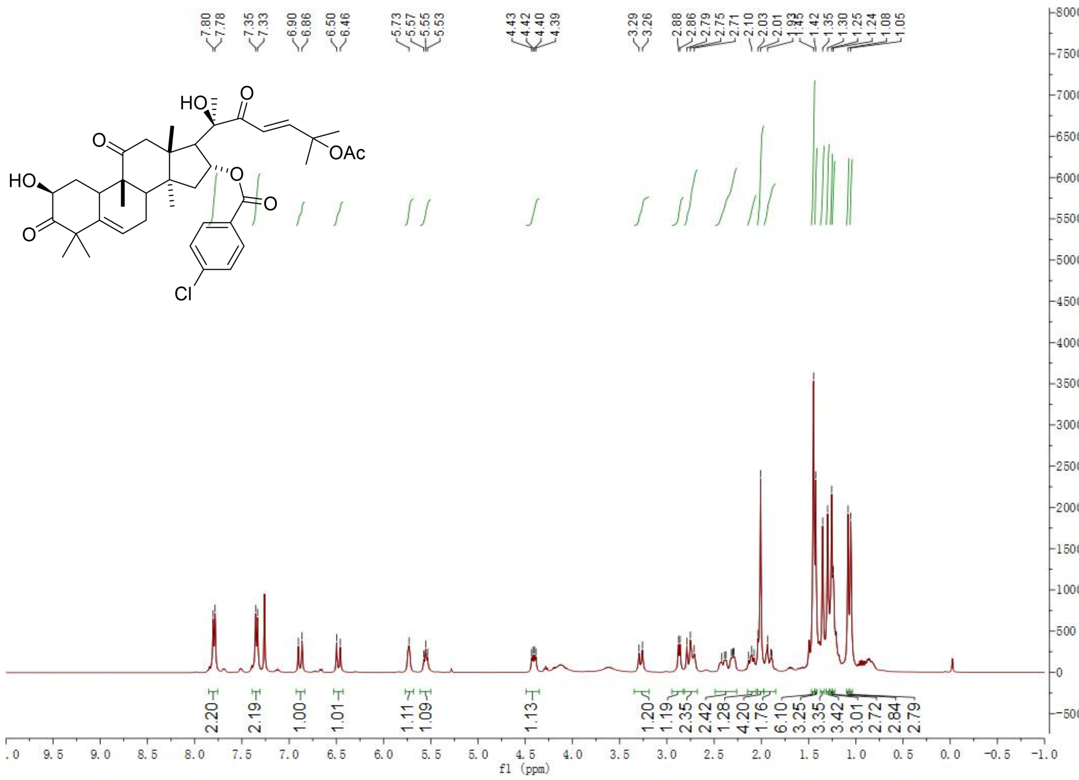

^1H NMR of compound **3j**

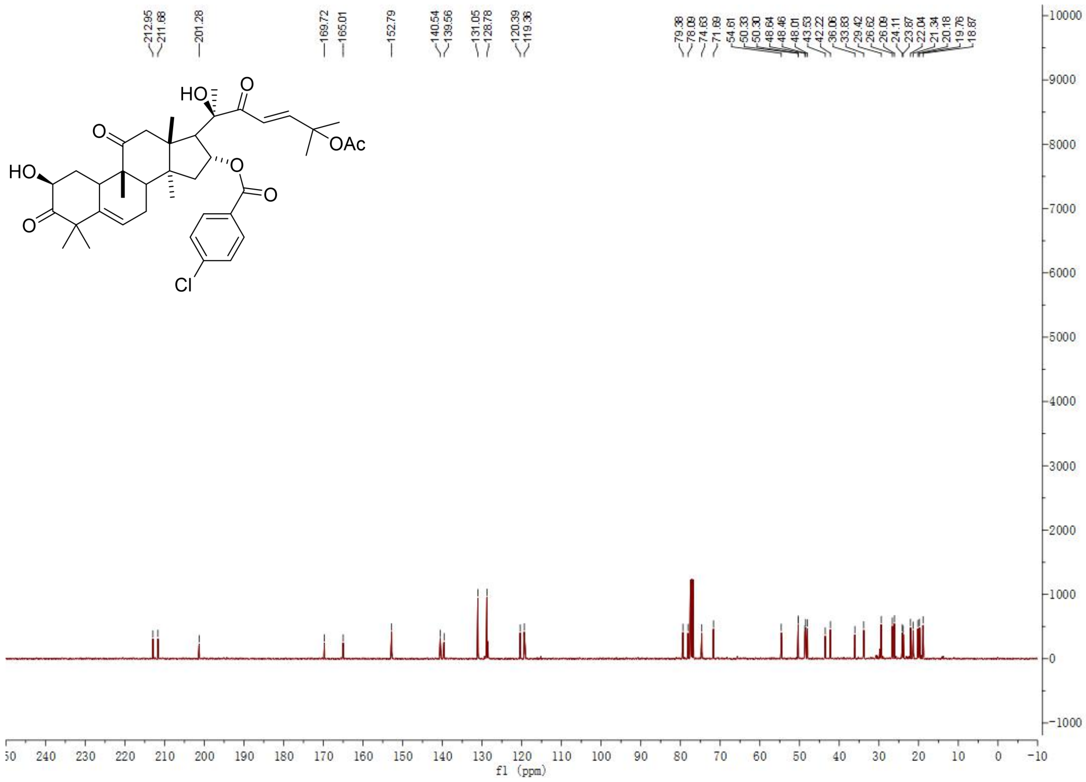

^{13}C NMR of compound **3j**

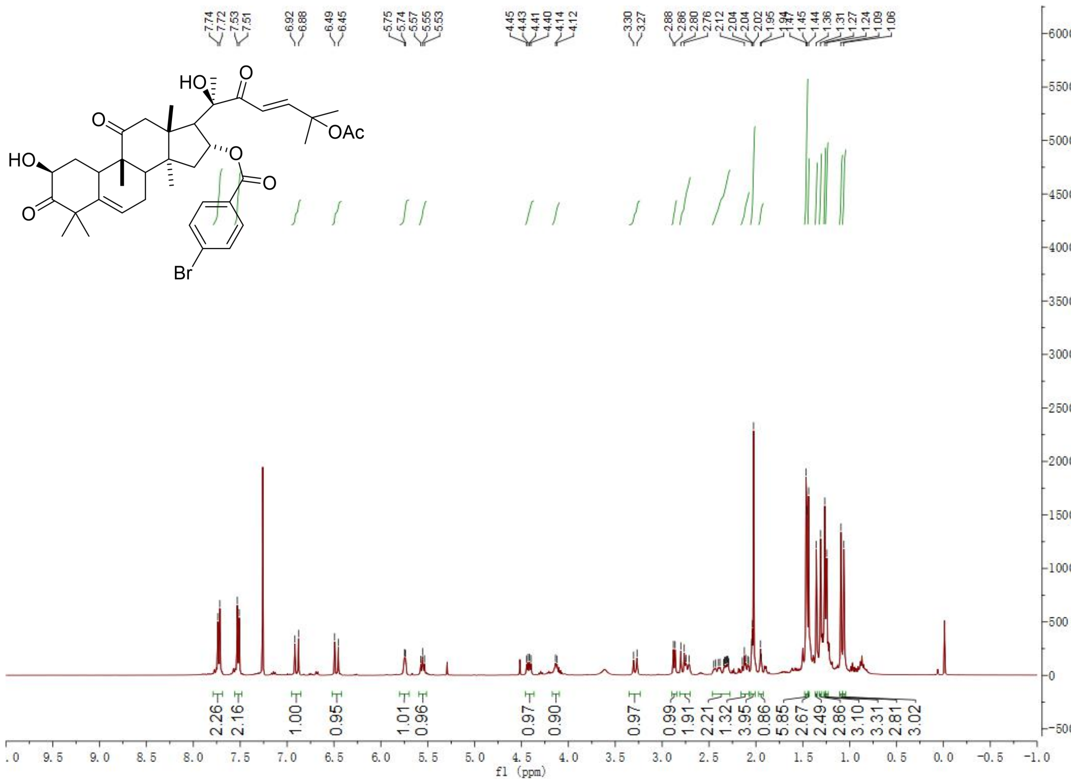

^1H NMR of compound **3k**

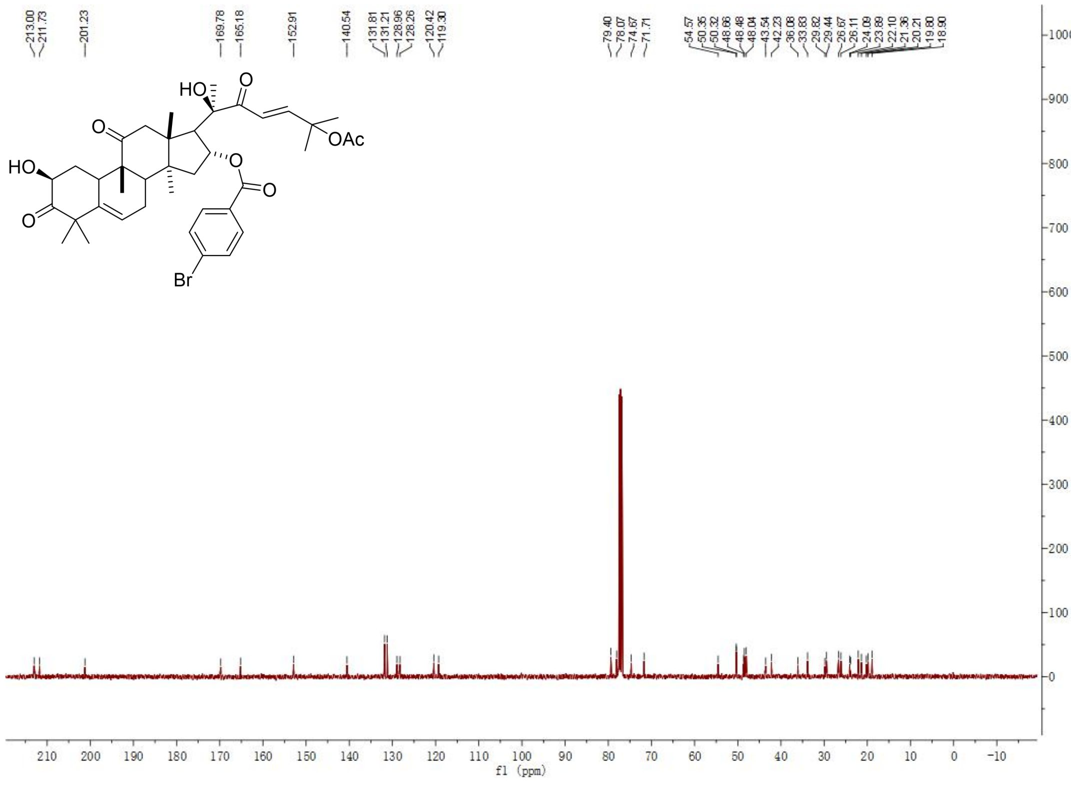

^{13}C NMR of compound **3k**

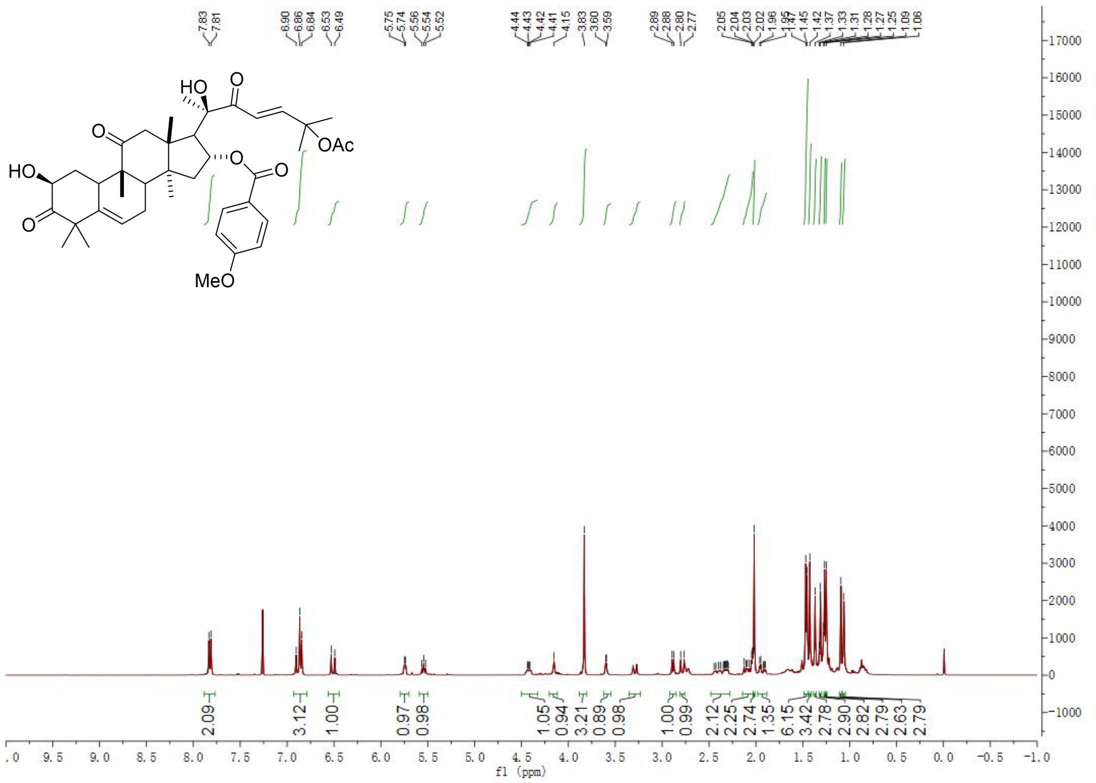

^{1}H NMR of compound **3l**

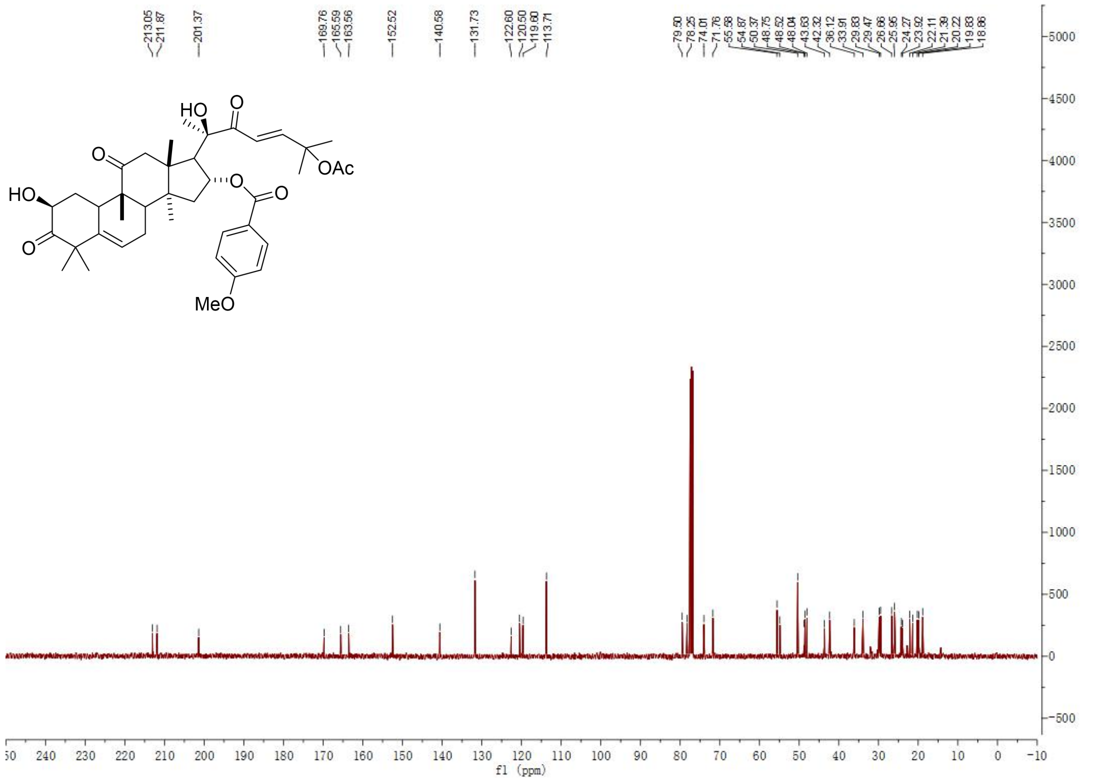

^{13}C NMR of compound **3l**

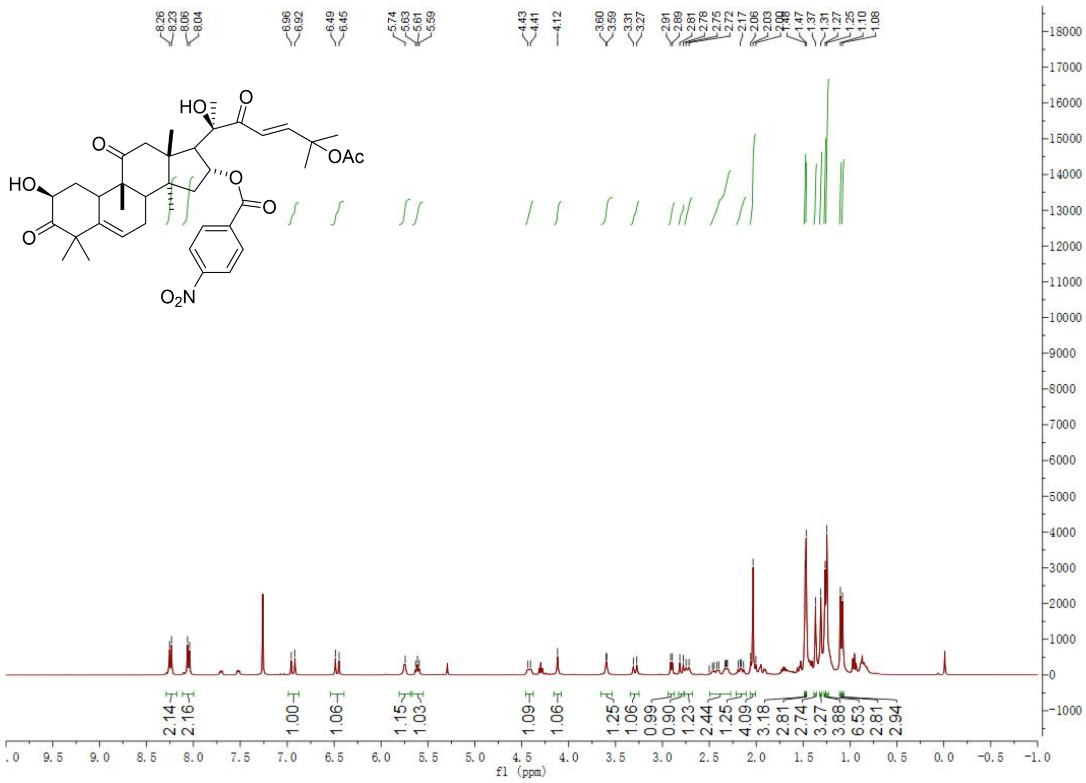

^{1}H NMR of compound **3m**

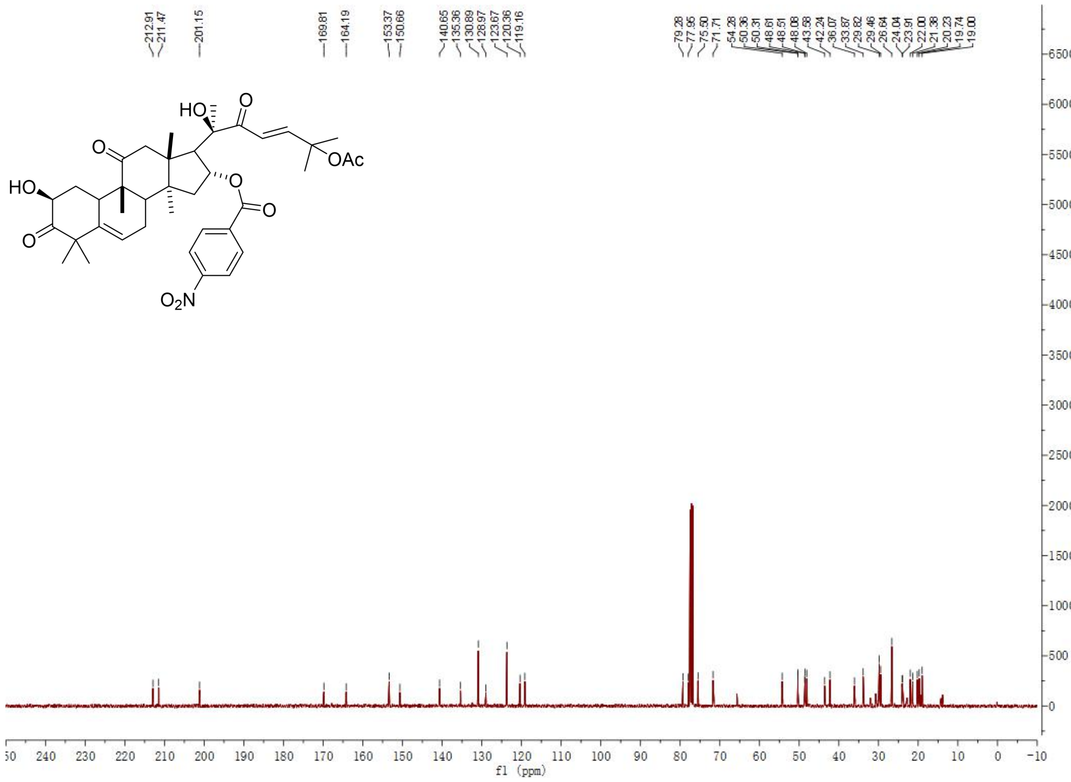

^{13}C NMR of compound **3m**

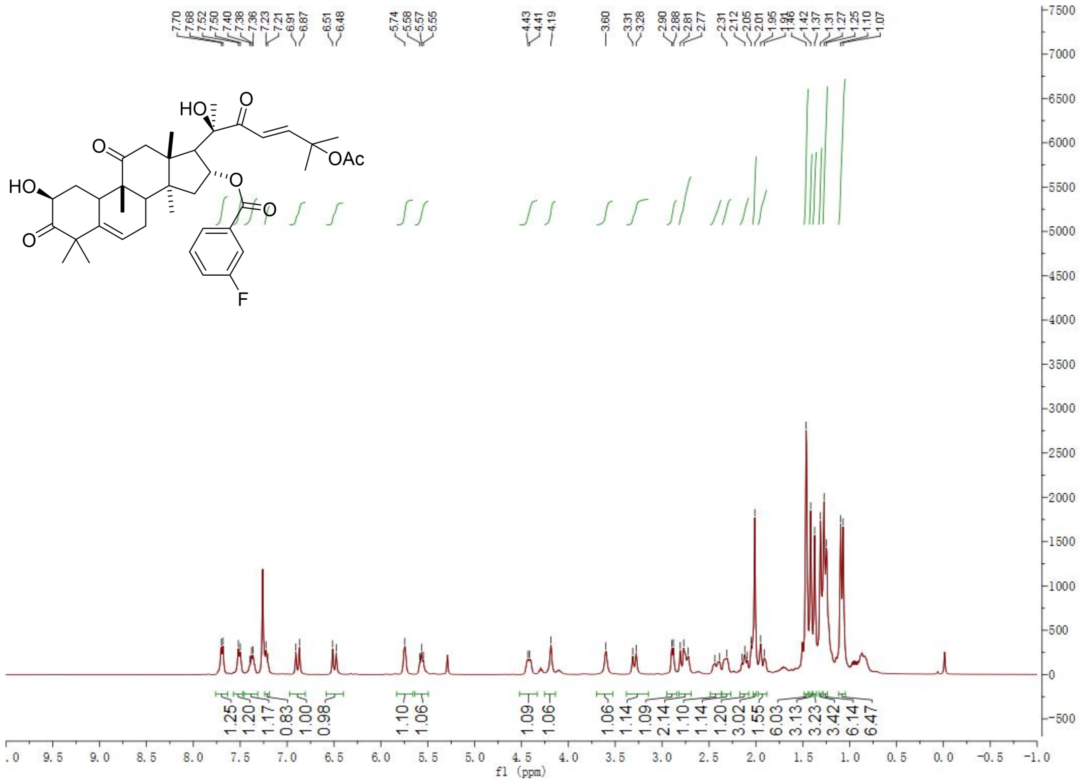

^{1}H NMR of compound **3n**

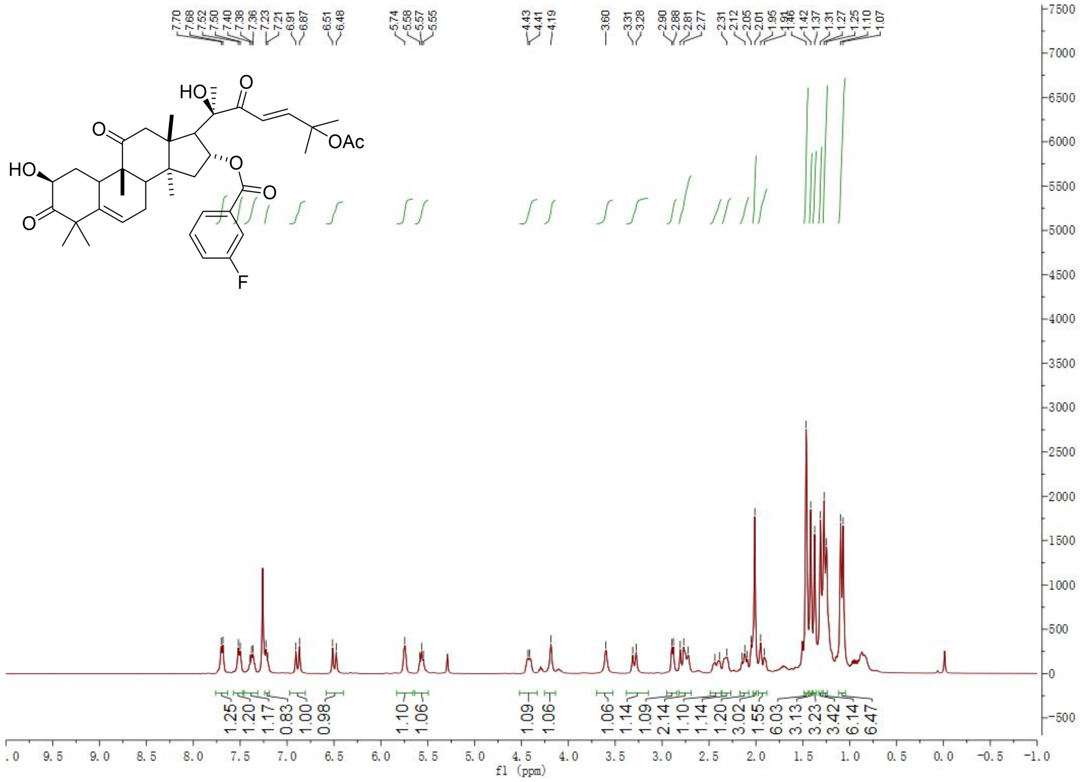

^{13}C NMR of compound **3n**

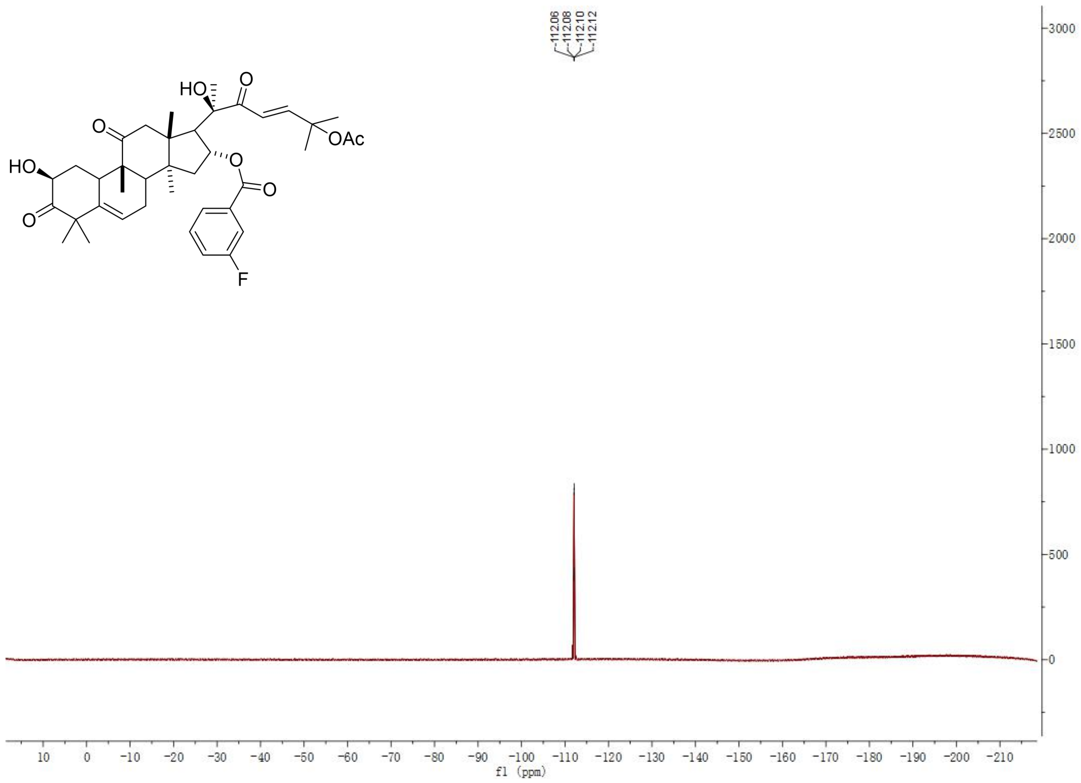

^{19}F NMR of compound **3n**

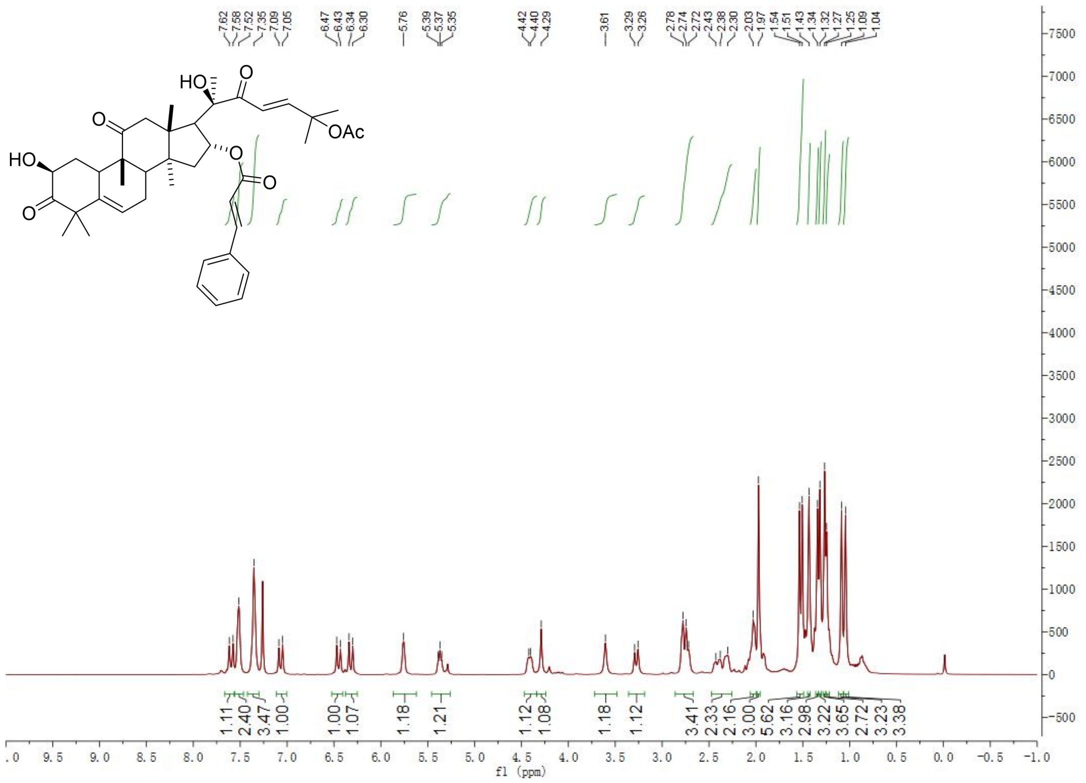

^{1}H NMR of compound **3o**

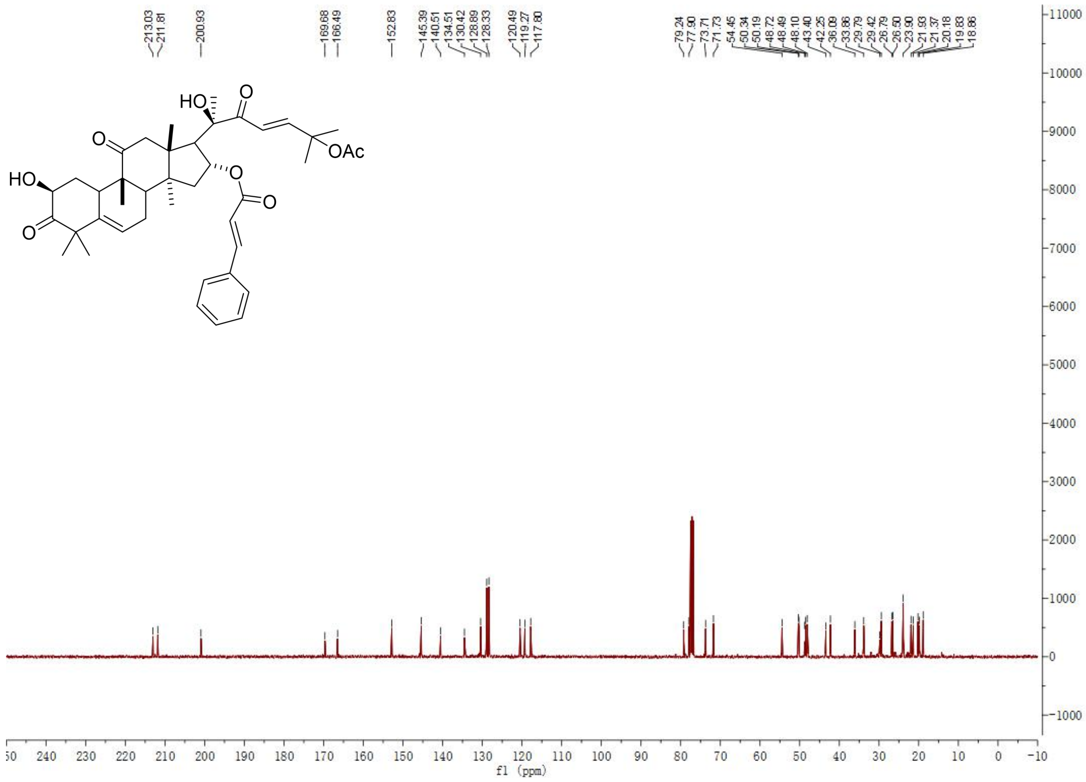

^{13}C NMR of compound **3o**

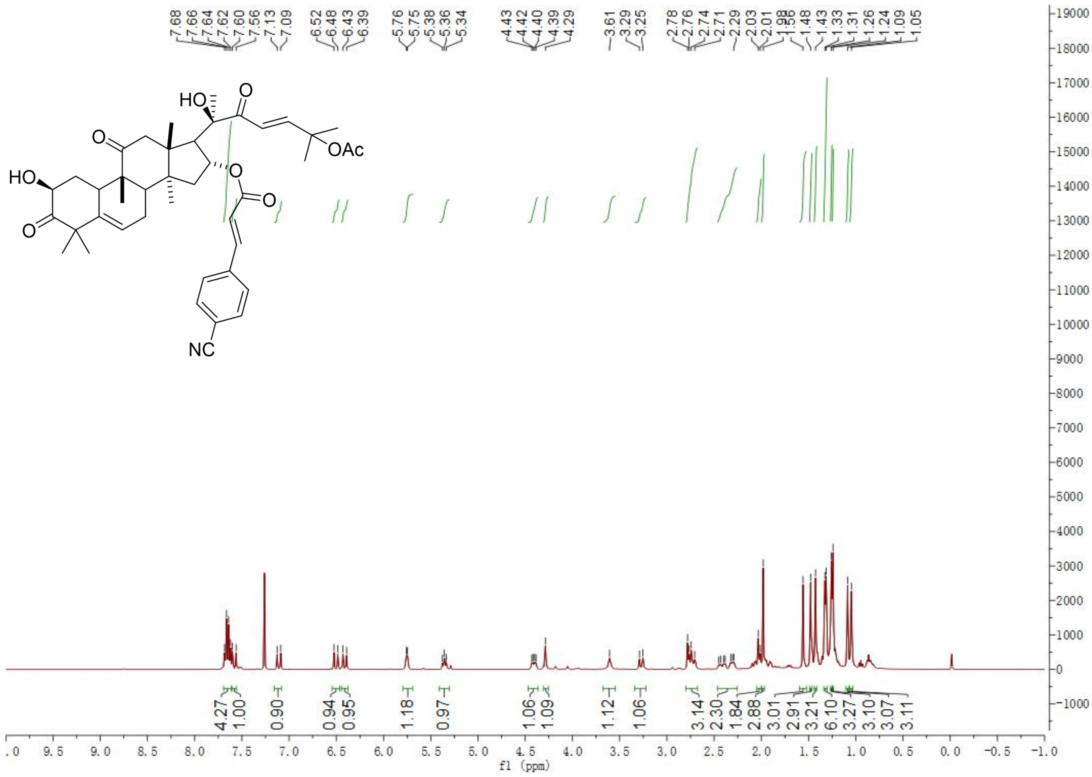

^{1}H NMR of compound **3p**

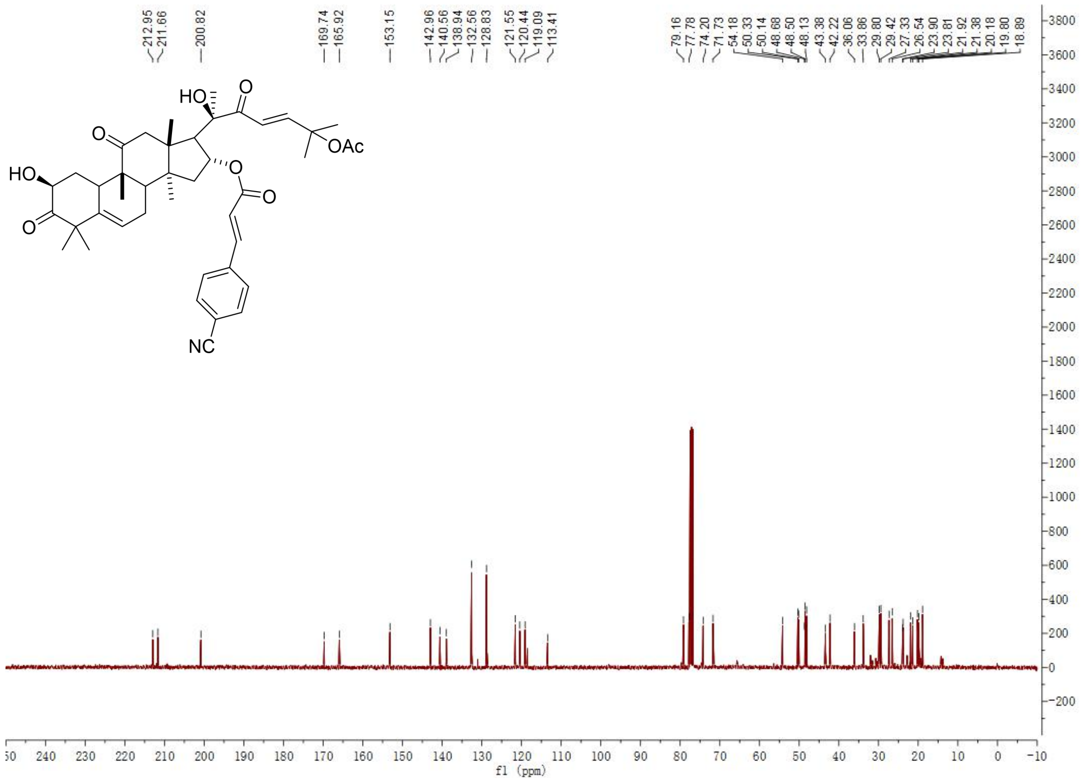

^{13}C NMR of compound **3p**

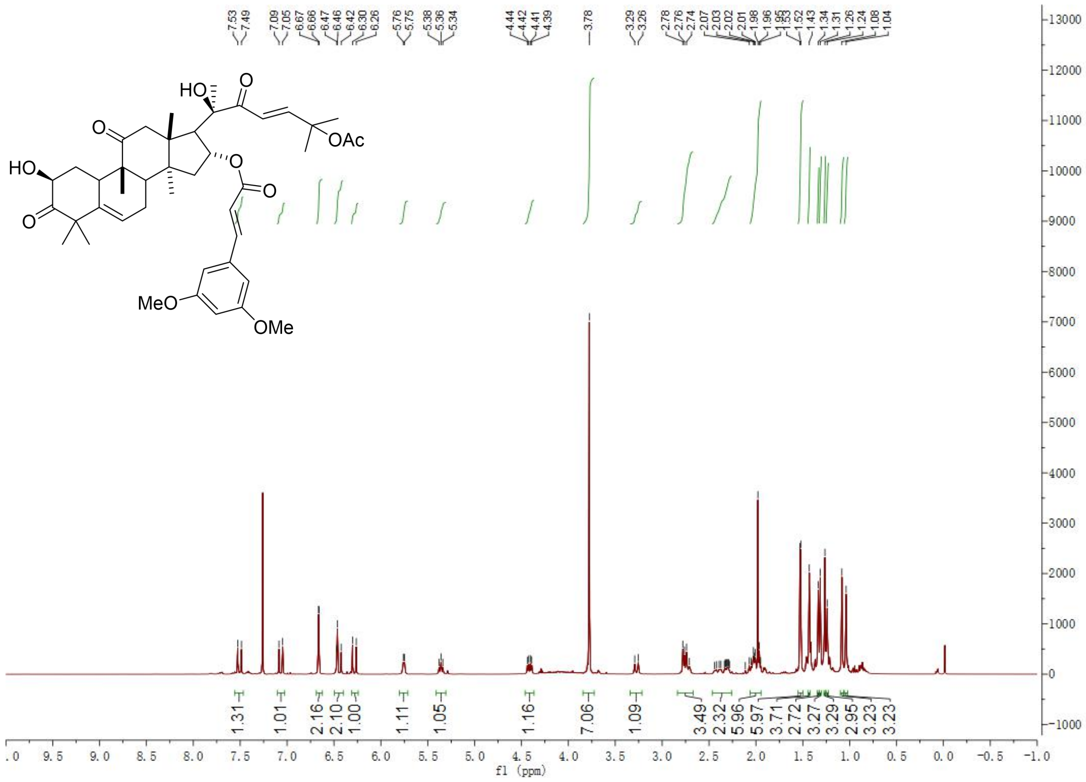

^{1}H NMR of compound **3q**

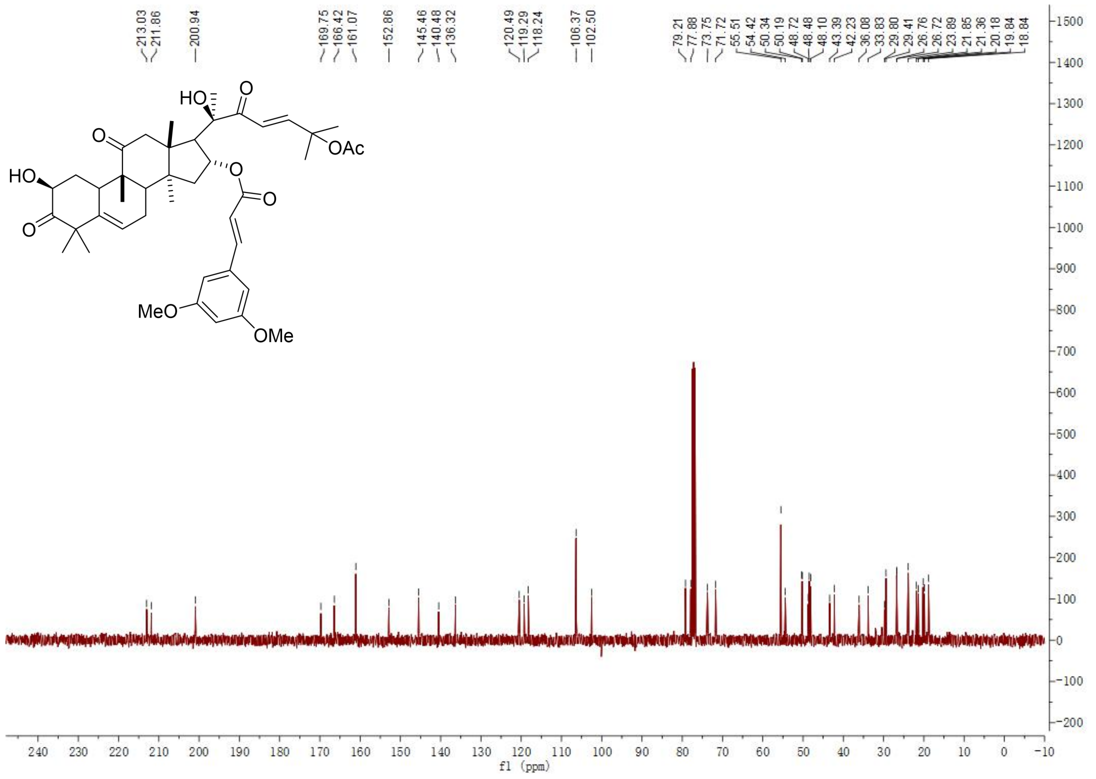

^{13}C NMR of compound **3q**

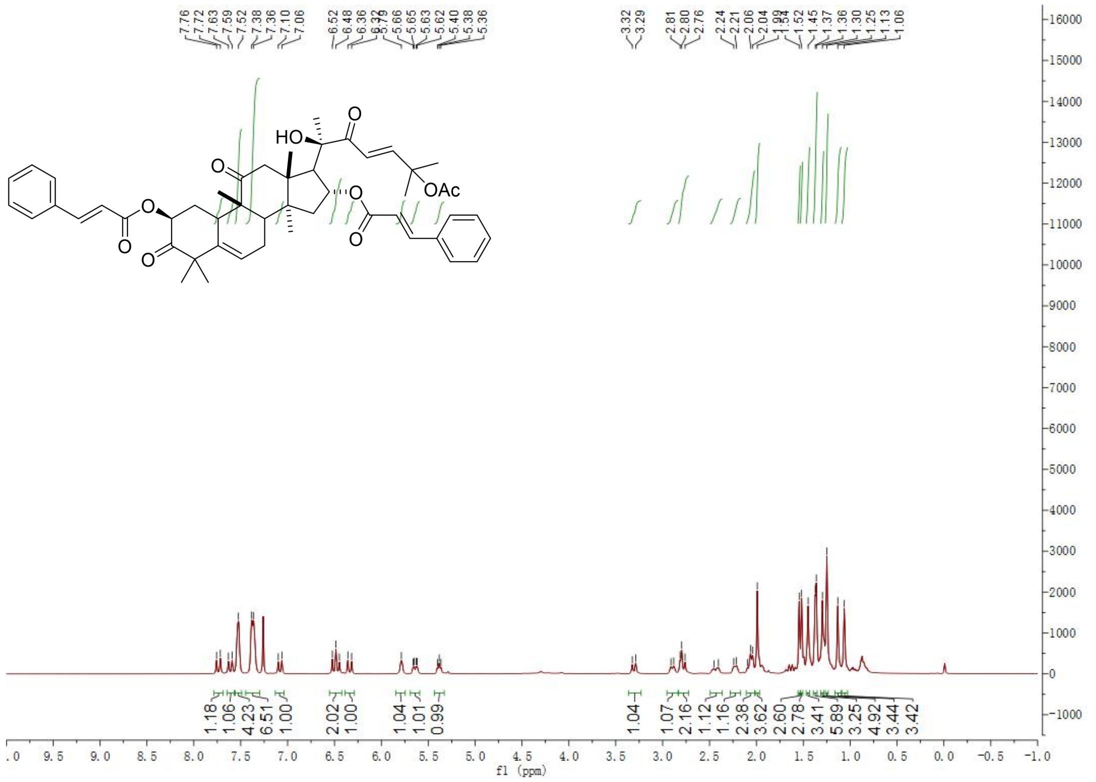

^{1}H NMR of compound **4**

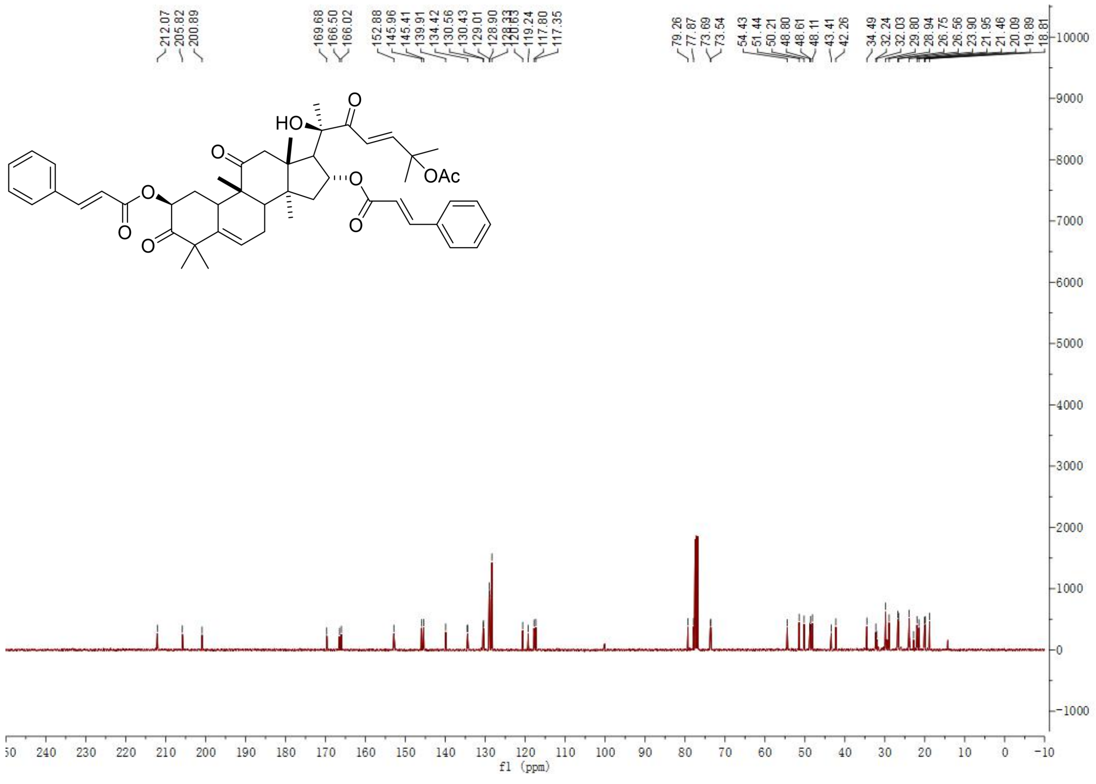

^{13}C NMR of compound **3q**

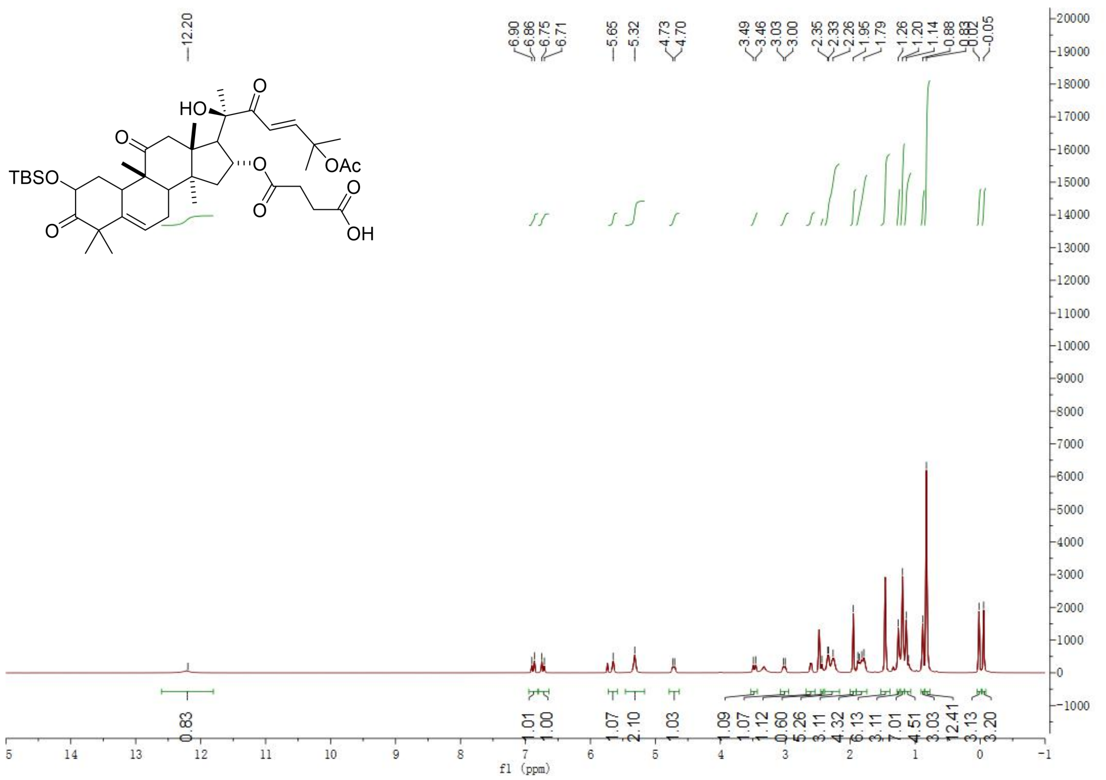

^{1}H NMR of compound **8**

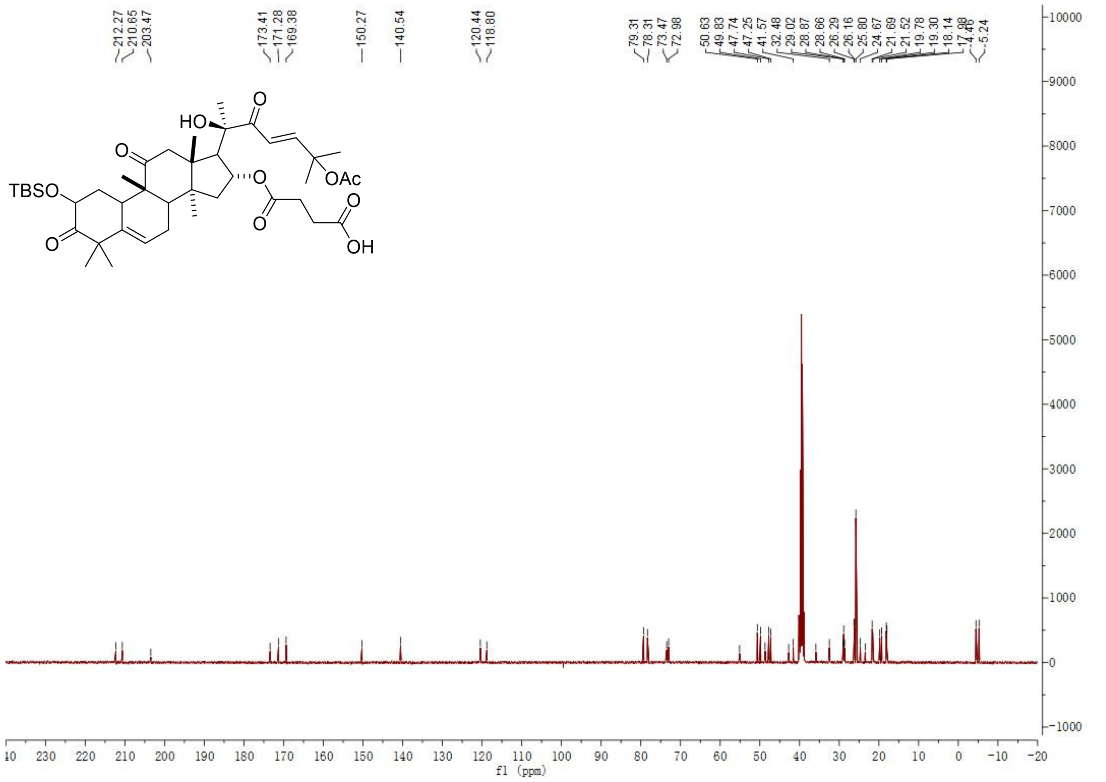

^{13}C NMR of compound **8**

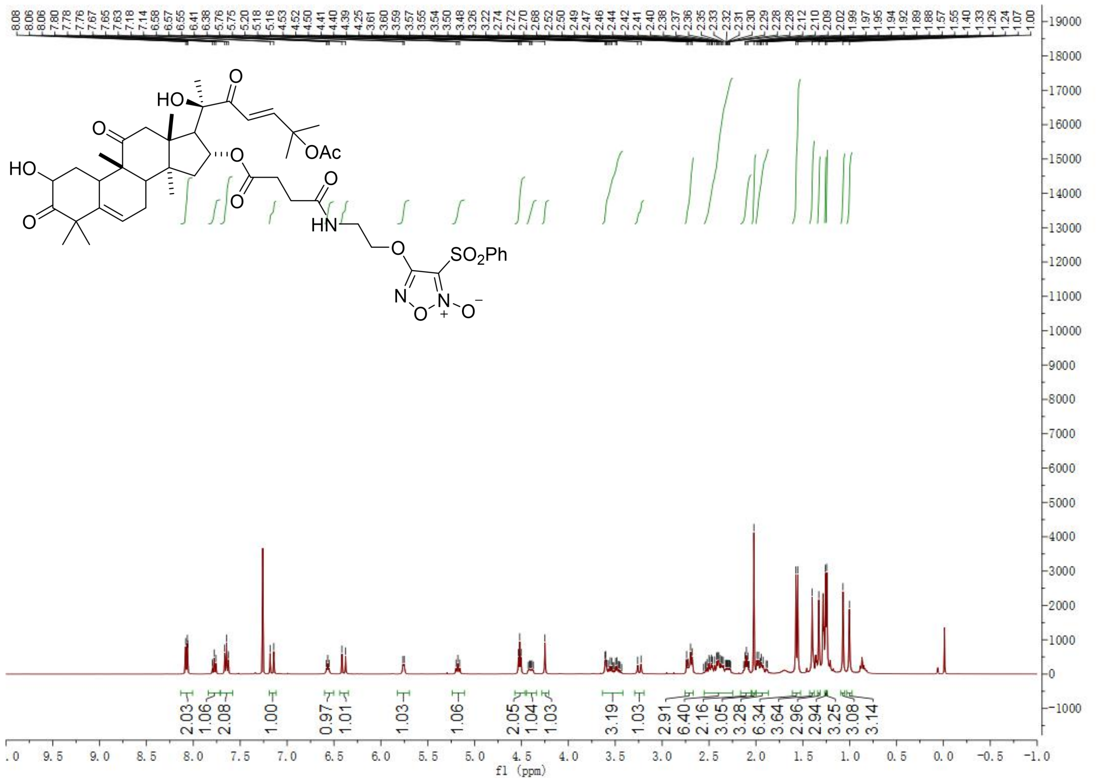

^{1}H NMR of compound **9a**

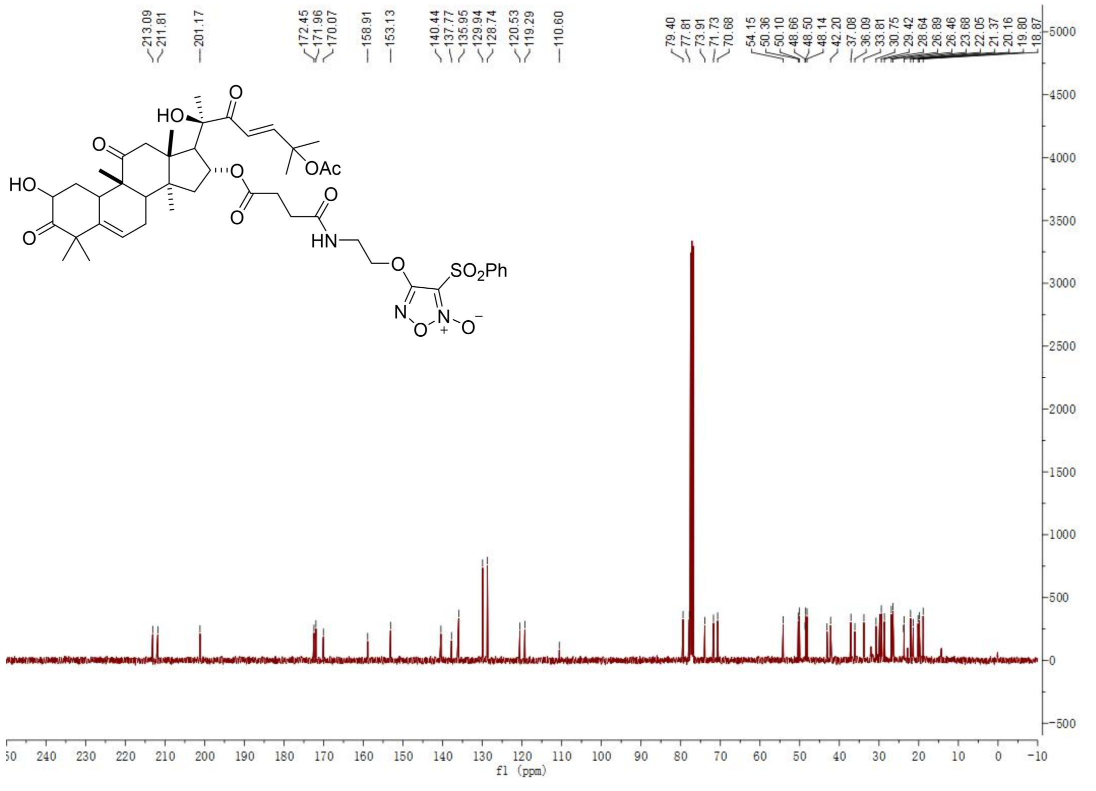

^{13}C NMR of compound **9a**

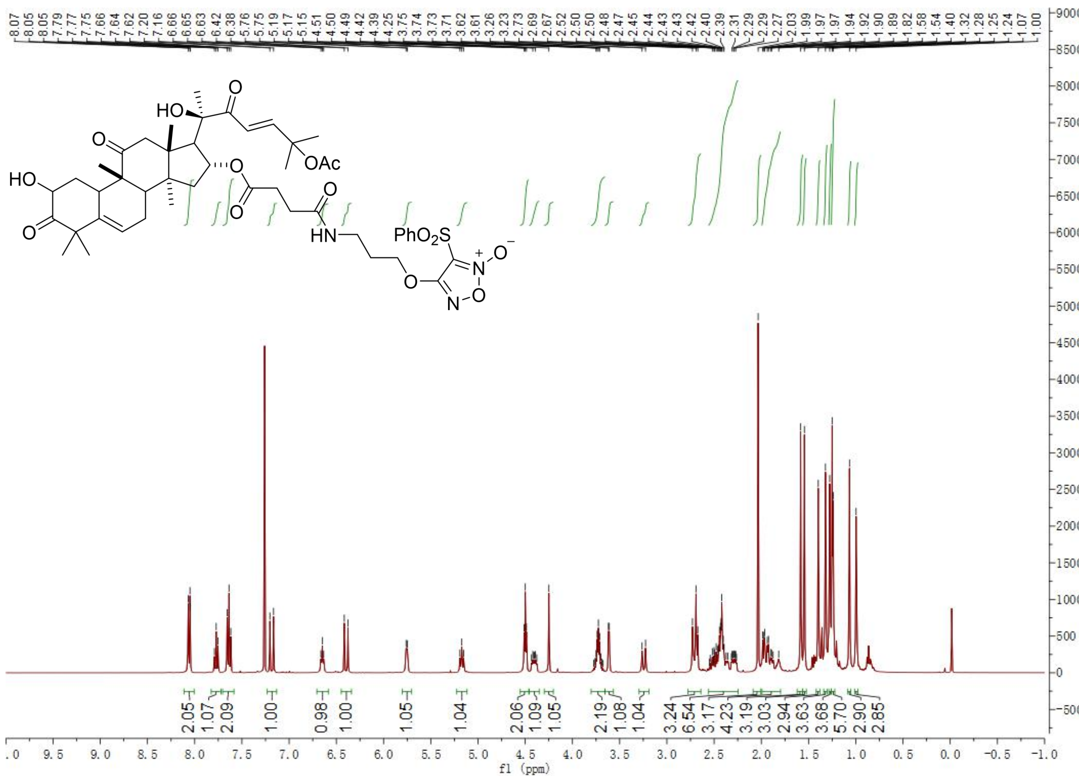

^{1}H NMR of compound **9b**

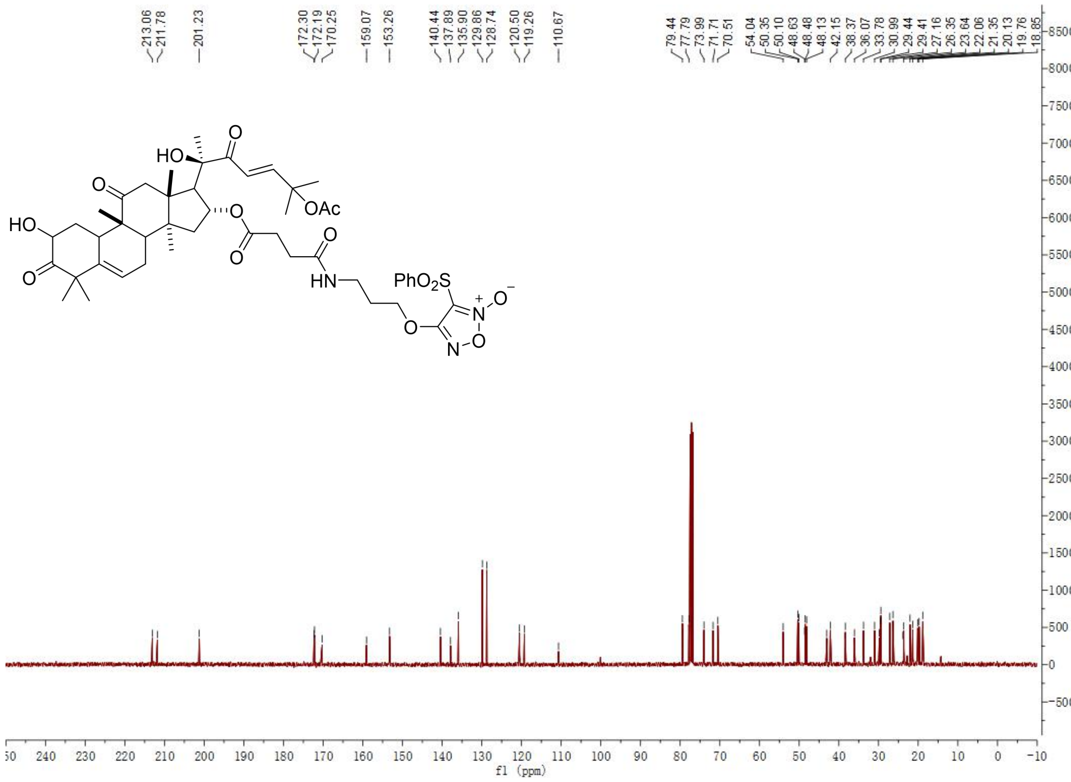

^{13}C NMR of compound **9b**

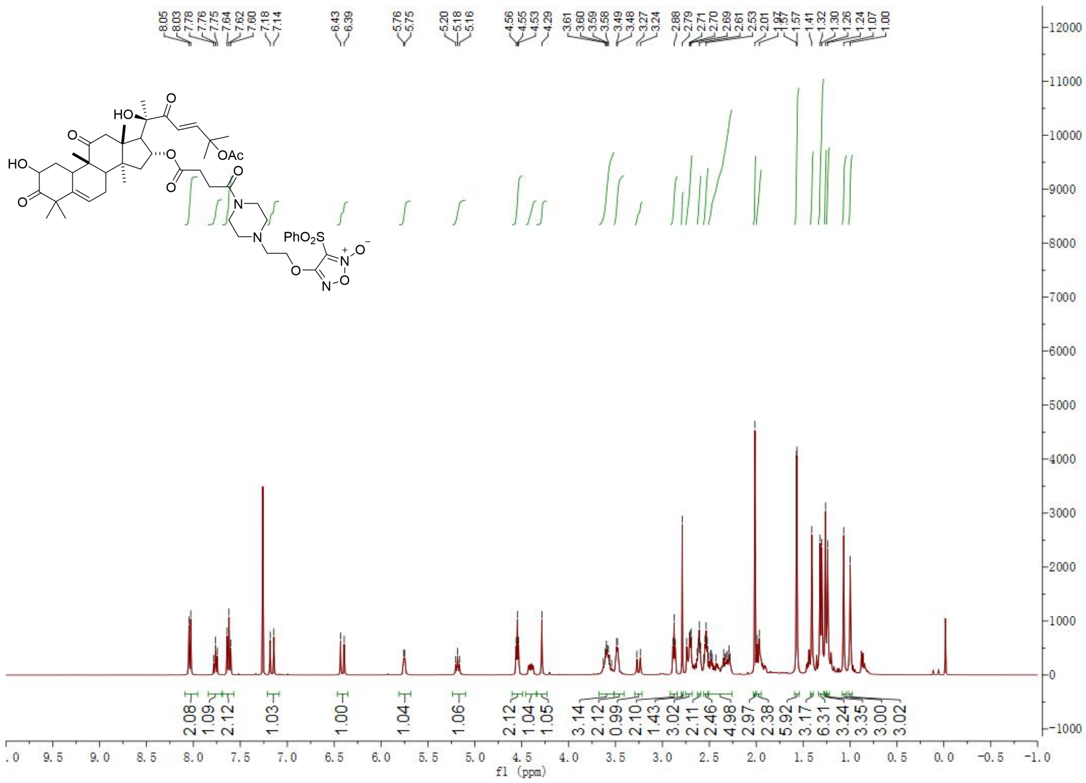

^1H NMR of compound **9c**

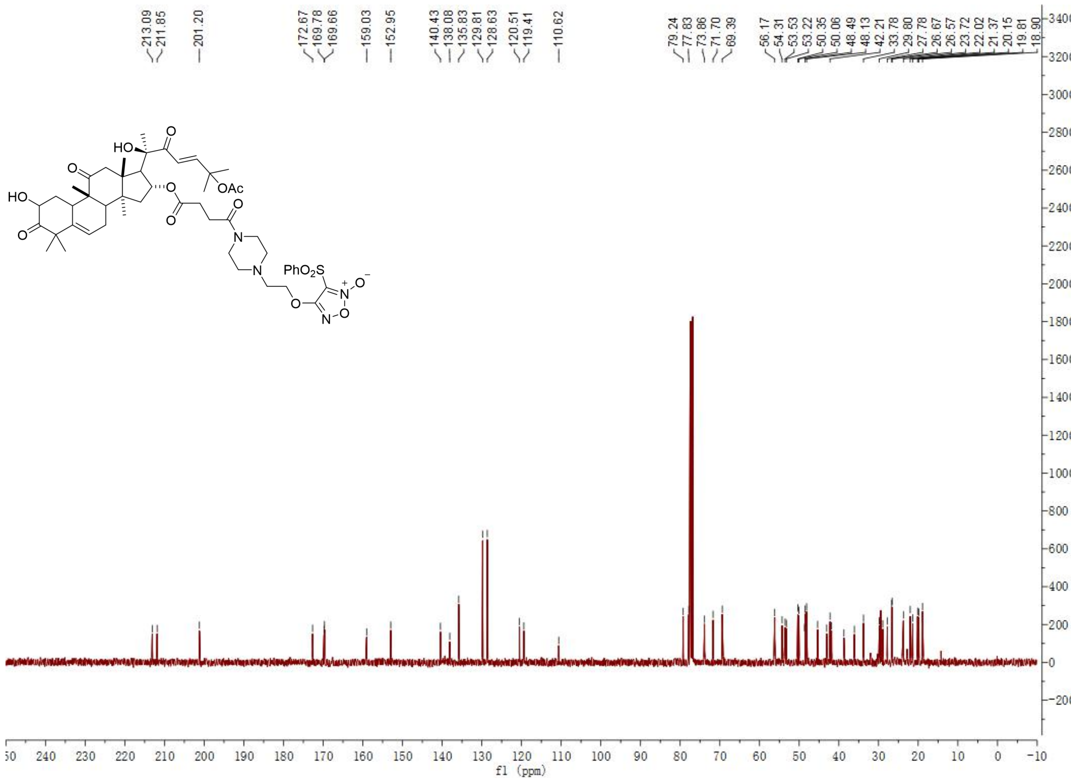

^{13}C NMR of compound **9c**

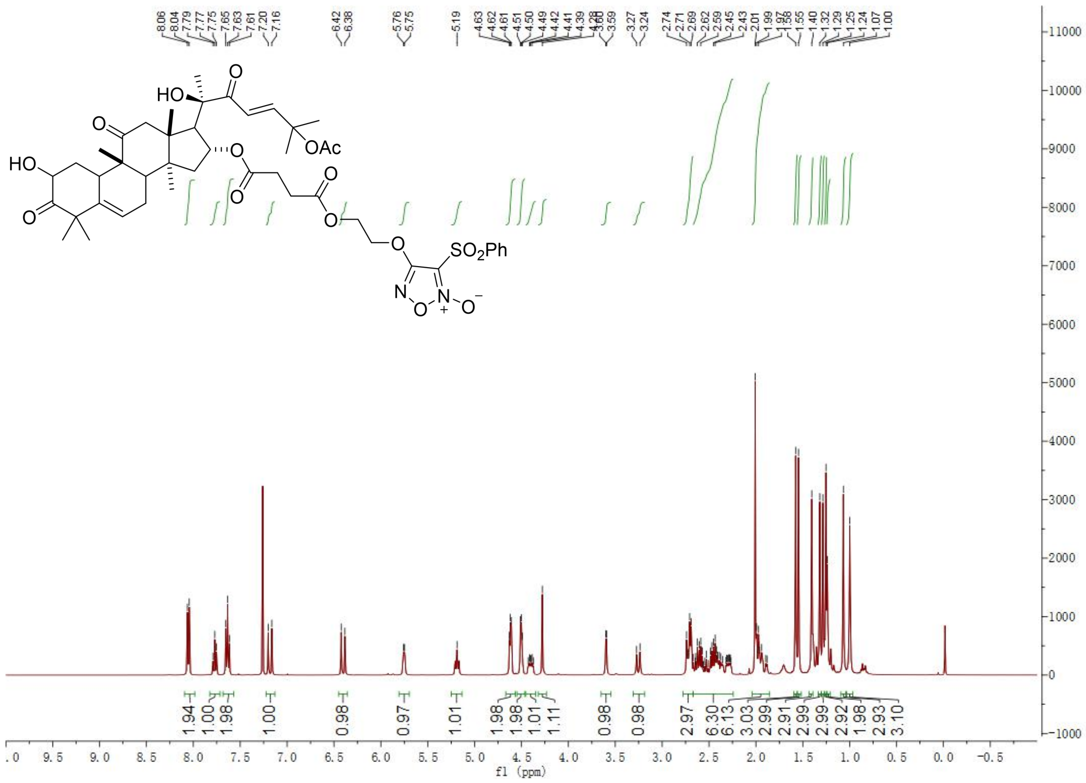

^{1}H NMR of compound **10a**

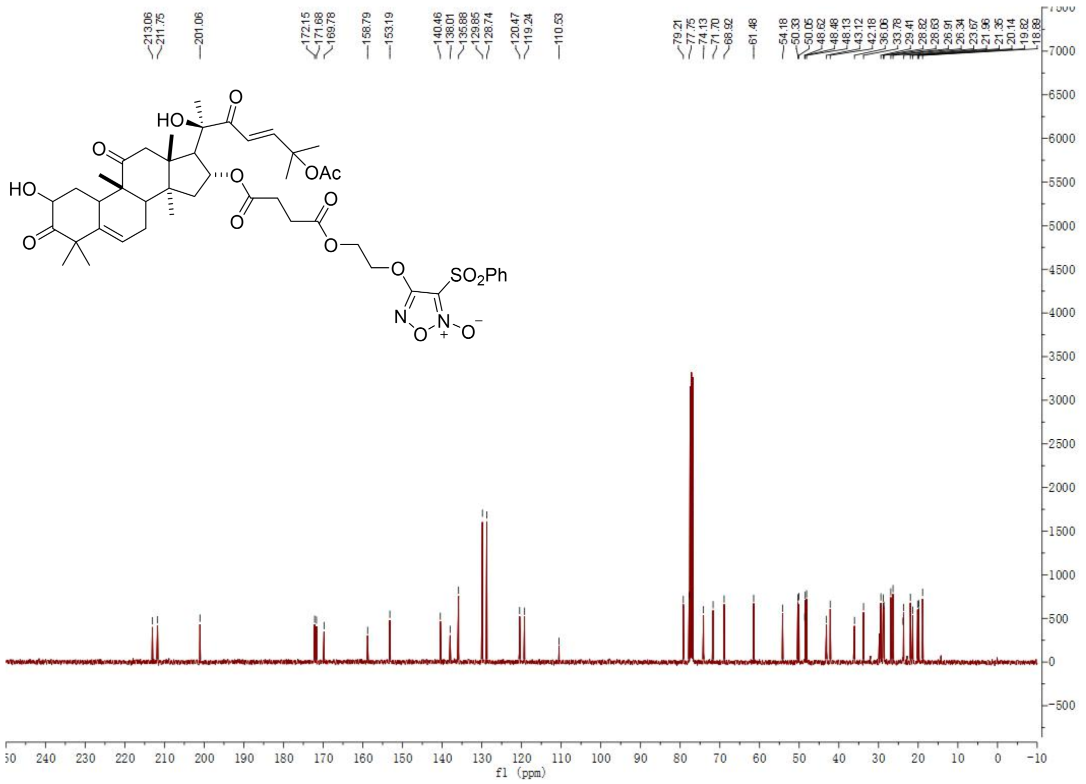

^{13}C NMR of compound **10a**

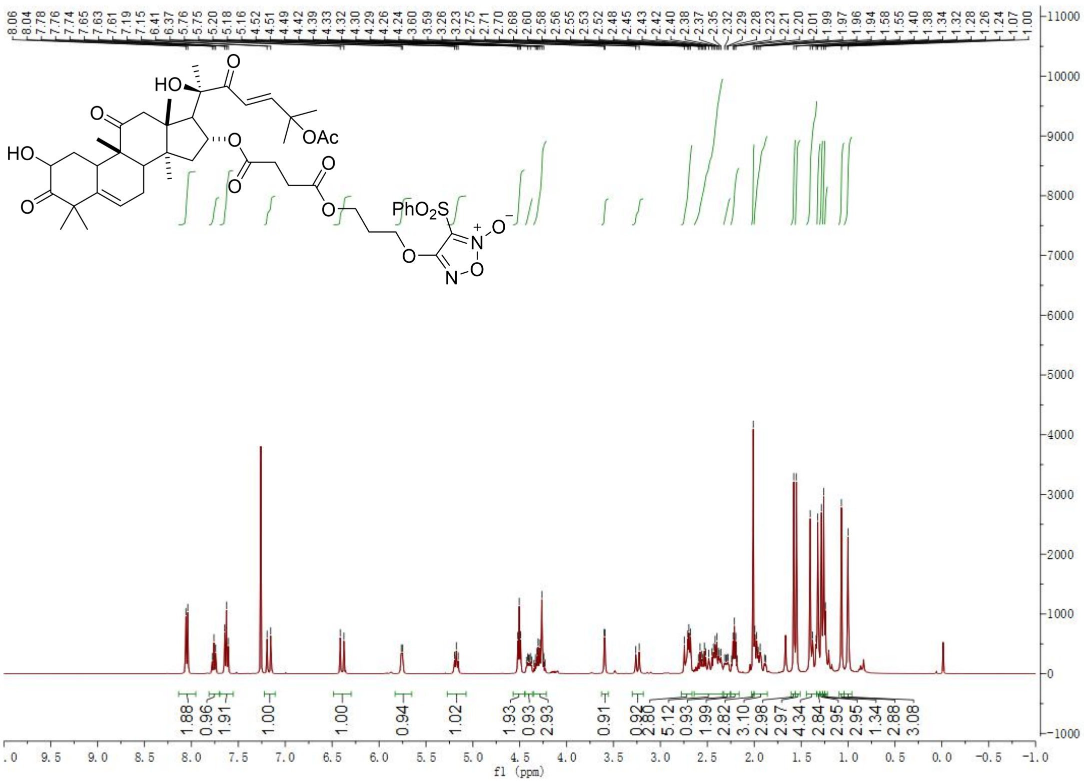

^{1}H NMR of compound **10b**

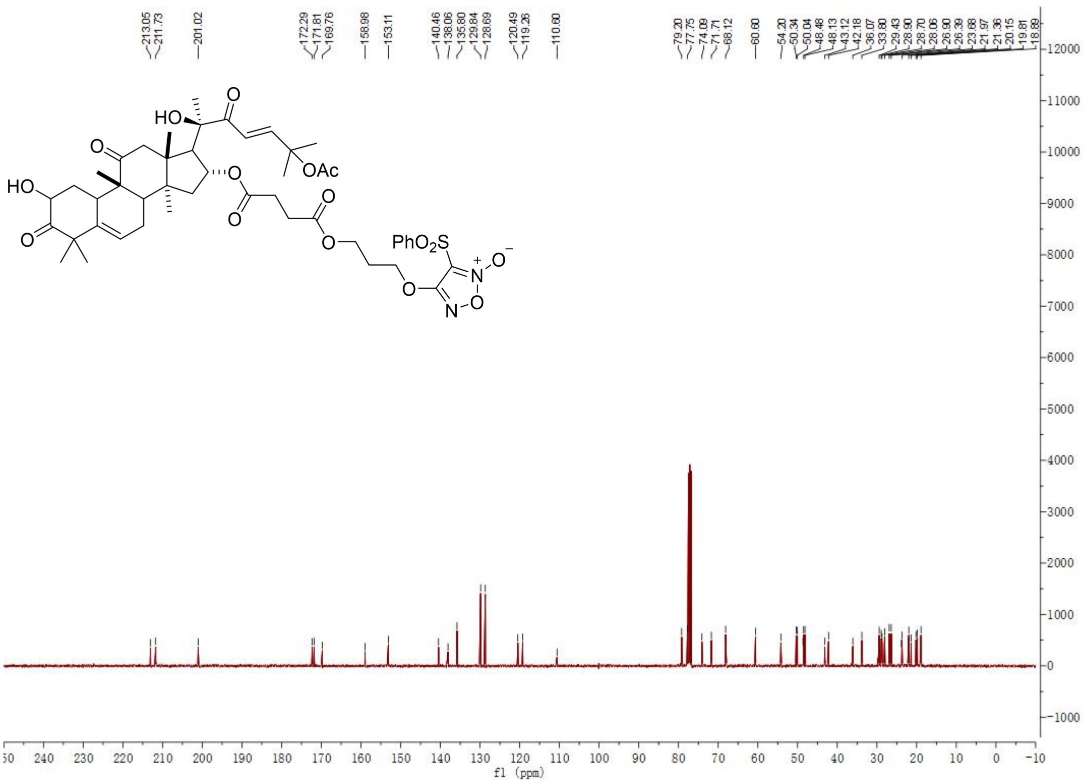

^{13}C NMR of compound **10b**

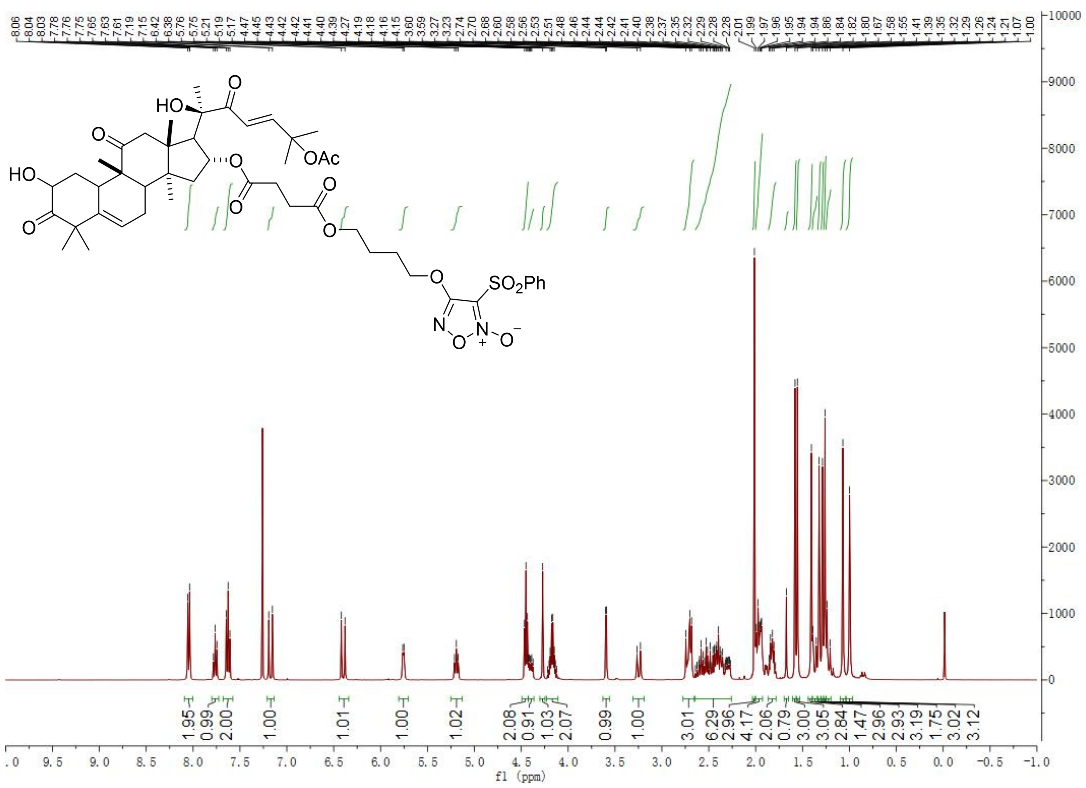

^{1}H NMR of compound **10c**

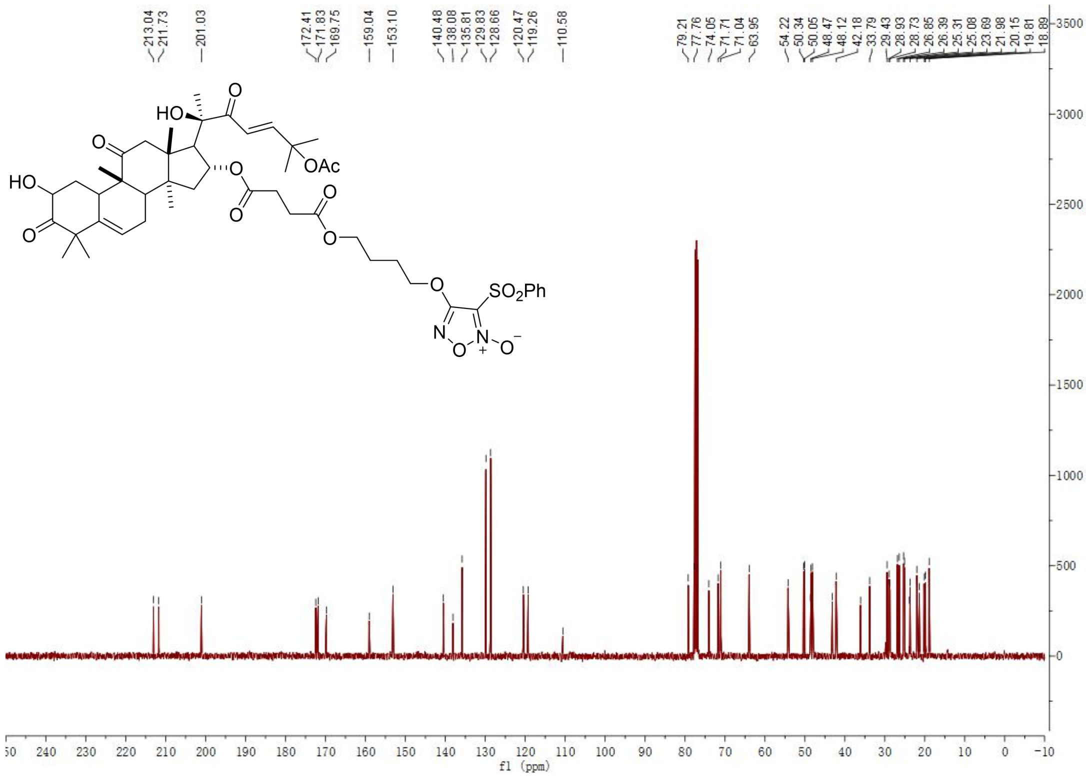

^{13}C NMR of compound **10c**

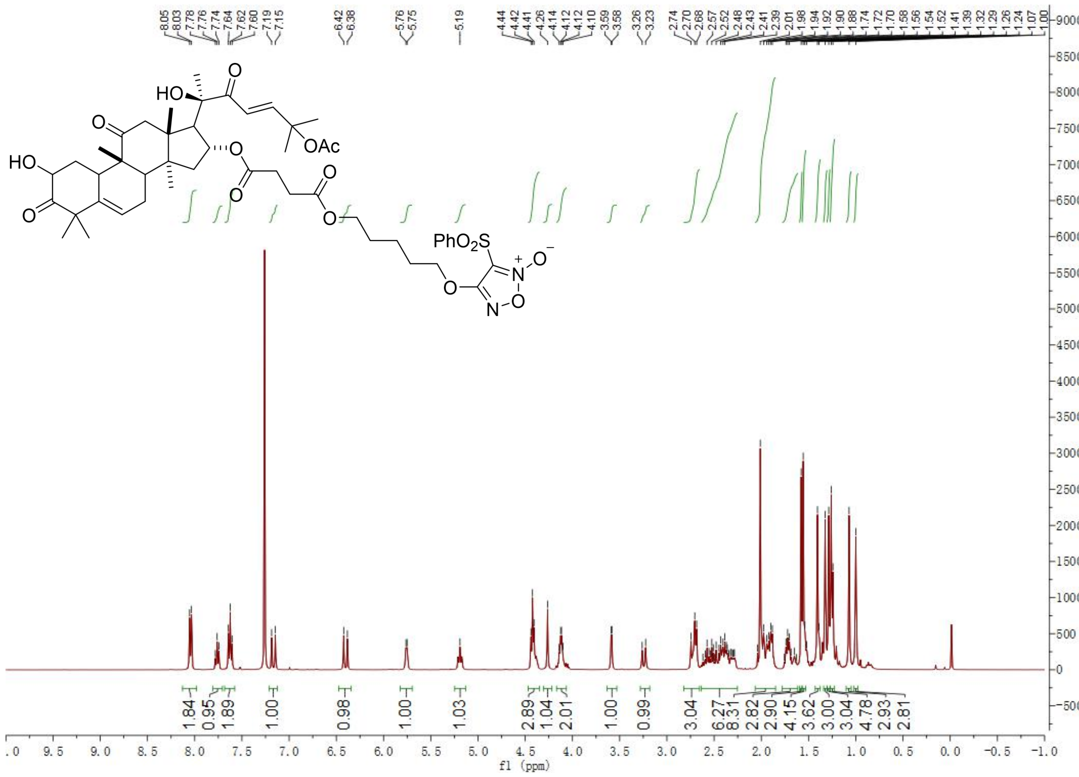

^1H NMR of compound **10d**

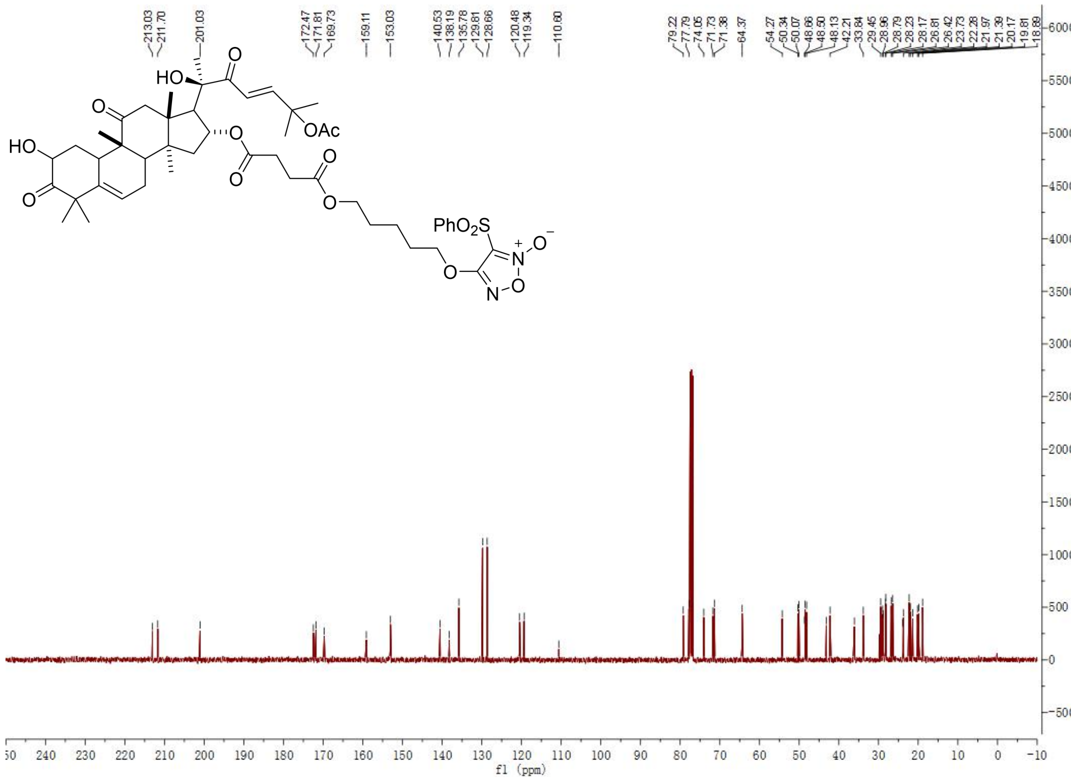

^{13}C NMR of compound **10d**

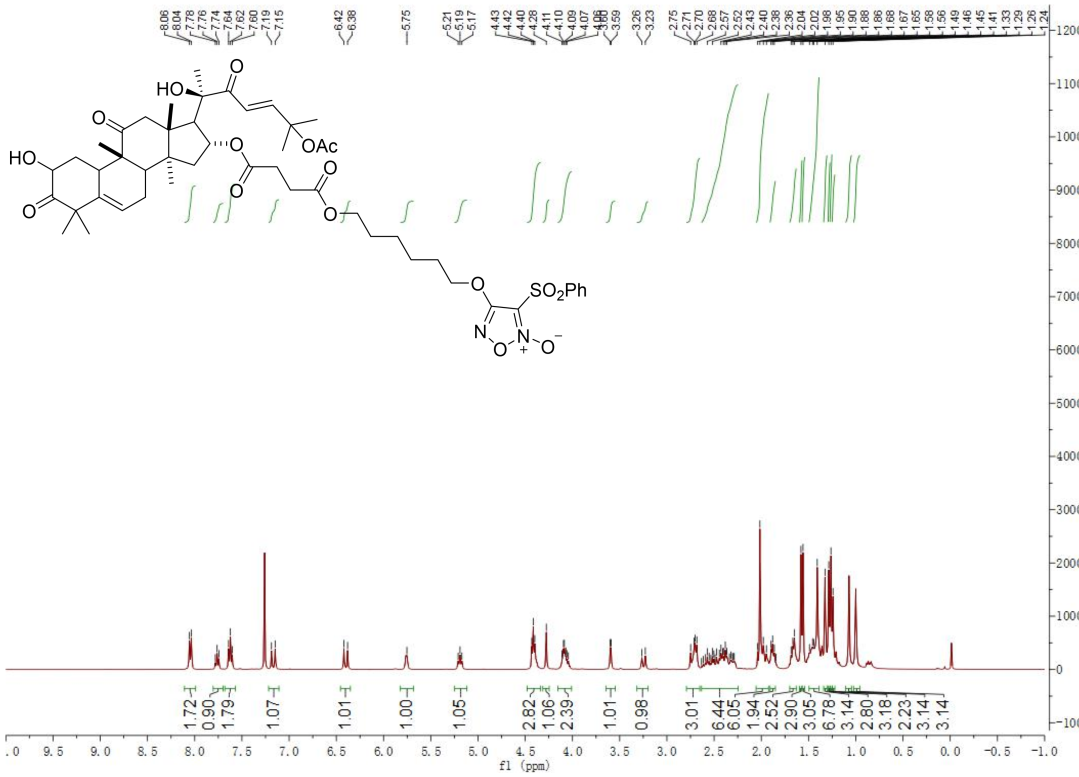

^{1}H NMR of compound **10e**

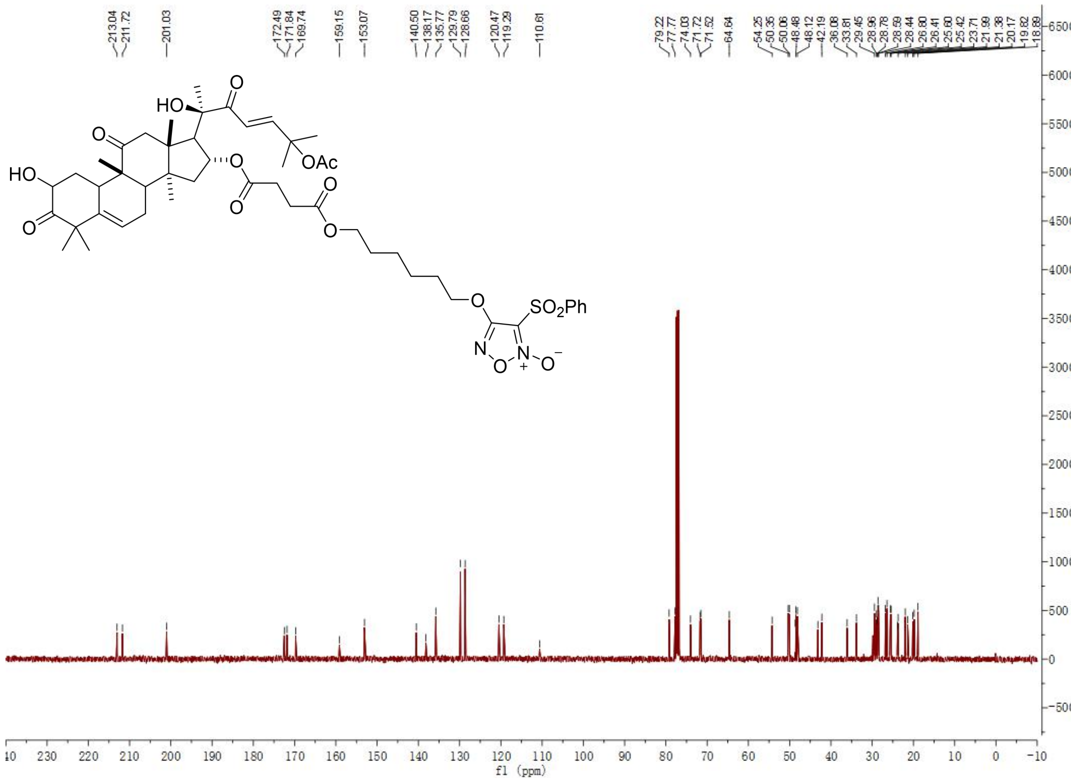

^{13}C NMR of compound **10e**

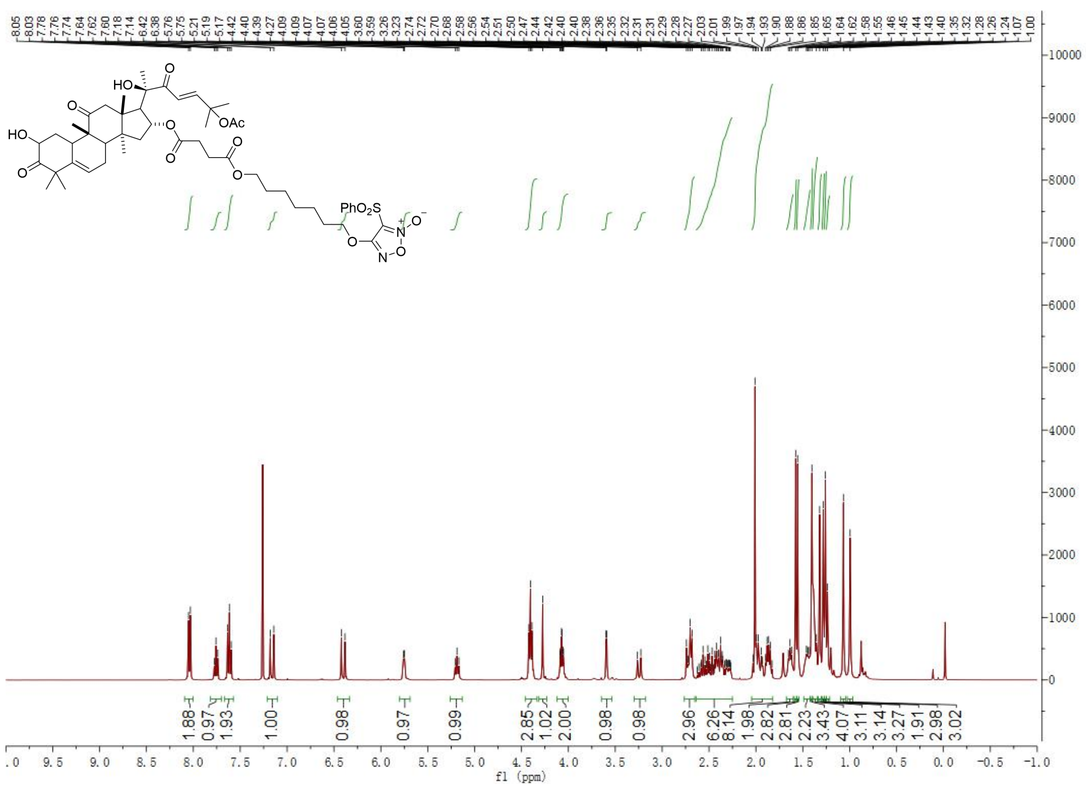

^{1}H NMR of compound **10f**

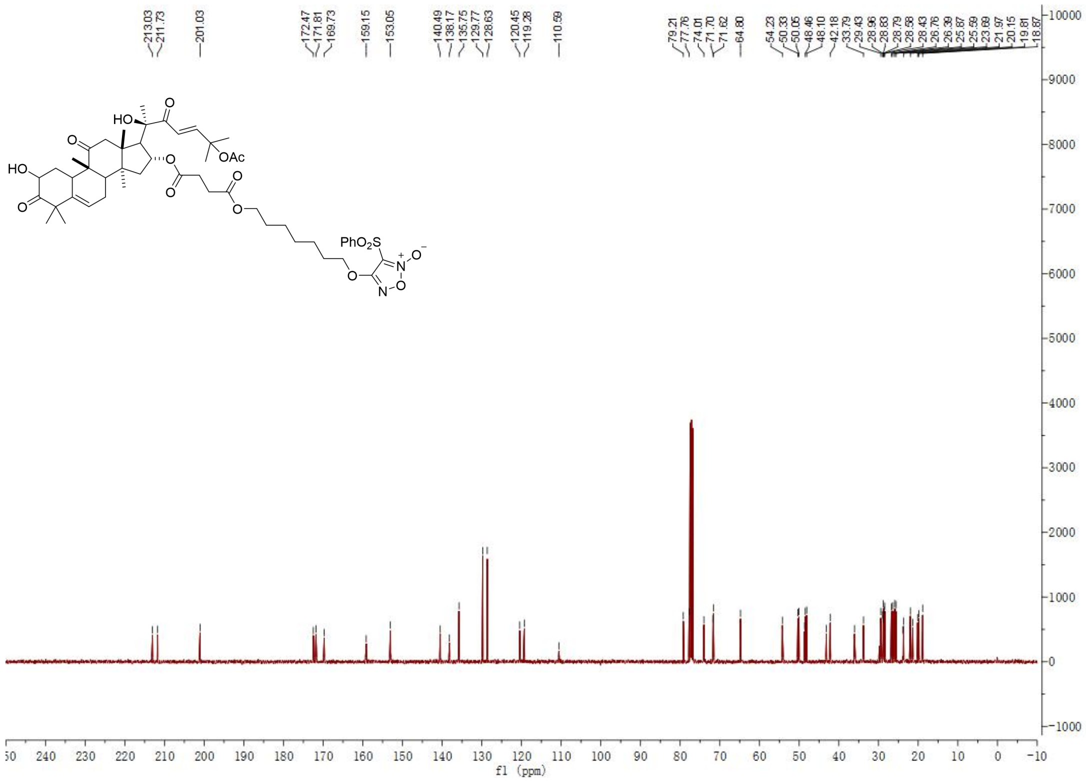

^{13}C NMR of compound **10f**

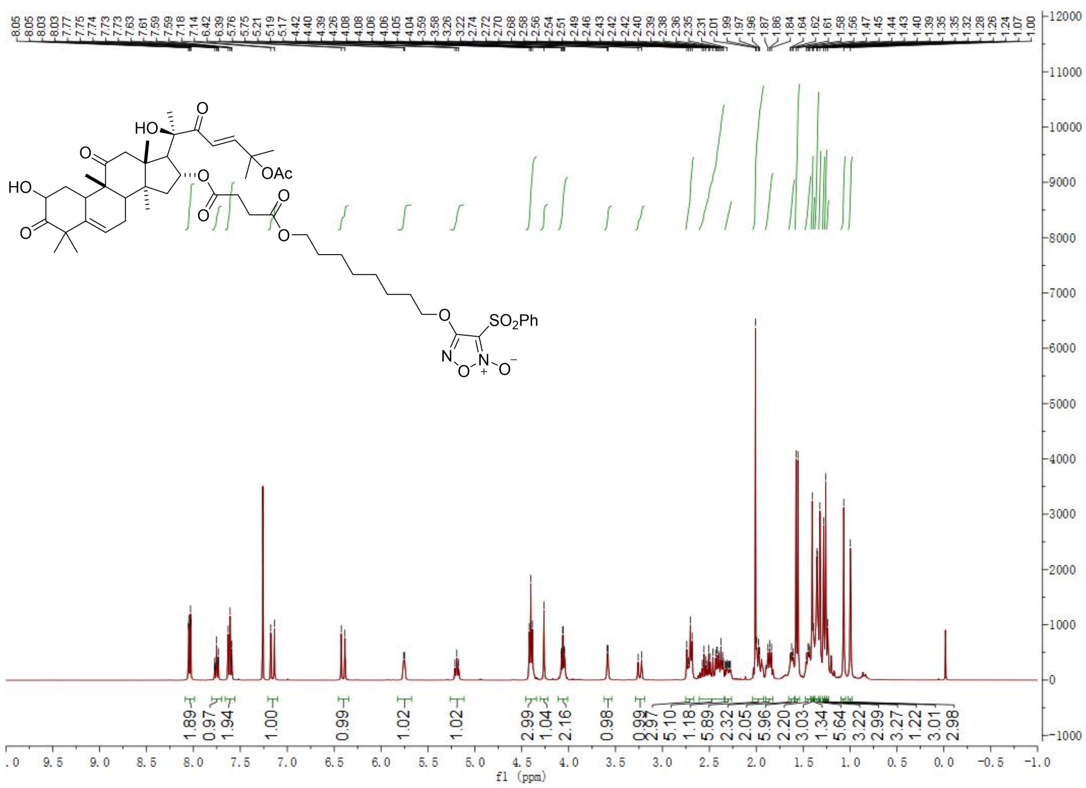

^{1}H NMR of compound **10g**

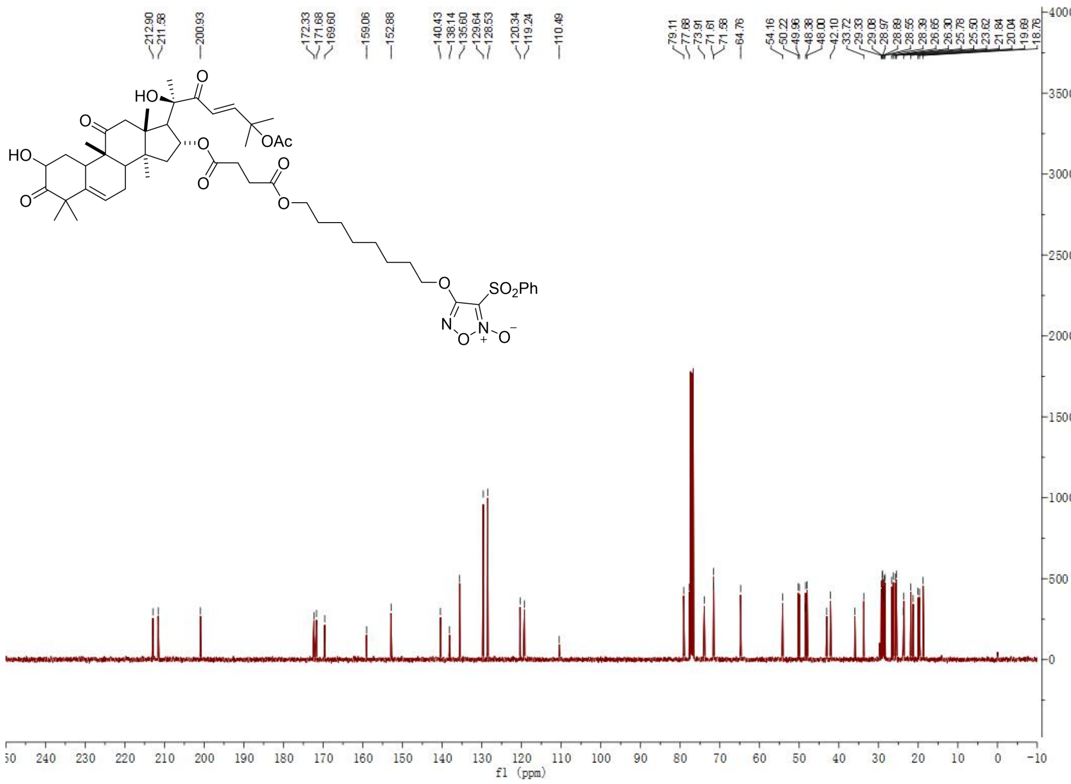

^{13}C NMR of compound **10g**

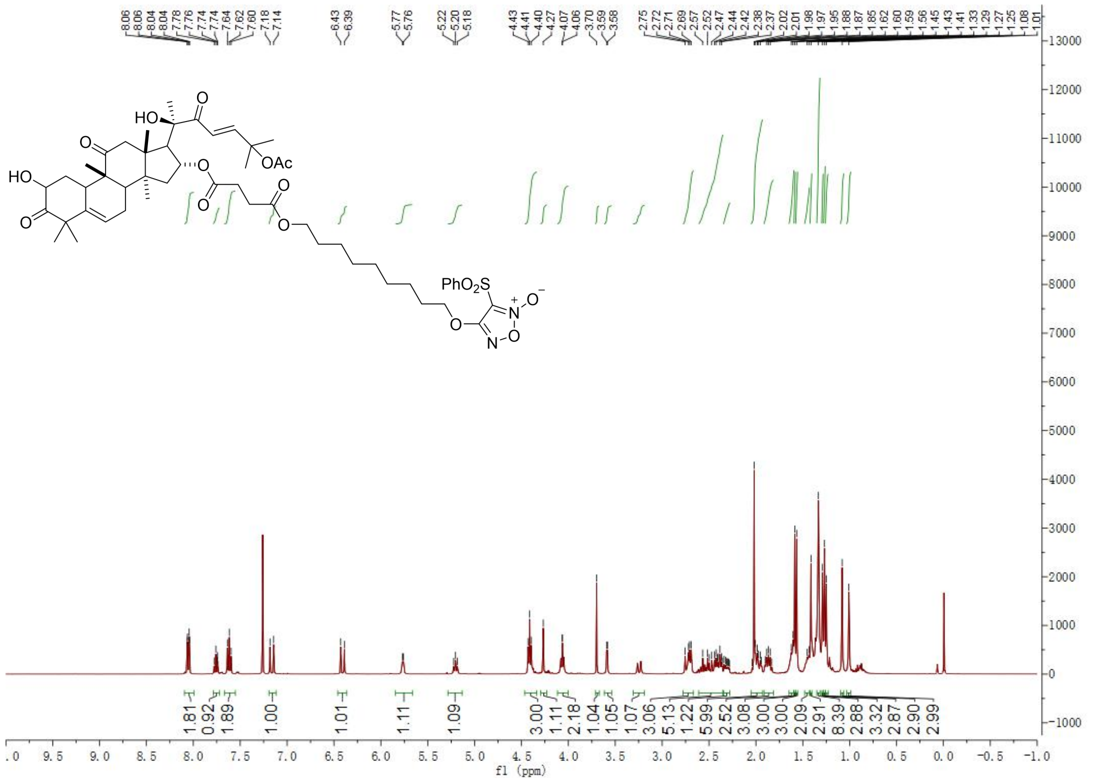

^{1}H NMR of compound **10h**

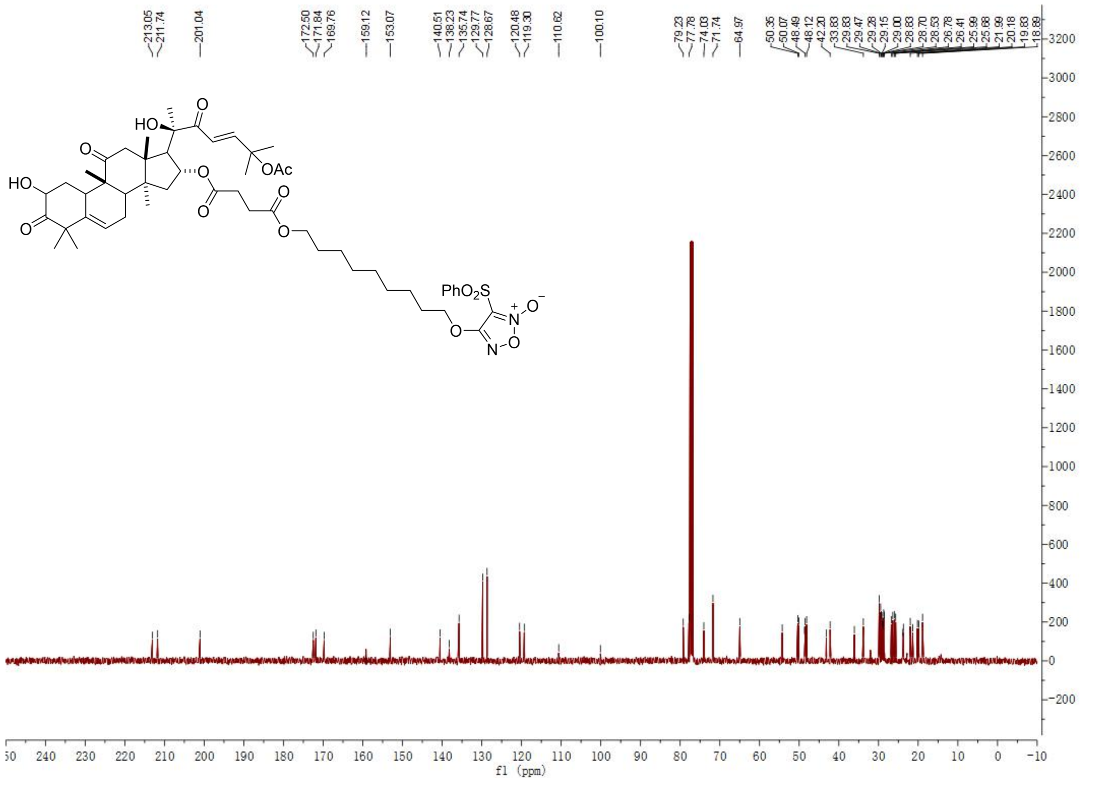

^{13}C NMR of compound **10h**

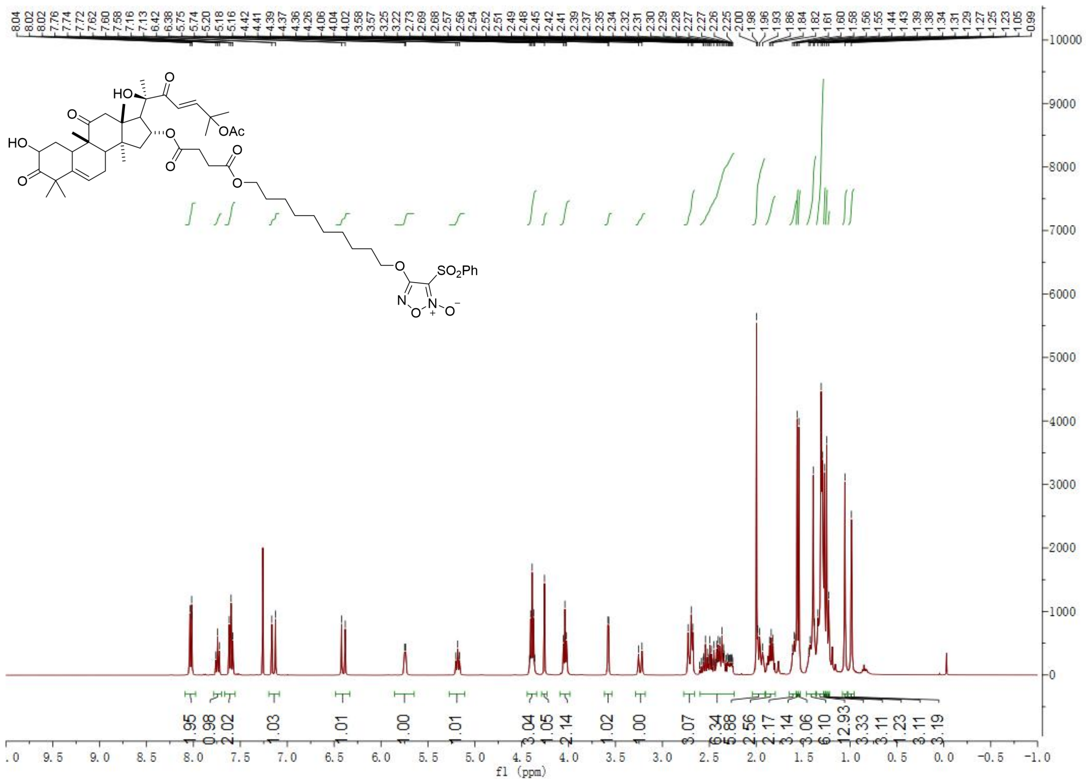

^{1}H NMR of compound **10i**

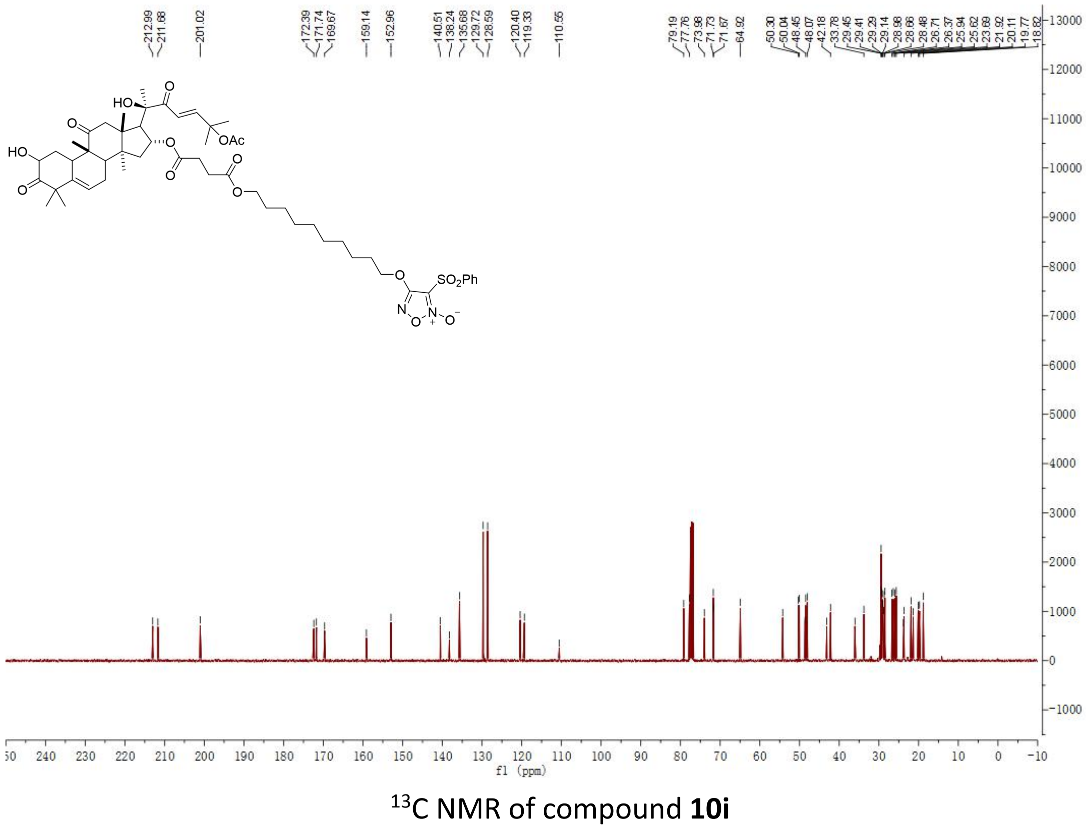

^{13}C NMR of compound **10i**

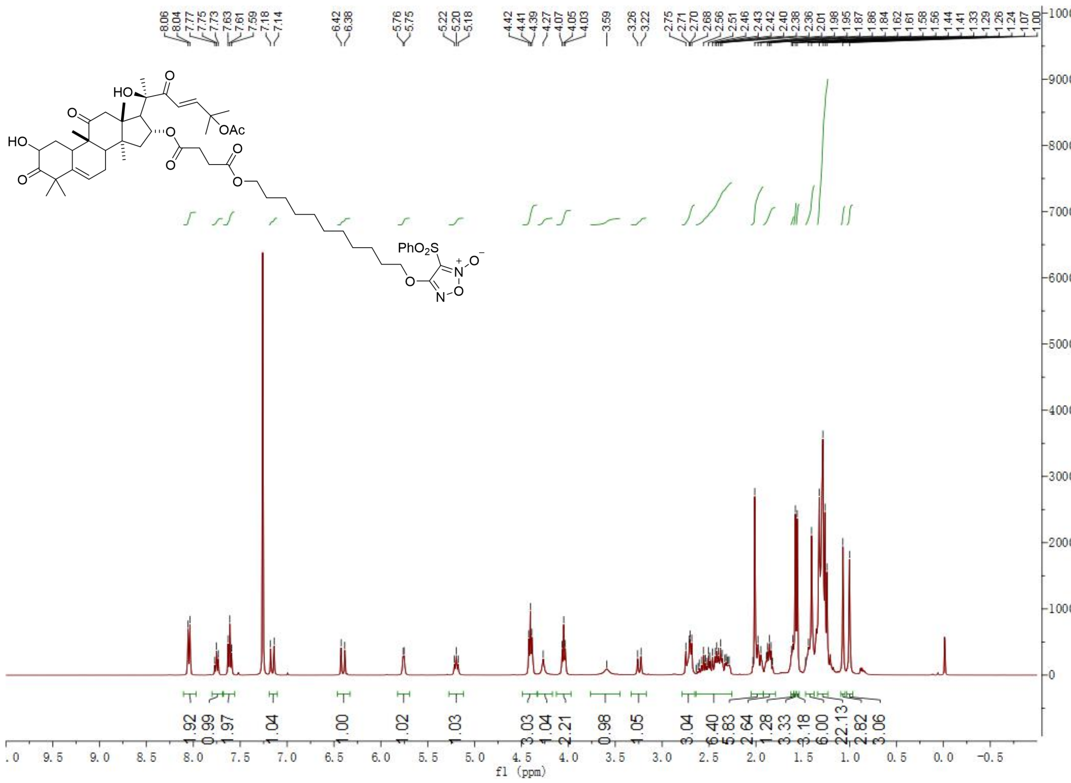

^{1}H NMR of compound **10j**

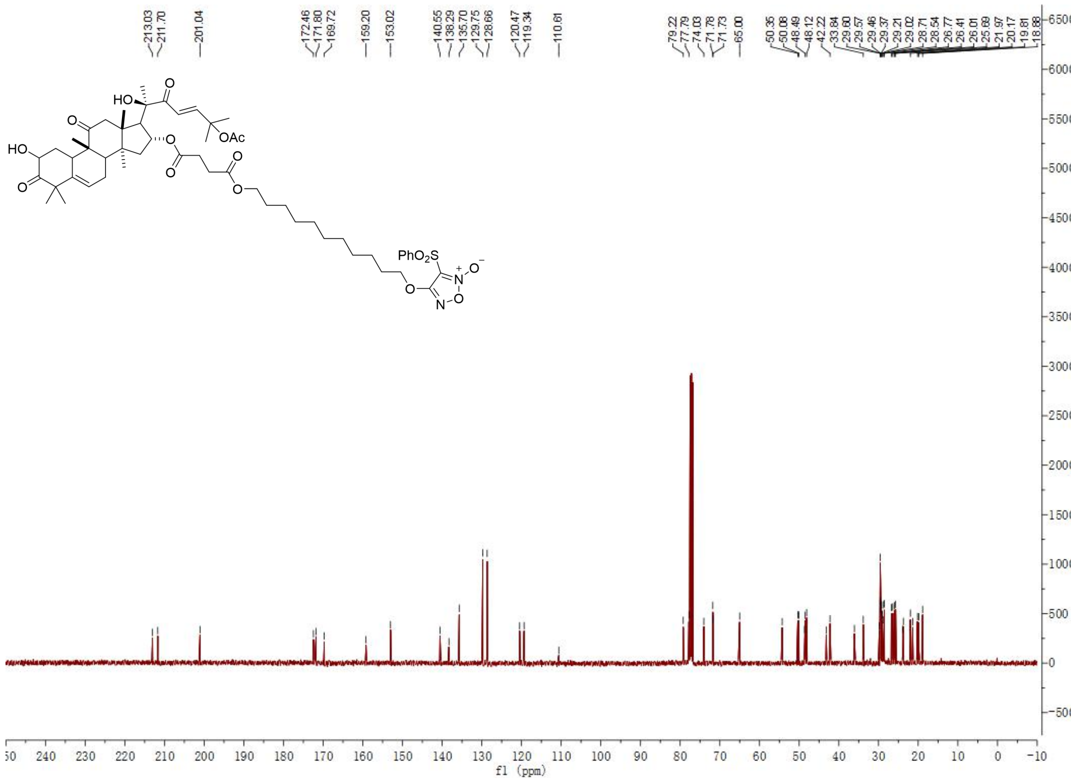

^{13}C NMR of compound **10j**

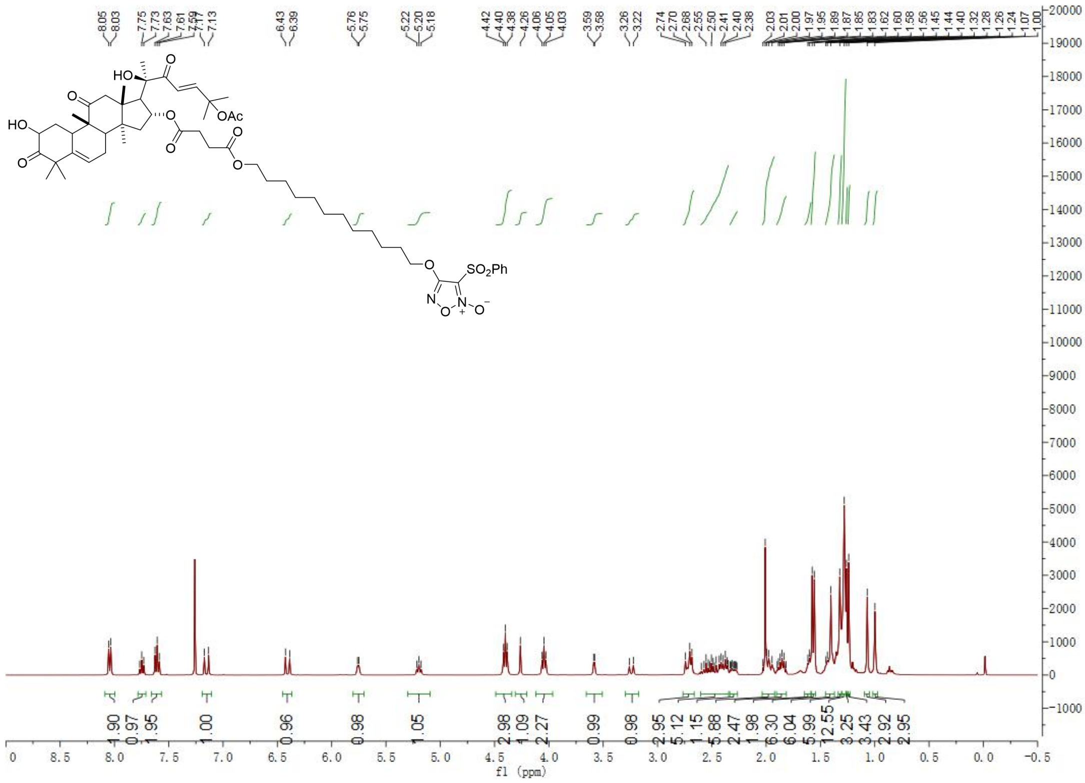

^{1}H NMR of compound **10k**

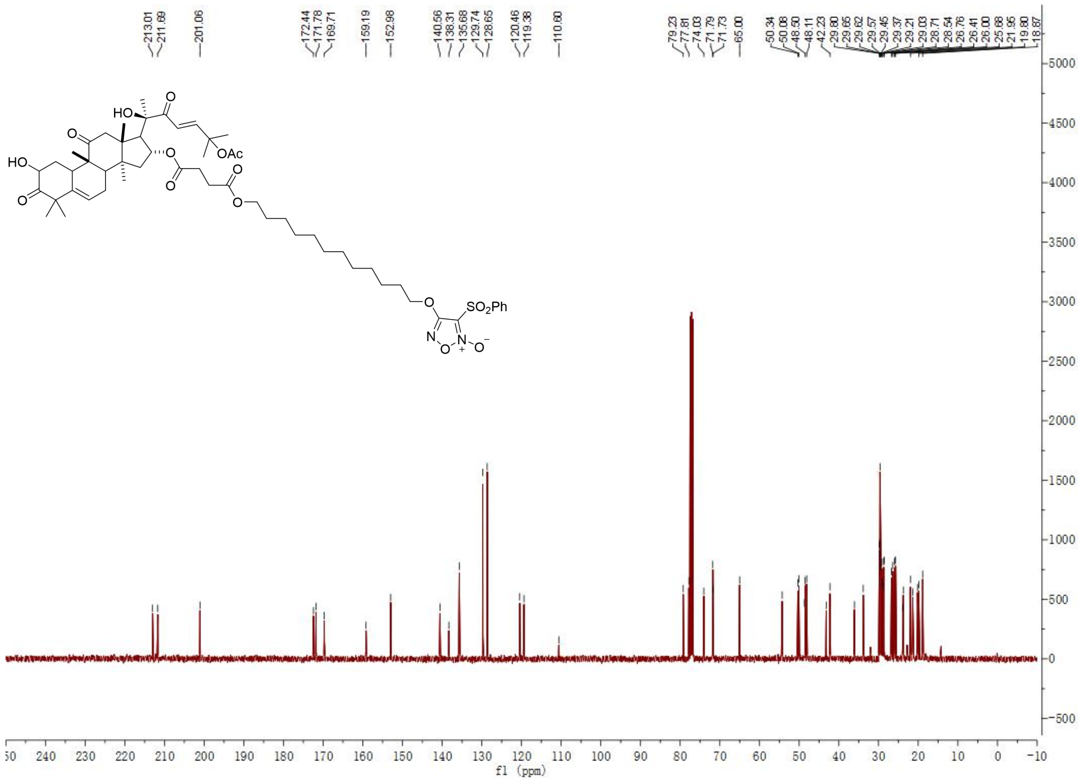

^{13}C NMR of compound **10k**

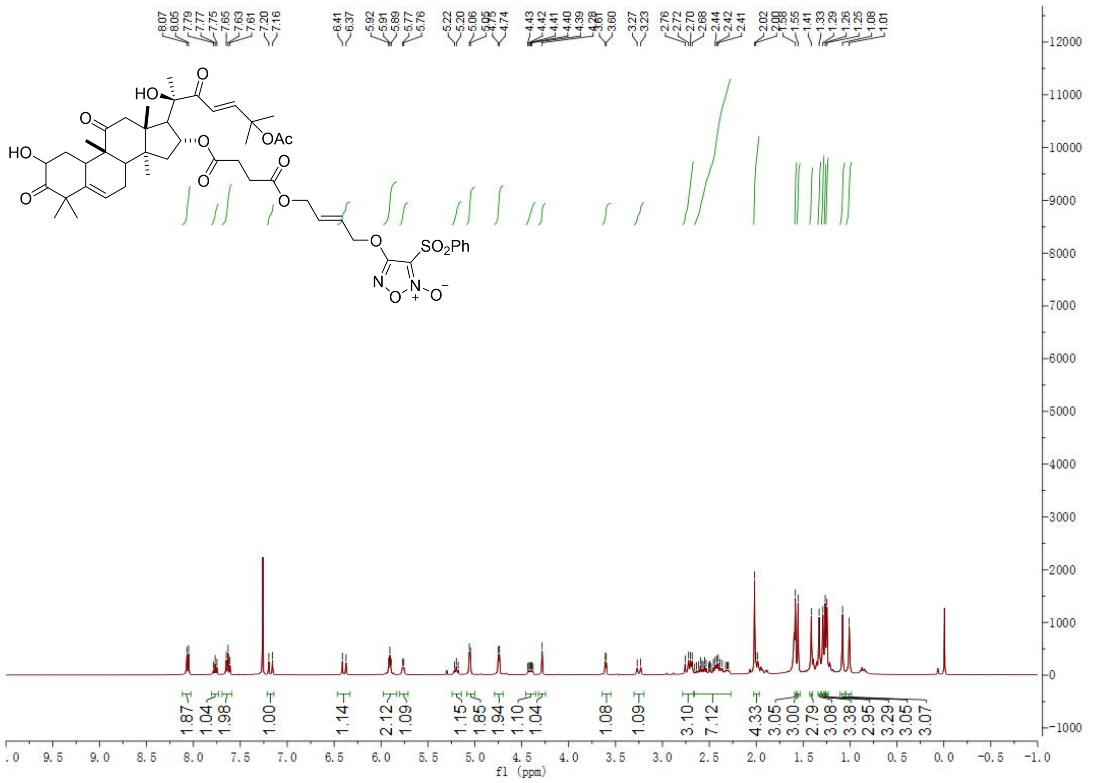

^{1}H NMR of compound **10l**

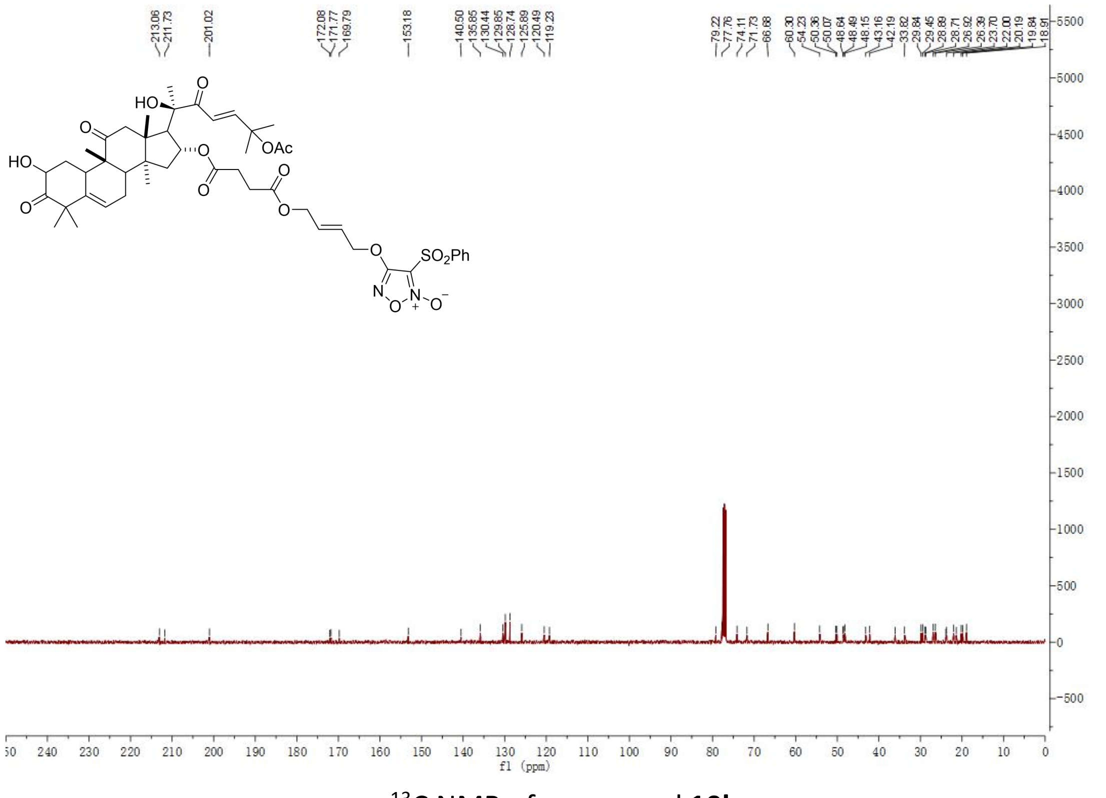

^{13}C NMR of compound **10l**

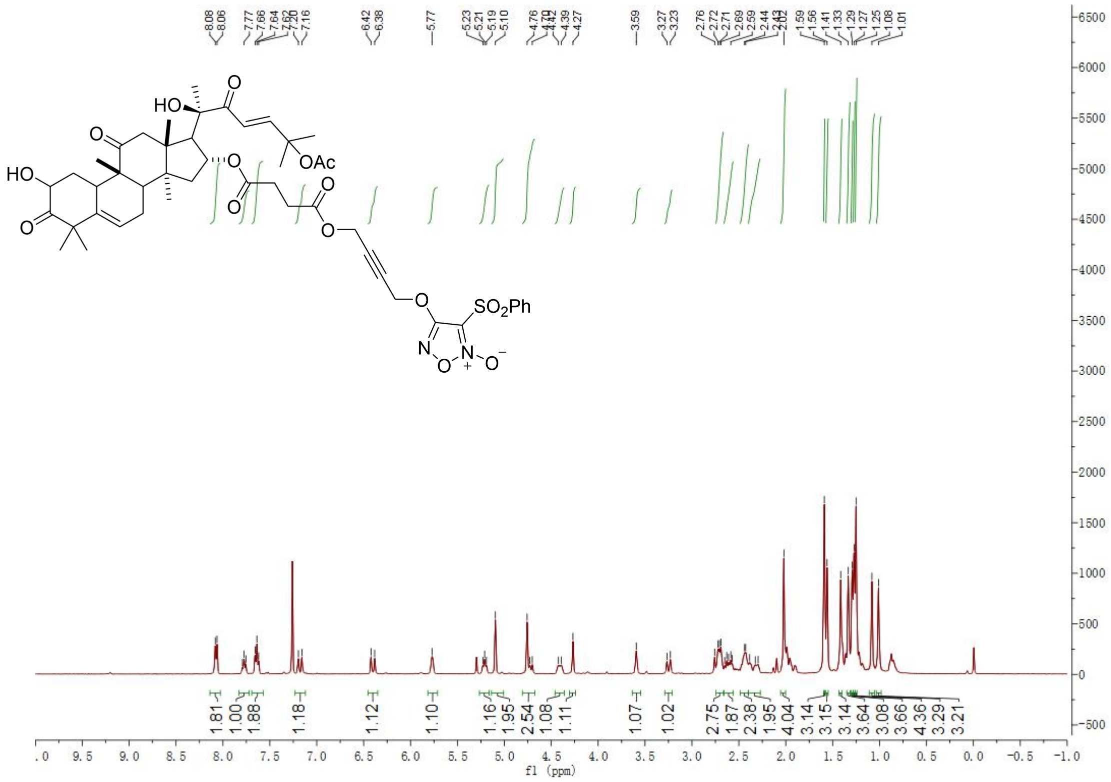

^{1}H NMR of compound **10m**

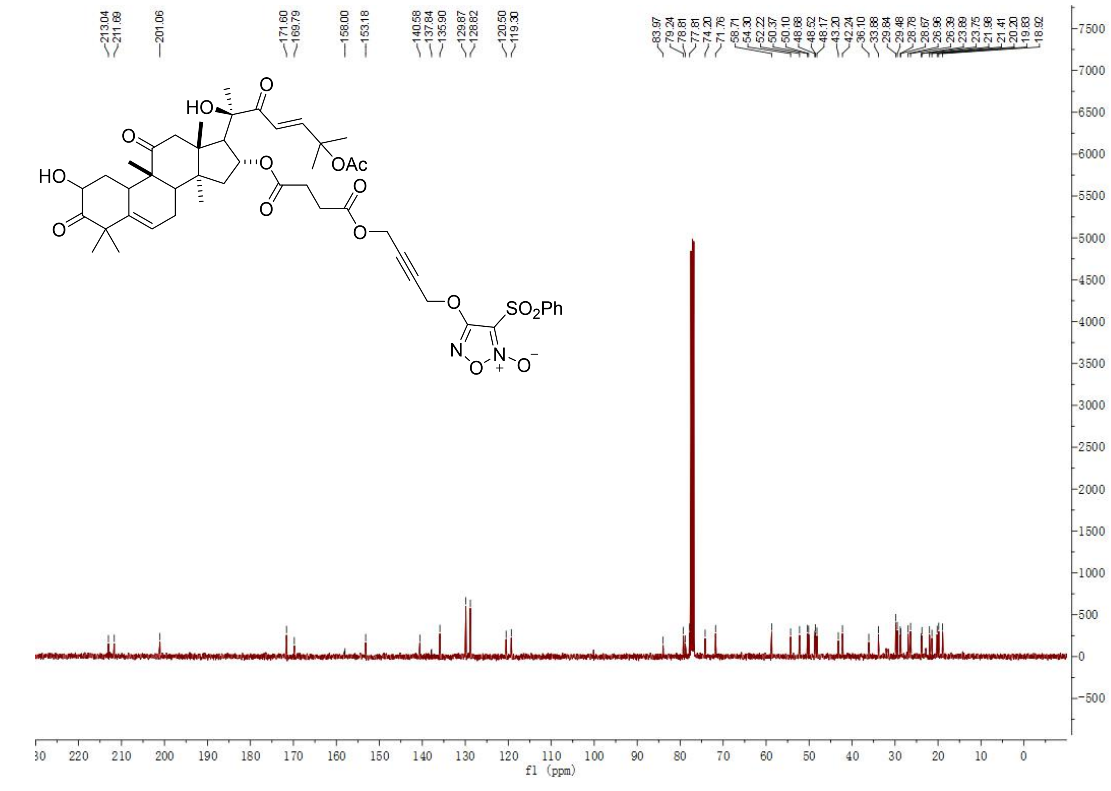

^{13}C NMR of compound **10m**

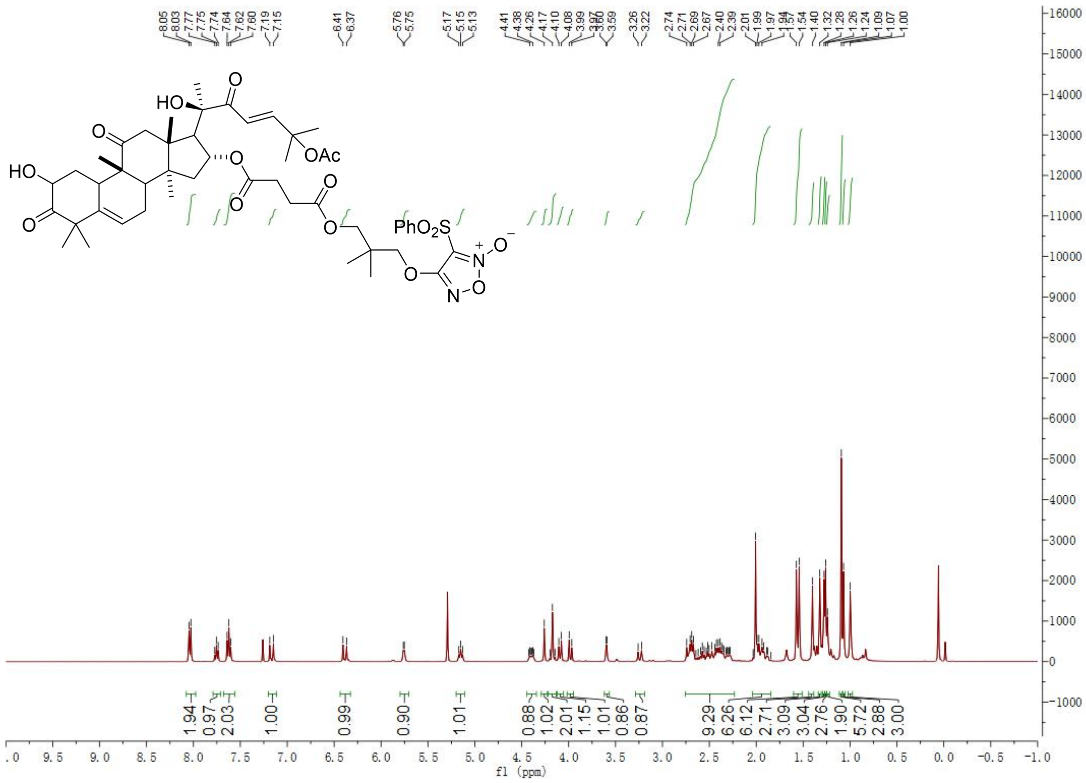

^{1}H NMR of compound **10n**

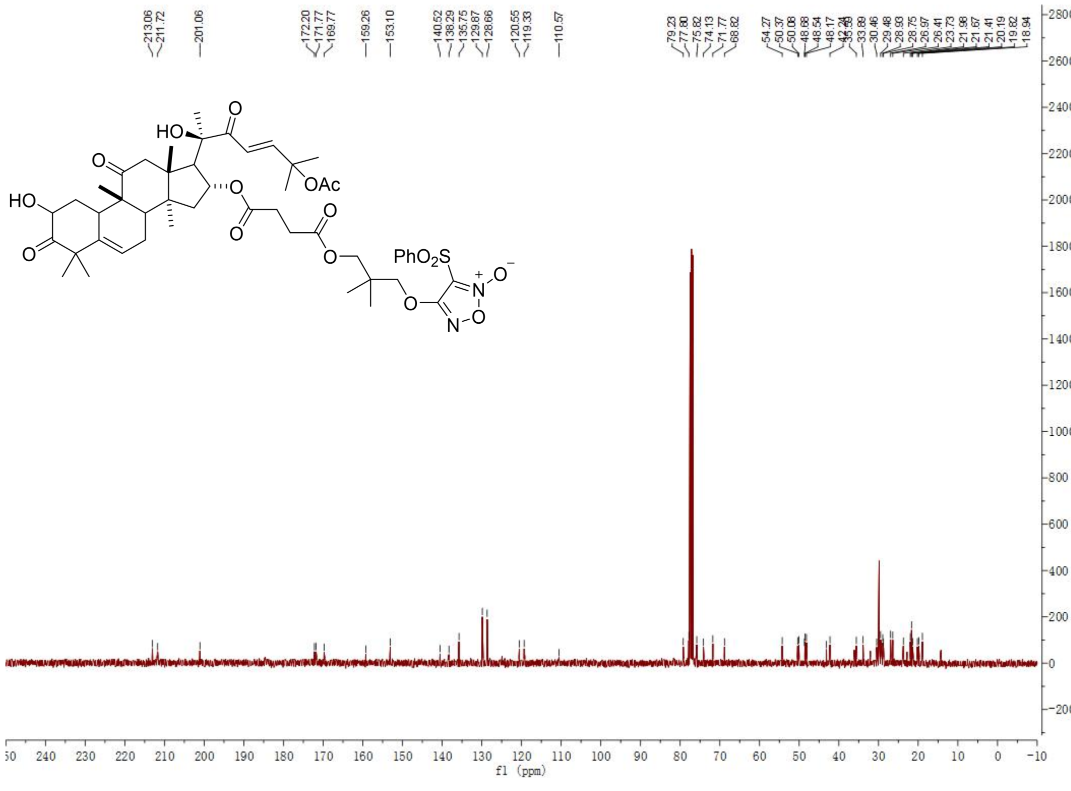

^{13}C NMR of compound **10n**

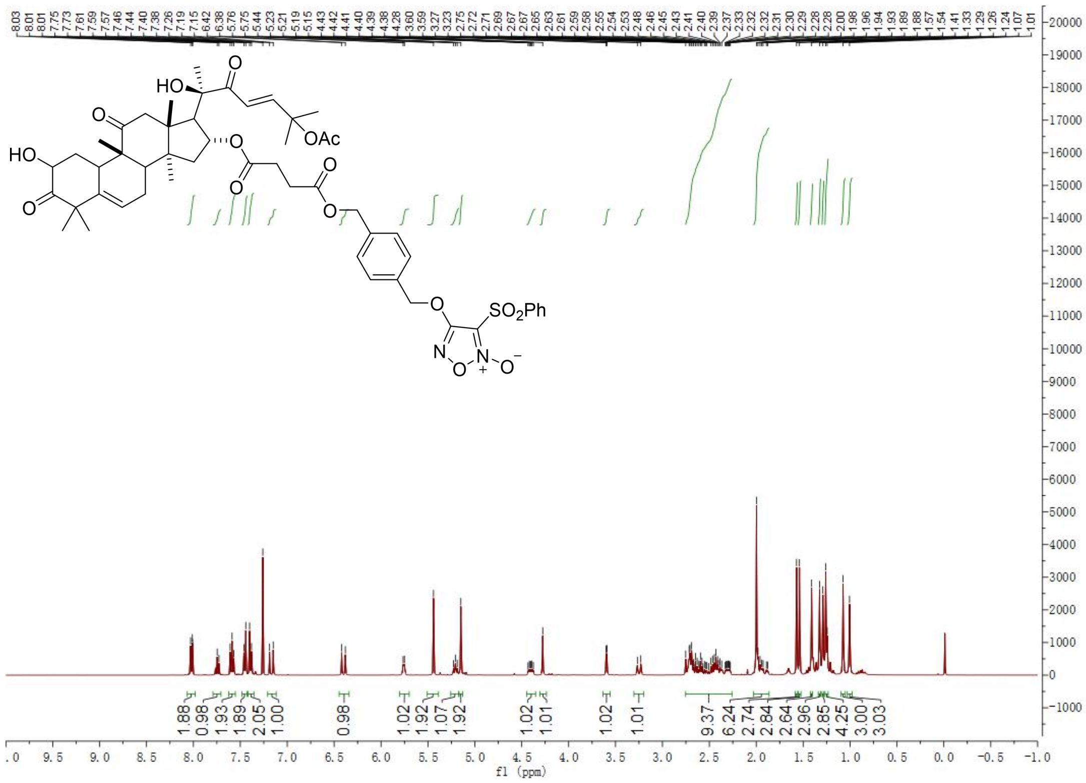

^{1}H NMR of compound **10o**

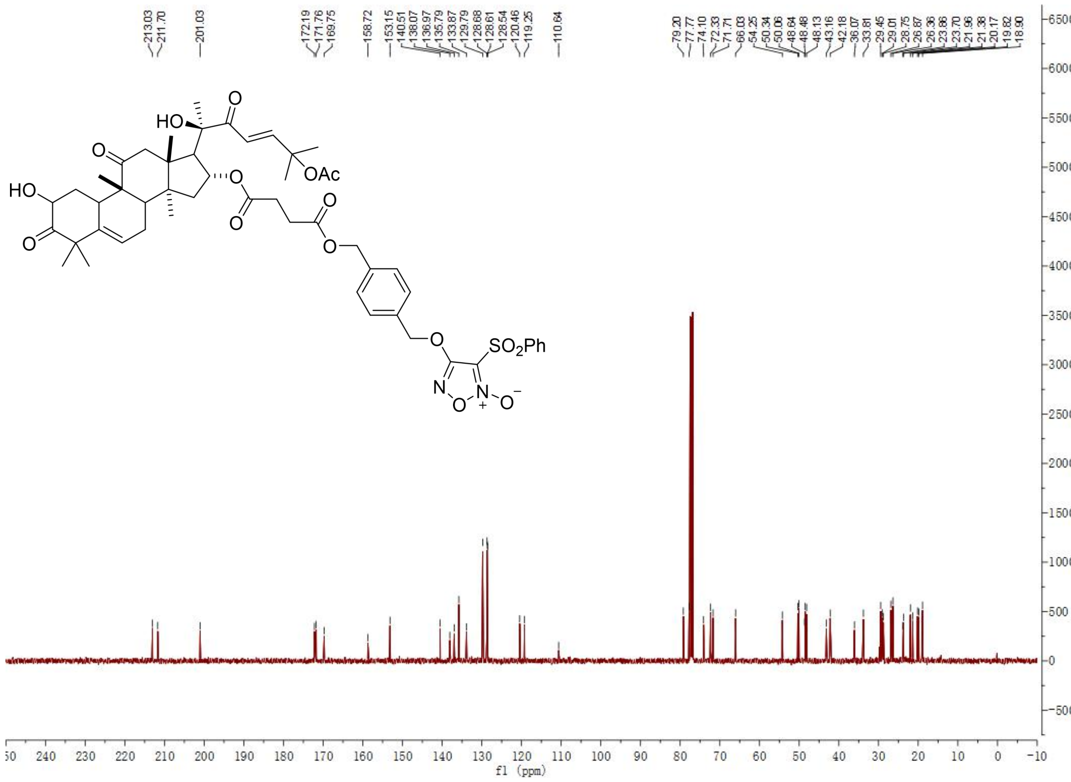

^{13}C NMR of compound **10o**

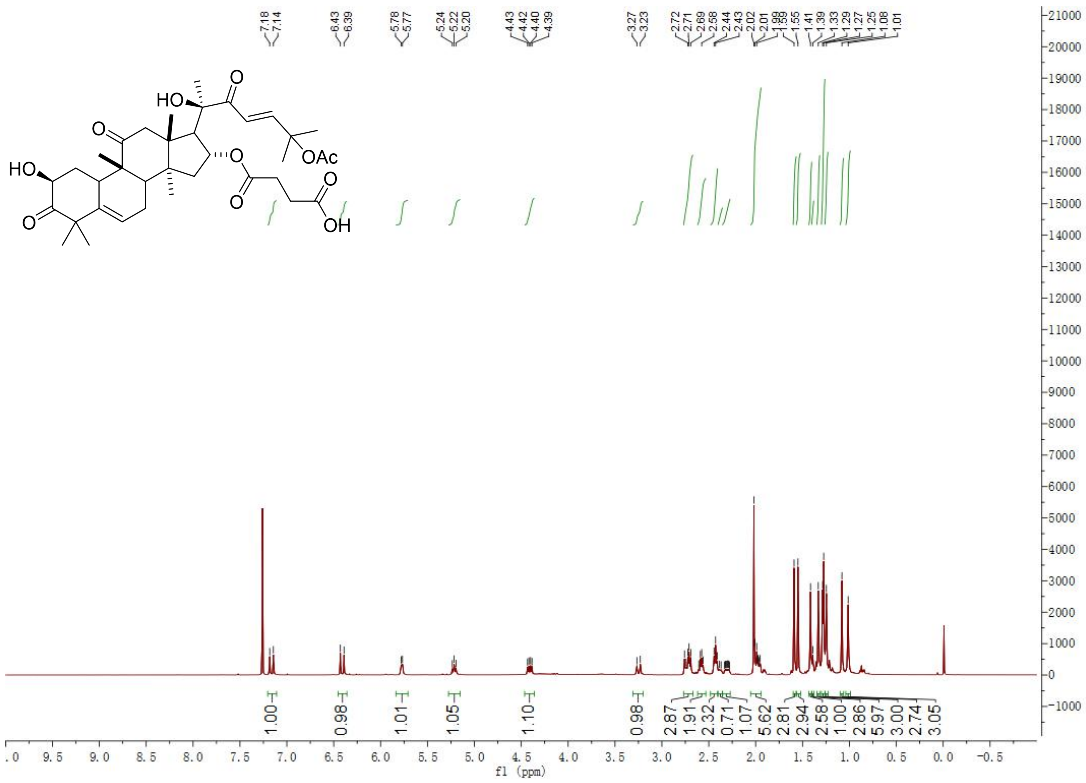

^{1}H NMR of compound **11**

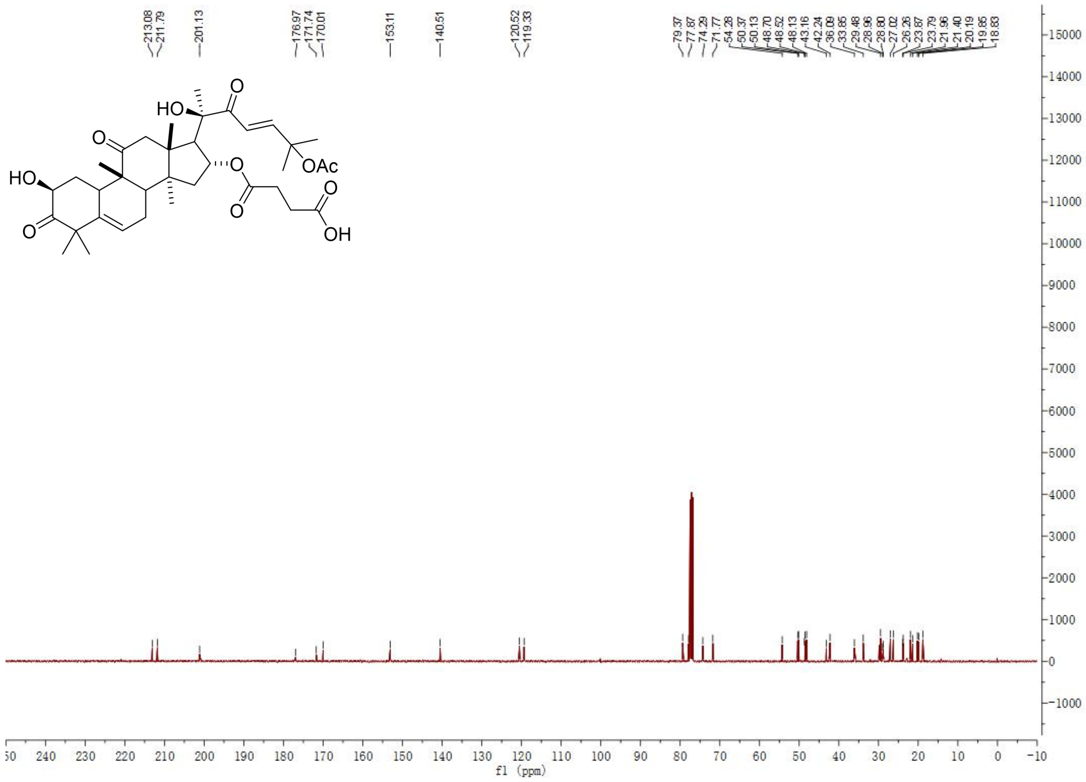

^{13}C NMR of compound **11**

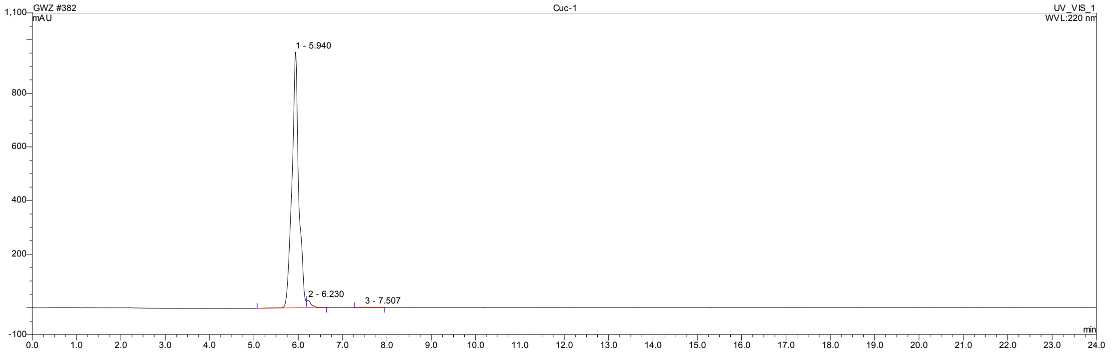

Compound **1**

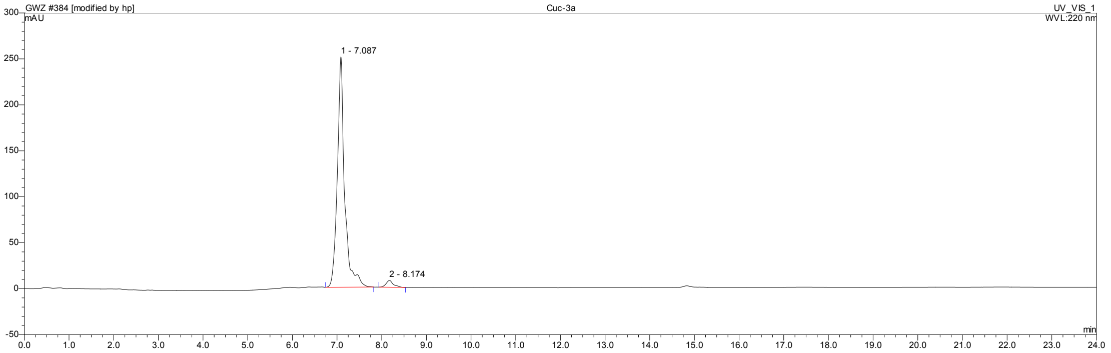

Compound **3a**

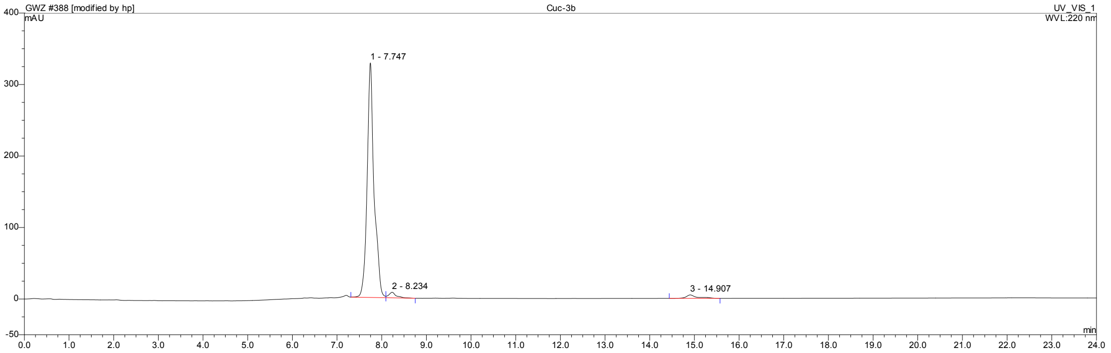

Compound **3b**

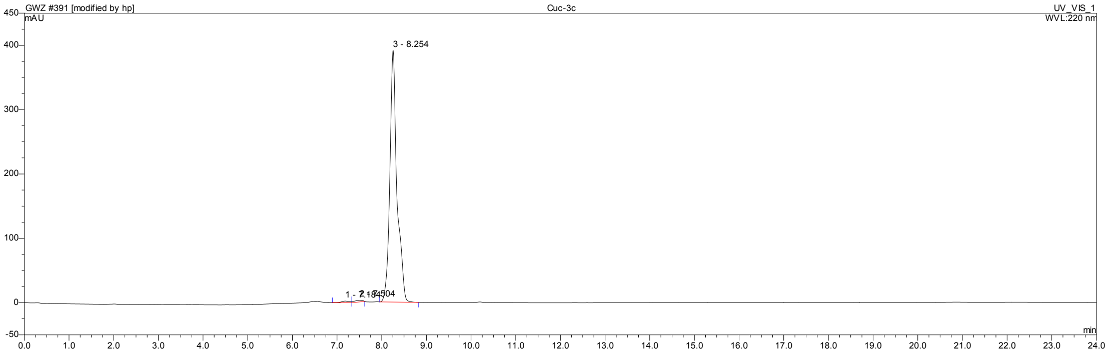

Compound **3c**

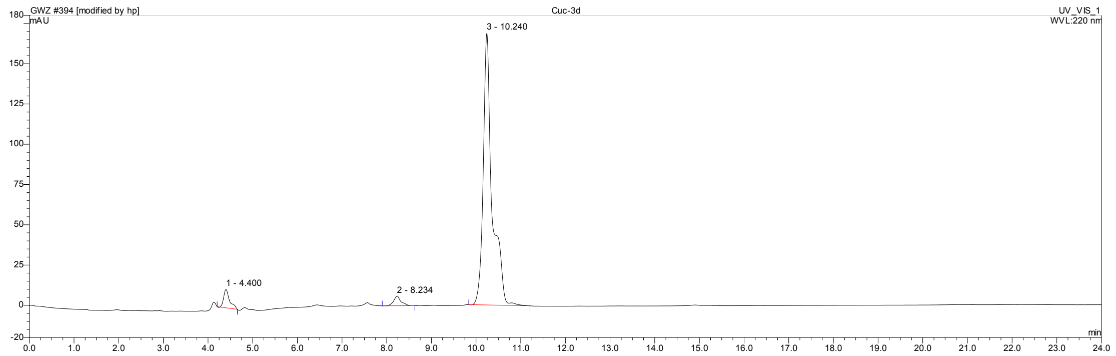

Compound
3d

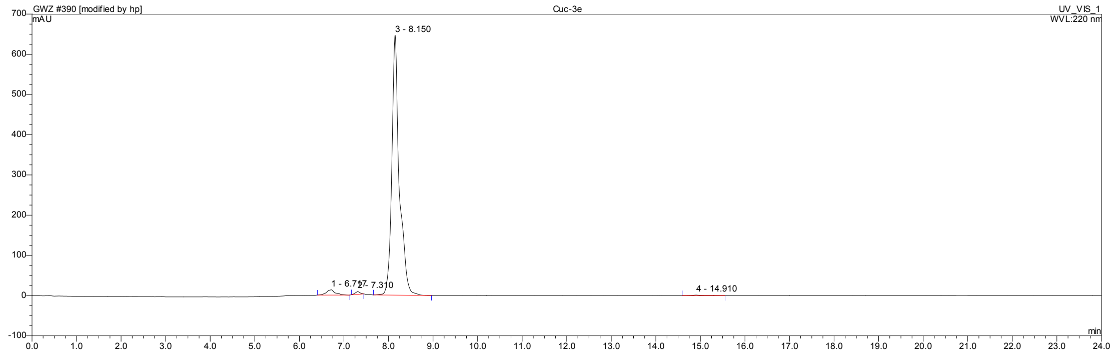

Compound **3e**

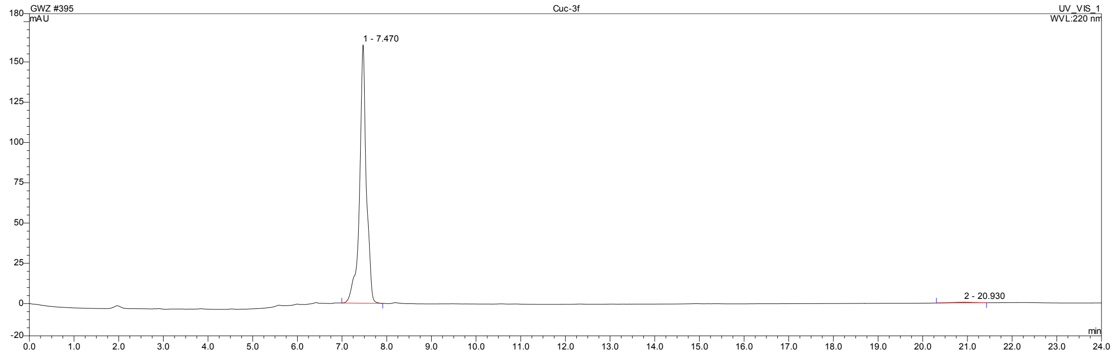

Compound **3f**

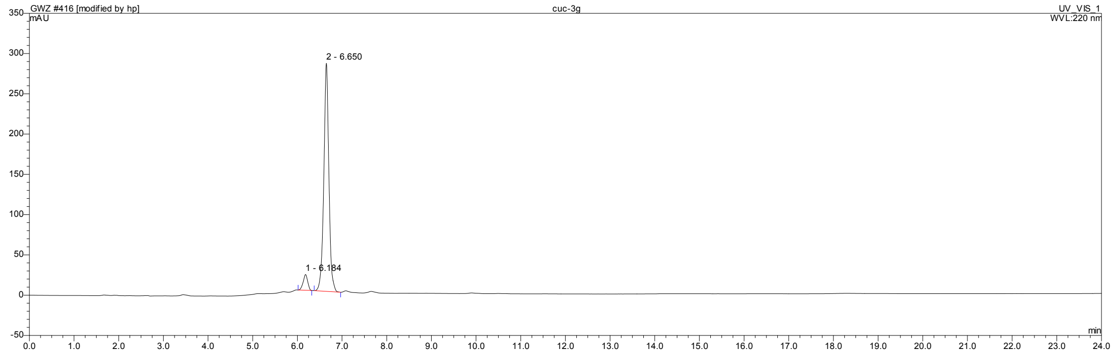

Compound **3g**

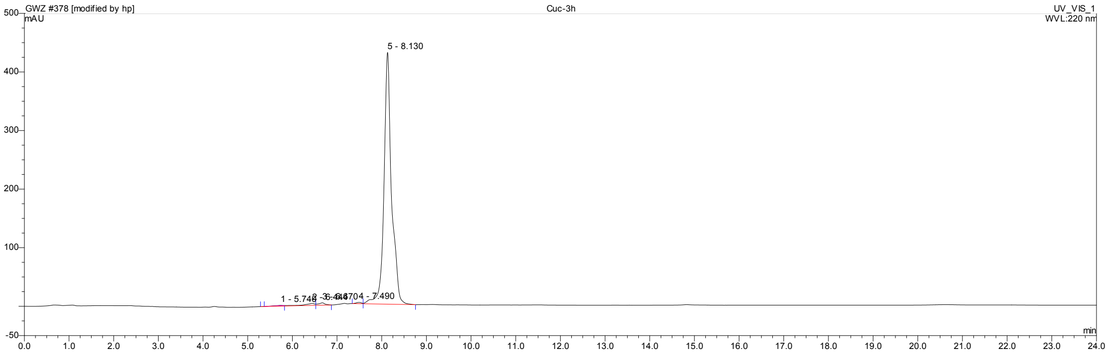

Compound **3h**

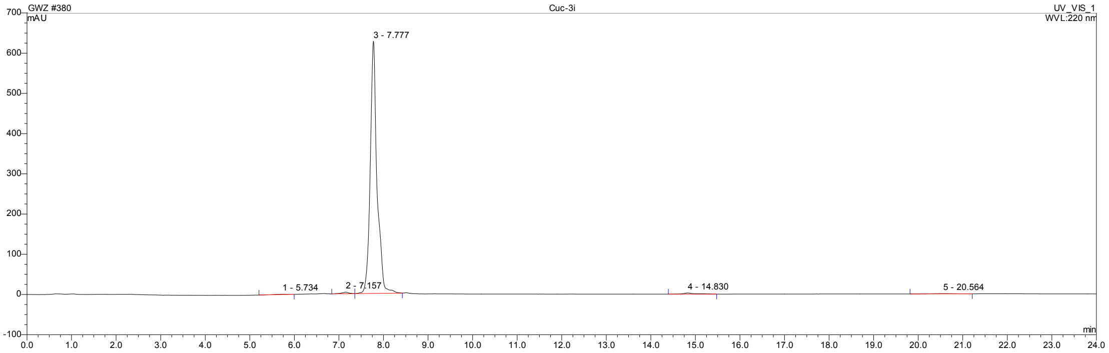

Compound **3i**

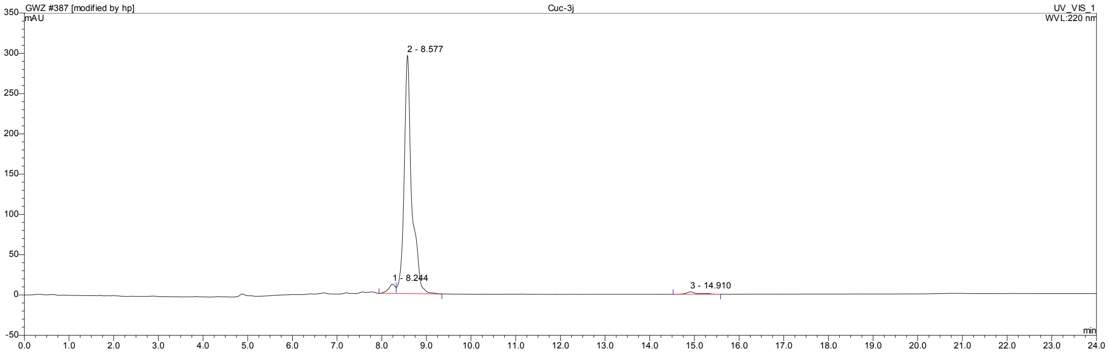

Compound **3j**

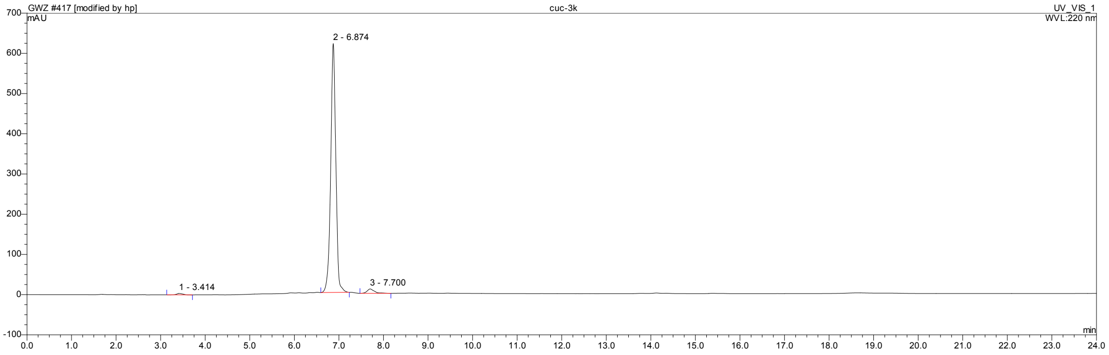

Compound **3k**

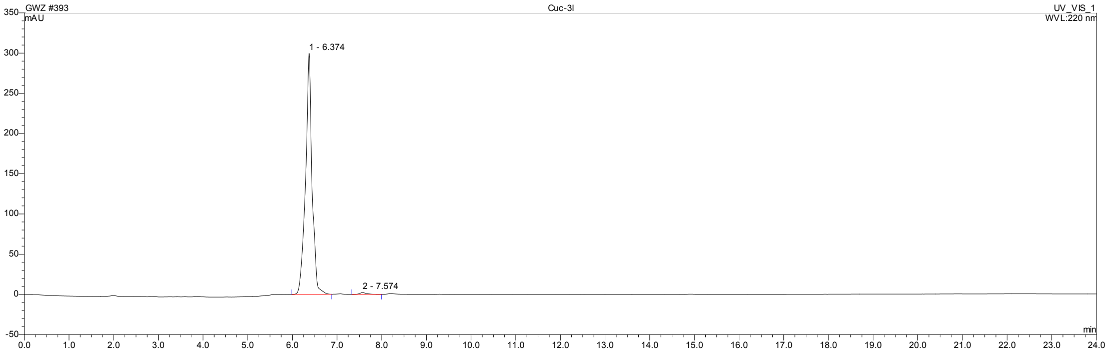

Compound **3l**

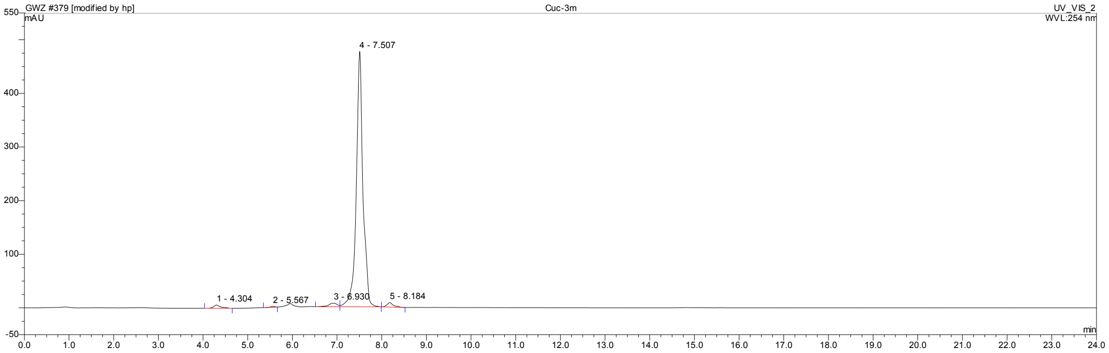

Compound **3m**

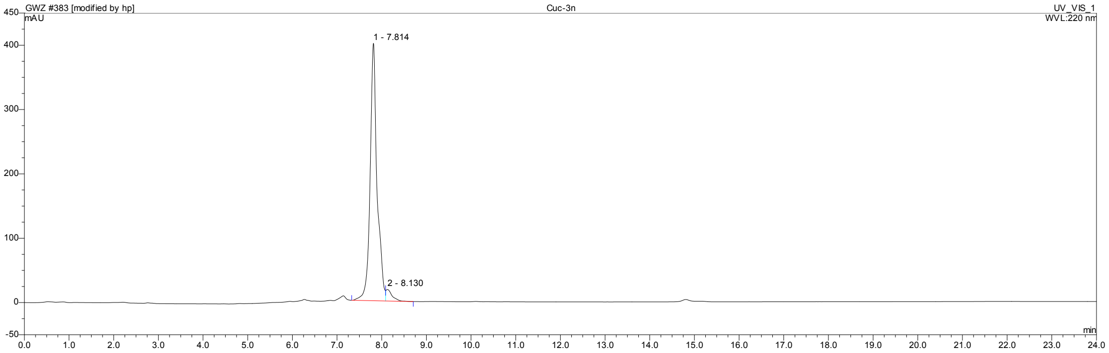

Compound **3n**

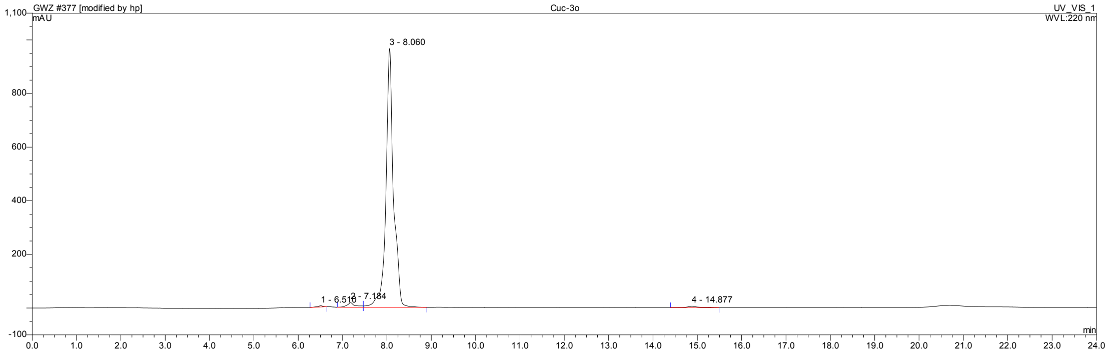

Compound **3o**

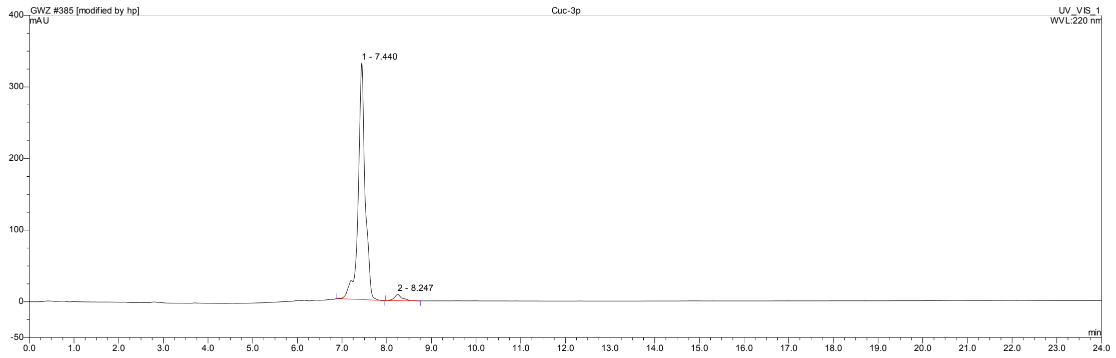

Compound **3p**

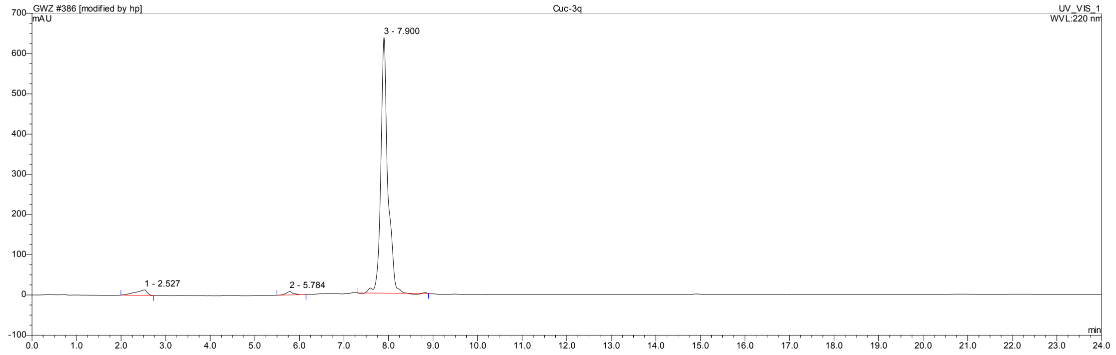

Compound **3q**

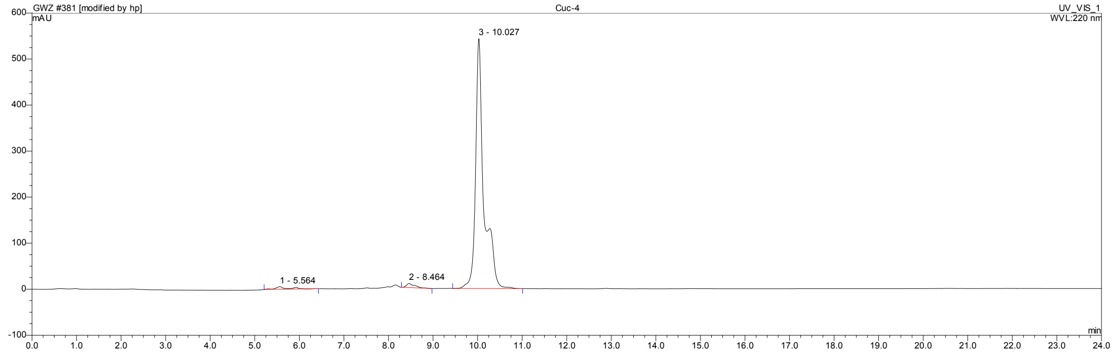

Compound **4**

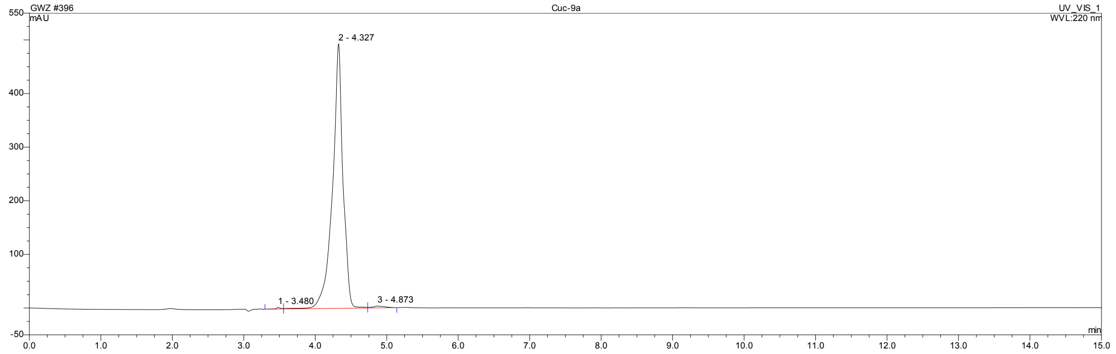

Compound **9a**

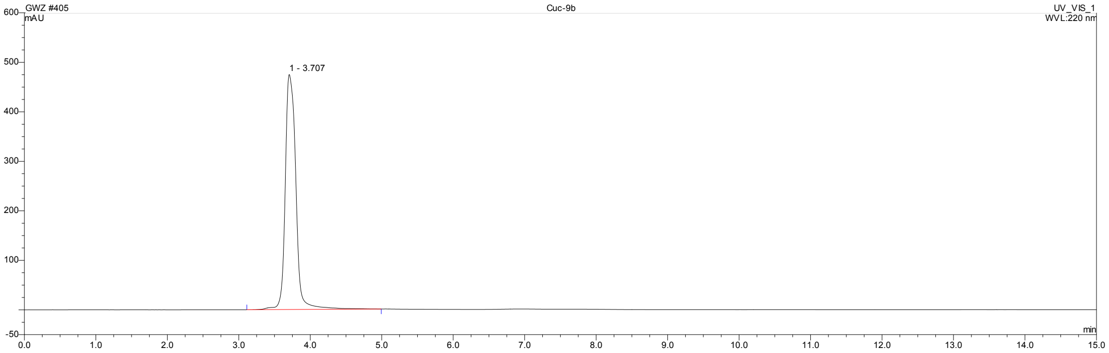

Compound **9b**

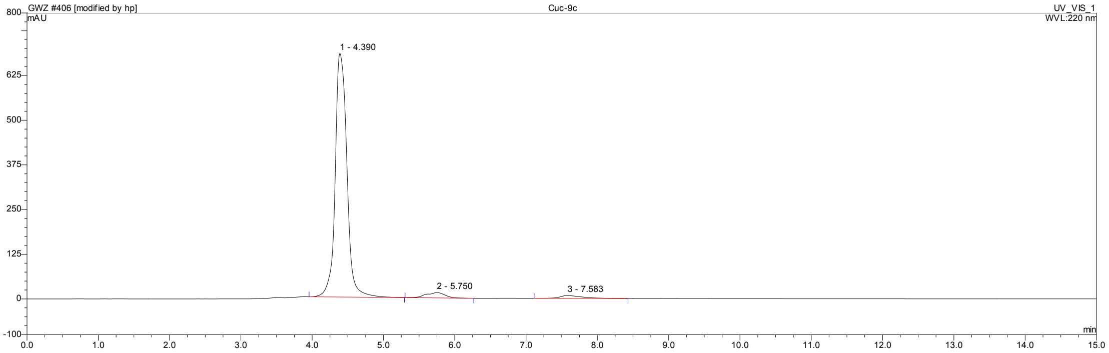

Compound **9c**

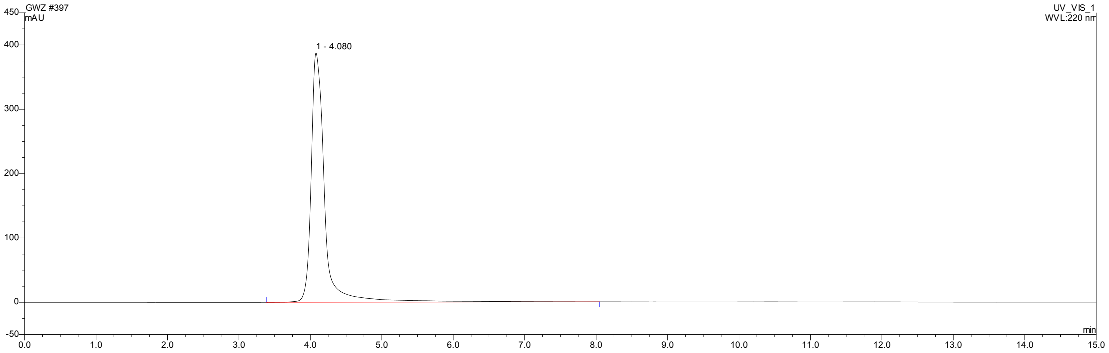

Compound **10a**

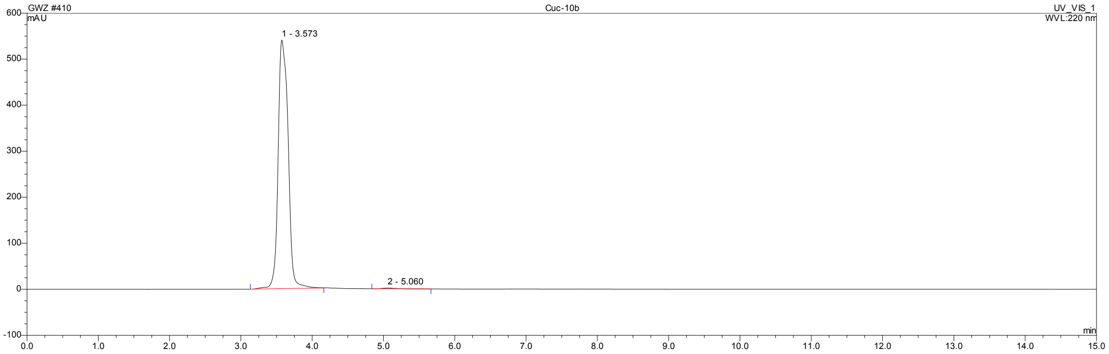

Compound **10b**

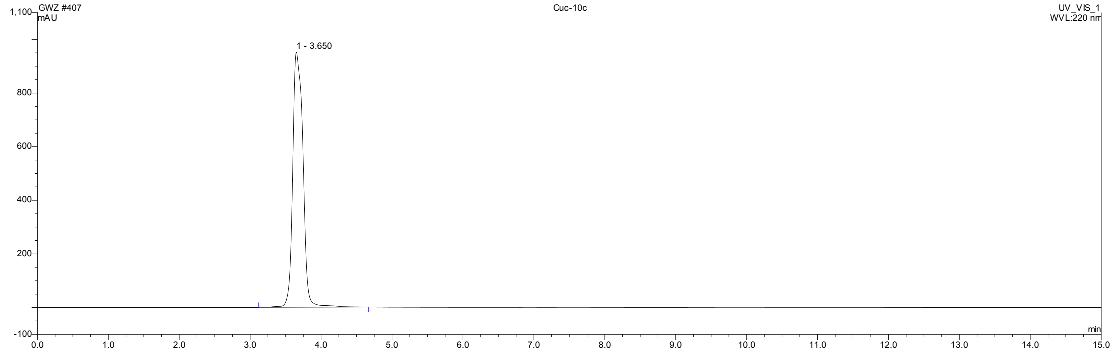

Compound **10c**

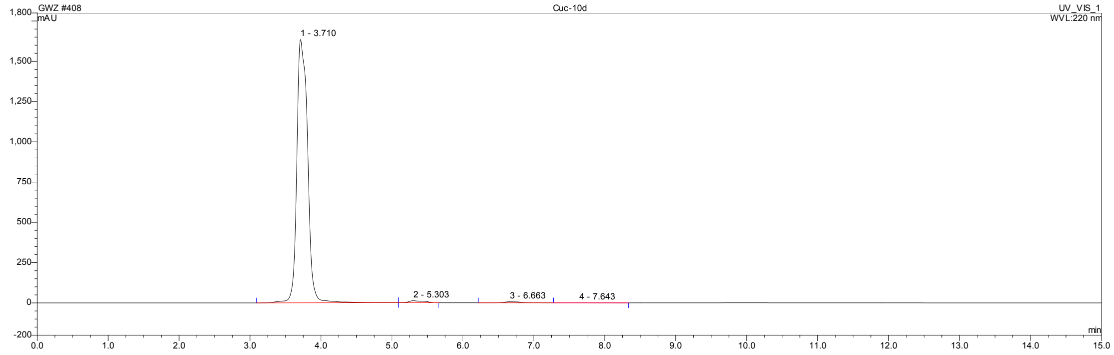

Compound **10d**

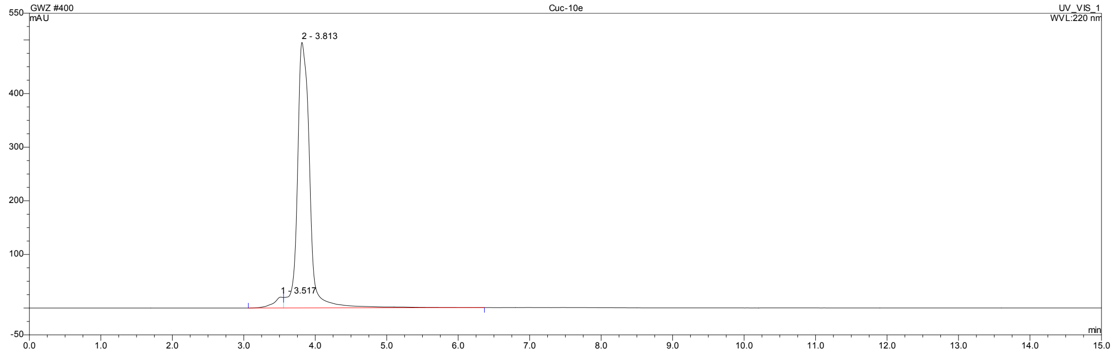

Compound **10e**

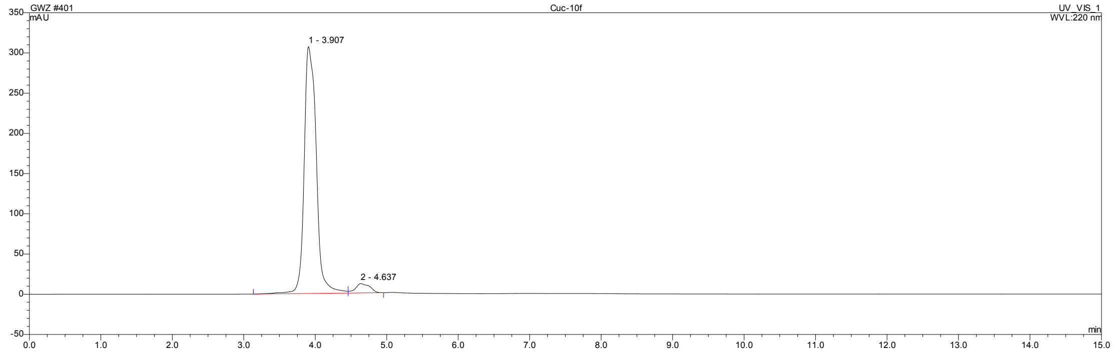

Compound **10f**

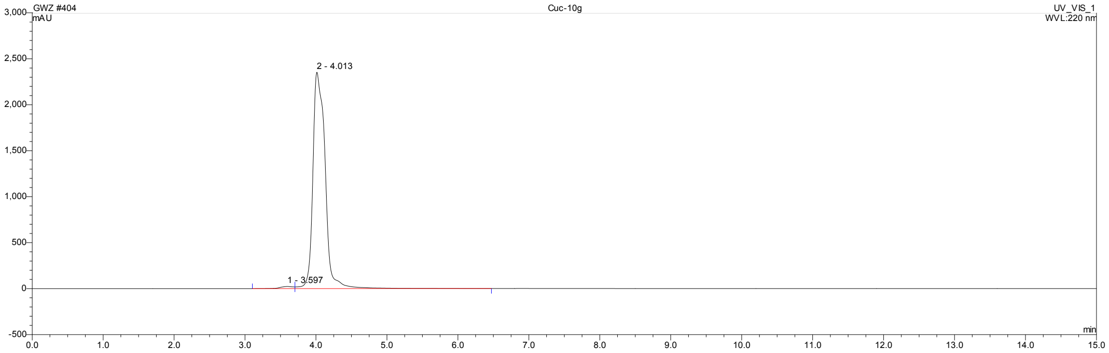

Compound **10g**

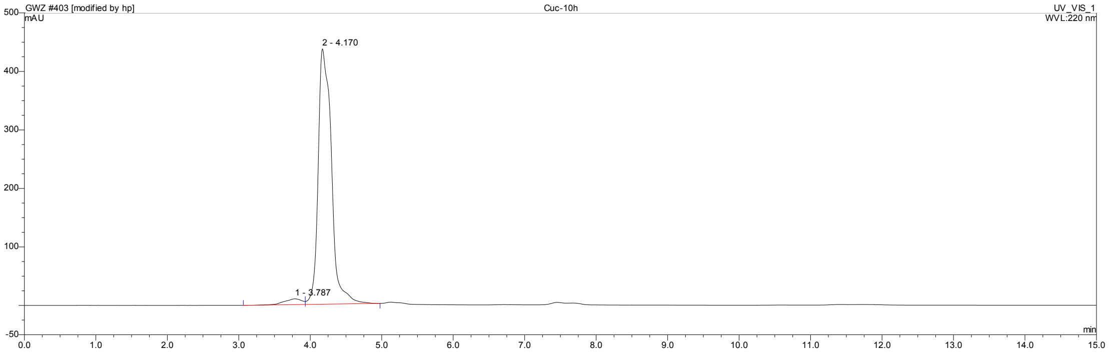

Compound **10h**

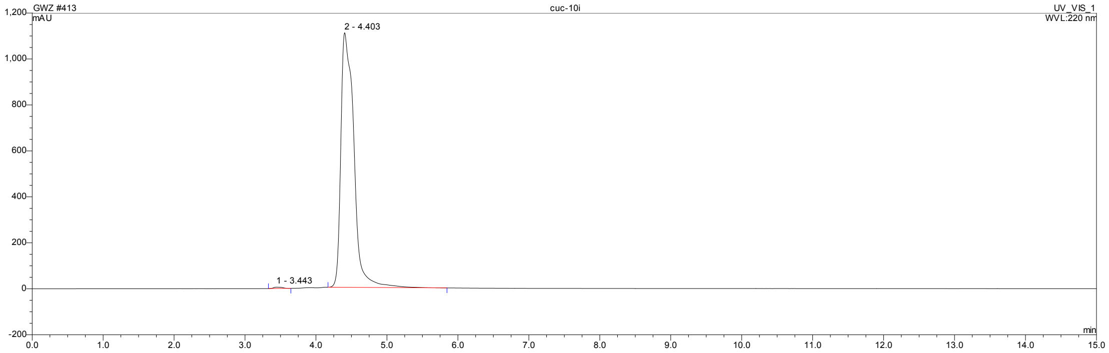

Compound **10i**

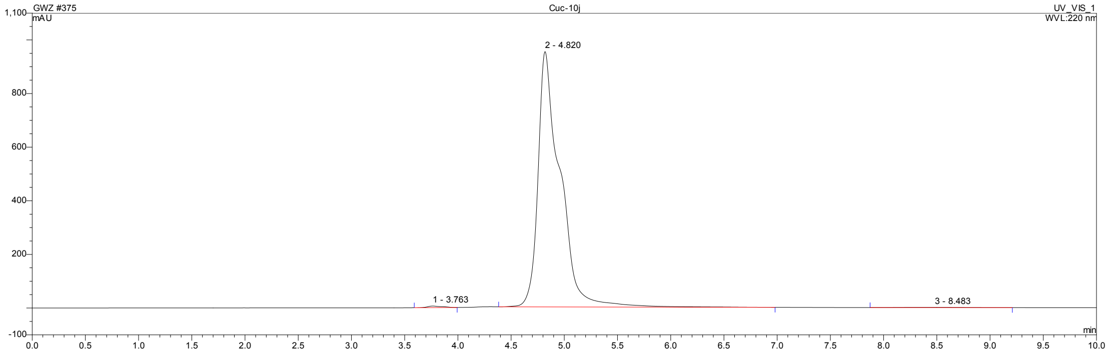

Compound **10j**

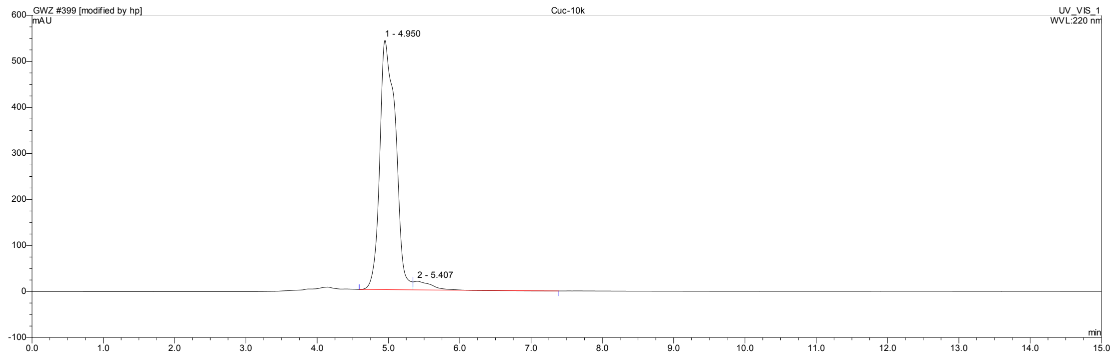

Compound **10k**

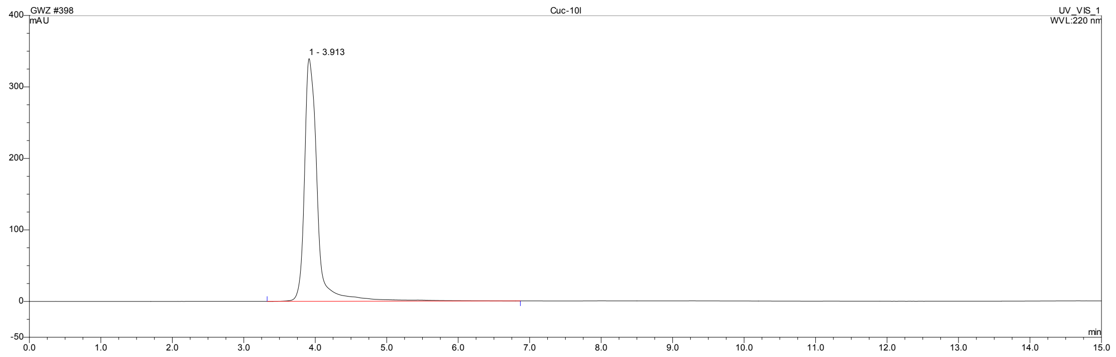

Compound **10l**

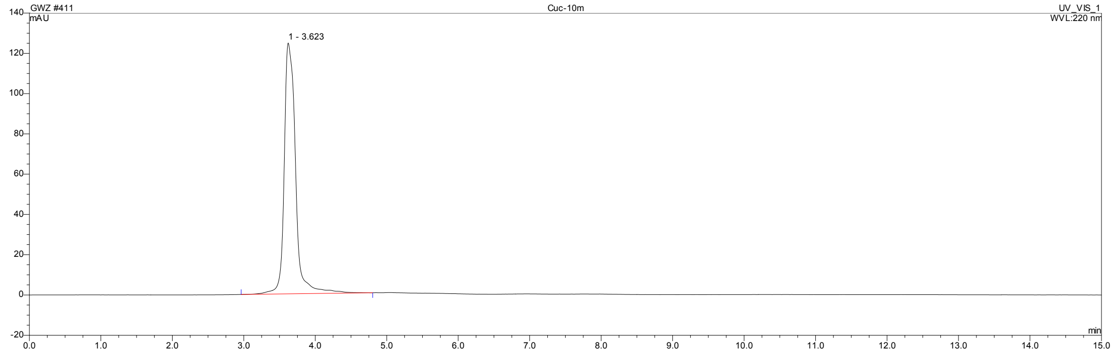

Compound **10m**

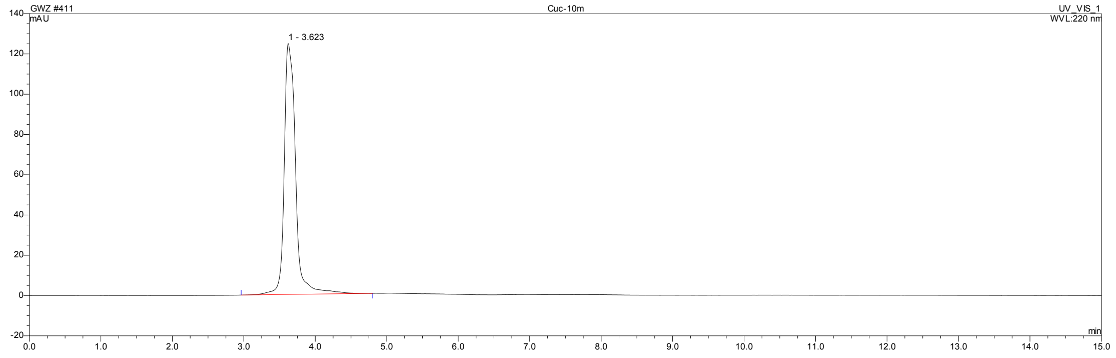

Compound **10n**

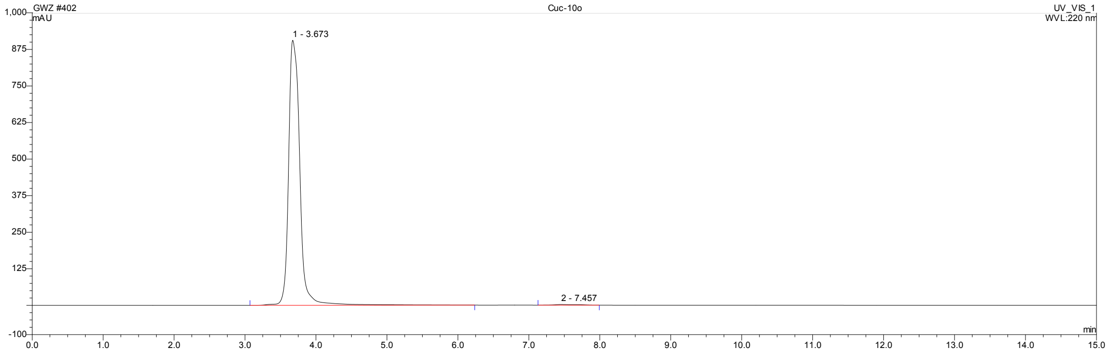

Compound **10o**

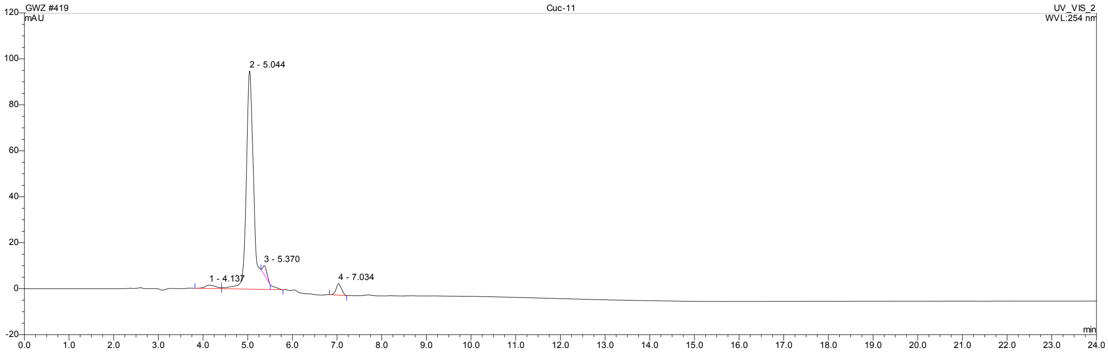

Compound **11**

3. The dose-response curves of compounds

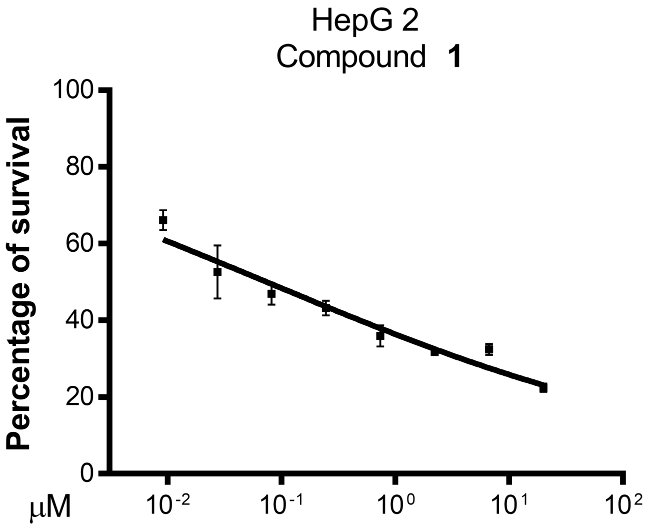

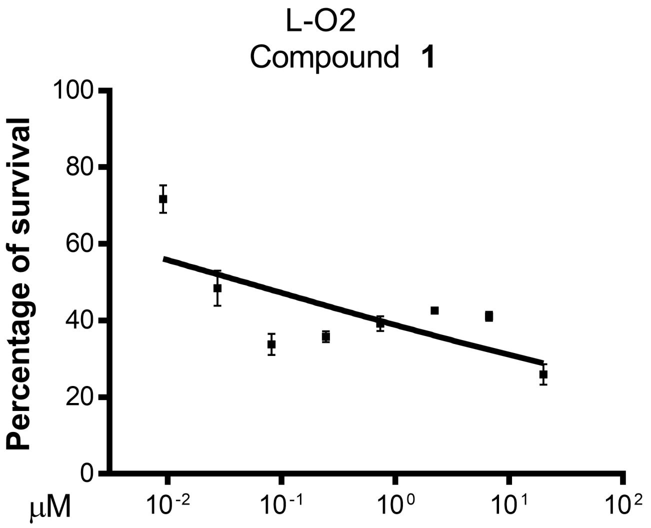

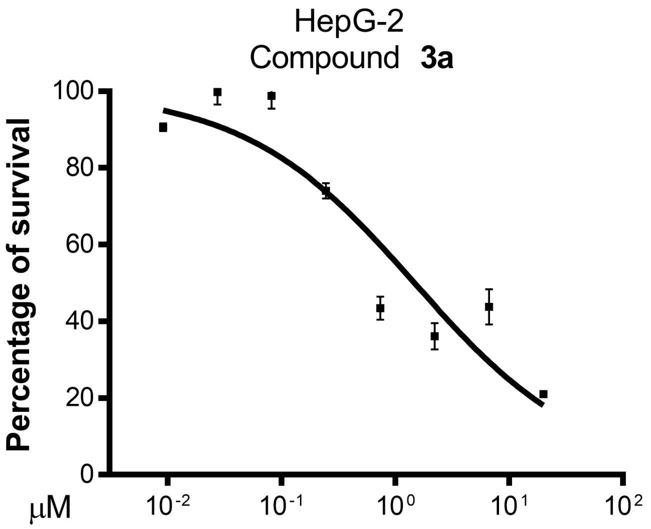

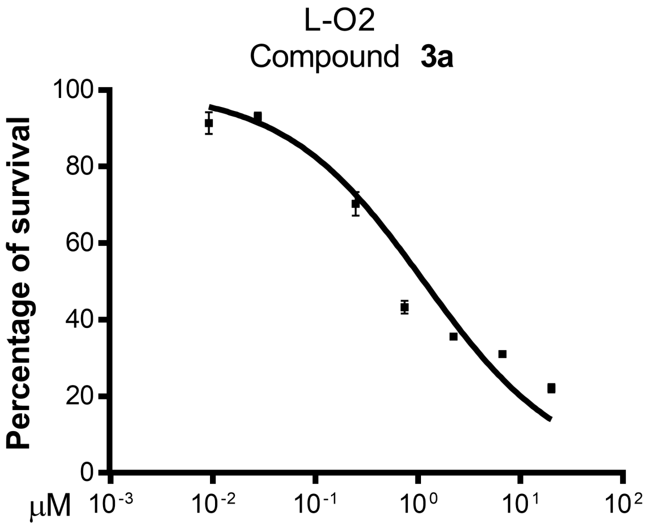
L-O2
Compound **3a**
Percentage of survival
0
20
40
60
80
100
μM
10^{-3}
10^{-2}
10^{-1}
10^{0}
10^{1}
10^{2}

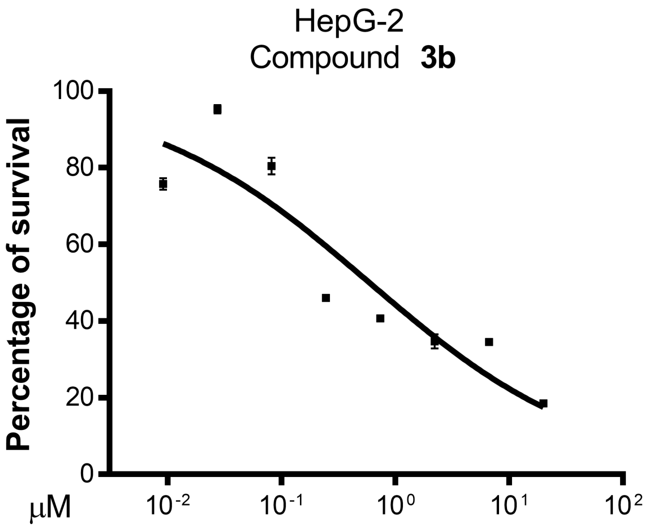
HepG-2
Compound **3b**
Percentage of survival
0
20
40
60
80
100
μM
10^{-2}
10^{-1}
10^{0}
10^{1}
10^{2}

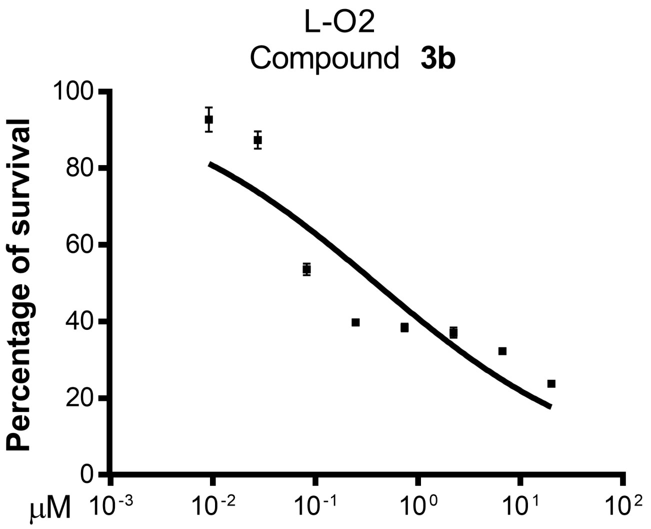
L-O2
Compound **3b**
Percentage of survival
0
20
40
60
80
100
μM
10^{-3}
10^{-2}
10^{-1}
10^{0}
10^{1}
10^{2}

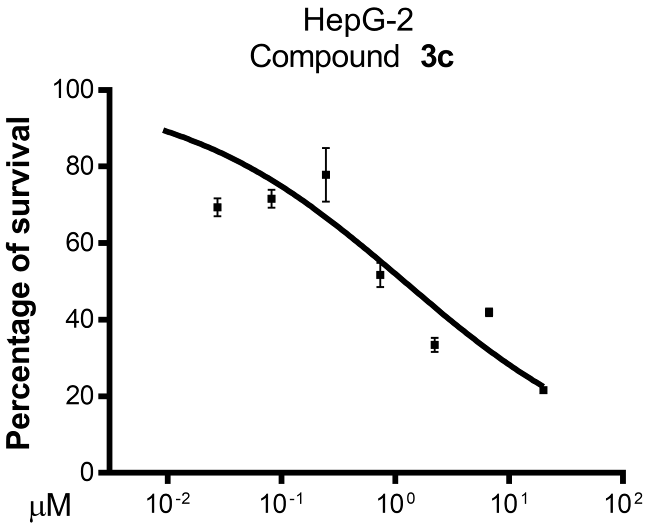
HepG-2
Compound **3c**
Percentage of survival
100
80
60
40
20
0
μM
10^{-2}
10^{-1}
10^{0}
10^{1}
10^{2}

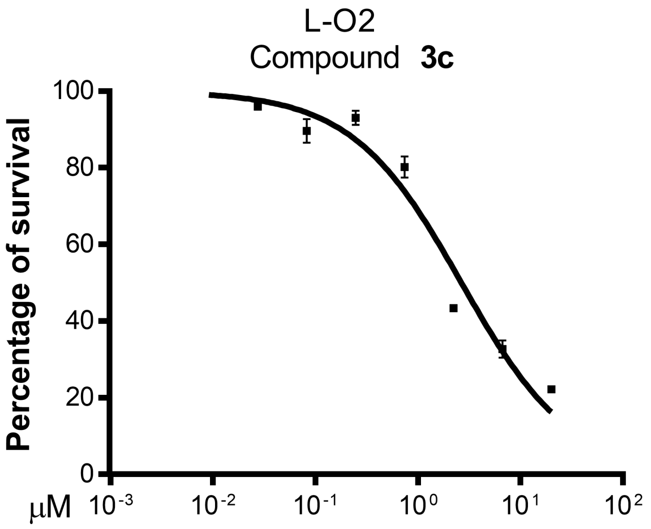
L-O2
Compound **3c**
Percentage of survival
100
80
60
40
20
0
μM
10^{-3}
10^{-2}
10^{-1}
10^{0}
10^{1}
10^{2}

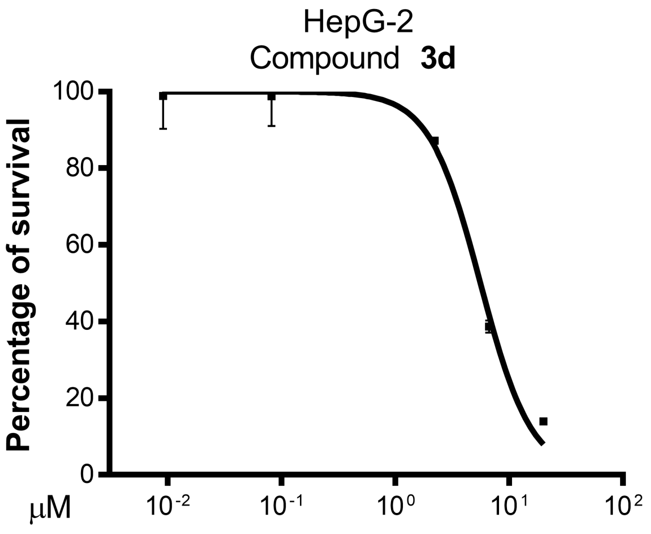
HepG-2
Compound **3d**
Percentage of survival
100
80
60
40
20
0
μM
10^{-2}
10^{-1}
10^{0}
10^{1}
10^{2}

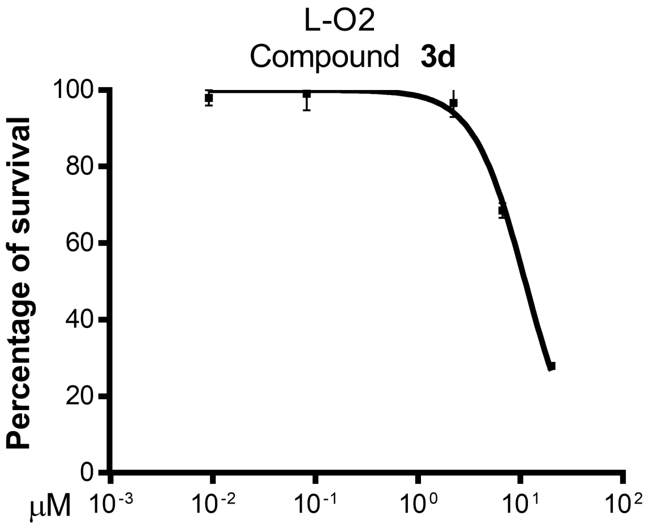
L-O2
Compound 3d
Percentage of survival
100
80
60
40
20
0
μM
10^{-3}
10^{-2}
10^{-1}
10^{0}
10^{1}
10^{2}

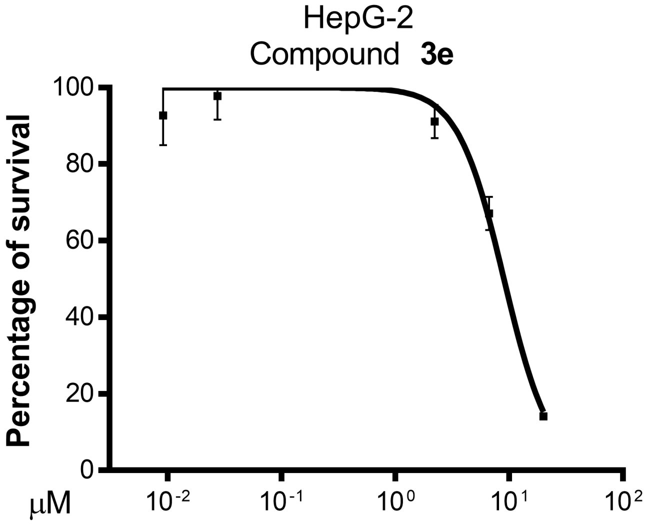
HepG-2
Compound 3e
Percentage of survival
100
80
60
40
20
0
μM
10^{-2}
10^{-1}
10^{0}
10^{1}
10^{2}

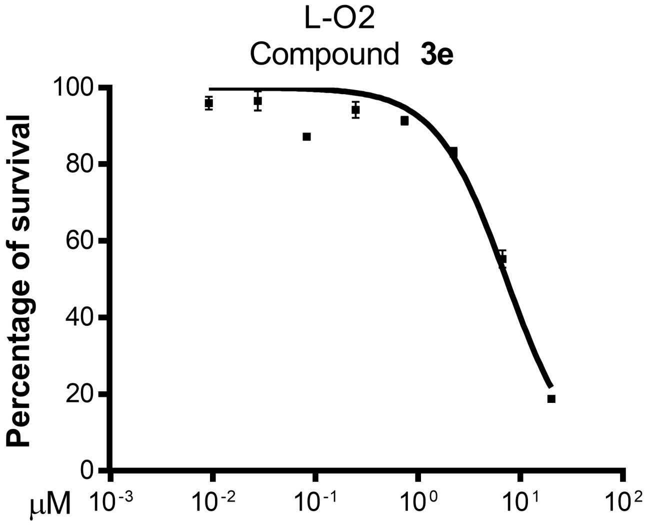
L-O2
Compound 3e
Percentage of survival
100
80
60
40
20
0
μM
10^{-3}
10^{-2}
10^{-1}
10^{0}
10^{1}
10^{2}

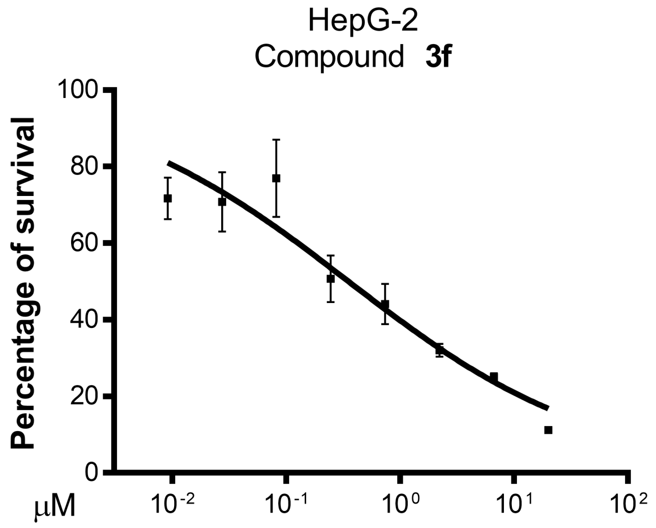
HepG-2
Compound **3f**
Percentage of survival
100
80
60
40
20
0
μM
10^{-2}
10^{-1}
10^{0}
10^{1}
10^{2}

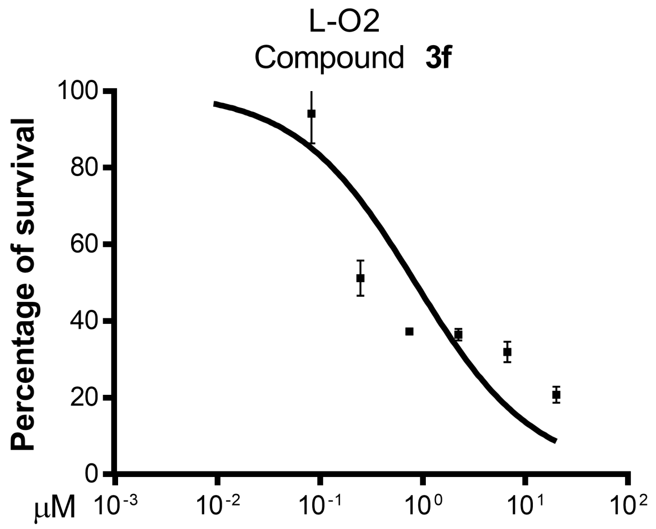
L-O2
Compound **3f**
Percentage of survival
100
80
60
40
20
0
μM
10^{-3}
10^{-2}
10^{-1}
10^{0}
10^{1}
10^{2}

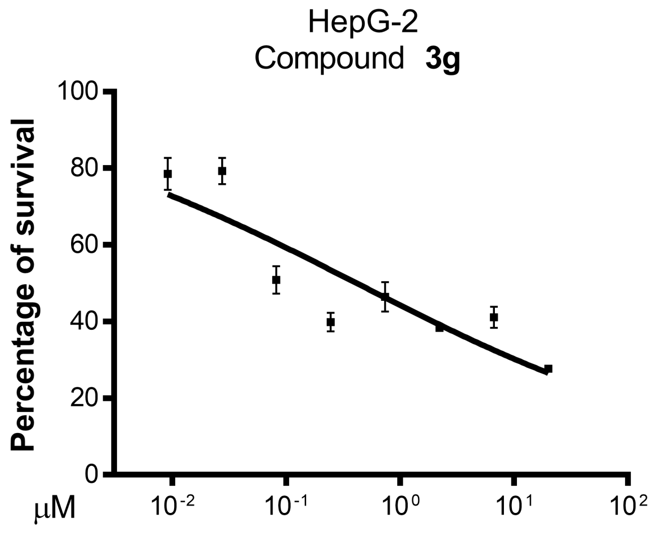
HepG-2
Compound **3g**
Percentage of survival
100
80
60
40
20
0
μM
10^{-2}
10^{-1}
10^{0}
10^{1}
10^{2}

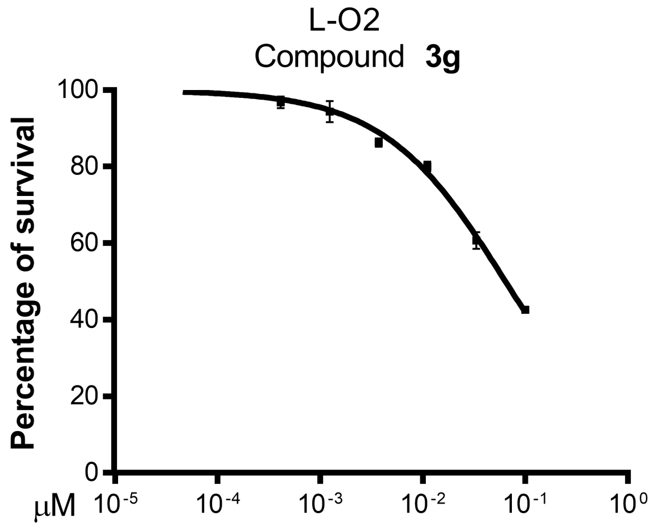
L-O2
Compound 3g
Percentage of survival
100
80
60
40
20
0
μM
10^{-5}
10^{-4}
10^{-3}
10^{-2}
10^{-1}
10^{0}

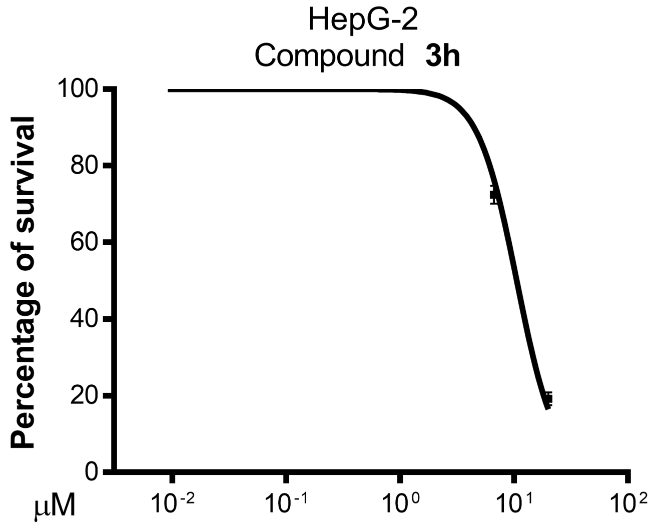
HepG-2
Compound 3h
Percentage of survival
100
80
60
40
20
0
μM
10^{-2}
10^{-1}
10^{0}
10^{1}
10^{2}

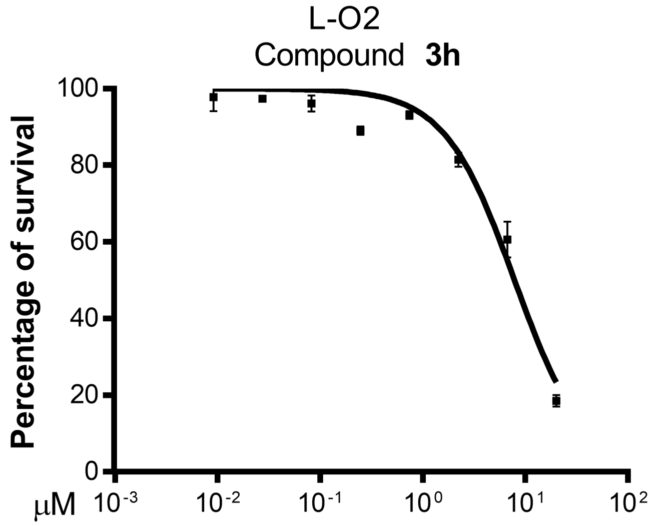
L-O2
Compound 3h
Percentage of survival
100
80
60
40
20
0
μM
10^{-3}
10^{-2}
10^{-1}
10^{0}
10^{1}
10^{2}

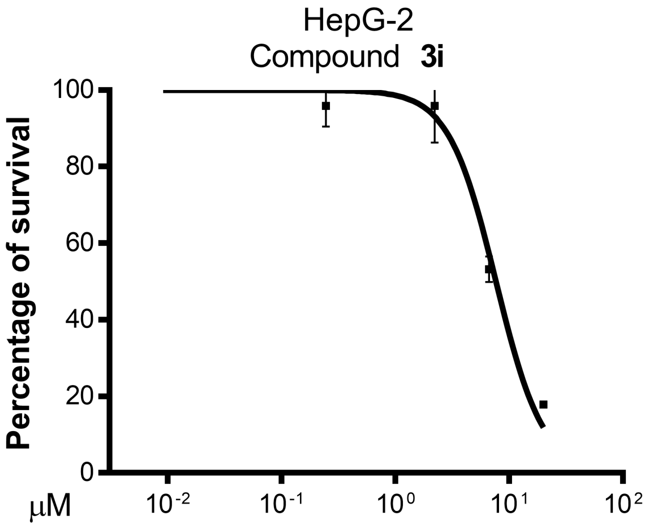
HepG-2
Compound 3i
Percentage of survival
100
80
60
40
20
0
μM
10^{-2} 10^{-1} 10^{0} 10^{1} 10^{2}

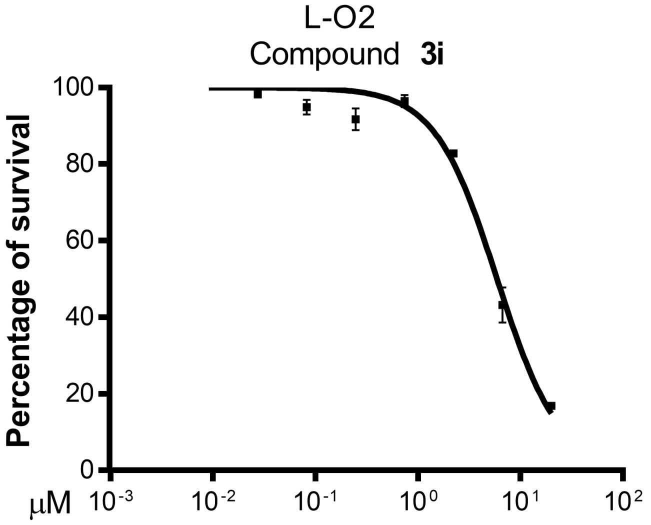
L-O2
Compound 3i
Percentage of survival
100
80
60
40
20
0
μM
10^{-3} 10^{-2} 10^{-1} 10^{0} 10^{1} 10^{2}

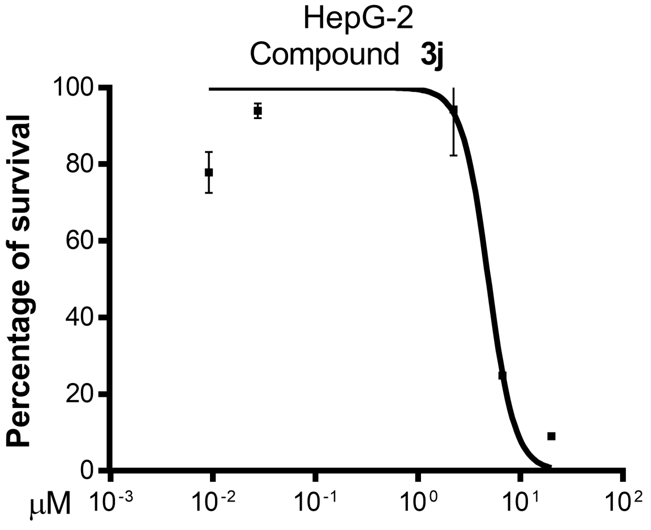
HepG-2
Compound 3j
Percentage of survival
100
80
60
40
20
0
μM
10^{-3} 10^{-2} 10^{-1} 10^{0} 10^{1} 10^{2}

L-O2
Compound **3j**

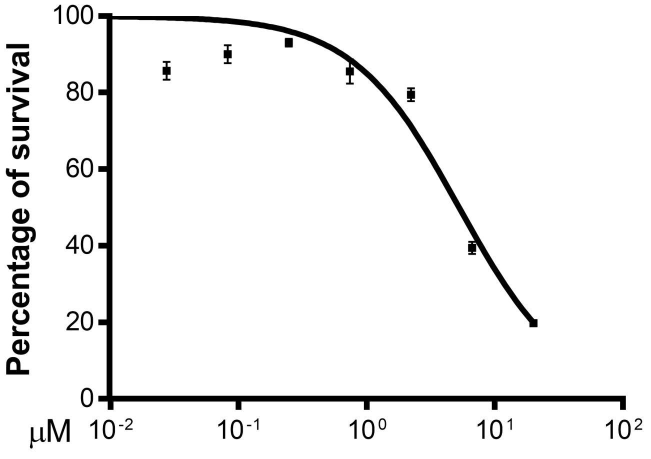

HepG-2
Compound **3k**

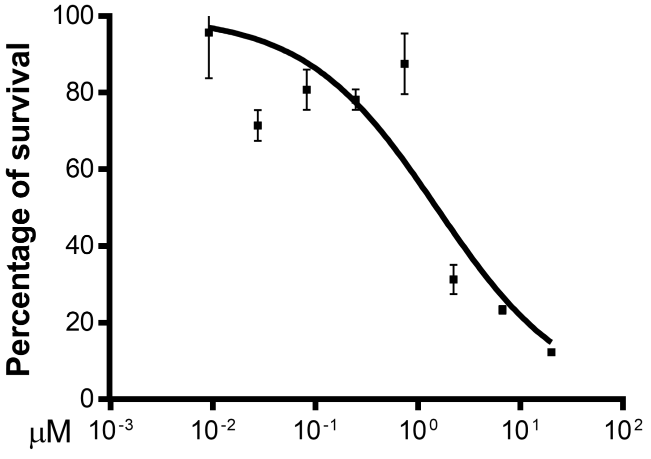

L-O2
Compound **3k**

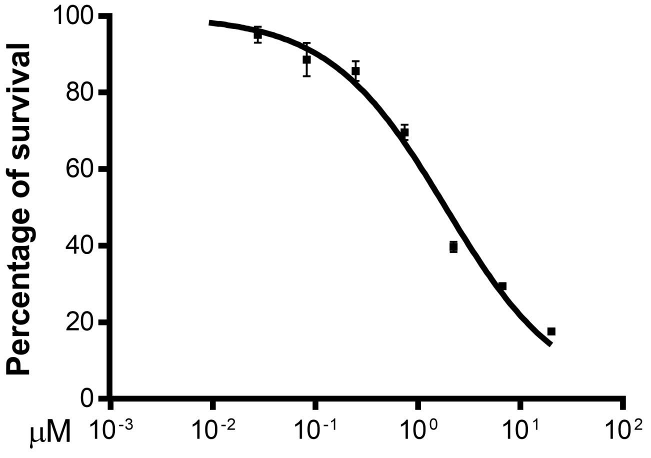

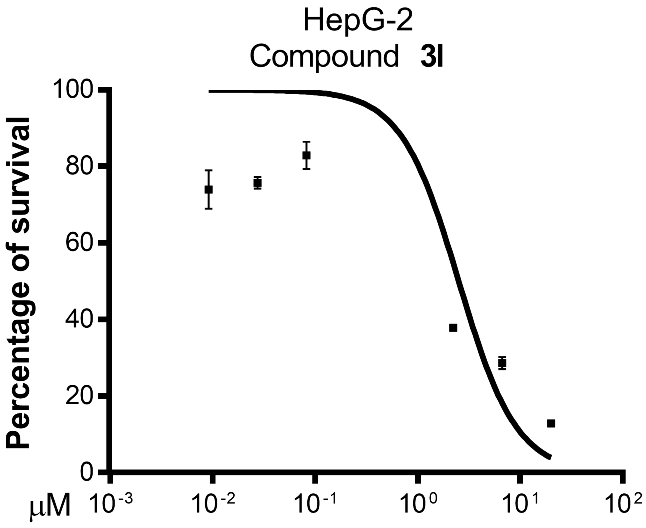
HepG-2
Compound 3l
Percentage of survival
0
20
40
60
80
100
μM
10^{-3}
10^{-2}
10^{-1}
10^{0}
10^{1}
10^{2}

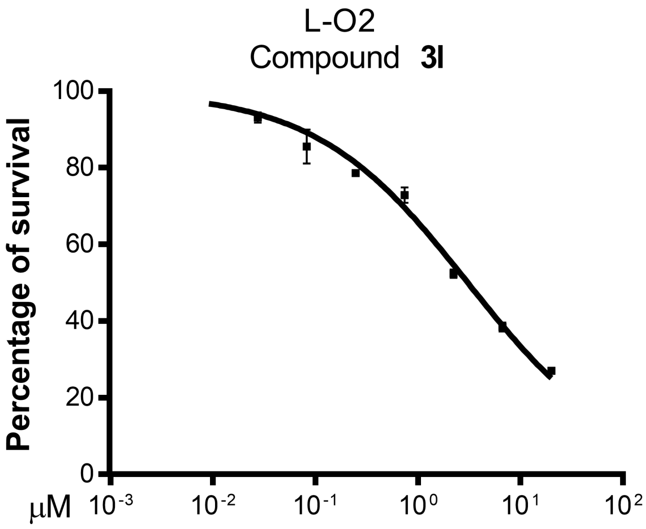
L-O2
Compound 3l
Percentage of survival
0
20
40
60
80
100
μM
10^{-3}
10^{-2}
10^{-1}
10^{0}
10^{1}
10^{2}

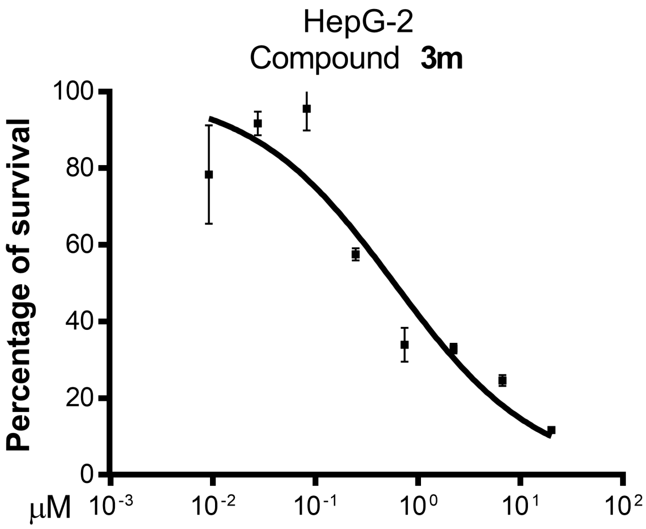
HepG-2
Compound 3m
Percentage of survival
0
20
40
60
80
100
μM
10^{-3}
10^{-2}
10^{-1}
10^{0}
10^{1}
10^{2}

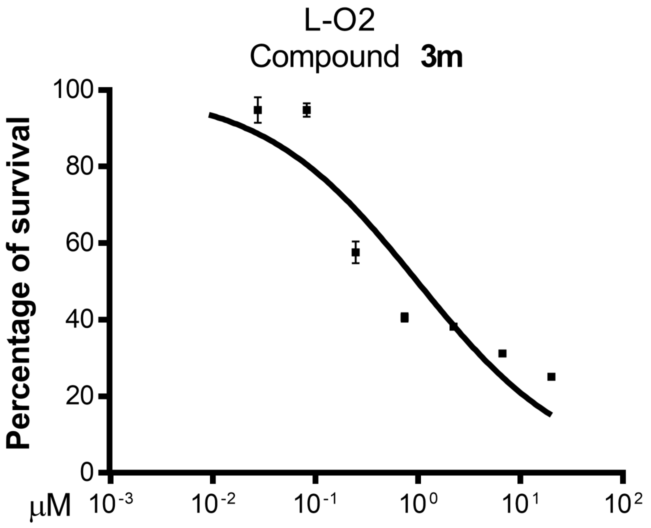
L-O2
Compound 3m
Percentage of survival
μM

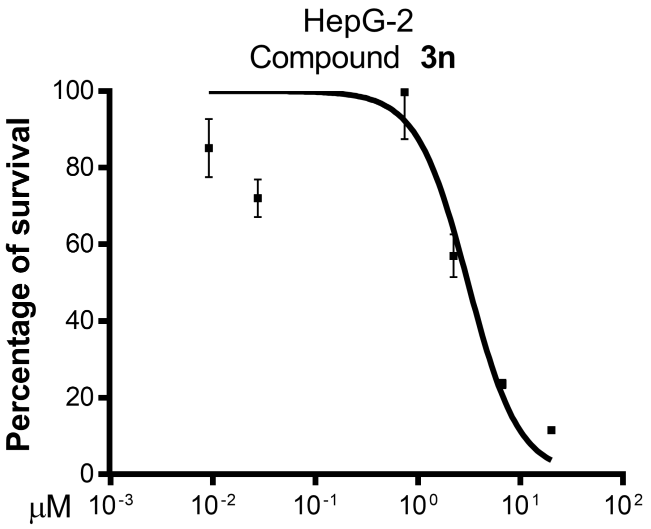
HepG-2
Compound 3n
Percentage of survival
μM

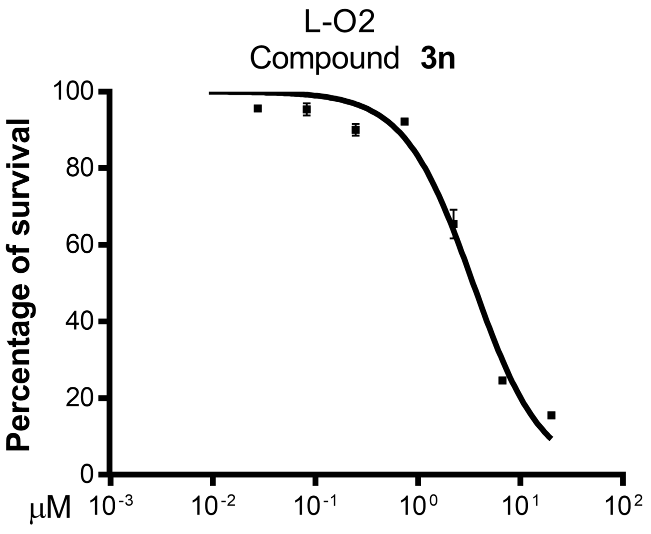
L-O2
Compound 3n
Percentage of survival
μM

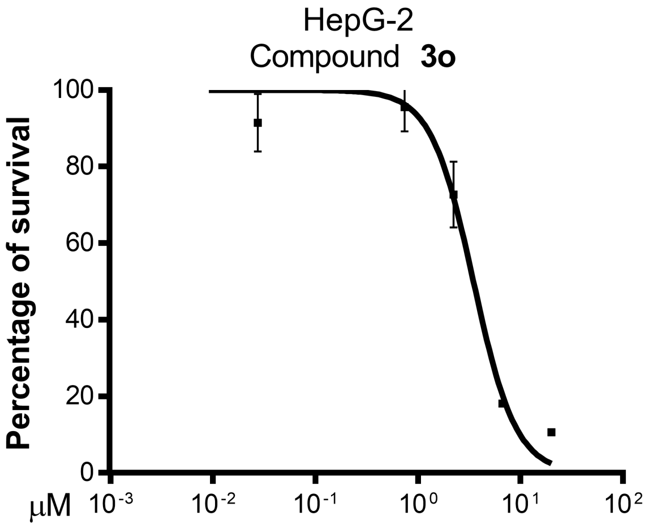

HepG-2
Compound **3o**
Percentage of survival
100
80
60
40
20
0
μM
10^{-3}
10^{-2}
10^{-1}
10^{0}
10^{1}
10^{2}

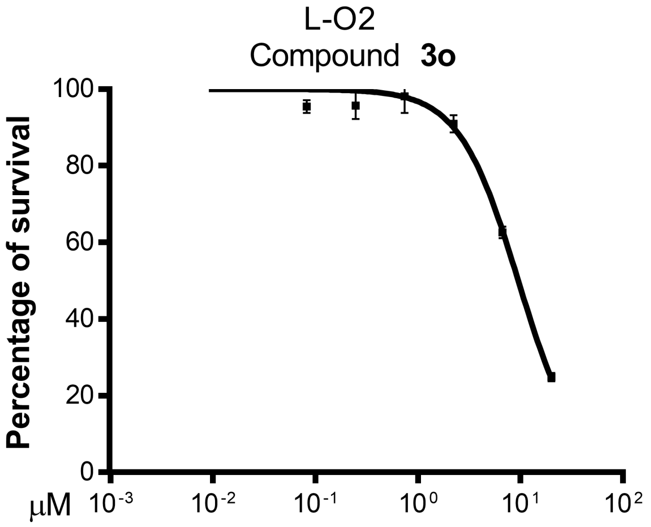

L-O2
Compound **3o**
Percentage of survival
100
80
60
40
20
0
μM
10^{-3}
10^{-2}
10^{-1}
10^{0}
10^{1}
10^{2}

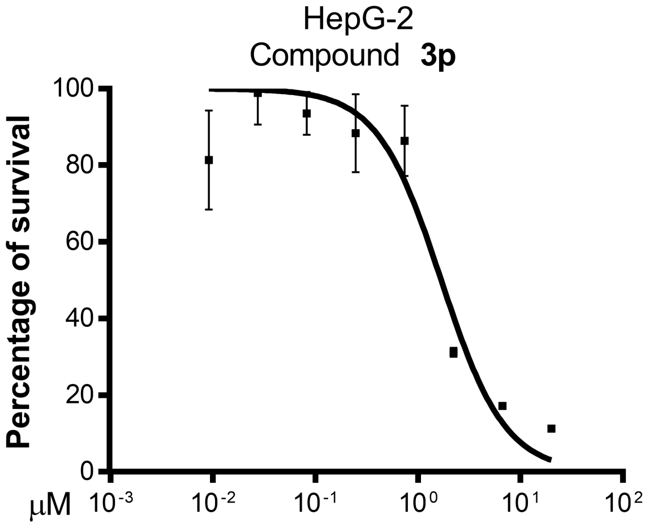

HepG-2
Compound **3p**
Percentage of survival
100
80
60
40
20
0
μM
10^{-3}
10^{-2}
10^{-1}
10^{0}
10^{1}
10^{2}

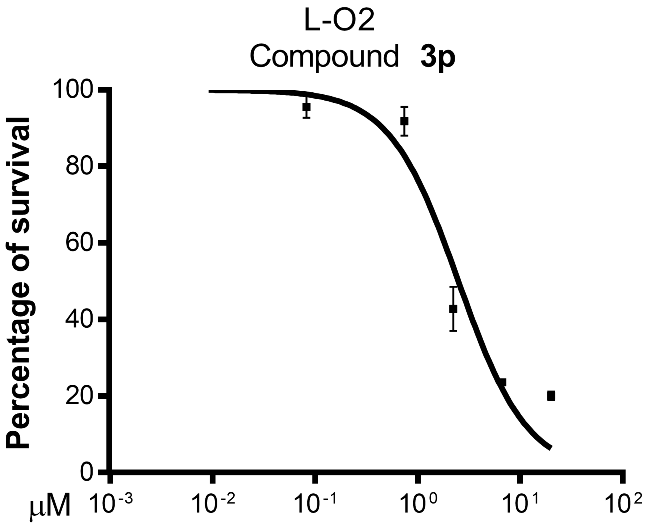
L-O2
Compound 3p
Percentage of survival
100
80
60
40
20
0
μM
10^{-3}
10^{-2}
10^{-1}
10^{0}
10^{1}
10^{2}

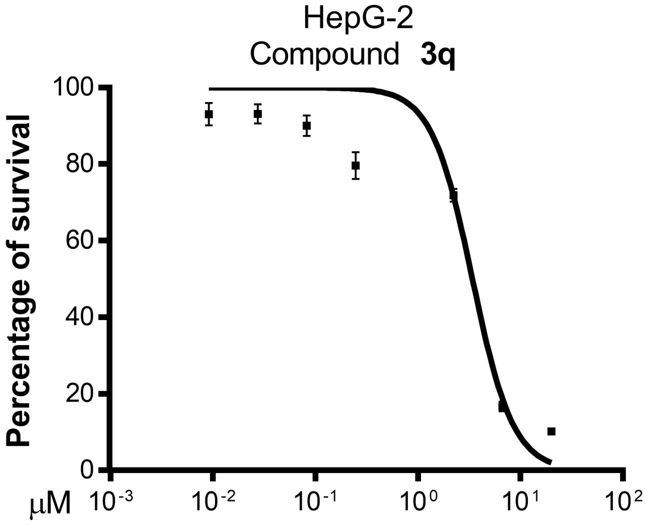
HepG-2
Compound 3q
Percentage of survival
100
80
60
40
20
0
μM
10^{-3}
10^{-2}
10^{-1}
10^{0}
10^{1}
10^{2}

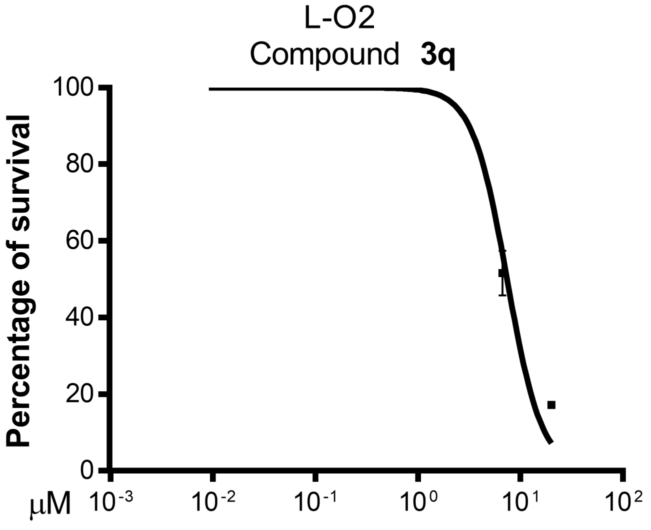
L-O2
Compound 3q
Percentage of survival
100
80
60
40
20
0
μM
10^{-3}
10^{-2}
10^{-1}
10^{0}
10^{1}
10^{2}

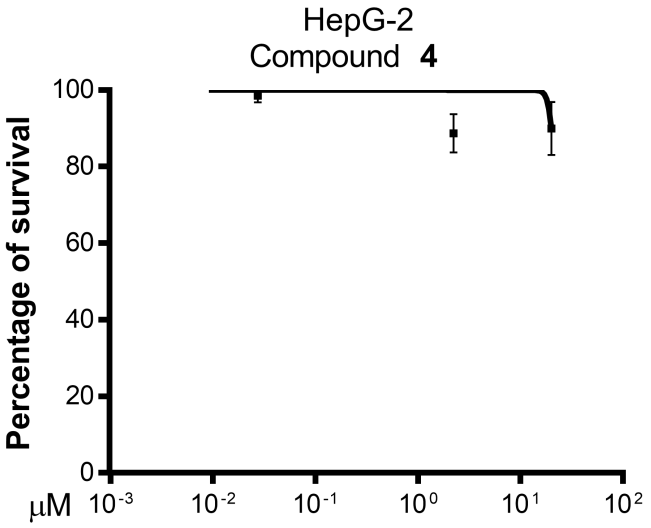
HepG-2
Compound 4
Percentage of survival
100
80
60
40
20
0
μM
10^{-3}
10^{-2}
10^{-1}
10^{0}
10^{1}
10^{2}

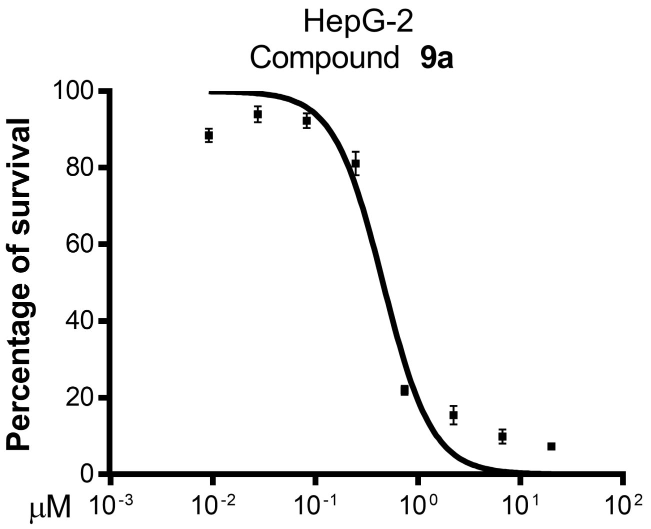
HepG-2
Compound 9a
Percentage of survival
100
80
60
40
20
0
μM
10^{-3}
10^{-2}
10^{-1}
10^{0}
10^{1}
10^{2}

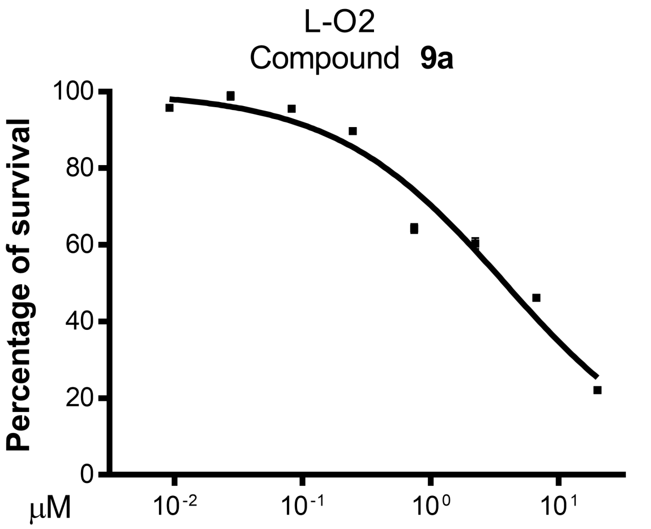
L-O2
Compound 9a
Percentage of survival
100
80
60
40
20
0
μM
10^{-2}
10^{-1}
10^{0}
10^{1}

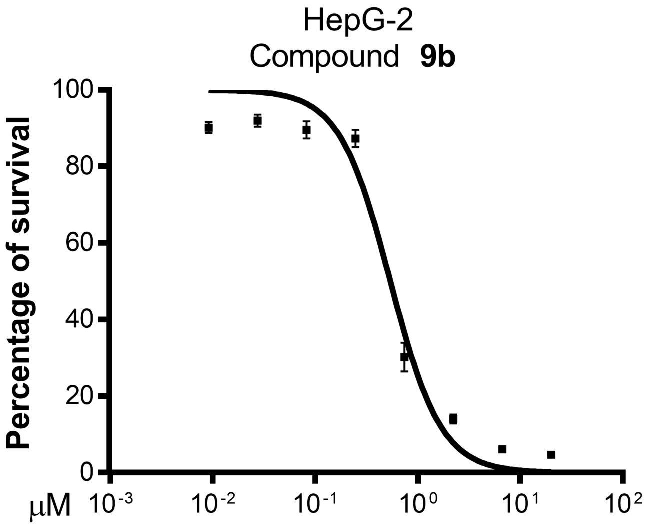
HepG-2
Compound 9b
Percentage of survival
100
80
60
40
20
0
μM
10-3
10-2
10-1
100
101
102

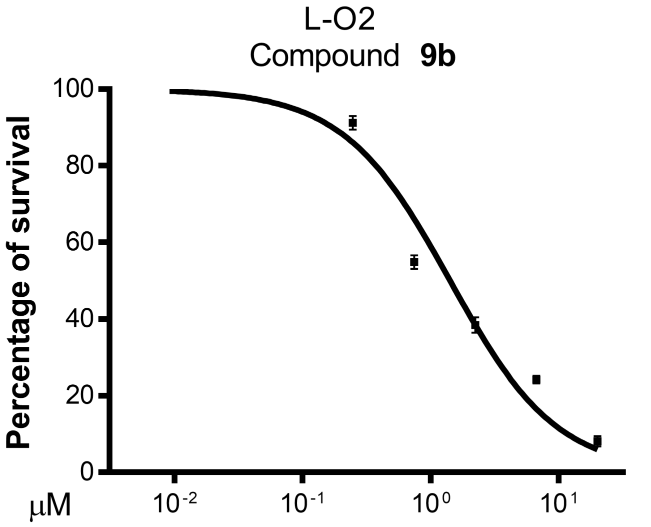
L-O2
Compound 9b
Percentage of survival
100
80
60
40
20
0
μM
10-2
10-1
100
101

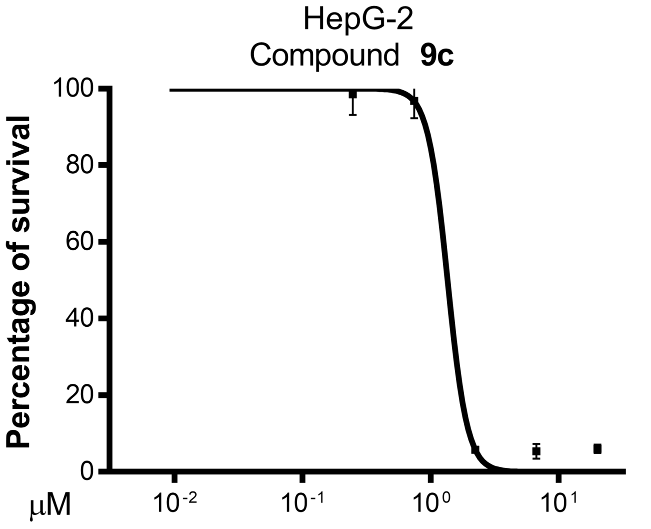
HepG-2
Compound 9c
Percentage of survival
100
80
60
40
20
0
μM
10-2
10-1
100
101

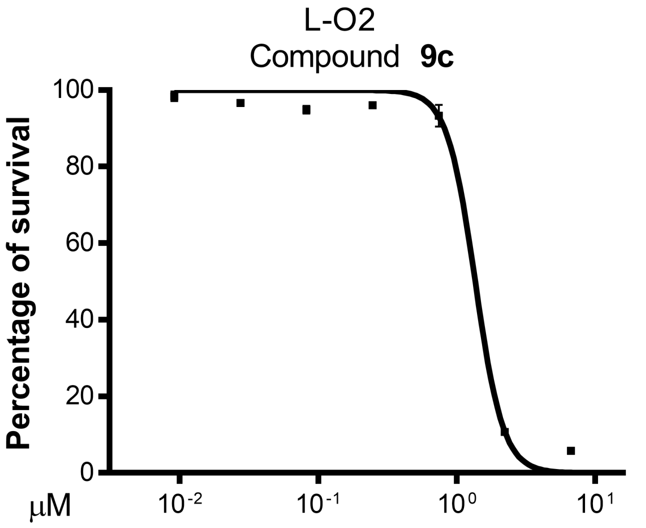
L-O2
Compound 9c
Percentage of survival
100
80
60
40
20
0
μM
10^{-2}
10^{-1}
10^{0}
10^{1}

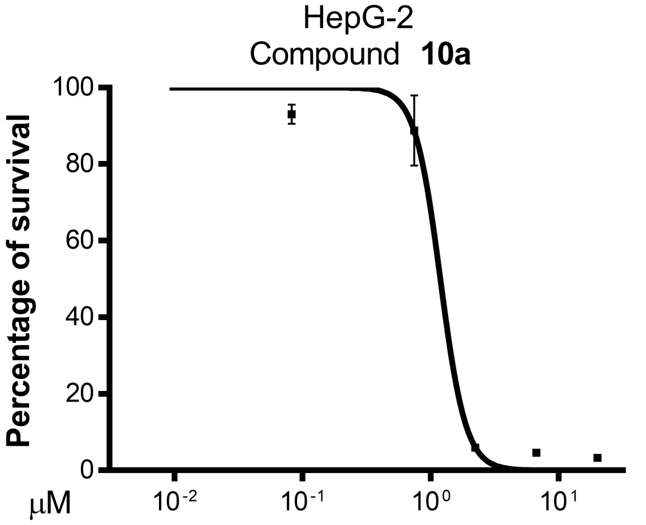
HepG-2
Compound 10a
Percentage of survival
100
80
60
40
20
0
μM
10^{-2}
10^{-1}
10^{0}
10^{1}

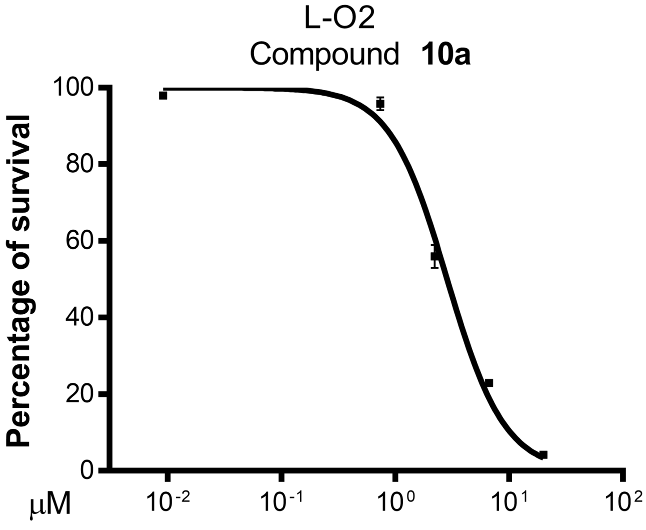
L-O2
Compound 10a
Percentage of survival
100
80
60
40
20
0
μM
10^{-2}
10^{-1}
10^{0}
10^{1}
10^{2}

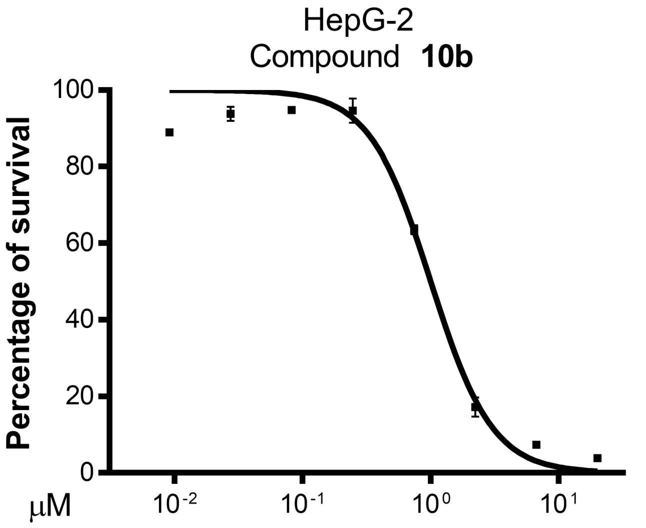
HepG-2
Compound **10b**
Percentage of survival
100
80
60
40
20
0
μM
10^{-2}
10^{-1}
10^{0}
10^{1}

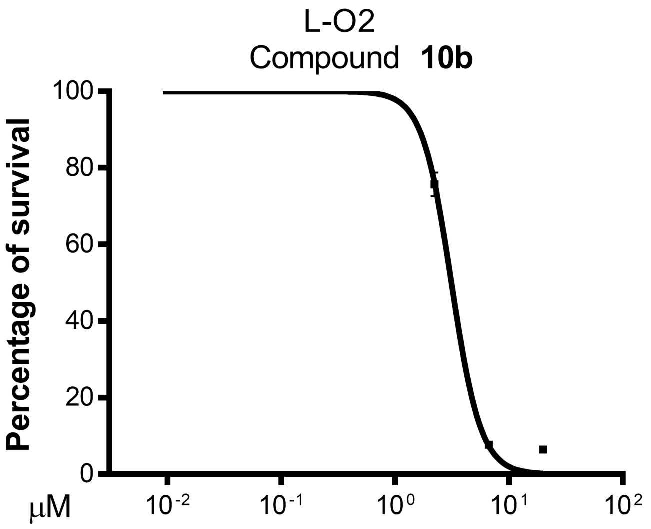
L-O2
Compound **10b**
Percentage of survival
100
80
60
40
20
0
μM
10^{-2}
10^{-1}
10^{0}
10^{1}
10^{2}

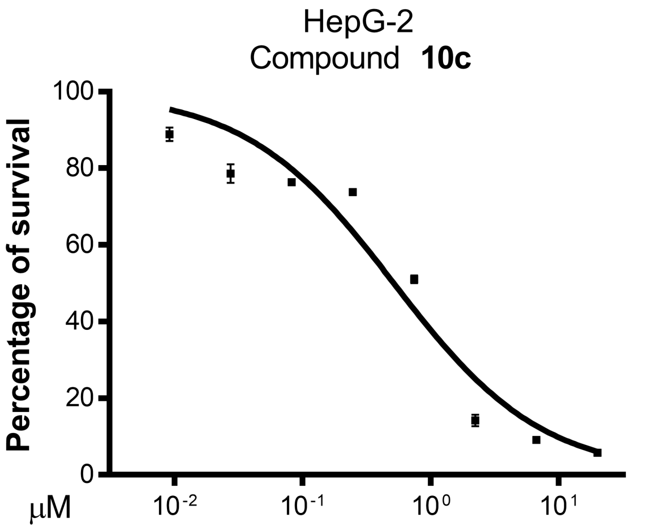
HepG-2
Compound **10c**
Percentage of survival
100
80
60
40
20
0
μM
10^{-2}
10^{-1}
10^{0}
10^{1}

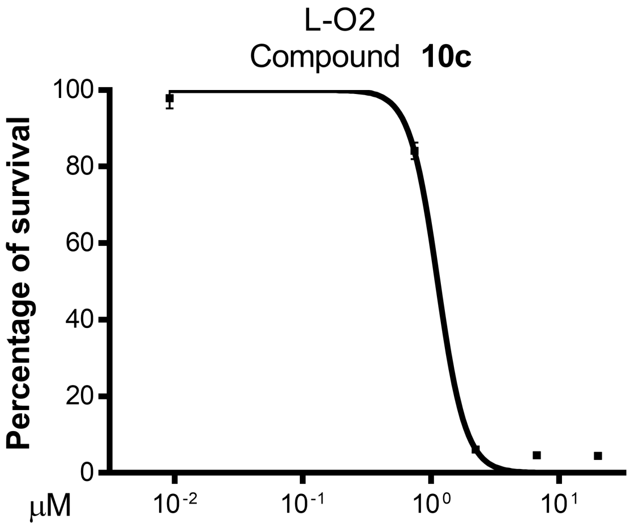
L-O2
Compound **10c**
Percentage of survival
100
80
60
40
20
0
μM
10^{-2}
10^{-1}
10^{0}
10^{1}

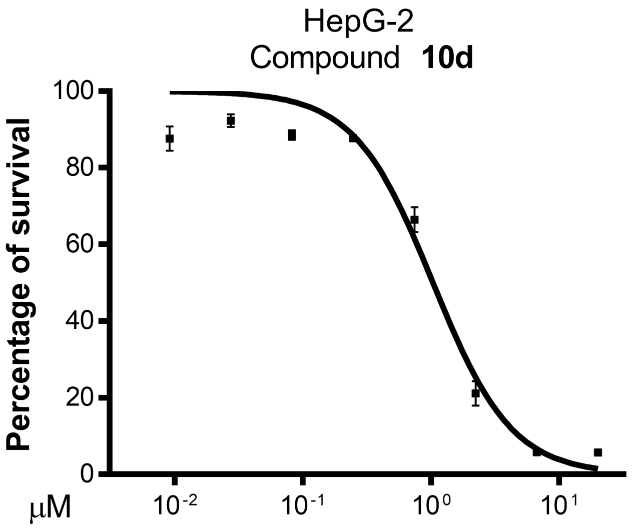
HepG-2
Compound **10d**
Percentage of survival
100
80
60
40
20
0
μM
10^{-2}
10^{-1}
10^{0}
10^{1}

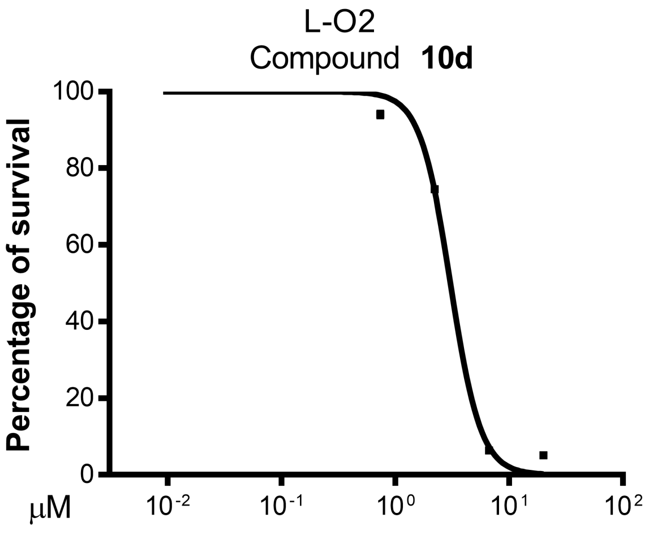
L-O2
Compound **10d**
Percentage of survival
100
80
60
40
20
0
μM
10^{-2}
10^{-1}
10^{0}
10^{1}
10^{2}

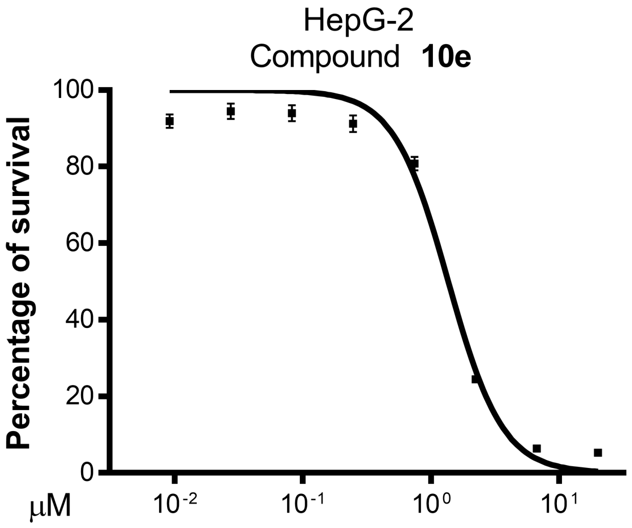
HepG-2
Compound 10e
Percentage of survival
100
80
60
40
20
0
μM
10^{-2}
10^{-1}
10^{0}
10^{1}

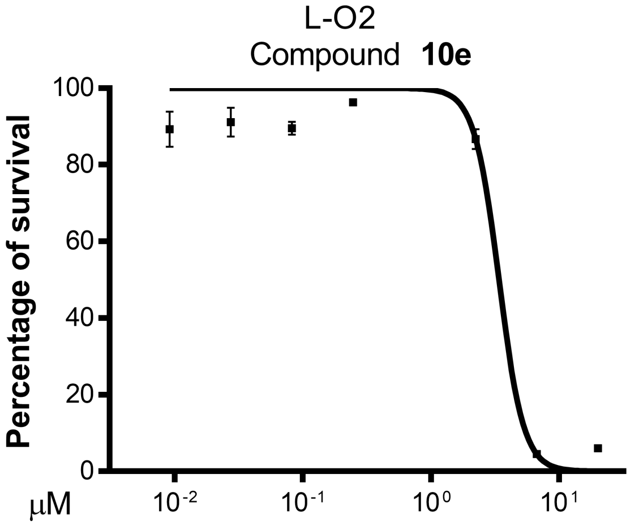
L-O2
Compound 10e
Percentage of survival
100
80
60
40
20
0
μM
10^{-2}
10^{-1}
10^{0}
10^{1}

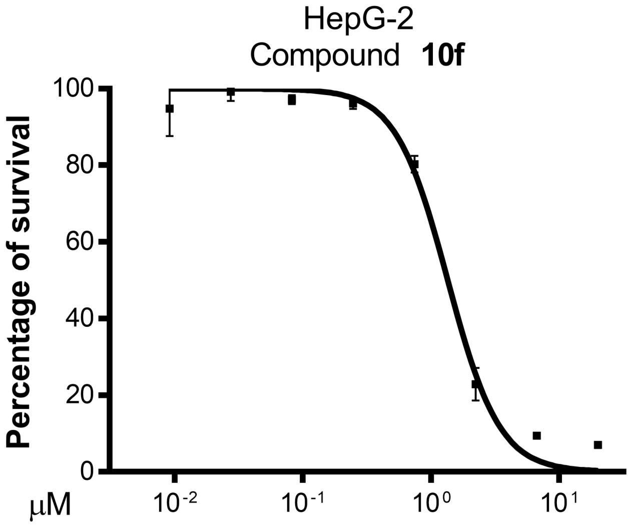
HepG-2
Compound 10f
Percentage of survival
100
80
60
40
20
0
μM
10^{-2}
10^{-1}
10^{0}
10^{1}

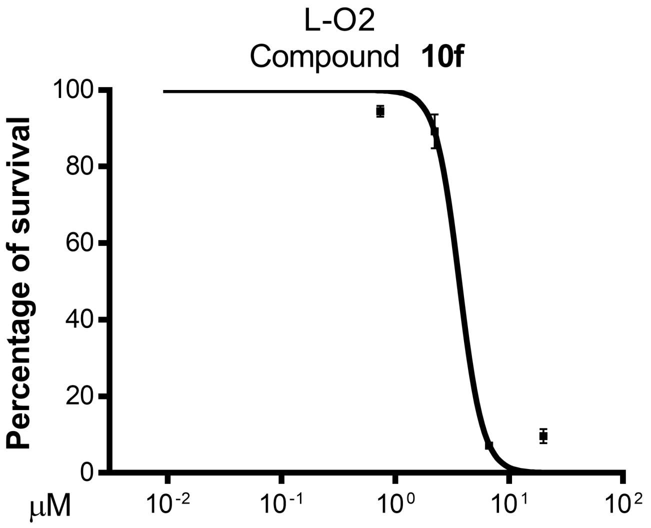
L-O2
Compound 10f
Percentage of survival
100
80
60
40
20
0
μM
10^{-2}
10^{-1}
10^{0}
10^{1}
10^{2}

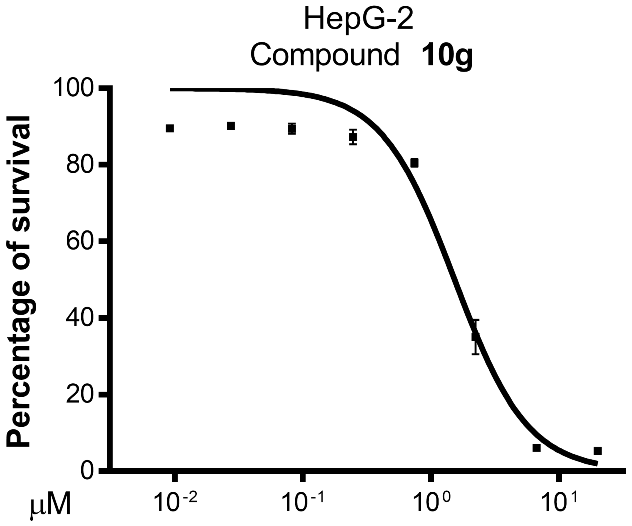
HepG-2
Compound 10g
Percentage of survival
100
80
60
40
20
0
μM
10^{-2}
10^{-1}
10^{0}
10^{1}

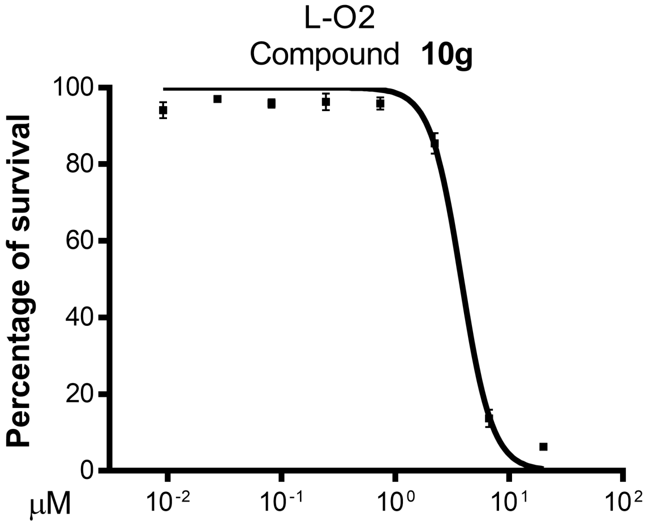
L-O2
Compound 10g
Percentage of survival
100
80
60
40
20
0
μM
10^{-2}
10^{-1}
10^{0}
10^{1}
10^{2}

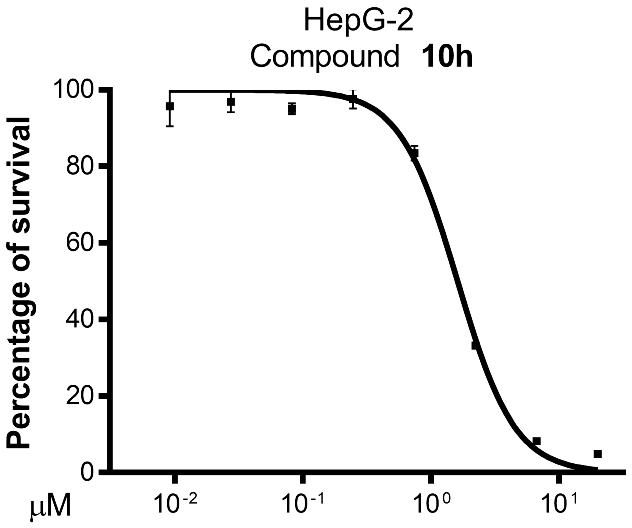
HepG-2
Compound **10h**
Percentage of survival
100
80
60
40
20
0
μM
10^{-2}
10^{-1}
10^{0}
10^{1}

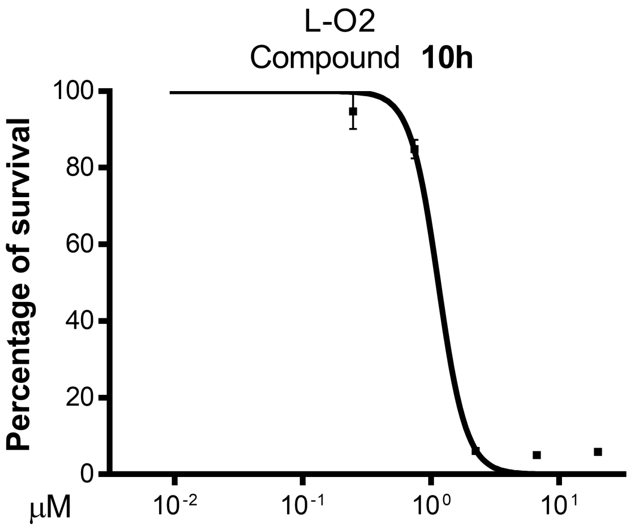
L-O2
Compound **10h**
Percentage of survival
100
80
60
40
20
0
μM
10^{-2}
10^{-1}
10^{0}
10^{1}

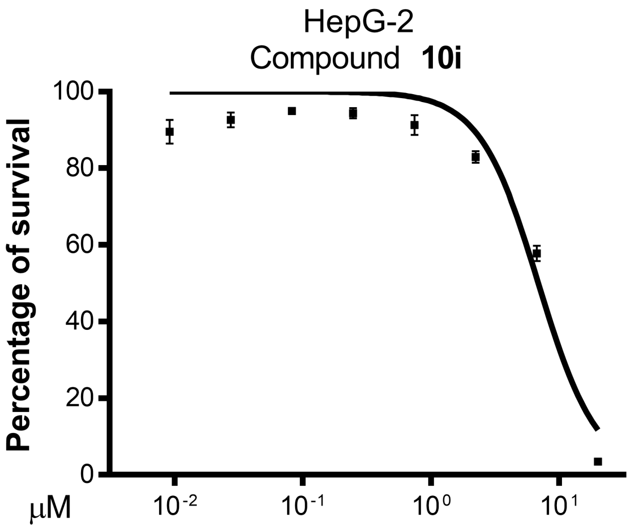
HepG-2
Compound **10i**
Percentage of survival
100
80
60
40
20
0
μM
10^{-2}
10^{-1}
10^{0}
10^{1}

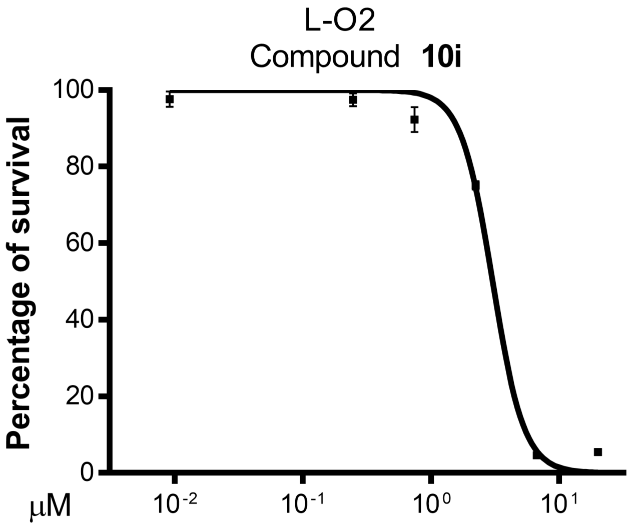

L-O2
Compound **10i**
Percentage of survival
100
80
60
40
20
0
μM
10^{-2}
10^{-1}
10^{0}
10^{1}

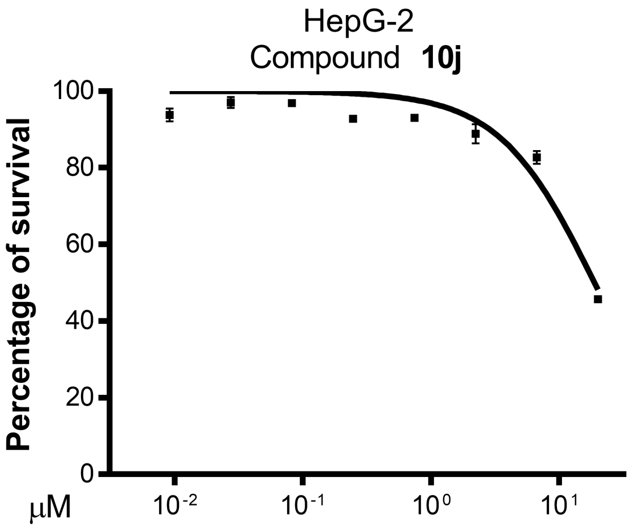

HepG-2
Compound **10j**
Percentage of survival
100
80
60
40
20
0
μM
10^{-2}
10^{-1}
10^{0}
10^{1}

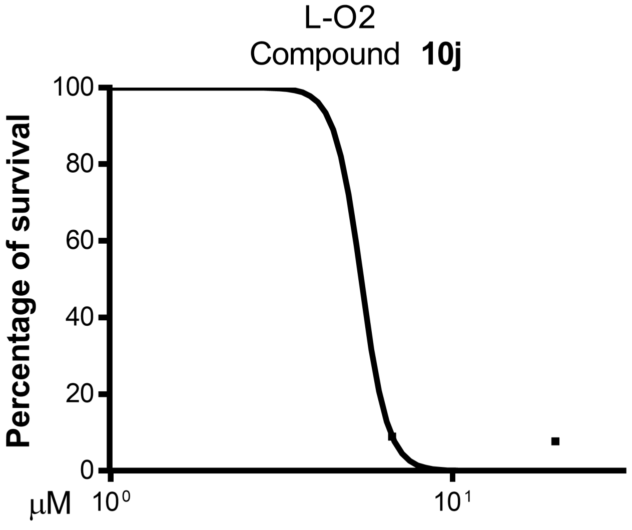

L-O2
Compound **10j**
Percentage of survival
100
80
60
40
20
0
μM
10^{0}
10^{1}

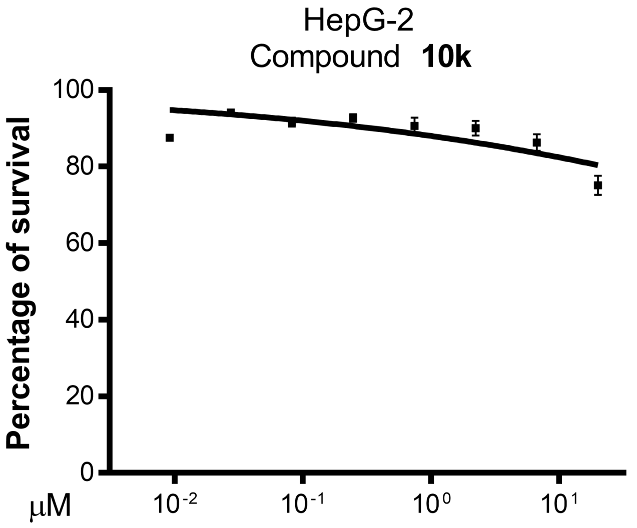
HepG-2
Compound **10k**
Percentage of survival
100
80
60
40
20
0
μM
10^{-2}
10^{-1}
10^{0}
10^{1}

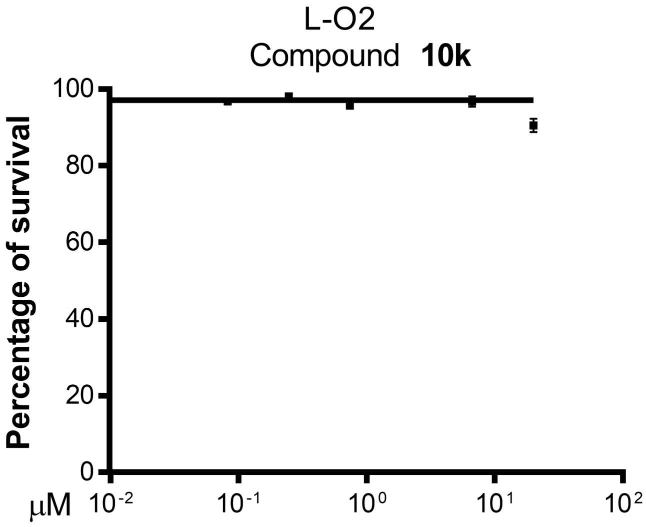
L-O2
Compound **10k**
Percentage of survival
100
80
60
40
20
0
μM
10^{-2}
10^{-1}
10^{0}
10^{1}
10^{2}

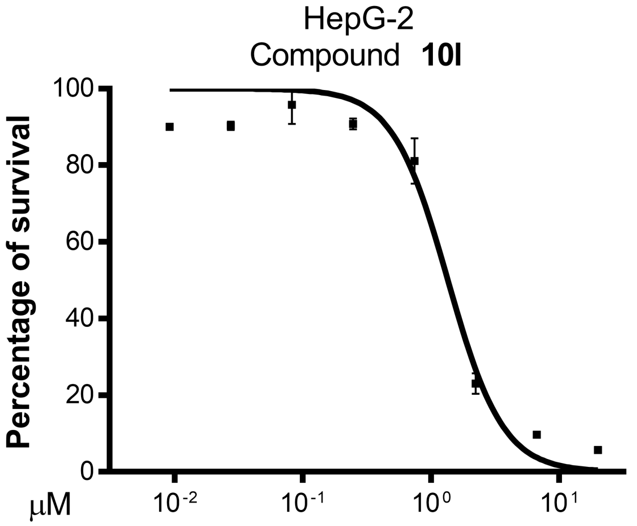
HepG-2
Compound **10l**
Percentage of survival
100
80
60
40
20
0
μM
10^{-2}
10^{-1}
10^{0}
10^{1}

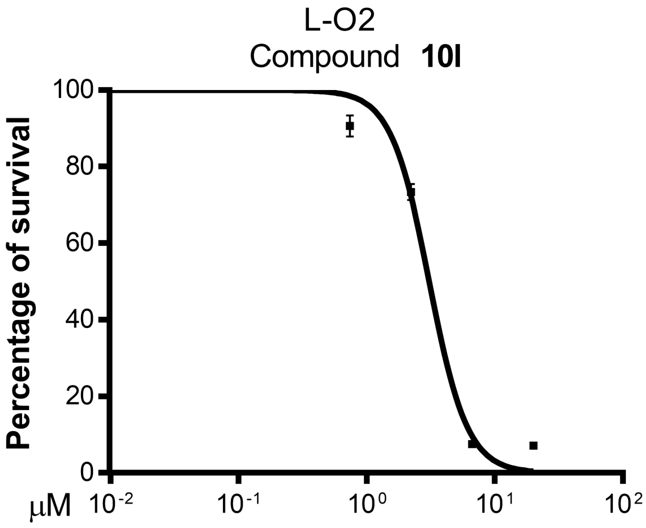
L-O2
Compound 10l
Percentage of survival
100
80
60
40
20
0
μM
10^{-2}
10^{-1}
10^{0}
10^{1}
10^{2}

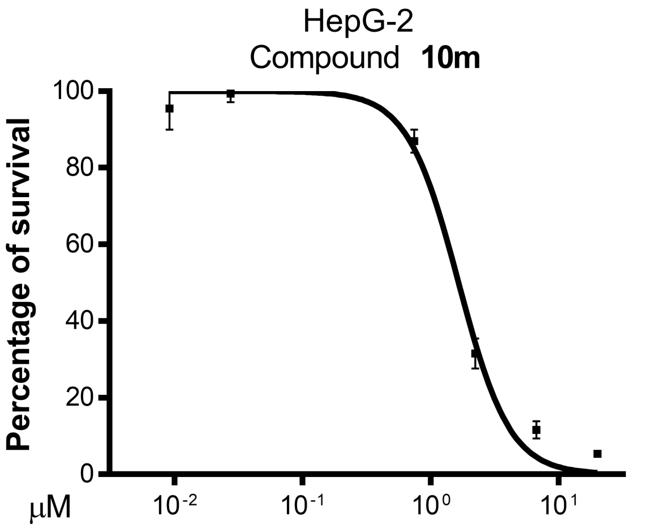
HepG-2
Compound 10m
Percentage of survival
100
80
60
40
20
0
μM
10^{-2}
10^{-1}
10^{0}
10^{1}

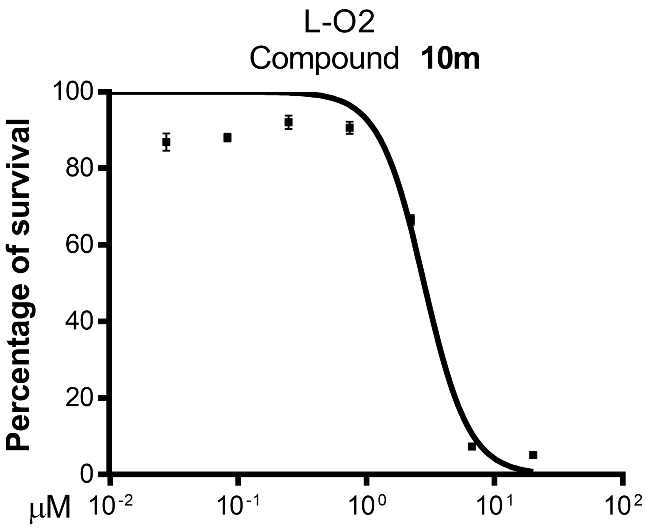
L-O2
Compound 10m
Percentage of survival
100
80
60
40
20
0
μM
10^{-2}
10^{-1}
10^{0}
10^{1}
10^{2}

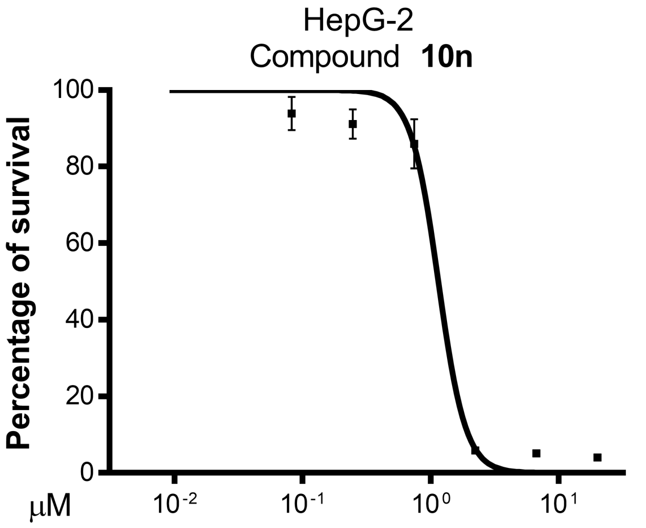
HepG-2
Compound **10n**
Percentage of survival
100
80
60
40
20
0
μM
10^{-2}
10^{-1}
10^{0}
10^{1}

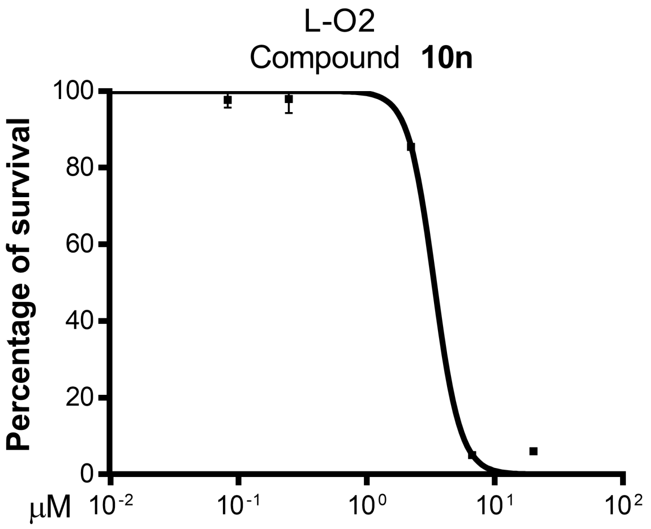
L-O2
Compound **10n**
Percentage of survival
100
80
60
40
20
0
μM
10^{-2}
10^{-1}
10^{0}
10^{1}
10^{2}

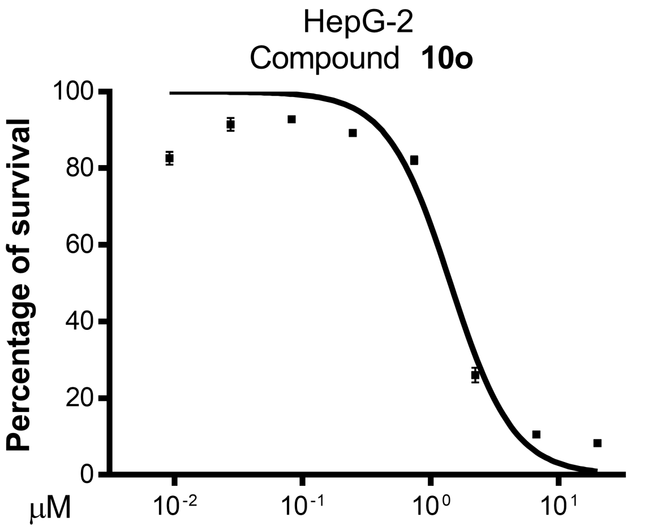
HepG-2
Compound **10o**
Percentage of survival
100
80
60
40
20
0
μM
10^{-2}
10^{-1}
10^{0}
10^{1}

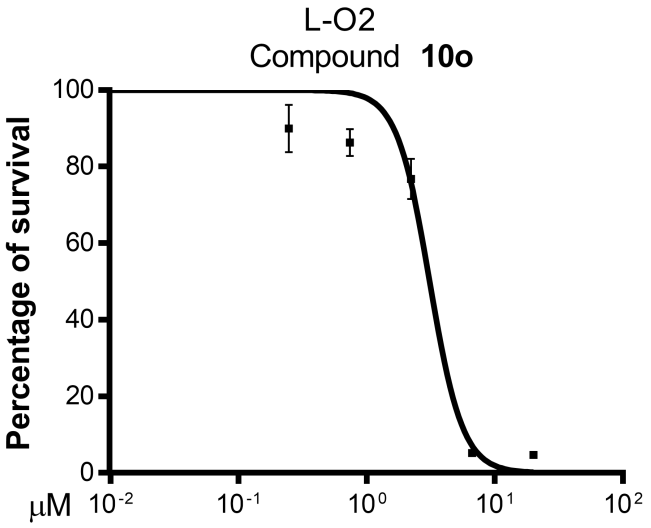

L-O2
Compound **10o**
100
80
60
40
20
0
Percentage of survival
μM
10^{-2}
10^{-1}
10^{0}
10^{1}
10^{2}

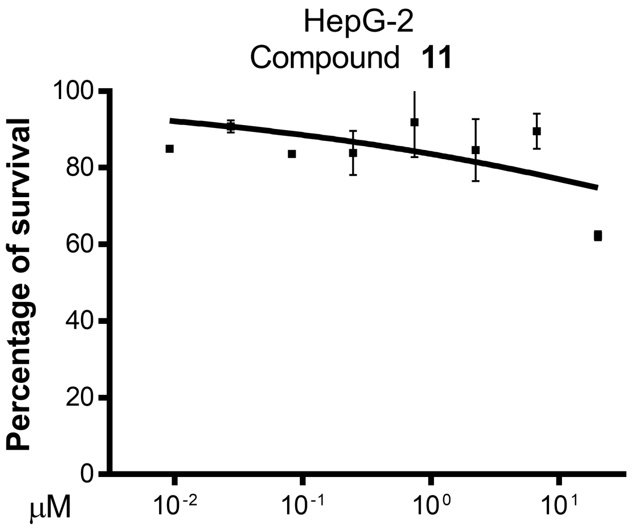

HepG-2
Compound **11**
100
80
60
40
20
0
Percentage of survival
μM
10^{-2}
10^{-1}
10^{0}
10^{1}
